# Education, intelligence, and 20 gastrointestinal disorders: A Mendelian randomization study

**DOI:** 10.1097/MD.0000000000040825

**Published:** 2024-12-06

**Authors:** Jun He, Yunzhi Lin, Zhen Ding

**Affiliations:** a Hepatobiliary Surgery, Chaohu Hospital of Anhui Medical University, Hefei, China.

**Keywords:** education attainment, gastrointestinal diseases, intelligence, liver, Mendelian randomization, pancreas

## Abstract

Previous observational studies have suggested that higher levels of education attainment and intelligence (IQ) are associated with better health outcomes in humans. However, the causal link between education attainment and IQ and their association with health outcomes remains unclear. This study aims to investigate the distinct impacts of intelligence and educational attainment on gastrointestinal symptoms. From the genome-wide association between educational attainment and the IQ study database, results were obtained from the FinnGen summary database. We used univariate and multivariate Mendelian randomization (MR) techniques to explore the relationship between exposures and outcomes. To assess the validity of inverse-variance-weighted-based results, we used several supplementary analytical techniques and performed sensitivity analysis. Our multivariate MR study confirmed the findings from univariable analyses and showed a genetically predicted causal association between educational attainment and 8 gastrointestinal disorders, including gastroesophageal reflux disease, chronic gastritis, gastroduodenal ulcer, cirrhosis, cholelithiasis, acute pancreatitis, chronic pancreatitis, and irritable bowel syndrome. Our univariate MR study found an association between IQ and 6 gastrointestinal conditions: gastroesophageal reflux disease, cirrhosis, cholelithiasis, acute pancreatitis, pancreatic malignancy, and irritable bowel syndrome. However, the connection was much weaker in multivariate MR analysis. Our study revealed causal relationships between gastrointestinal disorders and educational attainment. Educational attainment may mediate between intelligence and the impacts on the gastrointestinal system. However, further research is required to understand the underlying pathogenic processes completely.

## 1. Introduction

Research on various gastrointestinal illnesses has recently attracted interest from the medical community. According to a recent study, gastrointestinal, liver, and pancreatic illnesses account for a significant amount of healthcare spending in the United States, costing the country billions of dollars annually, and severely impair both people’s quality of life and the economy of the country.^[1]^ Therefore, it is essential to understand the causes and prevention of gastrointestinal disorders. Recently, numerous investigations have been conducted on the causes and preventative elements of gastrointestinal illnesses. Examples include studies on diet,^[2]^ smoking,^[3]^ alcohol consumption,^[4]^ digestive enzyme supplementation,^[5]^ and antioxidant supplementation.^[6]^ The link between low educational attainment (EA) and an elevated risk of liver disease has been clearly shown in a study on 11,107 Italian participants, underscoring the importance of EA in human health.^[7]^ Lower educational attainment was linked to an increased chance of developing esophageal, stomach, colon, and other cancers, according to an 8.2-year follow-up research.^[8]^ Additionally, a meta-analysis of 16 prospective studies revealed a significant correlation between higher intelligence (IQ) and decreased mortality. However, the full realization of the biological mechanisms of this nexus will require further research.^[9]^ Investigating the relationship between intellect and health consequences has long piqued the curiosity of researchers.^[9–11]^ These investigations imply that high IQ may enhance health, although several variables may affect this association.

Earlier observational studies may have been biased and confounded by other factors, such as reverse causality. The limitations of randomized controlled trials include the significant time and financial resources required, and strict inclusion and exclusion criteria that may limit the applicability of research findings in real-world contexts. Additionally, randomized controlled trials are only appropriate for intervention research and cannot be used for etiology or natural history studies, while the improper use of a placebo or risk factors can violate medical ethics.^[12–14]^ Mendelian randomization (MR) studies use genetic variations as exposures to examine the association between phenotype and disease to address these limitations, while avoiding the reverse causation connection and bias of confounding factors.^[15,16]^

Advances in MR research have significantly advanced due to the introduction of large-scale genome-wide association studies (GWAS), which have allowed the identification of related genetic variations and single nucleotide polymorphisms (SNPs) through DNA and whole-genome sequencing. EA and IQ have previously been linked to health disorders, including COVID-19,^[17]^ cardiometabolic factors,^[18]^ and Alzheimer disease.^[19]^ The MR analysis of gastrointestinal illnesses has received less attention, nevertheless. In this work, we used GWAS datasets to explore the causal relationship between EA and IQ on 20 gastrointestinal illnesses using MR analysis. It is important to note that prior research has shown a causal relationship between EA and IQ,^[20]^ with more education years being linked to higher IQ and vice versa. Our study builds upon and expands on this previous research by investigating the possible causal connection between EA and IQ with 20 gastrointestinal illnesses using MR analysis.^[21]^ Our study offers valuable data to complement and extend the prior findings.

## 2. Materials and methods

In MR analysis, 3 instrumental variable assumptions are essential for establishing causality.^[22,23]^ The first assumption asserts that the instrumental variables used in this study were related to exposure of interest. The second assumption seeks to minimize the impact of confounding factors on the relationship between the instrumental variables and outcomes. The third assumption requires that exposure to interest is the only factor influencing the relationship between the instrumental variables and the outcome. It is crucial to satisfy these IV assumptions to ensure the validity of causal inference in MR analysis. The GWAS data used in this study were sourced from publicly available databases and can be obtained from appropriate websites. All participants in the current study completed an informed consent form after receiving ethical permission from the relevant ethics committee. Ethical approval was not required because no participants’ primary data were used in this study.

### 2.1. Selection of exposure data and instrument variables

The Lee et al study served as the basis for the instrument variable for EA.^[24]^ The GWAS for EA was measured by the years of education completion, using the International Standard Classification of Education to construct variables to exclude heterogeneity between samples, with the minimum age of participants being 30 years old.^[25]^ We set *P* < 5E‐08 and removed linkage disequilibrium (clump *r*^2^ < 0.001 and clump distance > 10,000 kb) before choosing 317 SNPs from the 766,345-person sample population as the relevant instrumental variables (Table S11, Supplemental Digital Content, http://links.lww.com/MD/O101). The instrumental variables for IQ were derived from a large genetic association study of intelligence in a total of 269,867 samples from 14 European pedigree cohorts,^[26]^ which determined that intelligence is highly genetic and ultimately screened for 165 SNPs as instrument variables for IQ. If the filtered instrumental variable is not included in the results, it is excluded from further analysis. To be sure that the effects of these SNPs on exposure matched the same alleles as the effects on outcome, we also deleted the allele frequencies of the palindromic SNPs (the same allele appears in the positive and negative chains) (Table S11, Supplemental Digital Content, http://links.lww.com/MD/O101).

### 2.2. Data sources for the results

The 20 gastrointestinal conditions that were the subject of this study’s examination were found in the FinnGen database (Table S11, Supplemental Digital Content, http://links.lww.com/MD/O101, RRID: SCR_022254), a sizable biospecimen repository created to study the genetics and health information of the Finnish population. By examining the Finnish population’s genetic information and health data, FinnGen aims to identify genes linked to illnesses and probable causal factors. Due to its outstanding public healthcare system and reliable diagnostic equipment, the FinnGen database is a useful instrument for researching the relationship between genetics and disease. Since 1969, information from the Finnish population’s national health registration has been gathered and connected to unique registry numbers, enabling a deeper understanding of the connection between genetics and health.^[27,28]^ We used GWAS data from the FinnGen database, which was necessary to investigate 20 gastrointestinal illnesses. There was no sample overlap between the exposure and results from the sample groups used for this study, which were of European ethnic background.

### 2.3. MR analysis

We use MR analysis to investigate the causal relationships between EA, IQ, and 20 gastrointestinal diseases. Our main analytical technique is the multiplicative random effects inverse-variance weighted (IVW) method, which employs weighted linear regression under the assumption of mutual independence among the chosen instrumental variables. To ensure the robustness of the IVW method, we used MR Egger and weighted median methods as supplementary methods.^[29,30]^ The multiplicity of instrumental variables was estimated using the MR-Egger technique, which was adjusted for IVW by including an intercept component to account for potential bias caused by horizontal pleiotropy.^[31]^ Cochran Q value was used to evaluate heterogeneity in the IVW approach, and heterogeneity between instrumental variables was considered at *P* < .05.^[32]^ We used the MR-PRESSO software to identify and exclude aberrant SNPs that could impact the outcomes of the MR analysis, to address possible concerns of heterogeneity and horizontal pleiotropy among instrumental variables.^[33]^ Multivariate MR imaging was used to analyze the reciprocal adjustment between EA and IQ to determine their individual effects on gastrointestinal disorders.^[34]^ To assess the robustness of the IVW approach, the MR-Lasso and MR-Robust were employed as additional multivariate analytic methods.^[35]^ Furthermore, to eliminate bias from weak instrumental factors, we evaluated the strength of the instrumental variables using *F*-value statistics, computed as β²/SE², and omitted those with *F*-values <10.^[36,37]^ All statistical analyses were conducted using R software (RRID: SCR_000432) and various packages, including TwoSampleMR, MR-PRESSO, and Mendelian randomization packages.^[33,38,39]^ To exclude false positive results, we used the false-positive rate (FDR) method to adjust the *P*-value in the univariate analyses, and FDR-adjusted *P*-value under .05 was considered statistically significant in observational analyses.

## 3. Results

After computation, the *F*-values of the chosen instrumental variables were >10, indicating no weak instrumental variable bias. The instrumental variable F-statistic values ranged from EA and 29 to 133 for IQ (Table S1, Supplemental Digital Content, http://links.lww.com/MD/O101, Table S2, Supplemental Digital Content, http://links.lww.com/MD/O101). See Table S3, Supplemental Digital Content, http://links.lww.com/MD/O101 and Table S4, Supplemental Digital Content, http://links.lww.com/MD/O101 for additional information on the instrumental variables in the outcomes.

### 3.1. EA and gastrointestinal disorders

**Figure 1.**
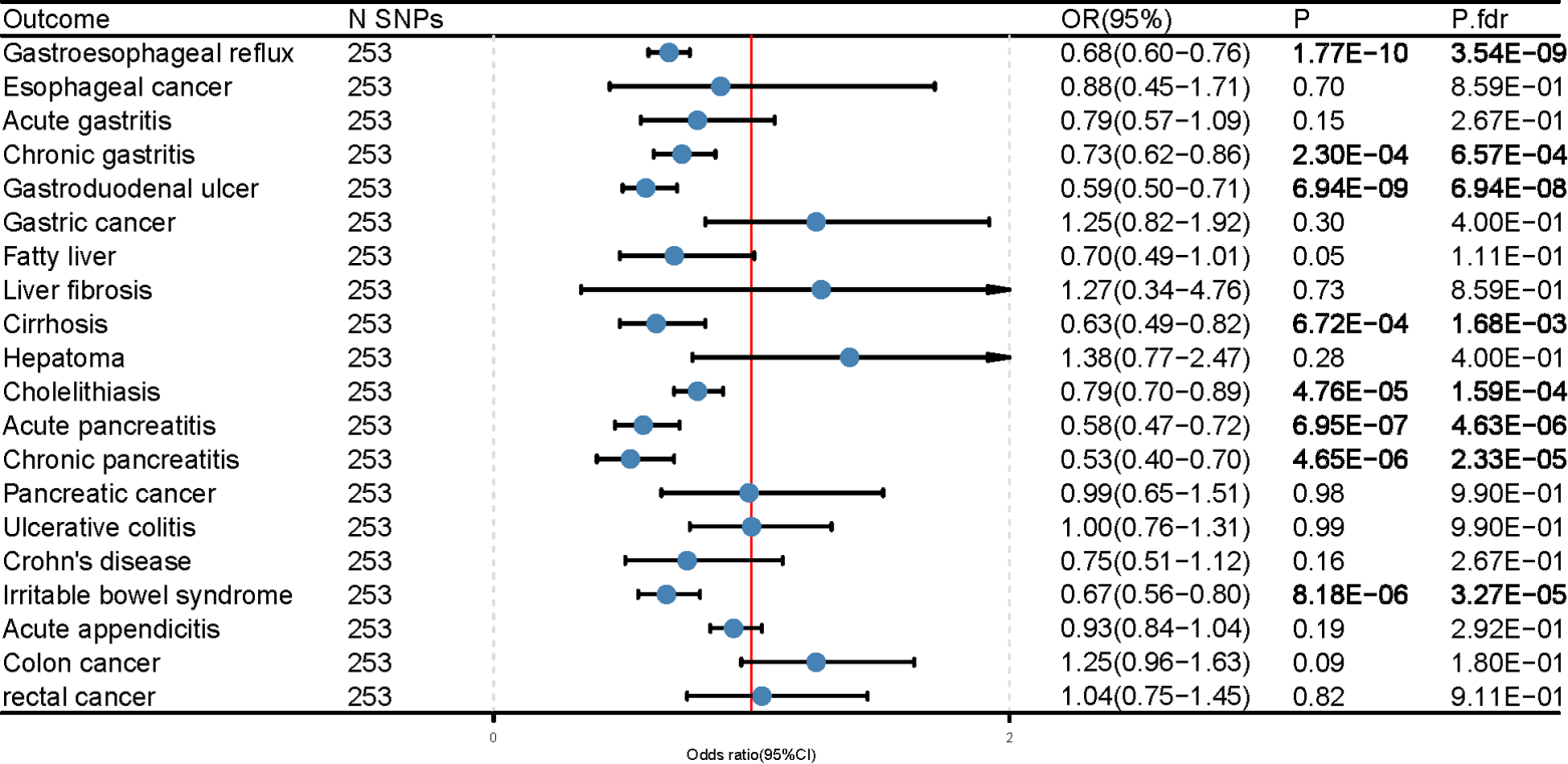
Univariate Mendelian randomization results for educational attainment and 20 gastrointestinal diseases.

**Figure 2.**
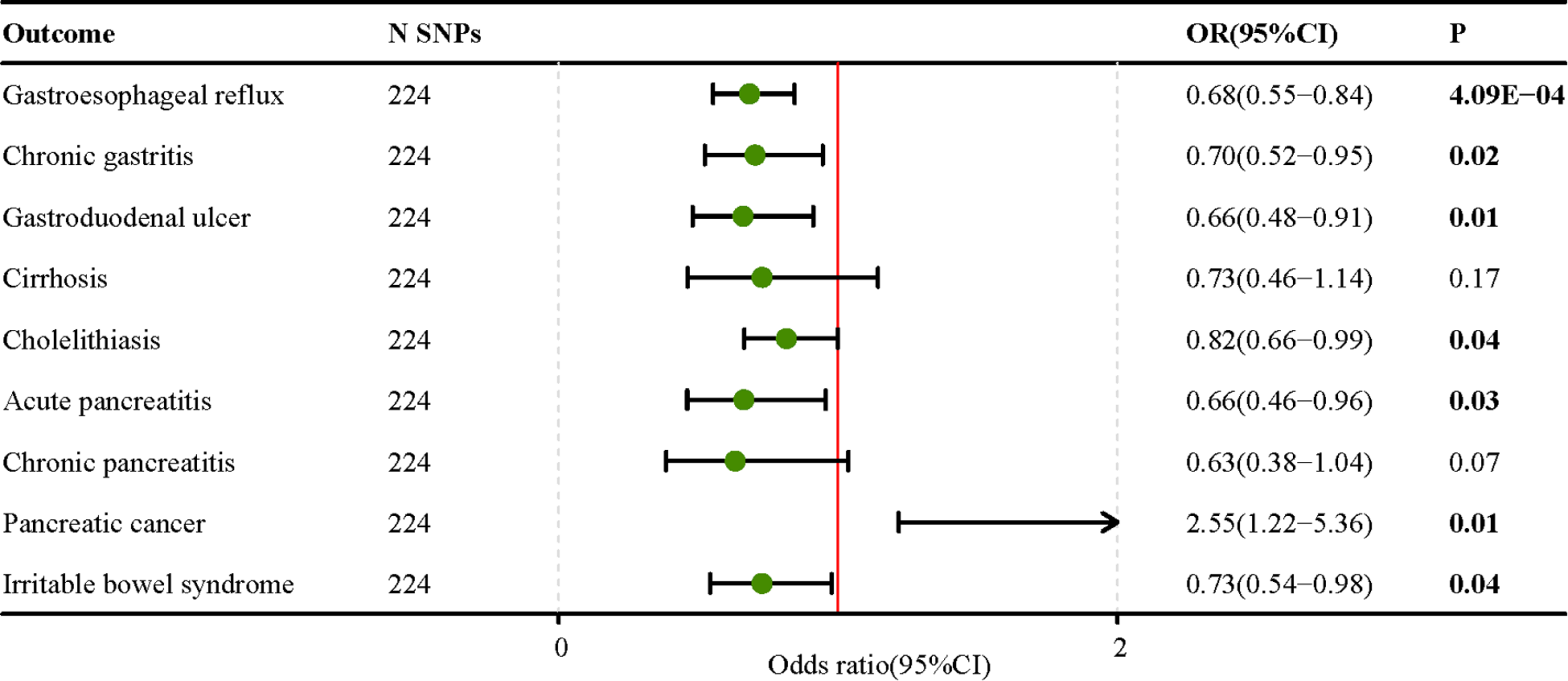
Educational attainment and 9 gastrointestinal diseases multivariate Mendelian randomization results after correcting for intelligence.

We found that EA was substantially associated with 8 gastrointestinal disorders in a two-sample MR study that included EA and 20 gastrointestinal diseases. More specifically, genetically predicted EA was significantly negatively correlated with gastroesophageal reflux (OR = 0.68; 95% CI, 0.60–0.76; P-fdr = 3.54E‐09), chronic gastritis (OR = 0.73; 95% CI, 0.62–0.86; P-fdr = 6.57E‐04), gastroduodenal ulcer (OR = 0.59; 95% CI, 0.50–0.71; P-fdr = 6.94e‐08), cirrhosis (OR = 0.63; 95% CI, 0.49–0.82; P-fdr = 1.68E‐03), cholelithiasis (OR = 0.79; 95% CI, 0.70–0.89; P-fdr = 1.59E‐04), acute pancreatitis (OR = 0.58; 95% CI 0.47–0.72; P-fdr = 4.63E‐06), chronic pancreatitis (OR = 0.53; 95% CI, 0.40–0.70; P-fdr = 2.33E‐05), and irritable bowel syndrome (OR = 0.67; 95% CI, 0.56–0.80; P -fdr = 3.27E‐05) (Fig. 1, Table S5, Supplemental Digital Content, http://links.lww.com/MD/O101). Sensitivity analysis did not reveal pleiotropy, while 1 to 2 outliers were detected in the analysis of gastroesophageal reflux disease, cholelithiasis, ulcerative colitis, and acute appendicitis using the MR-PRESSO method. However, the results remained unchanged even after removing these outliers, indicating the robustness of our findings (Table S6, Supplemental Digital Content, http://links.lww.com/MD/O101). Multivariate MR data corrected for IQ revealed that the genetically predicted relationships between EA and gastrointestinal illness were similar to the univariate results. These findings were corroborated by complementary approaches and sensitivity studies (Fig. 2). While there appears to be a potential link between EA and pancreatic malignancy in multivariate MR analysis (OR = 2.55; 95% CI, 1.22–5.36; *P* = .01), our supplementary MR Egger and MR Robust analyses did not provide meaningful results. Furthermore, the intercept value of the MR-Egger analysis suggested pleiotropy (Table S7, Supplemental Digital Content, http://links.lww.com/MD/O101). These findings suggest that unmeasured confounding factors may have influenced the observed association. Further research is required to confirm these results.

### 3.2. IQ and gastrointestinal disorders

**Figure 3.**
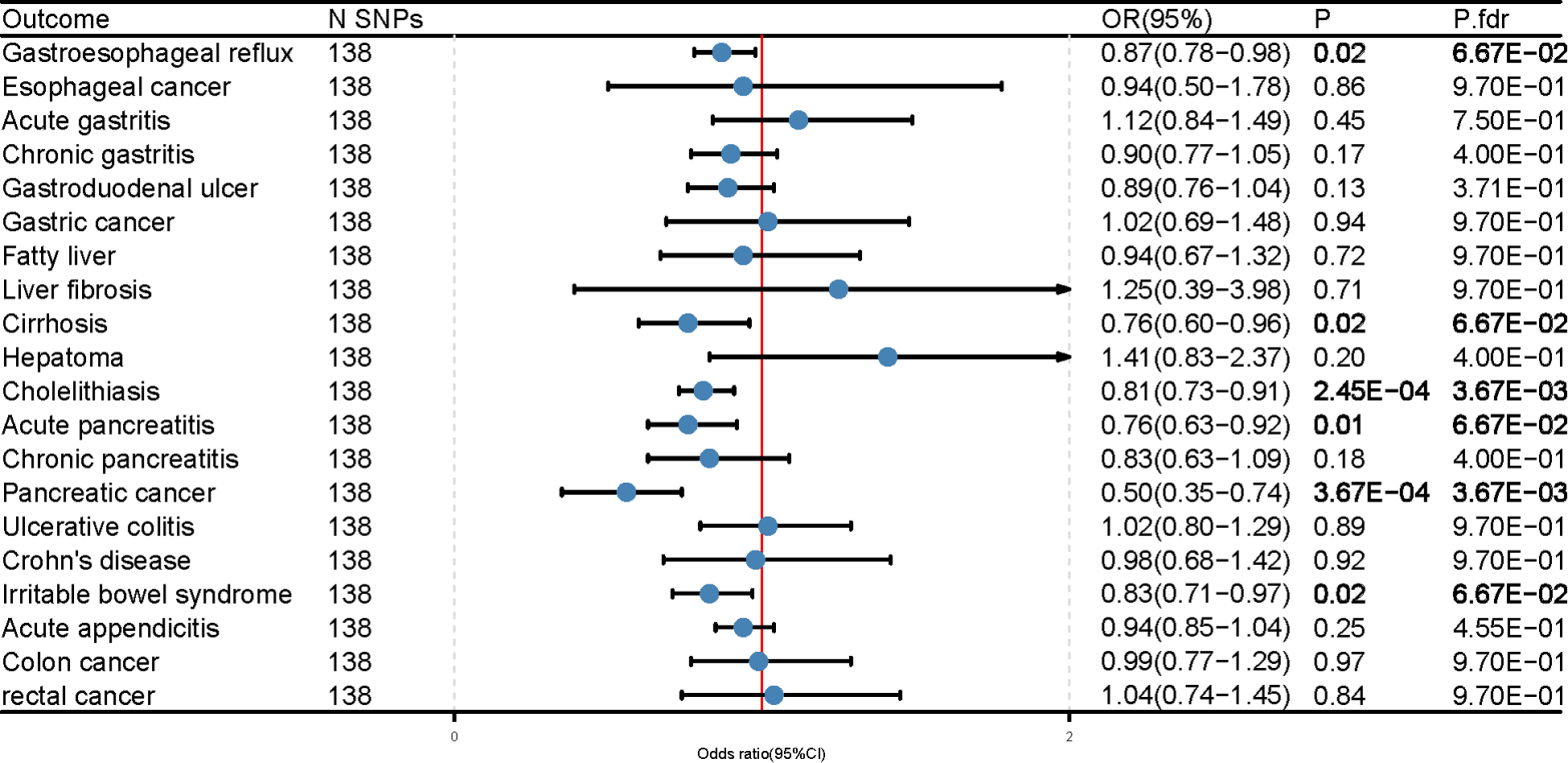
Univariate Mendelian randomization results for intelligence and 20 gastrointestinal diseases.

**Figure 4.**
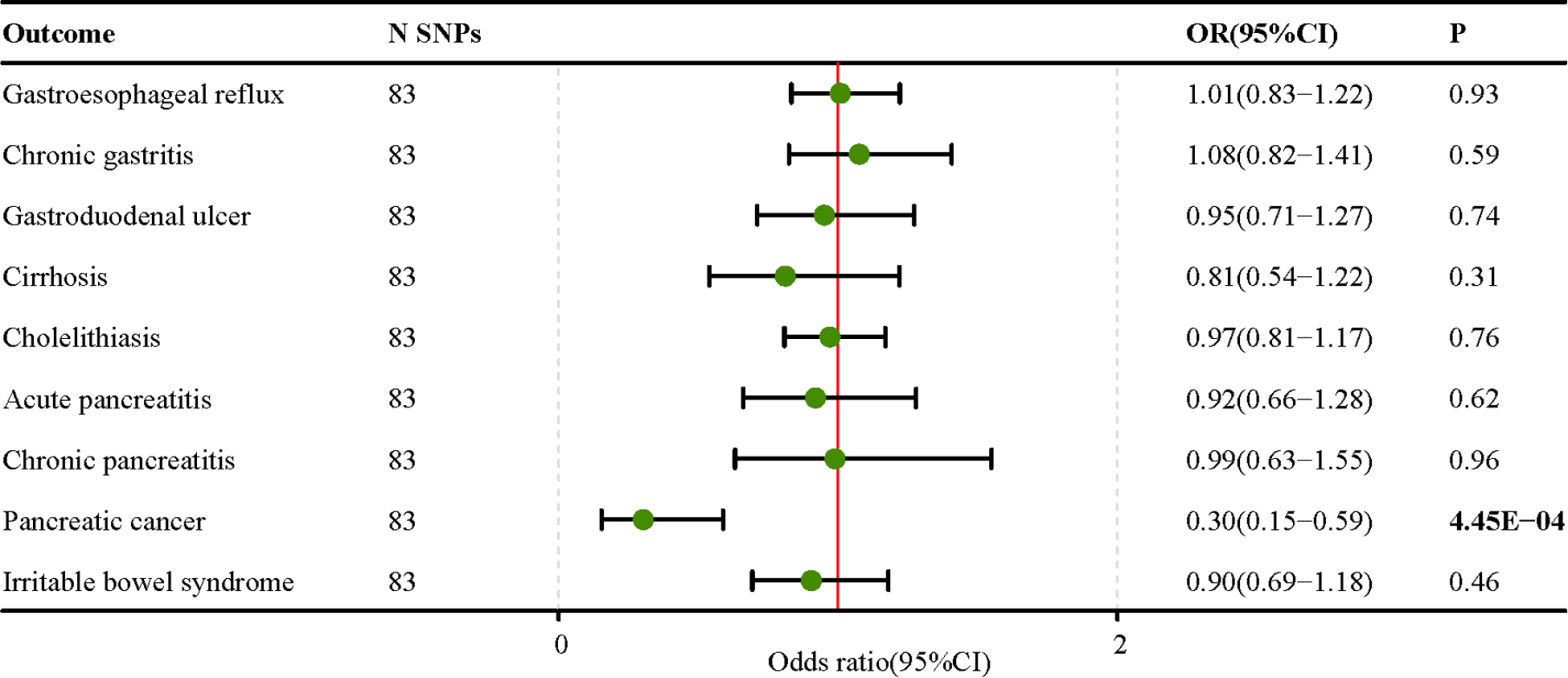
Intelligence and 9 gastrointestinal diseases multivariate Mendelian randomization results after correcting for educational attainment.

MR results showed that genetic predictive IQ was negatively correlated with cholelithiasis (OR = 0.81; 95% CI, 0.73–0.91; P-fdr = 3.67E‐03), pancreatic cancer (OR = 0.50; 95% CI, 0.35–0.74; P-fdr = 3.67E‐03) had a negative suggestive association (Fig. 3, Table S8, Supplemental Digital Content, http://links.lww.com/MD/O101), for gastroesophageal reflux disease (OR = 0.87. 95% CI, 0.78–0.98; P-fdr = 6.67E‐02), liver cirrhosis (OR = 0.76; 95% CI, 0.60–0.96; P-fdr = 0.07), acute pancreatitis (OR = 0.76; 95% CI, 0.63–0.92; P-fdr = 0.07), irritable bowel syndrome (OR = 0.83; 95% CI, 0.71–0.97; P-fdr = 0.07), after FDR correction, these *P*-values are no longer > .05, but still have suggestive significance. Similarly, the sensitivity analysis did not indicate the presence of horizontal pleiotropy, and the MR analysis remained unchanged after removing potential outliers in the IQ and gastrointestinal disease analyses using the MR-PRESSO method. This suggests that the results of our study are relatively consistent (Table S9, Supplemental Digital Content, http://links.lww.com/MD/O101). Multivariate MR analysis (Fig. 4) corrected for genetically predicted EA showed a protective effect of IQ against pancreatic cancer (OR = 0.30; 95% CI, 0.15–0.59; *P* = 4.45e‐04), consistent with the univariate MR results. In addition, multivariate MR results showed that the genetically predicted IQ was no longer associated with other gastrointestinal diseases (Table S10, Supplemental Digital Content, http://links.lww.com/MD/O101).

## 4. Discussion

We conducted a comprehensive MR study to investigate the relationship between IQ and EA in 20 patients with gastrointestinal illnesses. Our findings suggest no association between IQ and most of the gastrointestinal illnesses studied, and EA may have a protective effect against some of these illnesses. The results of the additional techniques, MR-Lasso and MR-Robust, were consistent with those of the IVW approach, suggesting that the MR results were generally reliable despite some minor heterogeneity and pleiotropy.

Previous studies have shown a negative correlation between EA and the risk of developing chronic diseases.^[40]^ Unhealthy behavior is closely related to low EA, which, is associated with a higher incidence of chronic diseases. According to various reports, individuals with low EA are more likely to engage in unhealthy behaviors, such as smoking, excessive alcohol consumption, leading sedentary lifestyles, and consuming diets high in saturated fat and sugar.^[41,42]^ These behaviors have been linked to an increased risk of developing various chronic diseases, including gastritis and other gastrointestinal disorders. A cohort study of Swedish students showed that higher education reduces the likelihood of unhealthy behaviors.^[41]^ According to a nationwide health assessment of the Italian population, the risk of conditions, including diabetes, hypertension, varicose veins, gastroduodenal ulcers, cholelithiasis, and cirrhosis, whose pathogenic processes are still unknown, declines with increasing EA exposure.^[43]^ Our findings support and extend these conclusions in line with previous studies that have used MR analysis to investigate the causal relationship between cognitive traits and gastrointestinal disorders. While these studies explored a limited number of gastrointestinal disorders,^[21]^ our research expands on these findings by providing evidence of the potential protective effect of EA on a broader range of gastrointestinal diseases.

Higher levels of education are associated with better lifestyles, which significantly lower the incidence rate of illnesses. Studies of young individuals in the US have revealed that those with lower EA engage in more negative activities, which lower their level of health.^[42]^ Brazilian epidemiological research on chronic illnesses conducted between 1998 and 2013 revealed that communities with low EA were more likely to experience chronic conditions, including diabetes and hypertension, which may be related to the unequal socioeconomic distribution of resources.^[44]^ MR analysis showed that smoking and sedentary activity were negatively correlated with EA levels,^[20]^ and Yuan et al employed MR techniques to confirm that these behaviors were risk factors for gastrointestinal disorders.^[45]^ This study demonstrated that various intermediary variables, including socioeconomic disparity, poor conduct, and harmful habits, moderate the causal association between EA and gastrointestinal disorders. Many plausible reasons exist for the apparent inverse correlation between EA and illness. Higher EA may be linked to a healthy lifestyle, which lowers the risk of developing illnesses. Higher EA may also be linked to reduced levels of chronic stress, which are established risk factors for many diseases (such as hypertension and cardiovascular disease).^[44,46]^

A recent meta-analysis of over 600,000 individuals highlighted a positive association between higher educational attainment and enhanced IQ, which persists throughout one’s lifetime.^[47]^ Deary et al conducted a genome-wide association study to evaluate the link between intelligence and health outcomes and discovered that intelligence was affected by various factors, including educational achievement.^[10]^ Our study demonstrated that a genetically predicted high IQ may decrease the risk of gastroesophageal reflux disease, cirrhosis, cholelithiasis, acute pancreatitis, pancreatic cancer, and irritable bowel syndrome. However, after considering the influence of educational achievement, the association between genetically predicted IQ scores and gastrointestinal illnesses became less pronounced. This finding suggests that the effect of IQ on gastrointestinal disorders may partially be mediated by educational attainment. Our findings are similar to previous studies, and we emphasize that our univariate and multivariate MR analyses revealed a possible link between IQ and pancreatic cancer, albeit with some pleiotropy. This can be partially attributed to individuals with higher intelligence often attaining higher levels of education and professional achievement, which can influence their lifestyle choices and overall health status.^[48]^ For instance, individuals with higher intelligence may afford a healthier lifestyle, including a balanced diet, easier access to adequate medical care during illness, and regular physical exercise. These lifestyle choices can help reduce the risk of certain diseases, including chronic conditions such as cancer. Furthermore, it is important to consider that factors such as sedentary behavior or obesity may increase the risk of cancer, particularly among individuals with cognitive impairment who may face challenges in adopting healthy behaviors.^[49–51]^ Our study adds to the limited literature on the relationship between IQ and gastrointestinal illnesses, emphasizing the need for further research to clarify the underlying mechanisms.

This study has several benefits. First, the MR analytical approach reduces the impact of confounding variables and reverse causation, and observational studies with results comparable to ours may provide a solid foundation for disease treatment and prevention. Second, since all the GWAS data in our analysis came from European populations, population stratification bias was significantly reduced. One limitation of this study was that our data originated solely from a European ancestry database, which may not represent other racial groups. Additionally, our data lacked replication analysis, potentially leading to partial and incomplete conclusions, reducing the reliability of our research findings. Furthermore, all of our research was based on publicly available databases, and these results were generated under assumed conditions without complete verification. Therefore, caution should be exercised when interpreting these results.

## 5. Conclusion

The results of our MR study indicated a possible link between gastrointestinal illness risk and IQ and EA levels. Further research is required to understand the pathogenic processes that drive this association. It is also important to note that focusing on people with low EA and IQ levels could be a successful strategy for lowering the prevalence of gastrointestinal disorders and public health expenses.

## Acknowledgments

We thank the researchers who contributed GWAS data. We would like to thank Editage (www.editage.com) for English language editing.

## Author contributions

**Conceptualization:** Jun He.

**Data curation:** Zhen Ding, Yunzhi Lin.

**Formal analysis:** Yunzhi Lin.

**Methodology:** Jun He.

**Software:** Jun He.

**Visualization:** Yunzhi Lin.

**Writing – original draft:** Jun He, Zhen Ding.

**Writing – review & editing:** Jun He, Zhen Ding.

## Supplementary Material


