# Supplementary material for: Education, intelligence, and 20 gastrointestinal disorders: A Mendelian randomization study
Source: Medicine (Baltimore). 2024 Dec 6;103(49):e40825. doi: 10.1097/MD.0000000000040825 (PMC11630976; doi:10.1097/MD.0000000000040825)
Supplement: Supplementary file 1 [file medi-103-e40825-s001.docx]

Supplementary table 1

| **Supplementary table 1. Education attainment instrument variable details and F statistics.** | | | | | | | | | |  |  |
| --- | --- | --- | --- | --- | --- | --- | --- | --- | --- | --- | --- |
| **SNP** | **effect_allele.exposure** | **other_allele.exposure** | **chr.exposure** | **pos.exposure** | **beta.exposure** | **se.exposure** | **eaf.exposure** | **pval.exposure** | **samplesize.exposure** | **R2** | **F** |
| rs10073890 | G | A | 5 | 1.2E+08 | -0.0126 | 0.00196 | 0.7364 | ####### | 766345 | ####### | 41.4578 |
| rs1008078 | T | C | 1 | 9.1E+07 | -0.0174 | 0.00173 | 0.4099 | ####### | 766345 | 0.00015 | 100.927 |
| rs10189857 | G | A | 2 | 6.1E+07 | -0.0173 | 0.00171 | 0.4184 | ####### | 766345 | 0.00014 | 101.762 |
| rs10191758 | G | A | 2 | 1.4E+08 | 0.01631 | 0.00175 | 0.381 | ####### | 766345 | 0.00013 | 86.8624 |
| rs10205801 | A | G | 2 | 2.3E+08 | -0.0105 | 0.00171 | 0.5068 | ####### | 766345 | ####### | 37.9197 |
| rs10215082 | G | A | 7 | 9.3E+07 | 0.01303 | 0.00172 | 0.5612 | ####### | 766345 | ####### | 57.3894 |
| rs10240905 | C | T | 7 | 3.2E+07 | 0.01167 | 0.00177 | 0.6684 | ####### | 766345 | ####### | 43.4706 |
| rs10456918 | C | A | 6 | 1.2E+08 | 0.01485 | 0.00224 | 0.182 | ####### | 766345 | ####### | 43.9498 |
| rs10460095 | A | G | 18 | 2.3E+07 | -0.0107 | 0.00171 | 0.5867 | ####### | 766345 | ####### | 38.8617 |
| rs1051474 | C | T | 9 | 1.1E+08 | 0.01301 | 0.00188 | 0.2738 | ####### | 766345 | ####### | 47.8893 |
| rs10760023 | G | C | 9 | 1.2E+08 | 0.01095 | 0.00183 | 0.3316 | ####### | 766345 | ####### | 35.8035 |
| rs10765775 | A | G | 11 | 9.6E+07 | 0.01488 | 0.00176 | 0.3963 | ####### | 766345 | 0.00011 | 71.4793 |
| rs10772644 | C | G | 12 | 1.3E+07 | 0.01614 | 0.00267 | 0.8929 | ####### | 766345 | ####### | 36.5413 |
| rs10773002 | T | A | 12 | 1.2E+08 | -0.0219 | 0.00197 | 0.7211 | ####### | 766345 | 0.00019 | 123.695 |
| rs10797055 | G | A | 1 | 1.6E+08 | 0.00986 | 0.00172 | 0.5102 | ####### | 766345 | ####### | 32.8622 |
| rs10798418 | T | C | 1 | 1.8E+08 | -0.0096 | 0.00173 | 0.466 | ####### | 766345 | ####### | 30.6007 |
| rs10856785 | T | C | 2 | 1.7E+07 | -0.0113 | 0.00192 | 0.7296 | ####### | 766345 | ####### | 34.7609 |
| rs10862376 | A | T | 12 | 8.2E+07 | 0.01616 | 0.00239 | 0.1361 | ####### | 766345 | ####### | 45.718 |
| rs10875121 | C | G | 1 | 9.8E+07 | 0.01834 | 0.00226 | 0.8571 | ####### | 766345 | ####### | 65.8539 |
| rs10887801 | T | G | 10 | 9E+07 | 0.01087 | 0.00171 | 0.4371 | ####### | 766345 | ####### | 40.408 |
| rs10940921 | G | T | 5 | 3.1E+07 | -0.0109 | 0.00177 | 0.5697 | ####### | 766345 | ####### | 37.8538 |
| rs10963297 | G | C | 9 | 1791832 | 0.01904 | 0.00198 | 0.2517 | ####### | 766345 | 0.00014 | 92.4706 |
| rs10994777 | A | G | 10 | 6.3E+07 | 0.0146 | 0.00232 | 0.1395 | ####### | 766345 | ####### | 39.6032 |
| rs11023749 | A | G | 11 | 1.6E+07 | 0.01132 | 0.0018 | 0.6701 | ####### | 766345 | ####### | 39.5501 |
| rs1105307 | A | G | 9 | 7.2E+07 | -0.0117 | 0.00195 | 0.2449 | ####### | 766345 | ####### | 36.1849 |
| rs1106090 | A | G | 2 | 5.8E+07 | 0.01173 | 0.00175 | 0.6259 | ####### | 766345 | ####### | 44.9283 |
| rs11081529 | C | T | 18 | 7.6E+07 | -0.0131 | 0.00186 | 0.2568 | ####### | 766345 | ####### | 49.6798 |
| rs11123818 | A | G | 2 | 1E+08 | 0.02081 | 0.00175 | 0.3946 | ####### | 766345 | 0.00021 | 141.406 |
| rs111821073 | T | C | 9 | 9.9E+07 | 0.01385 | 0.00237 | 0.1633 | ####### | 766345 | ####### | 34.151 |
| rs112687095 | A | G | 4 | 1777045 | 0.01325 | 0.00238 | 0.165 | ####### | 766345 | ####### | 30.994 |
| rs112806496 | G | C | 2 | 8E+07 | 0.0187 | 0.00305 | 0.08503 | ####### | 766345 | ####### | 37.591 |
| rs113182709 | A | G | 8 | 1.4E+08 | 0.03225 | 0.00567 | 0.02381 | ####### | 766345 | ####### | 32.3514 |
| rs113520408 | A | G | 7 | 1.3E+08 | 0.01304 | 0.00192 | 0.2857 | ####### | 766345 | ####### | 46.1267 |
| rs113615161 | T | C | 7 | 1.1E+08 | -0.0147 | 0.0025 | 0.1395 | ####### | 766345 | ####### | 34.6685 |
| rs1143770 | T | C | 11 | 1.2E+08 | 0.01136 | 0.00172 | 0.5918 | ####### | 766345 | ####### | 43.6214 |
| rs115000530 | T | A | 3 | 7E+07 | 0.02892 | 0.00381 | 0.06122 | ####### | 766345 | ####### | 57.6165 |
| rs115454970 | T | G | 4 | 6.7E+07 | -0.0119 | 0.00199 | 0.3044 | ####### | 766345 | ####### | 35.4593 |
| rs11601122 | G | A | 11 | 7.2E+07 | -0.0195 | 0.0023 | 0.1497 | ####### | 766345 | ####### | 71.6599 |
| rs11620355 | A | G | 13 | 9.2E+07 | 0.01756 | 0.003 | 0.1156 | ####### | 766345 | ####### | 34.2615 |
| rs11627087 | G | A | 14 | 5.7E+07 | -0.0179 | 0.00325 | 0.08503 | ####### | 766345 | ####### | 30.2669 |
| rs11635092 | A | G | 15 | 2.7E+07 | -0.0123 | 0.00177 | 0.3639 | ####### | 766345 | ####### | 48.3693 |
| rs11657342 | A | G | 17 | 7.9E+07 | 0.01404 | 0.00191 | 0.3554 | ####### | 766345 | ####### | 54.034 |
| rs11663602 | A | C | 18 | 7.8E+07 | -0.0121 | 0.0019 | 0.2568 | ####### | 766345 | ####### | 40.7581 |
| rs11678980 | A | G | 2 | 1.6E+08 | -0.0174 | 0.00172 | 0.4456 | ####### | 766345 | 0.00015 | 102.81 |
| rs11681861 | G | T | 2 | 3E+07 | -0.0144 | 0.00259 | 0.1565 | ####### | 766345 | ####### | 30.6976 |
| rs11694904 | T | C | 2 | 1.8E+08 | 0.01215 | 0.00185 | 0.3384 | ####### | 766345 | ####### | 43.1329 |
| rs11732657 | A | G | 4 | 1.7E+08 | -0.0127 | 0.00197 | 0.7007 | ####### | 766345 | ####### | 41.8222 |
| rs117468730 | A | G | 16 | 1E+07 | -0.0352 | 0.00597 | 0.0119 | ####### | 766345 | ####### | 34.7843 |
| rs11752914 | C | T | 6 | 1.6E+08 | -0.0121 | 0.00216 | 0.1973 | ####### | 766345 | ####### | 31.2771 |
| rs11772580 | T | G | 7 | 1E+08 | -0.012 | 0.00201 | 0.2534 | ####### | 766345 | ####### | 35.5833 |
| rs117799466 | C | G | 15 | 3.5E+07 | 0.01173 | 0.00198 | 0.3776 | ####### | 766345 | ####### | 35.0966 |
| rs11871429 | G | A | 17 | 4.3E+07 | -0.0143 | 0.00202 | 0.2041 | ####### | 766345 | ####### | 49.7653 |
| rs12028010 | C | T | 1 | 4.2E+07 | -0.017 | 0.00202 | 0.2228 | ####### | 766345 | ####### | 70.4935 |
| rs12134151 | C | G | 1 | 9.6E+07 | -0.0125 | 0.0017 | 0.5221 | ####### | 766345 | ####### | 53.6341 |
| rs12332731 | A | T | 5 | 9.3E+07 | 0.01374 | 0.00218 | 0.2024 | ####### | 766345 | ####### | 39.7247 |
| rs12375949 | C | T | 9 | 1.2E+08 | 0.01447 | 0.00172 | 0.5697 | ####### | 766345 | 0.0001 | 70.775 |
| rs12468040 | G | T | 2 | 4.5E+07 | -0.0143 | 0.00175 | 0.6037 | ####### | 766345 | ####### | 66.9592 |
| rs12503522 | T | C | 4 | 9.5E+07 | -0.0113 | 0.00188 | 0.2483 | ####### | 766345 | ####### | 35.8088 |
| rs12519073 | T | C | 5 | 1.4E+08 | -0.0122 | 0.00202 | 0.2381 | ####### | 766345 | ####### | 36.5366 |
| rs12574281 | C | A | 11 | 1.3E+08 | 0.01077 | 0.00176 | 0.3997 | ####### | 766345 | ####### | 37.4461 |
| rs12602286 | T | G | 17 | 1.9E+07 | 0.01701 | 0.00255 | 0.8861 | ####### | 766345 | ####### | 44.4967 |
| rs12643771 | T | C | 4 | 1.4E+08 | 0.01518 | 0.00184 | 0.3112 | ####### | 766345 | ####### | 68.0625 |
| rs12682775 | C | T | 9 | 1.4E+08 | 0.01187 | 0.00204 | 0.2143 | ####### | 766345 | ####### | 33.8564 |
| rs12804787 | G | A | 11 | 1.3E+08 | -0.0181 | 0.00327 | 0.06803 | ####### | 766345 | ####### | 30.7737 |
| rs1291818 | C | T | 10 | 1.1E+07 | -0.0109 | 0.0017 | 0.5153 | ####### | 766345 | ####### | 40.7344 |
| rs12940014 | C | T | 17 | 1.8E+07 | 0.00936 | 0.0017 | 0.5204 | ####### | 766345 | ####### | 30.3147 |
| rs12955211 | A | T | 18 | 2.6E+07 | 0.01097 | 0.00182 | 0.3554 | ####### | 766345 | ####### | 36.3304 |
| rs13010566 | C | A | 2 | 1.3E+08 | 0.0106 | 0.0017 | 0.5612 | ####### | 766345 | ####### | 38.8789 |
| rs13029509 | A | G | 2 | 2.2E+08 | -0.0105 | 0.0017 | 0.4677 | ####### | 766345 | ####### | 38.0762 |
| rs13090388 | T | C | 3 | 4.9E+07 | 0.02852 | 0.00184 | 0.3095 | ####### | 766345 | 0.00035 | 240.25 |
| rs13130765 | C | G | 4 | 4.5E+07 | -0.0101 | 0.00173 | 0.4558 | ####### | 766345 | ####### | 34.3545 |
| rs13141210 | T | C | 4 | 6.8E+07 | 0.01361 | 0.00172 | 0.5085 | ####### | 766345 | ####### | 62.6123 |
| rs13145650 | T | C | 4 | 1.2E+08 | -0.0192 | 0.00306 | 0.90816 | ####### | 766345 | ####### | 39.2875 |
| rs1334297 | A | G | 13 | 5.8E+07 | 0.02449 | 0.00192 | 0.784 | ####### | 766345 | 0.0002 | 162.695 |
| rs13422673 | T | C | 2 | 1.6E+08 | -0.012 | 0.0017 | 0.4847 | ####### | 766345 | ####### | 49.9101 |
| rs1363862 | A | G | 5 | 6E+07 | -0.0117 | 0.00192 | 0.2602 | ####### | 766345 | ####### | 37.1973 |
| rs1381247 | C | T | 17 | 2302387 | -0.0101 | 0.00182 | 0.2908 | ####### | 766345 | ####### | 30.9796 |
| rs1391438 | C | T | 4 | 1.1E+08 | -0.0167 | 0.00183 | 0.6854 | ####### | 766345 | 0.00012 | 83.2781 |
| rs1427298 | T | C | 2 | 1.5E+08 | 0.0102 | 0.00172 | 0.4116 | ####### | 766345 | ####### | 35.1677 |
| rs1450782 | G | T | 4 | 1.1E+08 | -0.0095 | 0.00173 | 0.602 | ####### | 766345 | ####### | 29.8381 |
| rs1455350 | A | T | 2 | 2E+08 | -0.0161 | 0.0017 | 0.4711 | ####### | 766345 | 0.00013 | 90.1383 |
| rs152603 | G | A | 5 | 1.1E+08 | 0.01019 | 0.00177 | 0.3861 | ####### | 766345 | ####### | 33.1438 |
| rs1558727 | T | C | 12 | 9.8E+07 | -0.0107 | 0.0017 | 0.4847 | ####### | 766345 | ####### | 39.5419 |
| rs1566085 | T | G | 8 | 1.4E+08 | 0.01645 | 0.00171 | 0.5697 | ####### | 766345 | 0.00013 | 92.5421 |
| rs1569092 | A | G | 1 | 7.5E+07 | 0.01807 | 0.00234 | 0.182 | ####### | 766345 | ####### | 59.6327 |
| rs1584469 | T | C | 5 | 1.2E+08 | -0.013 | 0.00185 | 0.3112 | ####### | 766345 | ####### | 49.6073 |
| rs1592757 | C | G | 5 | 1E+08 | -0.0105 | 0.0018 | 0.3759 | ####### | 766345 | ####### | 33.7045 |
| rs1595973 | T | C | 4 | 6.6E+07 | -0.01 | 0.00173 | 0.5595 | ####### | 766345 | ####### | 33.5462 |
| rs1618725 | T | C | 18 | 2.1E+07 | 0.01477 | 0.00174 | 0.5204 | ####### | 766345 | 0.00011 | 72.0547 |
| rs1620977 | G | A | 1 | 7.3E+07 | -0.0205 | 0.00195 | 0.6905 | ####### | 766345 | 0.00018 | 110.089 |
| rs1671770 | C | A | 12 | 1.2E+08 | -0.0134 | 0.00223 | 0.8061 | ####### | 766345 | ####### | 36.2156 |
| rs16846463 | G | A | 2 | 1.4E+08 | -0.0226 | 0.00283 | 0.1173 | ####### | 766345 | 0.00011 | 63.5485 |
| rs16854920 | C | T | 1 | 2E+08 | 0.01007 | 0.00181 | 0.3554 | ####### | 766345 | ####### | 30.9529 |
| rs1689510 | C | G | 12 | 5.6E+07 | 0.01761 | 0.0018 | 0.3435 | ####### | 766345 | 0.00014 | 95.7136 |
| rs16995054 | T | C | 20 | 1.5E+07 | -0.0139 | 0.00208 | 0.2007 | ####### | 766345 | ####### | 44.6584 |
| rs17048855 | A | G | 3 | 8258174 | 0.01184 | 0.00179 | 0.3248 | ####### | 766345 | ####### | 43.7519 |
| rs17110109 | C | T | 12 | 5.5E+07 | 0.01023 | 0.00175 | 0.3776 | ####### | 766345 | ####### | 34.1724 |
| rs17126938 | C | T | 10 | 1.1E+08 | 0.01536 | 0.0025 | 0.1207 | ####### | 766345 | ####### | 37.7487 |
| rs17425572 | G | A | 9 | 8.8E+07 | -0.0122 | 0.0017 | 0.5425 | ####### | 766345 | ####### | 51.84 |
| rs17489649 | G | A | 5 | 1.1E+08 | -0.0139 | 0.00181 | 0.3265 | ####### | 766345 | ####### | 58.9756 |
| rs175325 | A | T | 20 | 2.2E+07 | -0.0118 | 0.00174 | 0.5816 | ####### | 766345 | ####### | 45.9123 |
| rs17551064 | G | A | 7 | 5E+07 | -0.0149 | 0.0023 | 0.1599 | ####### | 766345 | ####### | 42.137 |
| rs17563464 | A | C | 5 | 2.7E+07 | -0.0148 | 0.00212 | 0.2041 | ####### | 766345 | ####### | 48.5388 |
| rs17565975 | A | G | 11 | 1.1E+08 | -0.0114 | 0.00171 | 0.5306 | ####### | 766345 | ####### | 44.6005 |
| rs17598675 | C | T | 4 | 1.8E+08 | 0.01199 | 0.0017 | 0.5187 | ####### | 766345 | ####### | 49.744 |
| rs176218 | T | G | 14 | 3E+07 | 0.01883 | 0.00215 | 0.2007 | ####### | 766345 | 0.00011 | 76.705 |
| rs1827540 | G | A | 5 | 6.3E+07 | -0.0106 | 0.0017 | 0.466 | ####### | 766345 | ####### | 38.8789 |
| rs1866823 | A | G | 8 | 5.7E+07 | 0.01009 | 0.00171 | 0.551 | ####### | 766345 | ####### | 34.8169 |
| rs1882273 | C | G | 2 | 1.9E+08 | -0.0123 | 0.00181 | 0.3469 | ####### | 766345 | ####### | 46.255 |
| rs192436652 | T | C | 19 | 5.5E+07 | -0.035 | 0.00545 | 0.02211 | ####### | 766345 | ####### | 41.1716 |
| rs1925576 | G | A | 10 | 6.9E+07 | 0.00997 | 0.00171 | 0.4422 | ####### | 766345 | ####### | 33.9937 |
| rs1947114 | G | A | 2 | 1.7E+08 | 0.01071 | 0.00192 | 0.2551 | ####### | 766345 | ####### | 31.1155 |
| rs1949226 | A | T | 4 | 2.2E+07 | -0.0097 | 0.00174 | 0.5901 | ####### | 766345 | ####### | 30.7579 |
| rs1964927 | G | A | 21 | 4.3E+07 | -0.0142 | 0.00177 | 0.6378 | ####### | 766345 | ####### | 64.6343 |
| rs2052285 | A | G | 16 | 5.1E+07 | 0.01123 | 0.00175 | 0.5765 | ####### | 766345 | ####### | 41.1797 |
| rs2067854 | A | G | 14 | 7.2E+07 | 0.01477 | 0.00209 | 0.182 | ####### | 766345 | ####### | 49.9423 |
| rs2179152 | C | T | 6 | 2.6E+07 | 0.01455 | 0.00176 | 0.6429 | ####### | 766345 | ####### | 68.344 |
| rs2182505 | C | T | 6 | 5.2E+07 | -0.0109 | 0.00192 | 0.7364 | ####### | 766345 | ####### | 31.9932 |
| rs225291 | G | A | 17 | 3.4E+07 | -0.0121 | 0.00214 | 0.8027 | ####### | 766345 | ####### | 31.7064 |
| rs2256965 | G | A | 6 | 3.2E+07 | -0.0113 | 0.00176 | 0.5425 | ####### | 766345 | ####### | 41.0764 |
| rs2283076 | G | A | 7 | 1.3E+08 | -0.0114 | 0.00204 | 0.2143 | ####### | 766345 | ####### | 31.3929 |
| rs2287838 | A | G | 19 | 9959014 | -0.0115 | 0.00171 | 0.534 | ####### | 766345 | ####### | 45.385 |
| rs2302761 | T | C | 17 | 7358520 | 0.01354 | 0.00209 | 0.1905 | ####### | 766345 | ####### | 41.9706 |
| rs2347526 | C | T | 4 | 1.6E+08 | 0.01395 | 0.00179 | 0.6378 | ####### | 766345 | ####### | 60.7355 |
| rs2414072 | A | T | 15 | 5.1E+07 | -0.0101 | 0.00171 | 0.4864 | ####### | 766345 | ####### | 34.5414 |
| rs242093 | A | G | 14 | 6.9E+07 | -0.0103 | 0.00172 | 0.5476 | ####### | 766345 | ####### | 35.9303 |
| rs2441111 | A | G | 5 | 5.8E+07 | 0.01087 | 0.0017 | 0.5323 | ####### | 766345 | ####### | 40.8847 |
| rs2447535 | G | A | 8 | 1.2E+08 | 0.01181 | 0.00185 | 0.7245 | ####### | 766345 | ####### | 40.7527 |
| rs2478208 | C | G | 13 | 8.1E+07 | -0.0106 | 0.0017 | 0.5 | ####### | 766345 | ####### | 38.8789 |
| rs2545798 | A | T | 5 | 1.8E+08 | -0.0135 | 0.00171 | 0.4949 | ####### | 766345 | ####### | 61.9581 |
| rs2554835 | A | G | 18 | 7.4E+07 | 0.00974 | 0.00175 | 0.398 | ####### | 766345 | ####### | 30.9772 |
| rs2570497 | T | C | 2 | 1E+08 | -0.0123 | 0.00177 | 0.6735 | ####### | 766345 | ####### | 48.5266 |
| rs2725370 | C | T | 8 | 3.1E+07 | 0.01536 | 0.00187 | 0.7109 | ####### | 766345 | ####### | 67.4682 |
| rs277828 | A | C | 13 | 1.1E+08 | -0.0109 | 0.00196 | 0.2568 | ####### | 766345 | ####### | 30.984 |
| rs2787101 | T | C | 14 | 3E+07 | 0.00968 | 0.00174 | 0.6173 | ####### | 766345 | ####### | 30.9494 |
| rs2819336 | C | T | 1 | 4.4E+07 | -0.0183 | 0.00177 | 0.6616 | ####### | 766345 | 0.00015 | 106.661 |
| rs2820314 | C | A | 1 | 2E+08 | -0.011 | 0.0018 | 0.3163 | ####### | 766345 | ####### | 37.3457 |
| rs28373063 | C | G | 4 | 1.8E+08 | 0.01389 | 0.00229 | 0.2075 | ####### | 766345 | ####### | 36.7903 |
| rs28513670 | G | A | 15 | 6.6E+07 | 0.01477 | 0.00225 | 0.1531 | ####### | 766345 | ####### | 43.0919 |
| rs2885198 | G | A | 3 | 1.4E+08 | -0.0103 | 0.0017 | 0.5017 | ####### | 766345 | ####### | 36.3538 |
| rs2901616 | A | G | 1 | 7.2E+07 | 0.00941 | 0.00171 | 0.517 | ####### | 766345 | ####### | 30.2822 |
| rs2905426 | T | G | 19 | 1.9E+07 | 0.01037 | 0.00181 | 0.6446 | ####### | 766345 | ####### | 32.8247 |
| rs2923431 | C | G | 8 | 4.2E+07 | 0.0114 | 0.00176 | 0.6446 | ####### | 766345 | ####### | 41.9551 |
| rs2971970 | G | T | 7 | 1.3E+08 | 0.01654 | 0.00207 | 0.7721 | ####### | 766345 | ####### | 63.8455 |
| rs2998315 | G | A | 14 | 8.5E+07 | 0.01269 | 0.00171 | 0.5782 | ####### | 766345 | ####### | 55.072 |
| rs3013014 | A | G | 10 | 1.2E+07 | -0.0102 | 0.00172 | 0.6105 | ####### | 766345 | ####### | 35.444 |
| rs301800 | C | T | 1 | 8490603 | -0.0152 | 0.00224 | 0.8197 | ####### | 766345 | ####### | 45.8039 |
| rs3026996 | C | A | 1 | 1.6E+08 | -0.0154 | 0.00199 | 0.284 | ####### | 766345 | ####### | 59.6543 |
| rs31940 | A | G | 5 | 1.1E+07 | 0.01548 | 0.00246 | 0.1344 | ####### | 766345 | ####### | 39.5979 |
| rs320693 | C | G | 7 | 1.4E+08 | 0.01204 | 0.0017 | 0.4728 | ####### | 766345 | ####### | 50.1597 |
| rs337637 | A | G | 4 | 3.9E+07 | 0.01123 | 0.00177 | 0.3367 | ####### | 766345 | ####### | 40.2544 |
| rs34316 | C | A | 5 | 8.8E+07 | -0.0202 | 0.00177 | 0.5799 | ####### | 766345 | 0.0002 | 129.728 |
| rs34394051 | G | A | 1 | 6853091 | 0.01392 | 0.0024 | 0.1599 | ####### | 766345 | ####### | 33.64 |
| rs34485537 | T | C | 16 | 8.4E+07 | 0.01075 | 0.00173 | 0.3895 | ####### | 766345 | ####### | 38.6122 |
| rs34853711 | C | G | 7 | 2214187 | -0.016 | 0.00206 | 0.2466 | ####### | 766345 | ####### | 60.0249 |
| rs35039375 | G | A | 1 | 1.7E+08 | -0.0198 | 0.00293 | 0.09354 | ####### | 766345 | ####### | 45.8047 |
| rs35309068 | G | T | 12 | 1.5E+07 | 0.01321 | 0.00171 | 0.466 | ####### | 766345 | ####### | 59.6779 |
| rs35316276 | T | C | 16 | 6.8E+07 | 0.01173 | 0.00194 | 0.2942 | ####### | 766345 | ####### | 36.5589 |
| rs35417702 | T | C | 7 | 7.2E+07 | -0.0145 | 0.0017 | 0.5765 | ####### | 766345 | 0.0001 | 72.25 |
| rs35475880 | T | G | 3 | 1.7E+08 | -0.0151 | 0.00208 | 0.1837 | ####### | 766345 | ####### | 52.7718 |
| rs35532491 | T | A | 22 | 3.4E+07 | 0.02007 | 0.00286 | 0.1071 | ####### | 766345 | ####### | 49.2451 |
| rs36083520 | C | T | 4 | 2.4E+07 | 0.01629 | 0.00223 | 0.1667 | ####### | 766345 | ####### | 53.362 |
| rs36119825 | A | G | 7 | 4.9E+07 | 0.01063 | 0.00171 | 0.4694 | ####### | 766345 | ####### | 38.6433 |
| rs363096 | C | T | 4 | 3180021 | 0.01363 | 0.00172 | 0.5748 | ####### | 766345 | ####### | 62.7964 |
| rs3747631 | C | G | 1 | 2E+08 | 0.02207 | 0.00208 | 0.2279 | ####### | 766345 | 0.00017 | 112.584 |
| rs3788556 | C | T | 22 | 4E+07 | -0.0114 | 0.00171 | 0.5408 | ####### | 766345 | ####### | 44.2886 |
| rs3800546 | G | C | 6 | 1.7E+08 | -0.0118 | 0.00194 | 0.2704 | ####### | 766345 | ####### | 37.1848 |
| rs3809634 | G | A | 16 | 5.4E+07 | 0.01058 | 0.00185 | 0.335 | ####### | 766345 | ####### | 32.706 |
| rs3890802 | A | G | 5 | 1.5E+08 | -0.0113 | 0.00191 | 0.2687 | ####### | 766345 | ####### | 35.1879 |
| rs3897821 | G | A | 1 | 2.4E+08 | -0.015 | 0.0018 | 0.3503 | ####### | 766345 | 0.0001 | 69.6298 |
| rs401687 | C | G | 12 | 8.4E+07 | 0.01144 | 0.0017 | 0.4558 | ####### | 766345 | ####### | 45.285 |
| rs406413 | T | A | 5 | 1.1E+08 | -0.017 | 0.00209 | 0.2109 | ####### | 766345 | ####### | 65.7729 |
| rs4073894 | A | G | 7 | 1E+08 | 0.01524 | 0.00211 | 0.1769 | ####### | 766345 | ####### | 52.1681 |
| rs4328757 | T | C | 3 | 3.7E+07 | 0.01067 | 0.00174 | 0.6514 | ####### | 766345 | ####### | 37.6037 |
| rs4352658 | T | C | 6 | 8.8E+07 | -0.0212 | 0.00308 | 0.09014 | ####### | 766345 | ####### | 47.3773 |
| rs4369924 | A | G | 20 | 4.2E+07 | 0.01362 | 0.00234 | 0.1684 | ####### | 766345 | ####### | 33.8784 |
| rs4382592 | G | T | 9 | 1.3E+08 | 0.01636 | 0.00185 | 0.699 | ####### | 766345 | 0.00011 | 78.203 |
| rs4384309 | A | G | 10 | 1.3E+08 | 0.0109 | 0.00172 | 0.4796 | ####### | 766345 | ####### | 40.1602 |
| rs4392737 | G | A | 6 | 1.5E+08 | -0.0097 | 0.00173 | 0.3827 | ####### | 766345 | ####### | 31.4377 |
| rs4442732 | G | A | 14 | 6.1E+07 | -0.0106 | 0.00176 | 0.5969 | ####### | 766345 | ####### | 36.4789 |
| rs4497562 | G | A | 13 | 6.3E+07 | -0.012 | 0.00192 | 0.2823 | ####### | 766345 | ####### | 39.3234 |
| rs4667025 | A | G | 2 | 1.9E+08 | 0.00957 | 0.00174 | 0.3878 | ####### | 766345 | ####### | 30.25 |
| rs4700393 | G | A | 5 | 6E+07 | 0.02086 | 0.0017 | 0.5289 | ####### | 766345 | 0.00022 | 150.567 |
| rs4726070 | A | G | 7 | 1.5E+08 | 0.01251 | 0.00174 | 0.6207 | ####### | 766345 | ####### | 51.6911 |
| rs4733264 | C | G | 8 | 3.1E+07 | -0.0095 | 0.00174 | 0.631 | ####### | 766345 | ####### | 30.0606 |
| rs4743923 | C | T | 9 | 9.6E+07 | 0.00985 | 0.00176 | 0.3673 | ####### | 766345 | ####### | 31.3218 |
| rs4757957 | C | G | 11 | 1.3E+07 | 0.0141 | 0.00184 | 0.6514 | ####### | 766345 | ####### | 58.7222 |
| rs4766424 | G | C | 12 | 1954096 | -0.0141 | 0.00258 | 0.91156 | ####### | 766345 | ####### | 29.8675 |
| rs4778058 | C | T | 15 | 9.3E+07 | 0.01017 | 0.0017 | 0.5221 | ####### | 766345 | ####### | 35.7885 |
| rs4787457 | G | A | 16 | 2.9E+07 | -0.0174 | 0.00176 | 0.3146 | ####### | 766345 | 0.00013 | 97.8526 |
| rs4810227 | A | G | 20 | 6E+07 | 0.01272 | 0.00175 | 0.6344 | ####### | 766345 | ####### | 52.8321 |
| rs4839155 | G | T | 1 | 1.1E+08 | -0.0125 | 0.002 | 0.25 | ####### | 766345 | ####### | 39.125 |
| rs4846724 | A | G | 1 | 2.2E+08 | 0.01018 | 0.0017 | 0.4915 | ####### | 766345 | ####### | 35.859 |
| rs4870482 | G | C | 6 | 1.6E+08 | -0.0108 | 0.0019 | 0.2585 | ####### | 766345 | ####### | 32.49 |
| rs4888746 | G | A | 16 | 7.8E+07 | -0.0095 | 0.00174 | 0.3776 | ####### | 766345 | ####### | 29.9347 |
| rs4904523 | A | G | 14 | 9E+07 | -0.0094 | 0.0017 | 0.4592 | ####### | 766345 | ####### | 30.3147 |
| rs4945424 | A | C | 11 | 8E+07 | -0.0099 | 0.00171 | 0.415 | ####### | 766345 | ####### | 33.6536 |
| rs4964046 | G | A | 12 | 2.7E+07 | 0.01053 | 0.00178 | 0.335 | ####### | 766345 | ####### | 34.9959 |
| rs4972400 | A | G | 2 | 1.7E+08 | 0.01156 | 0.00181 | 0.352 | ####### | 766345 | ####### | 40.7905 |
| rs4984541 | G | A | 15 | 9.7E+07 | 0.01233 | 0.00207 | 0.2415 | ####### | 766345 | ####### | 35.4802 |
| rs510706 | C | G | 11 | 9E+07 | 0.01071 | 0.0018 | 0.6156 | ####### | 766345 | ####### | 35.4025 |
| rs535307 | G | A | 7 | 1.1E+08 | -0.01 | 0.00184 | 0.6769 | ####### | 766345 | ####### | 29.7736 |
| rs55736314 | G | C | 3 | 7.2E+07 | 0.01431 | 0.00174 | 0.4167 | ####### | 766345 | ####### | 67.6364 |
| rs55771711 | C | G | 10 | 1.3E+08 | 0.01555 | 0.00199 | 0.2279 | ####### | 766345 | ####### | 61.0597 |
| rs56391344 | A | G | 15 | 7.8E+07 | 0.01571 | 0.00197 | 0.2381 | ####### | 766345 | ####### | 63.5946 |
| rs575113 | A | G | 1 | 1.1E+08 | 0.01285 | 0.00186 | 0.2772 | ####### | 766345 | ####### | 47.7288 |
| rs59123361 | A | G | 1 | 1.1E+08 | -0.0209 | 0.00291 | 0.1054 | ####### | 766345 | ####### | 51.7806 |
| rs59480703 | C | G | 8 | 9653130 | -0.0124 | 0.00215 | 0.1633 | ####### | 766345 | ####### | 33.1026 |
| rs60483752 | C | G | 16 | 8.7E+07 | 0.01078 | 0.00172 | 0.5816 | ####### | 766345 | ####### | 39.2808 |
| rs6122735 | T | C | 20 | 4.8E+07 | 0.0105 | 0.00174 | 0.4133 | ####### | 766345 | ####### | 36.415 |
| rs6123924 | G | A | 20 | 5.8E+07 | -0.0153 | 0.00235 | 0.1599 | ####### | 766345 | ####### | 42.2777 |
| rs613872 | T | G | 18 | 5.3E+07 | -0.0175 | 0.00227 | 0.8282 | ####### | 766345 | ####### | 59.4326 |
| rs61747885 | T | G | 4 | 1.4E+08 | 0.01383 | 0.00235 | 0.1412 | ####### | 766345 | ####### | 34.6345 |
| rs62097985 | T | C | 18 | 5.1E+07 | -0.0129 | 0.00172 | 0.4116 | ####### | 766345 | ####### | 56.0757 |
| rs62157915 | C | T | 2 | 9.9E+07 | 0.02091 | 0.00348 | 0.05952 | ####### | 766345 | ####### | 36.1035 |
| rs62183776 | T | C | 2 | 1.7E+08 | -0.0131 | 0.00217 | 0.1905 | ####### | 766345 | ####### | 36.3326 |
| rs62184480 | T | C | 2 | 2.1E+08 | -0.0153 | 0.00191 | 0.2449 | ####### | 766345 | ####### | 64 |
| rs622169 | T | C | 1 | 2.4E+08 | 0.00999 | 0.00178 | 0.4677 | ####### | 766345 | ####### | 31.4986 |
| rs62439690 | A | G | 7 | 2.1E+07 | -0.0109 | 0.00194 | 0.267 | ####### | 766345 | ####### | 31.3946 |
| rs62444881 | T | C | 7 | 2052318 | 0.01815 | 0.00217 | 0.1905 | ####### | 766345 | 0.0001 | 69.9574 |
| rs6493265 | T | C | 15 | 4.8E+07 | -0.0139 | 0.00174 | 0.3895 | ####### | 766345 | ####### | 63.3579 |
| rs6513959 | G | A | 20 | 4.4E+07 | -0.0118 | 0.00185 | 0.2789 | ####### | 766345 | ####### | 40.4771 |
| rs6557171 | C | T | 6 | 1.5E+08 | 0.01567 | 0.00181 | 0.7245 | ####### | 766345 | ####### | 74.9516 |
| rs663234 | G | C | 1 | 5.8E+07 | -0.0101 | 0.00174 | 0.6122 | ####### | 766345 | ####### | 33.3606 |
| rs66568921 | G | T | 3 | 8.6E+07 | 0.01565 | 0.00182 | 0.3639 | ####### | 766345 | 0.00011 | 73.9411 |
| rs6731373 | A | G | 2 | 6.9E+07 | -0.0126 | 0.00181 | 0.3367 | ####### | 766345 | ####### | 48.1529 |
| rs6731967 | C | G | 2 | 2.4E+08 | -0.0119 | 0.00199 | 0.2092 | ####### | 766345 | ####### | 35.5192 |
| rs67885444 | T | C | 8 | 2.9E+07 | 0.01406 | 0.00232 | 0.1718 | ####### | 766345 | ####### | 36.7278 |
| rs67890737 | A | C | 2 | 2.3E+08 | -0.0114 | 0.00179 | 0.3265 | ####### | 766345 | ####### | 40.6317 |
| rs6803651 | T | G | 3 | 6.4E+07 | 0.01131 | 0.00172 | 0.415 | ####### | 766345 | ####### | 43.2383 |
| rs6805241 | C | T | 3 | 1.2E+08 | -0.0141 | 0.00203 | 0.1973 | ####### | 766345 | ####### | 48.4498 |
| rs6867851 | C | G | 5 | 9.2E+07 | -0.012 | 0.00173 | 0.4235 | ####### | 766345 | ####### | 48.1139 |
| rs6938002 | A | G | 6 | 3.8E+07 | -0.0101 | 0.00173 | 0.3963 | ####### | 766345 | ####### | 33.9491 |
| rs6959891 | G | A | 7 | 5.5E+07 | -0.0114 | 0.00189 | 0.2959 | ####### | 766345 | ####### | 36.1271 |
| rs7012546 | T | C | 8 | 1.1E+08 | 0.01009 | 0.00172 | 0.4201 | ####### | 766345 | ####### | 34.4132 |
| rs7016302 | G | C | 8 | 4833041 | 0.01243 | 0.00228 | 0.1769 | ####### | 766345 | ####### | 29.7216 |
| rs702606 | C | T | 5 | 5.3E+07 | -0.0143 | 0.0025 | 0.1701 | ####### | 766345 | ####### | 32.5813 |
| rs7029718 | A | G | 9 | 2.3E+07 | 0.02439 | 0.00174 | 0.4354 | ####### | 766345 | 0.00029 | 196.483 |
| rs7031698 | C | T | 9 | 8.2E+07 | 0.01248 | 0.00206 | 0.7755 | ####### | 766345 | ####### | 36.7024 |
| rs710629 | A | G | 12 | 6.8E+07 | 0.01053 | 0.00177 | 0.6565 | ####### | 766345 | ####### | 35.3924 |
| rs71646142 | T | C | 1 | 2.1E+08 | 0.01286 | 0.00217 | 0.1735 | ####### | 766345 | ####### | 35.1206 |
| rs7233920 | A | G | 18 | 3.7E+07 | -0.0132 | 0.00202 | 0.216 | ####### | 766345 | ####### | 42.3788 |
| rs7257460 | C | T | 19 | 3.1E+07 | -0.0115 | 0.00189 | 0.2704 | ####### | 766345 | ####### | 36.7018 |
| rs7278859 | T | A | 21 | 2E+07 | 0.01013 | 0.00185 | 0.3078 | ####### | 766345 | ####### | 29.983 |
| rs72807818 | A | G | 2 | 5.2E+07 | 0.01915 | 0.00252 | 0.1241 | ####### | 766345 | ####### | 57.7479 |
| rs72828517 | C | T | 6 | 1.9E+07 | 0.01836 | 0.00224 | 0.1412 | ####### | 766345 | ####### | 67.1814 |
| rs72840994 | G | T | 10 | 8.7E+07 | 0.01247 | 0.00216 | 0.182 | ####### | 766345 | ####### | 33.3292 |
| rs730384 | A | G | 14 | 7.5E+07 | 0.01016 | 0.00171 | 0.4558 | ####### | 766345 | ####### | 35.3017 |
| rs7315713 | T | A | 12 | 7.5E+07 | -0.0102 | 0.00187 | 0.6633 | ####### | 766345 | ####### | 29.8689 |
| rs7321274 | G | A | 13 | 6.9E+07 | -0.0128 | 0.00211 | 0.1956 | ####### | 766345 | ####### | 36.5137 |
| rs73301698 | A | G | 12 | 1.6E+07 | -0.0129 | 0.00208 | 0.2262 | ####### | 766345 | ####### | 38.5235 |
| rs7332724 | T | C | 13 | 9.2E+07 | -0.0115 | 0.00189 | 0.2619 | ####### | 766345 | ####### | 36.9587 |
| rs73344830 | G | A | 10 | 1E+08 | -0.0172 | 0.00172 | 0.602 | ####### | 766345 | 0.00014 | 100 |
| rs736282 | C | T | 14 | 9.4E+07 | -0.0108 | 0.0017 | 0.5153 | ####### | 766345 | ####### | 40.5095 |
| rs73874335 | T | C | 3 | 1.6E+08 | -0.0199 | 0.00361 | 0.05952 | ####### | 766345 | ####### | 30.3873 |
| rs743316 | C | T | 21 | 3.5E+07 | -0.0119 | 0.00208 | 0.182 | ####### | 766345 | ####### | 32.4571 |
| rs74643044 | C | T | 5 | 1E+08 | 0.02323 | 0.00386 | 0.02721 | ####### | 766345 | ####### | 36.2179 |
| rs74701752 | T | G | 1 | 2.1E+07 | 0.01591 | 0.00285 | 0.09524 | ####### | 766345 | ####### | 31.1638 |
| rs7481514 | G | A | 11 | 1.3E+08 | 0.01072 | 0.00178 | 0.665 | ####### | 766345 | ####### | 36.2702 |
| rs74998289 | G | T | 17 | 4.4E+07 | -0.0182 | 0.00213 | 0.2398 | ####### | 766345 | 0.00012 | 73.0905 |
| rs7594904 | C | T | 2 | 1E+08 | 0.00969 | 0.00173 | 0.4184 | ####### | 766345 | ####### | 31.3729 |
| rs7603132 | A | G | 2 | 4951548 | 0.01317 | 0.00215 | 0.1548 | ####### | 766345 | ####### | 37.5227 |
| rs76076331 | T | C | 2 | 1.1E+07 | 0.01873 | 0.00248 | 0.131 | ####### | 766345 | ####### | 57.039 |
| rs7650602 | C | T | 3 | 1.4E+08 | 0.00939 | 0.00171 | 0.4286 | ####### | 766345 | ####### | 30.1536 |
| rs76608582 | A | C | 19 | 4474725 | 0.02798 | 0.00445 | 0.04082 | ####### | 766345 | ####### | 39.5344 |
| rs76878669 | G | C | 11 | 6.6E+07 | -0.014 | 0.00205 | 0.2534 | ####### | 766345 | ####### | 46.5723 |
| rs77025239 | A | G | 3 | 1.8E+08 | -0.0142 | 0.00234 | 0.1088 | ####### | 766345 | ####### | 36.929 |
| rs77128898 | T | C | 11 | 6.1E+07 | -0.0277 | 0.00482 | 0.02211 | ####### | 766345 | ####### | 33.0029 |
| rs77702622 | A | G | 8 | 1.4E+08 | -0.0245 | 0.00351 | 0.07653 | ####### | 766345 | ####### | 48.602 |
| rs77719387 | A | T | 3 | 5E+07 | -0.046 | 0.00726 | 0.01701 | ####### | 766345 | ####### | 40.0937 |
| rs77835879 | G | A | 12 | 2.7E+07 | -0.016 | 0.00288 | 0.09014 | ####### | 766345 | ####### | 30.9028 |
| rs7796203 | A | G | 7 | 1.2E+08 | -0.0107 | 0.00171 | 0.5255 | ####### | 766345 | ####### | 39.4472 |
| rs7803932 | A | G | 7 | 7E+07 | 0.0143 | 0.00226 | 0.1565 | ####### | 766345 | ####### | 40.0364 |
| rs7808399 | G | A | 7 | 8075222 | 0.0107 | 0.00171 | 0.5476 | ####### | 766345 | ####### | 39.1539 |
| rs7833201 | C | G | 8 | 8.8E+07 | -0.0153 | 0.00262 | 0.1344 | ####### | 766345 | ####### | 34.1912 |
| rs7863447 | A | G | 9 | 1.4E+07 | 0.01678 | 0.00233 | 0.8333 | ####### | 766345 | ####### | 51.8647 |
| rs78721320 | A | G | 5 | 3304854 | 0.01307 | 0.00219 | 0.2041 | ####### | 766345 | ####### | 35.6175 |
| rs790647 | A | C | 10 | 1.1E+08 | -0.0148 | 0.00202 | 0.2347 | ####### | 766345 | ####### | 53.8262 |
| rs7920624 | T | A | 10 | 6.8E+07 | -0.0118 | 0.0017 | 0.4932 | ####### | 766345 | ####### | 48.2616 |
| rs7924036 | T | G | 10 | 6.5E+07 | 0.01501 | 0.0017 | 0.5391 | ####### | 766345 | 0.00011 | 77.9585 |
| rs79265434 | G | A | 7 | 2.5E+07 | 0.02331 | 0.00262 | 0.1173 | ####### | 766345 | 0.00011 | 79.1557 |
| rs79269403 | A | G | 3 | 1.1E+08 | 0.01447 | 0.00204 | 0.2228 | ####### | 766345 | ####### | 50.3126 |
| rs7928622 | T | A | 11 | 1.2E+07 | 0.01011 | 0.00181 | 0.3078 | ####### | 766345 | ####### | 31.1993 |
| rs795230 | T | C | 11 | 3.1E+07 | 0.00952 | 0.00172 | 0.4184 | ####### | 766345 | ####### | 30.6349 |
| rs79523955 | G | A | 1 | 2.9E+07 | -0.018 | 0.00283 | 0.09524 | ####### | 766345 | ####### | 40.5449 |
| rs7977614 | G | A | 12 | 1.1E+08 | 0.01325 | 0.00198 | 0.3078 | ####### | 766345 | ####### | 44.7818 |
| rs7993663 | C | T | 13 | 5.6E+07 | 0.0118 | 0.00178 | 0.3571 | ####### | 766345 | ####### | 43.9465 |
| rs8008382 | C | T | 14 | 1E+08 | 0.01208 | 0.00185 | 0.6871 | ####### | 766345 | ####### | 42.6374 |
| rs80171383 | A | G | 11 | 4.6E+07 | 0.0145 | 0.00241 | 0.1241 | ####### | 766345 | ####### | 36.1994 |
| rs8020034 | A | G | 14 | 2.7E+07 | 0.01782 | 0.00223 | 0.2058 | ####### | 766345 | 0.0001 | 63.8566 |
| rs818415 | G | T | 16 | 6.5E+07 | 0.01235 | 0.00219 | 0.182 | ####### | 766345 | ####### | 31.8014 |
| rs837080 | C | T | 8 | 1.3E+08 | 0.01092 | 0.0017 | 0.4932 | ####### | 766345 | ####### | 41.2617 |
| rs892612 | C | A | 5 | 1.4E+08 | 0.01464 | 0.00237 | 0.8418 | ####### | 766345 | ####### | 38.158 |
| rs894067 | A | G | 11 | 7.6E+07 | 0.01041 | 0.00175 | 0.3929 | ####### | 766345 | ####### | 35.3855 |
| rs9289300 | C | T | 3 | 1.3E+08 | 0.01512 | 0.00234 | 0.1837 | ####### | 766345 | ####### | 41.7515 |
| rs9320493 | G | A | 6 | 1.1E+08 | -0.0139 | 0.0024 | 0.8639 | ####### | 766345 | ####### | 33.7367 |
| rs9342482 | T | G | 6 | 6.6E+07 | 0.01264 | 0.00197 | 0.2908 | ####### | 766345 | ####### | 41.1682 |
| rs9349956 | C | A | 6 | 1.5E+07 | 0.01881 | 0.00225 | 0.2398 | ####### | 766345 | 0.00013 | 69.8896 |
| rs9372625 | A | G | 6 | 9.8E+07 | 0.02383 | 0.00176 | 0.4133 | ####### | 766345 | 0.00028 | 183.325 |
| rs9384679 | T | C | 6 | 1.1E+08 | -0.0096 | 0.00176 | 0.4082 | ####### | 766345 | ####### | 29.6901 |
| rs9386319 | G | A | 6 | 9.7E+07 | 0.00991 | 0.00174 | 0.4269 | ####### | 766345 | ####### | 32.4376 |
| rs9386787 | G | A | 6 | 1.1E+08 | 0.00958 | 0.0017 | 0.5136 | ####### | 766345 | ####### | 31.7565 |
| rs9436866 | C | A | 1 | 6.9E+07 | 0.01882 | 0.00289 | 0.09524 | ####### | 766345 | ####### | 42.4076 |
| rs9503598 | A | G | 6 | 3446263 | 0.01079 | 0.00171 | 0.4388 | ####### | 766345 | ####### | 39.8154 |
| rs9529119 | G | C | 13 | 3.2E+07 | -0.013 | 0.00204 | 0.8027 | ####### | 766345 | ####### | 40.2976 |
| rs9556958 | T | C | 13 | 9.9E+07 | -0.0108 | 0.0017 | 0.5289 | ####### | 766345 | ####### | 40.3599 |
| rs9616906 | A | G | 22 | 5.1E+07 | 0.01497 | 0.00172 | 0.4235 | ####### | 766345 | 0.00011 | 75.7507 |
| rs9679654 | C | T | 2 | 6.2E+07 | 0.01042 | 0.00172 | 0.4847 | ####### | 766345 | ####### | 36.7011 |
| rs969512 | T | A | 4 | 1.5E+08 | 0.01249 | 0.00179 | 0.2959 | ####### | 766345 | ####### | 48.6877 |
| rs9704097 | A | C | 11 | 2.5E+07 | -0.0103 | 0.00171 | 0.4728 | ####### | 766345 | ####### | 36.2812 |
| rs9882532 | C | T | 3 | 1.7E+07 | -0.0121 | 0.00177 | 0.3639 | ####### | 766345 | ####### | 46.5787 |
| rs9914918 | A | G | 17 | 4.7E+07 | 0.01155 | 0.00189 | 0.2823 | ####### | 766345 | ####### | 37.3457 |
| rs9933256 | G | A | 16 | 1246748 | -0.0113 | 0.00172 | 0.4082 | ####### | 766345 | ####### | 43.468 |
| rs9936270 | T | C | 16 | 1.2E+07 | -0.0136 | 0.00198 | 0.3078 | ####### | 766345 | ####### | 47.1789 |
| rs9938678 | T | A | 16 | 6.2E+07 | 0.01355 | 0.00205 | 0.2432 | ####### | 766345 | ####### | 43.6889 |
| rs9964724 | T | C | 18 | 3.5E+07 | 0.01978 | 0.00183 | 0.6599 | ####### | 766345 | 0.00018 | 116.829 |
| rs9995567 | A | G | 4 | 1.6E+08 | 0.00998 | 0.00178 | 0.3827 | ####### | 766345 | ####### | 31.4356 |

| **Supplementary table 2. Intelligence instrument variable details and F statistics.** | | | | | | | | |  |
| --- | --- | --- | --- | --- | --- | --- | --- | --- | --- |
| **SNP** | **effect_allele.exposure** | **other_allele.exposure** | **chr.exposure** | **pos.exposure** | **beta.exposure** | **se.exposure** | **pval.exposure** | **samplesize.exposure** | **F** |
| rs1007934 | A | G | 14 | 7.3E+07 | 0.01608 | 0.00281 | ####### | 269867 | 32.8328 |
| rs10189857 | G | A | 2 | 6.1E+07 | -0.019 | 0.00275 | ####### | 269867 | 47.7206 |
| rs10189912 | G | A | 2 | 1.4E+08 | 0.01934 | 0.00285 | ####### | 269867 | 45.9412 |
| rs1054442 | C | A | 12 | 4.9E+07 | 0.02146 | 0.00282 | ####### | 269867 | 58.0798 |
| rs10779271 | G | A | 1 | 2.2E+08 | -0.0164 | 0.00293 | ####### | 269867 | 31.3376 |
| rs10917152 | T | C | 1 | 2.2E+07 | 0.02421 | 0.00405 | ####### | 269867 | 35.7603 |
| rs10954779 | T | C | 8 | 3.1E+07 | -0.0164 | 0.00276 | ####### | 269867 | 35.1648 |
| rs11076962 | C | T | 16 | 5811367 | -0.0169 | 0.00304 | ####### | 269867 | 31.0025 |
| rs11079849 | T | C | 17 | 4.7E+07 | 0.01655 | 0.00296 | ####### | 269867 | 31.2593 |
| rs11210871 | G | C | 1 | 4.4E+07 | 0.01735 | 0.00293 | ####### | 269867 | 35.1295 |
| rs112780312 | A | G | 1 | 1.5E+08 | -0.0183 | 0.0031 | ####### | 269867 | 34.7983 |
| rs1145123 | C | T | 5 | 1.1E+08 | -0.0206 | 0.00277 | ####### | 269867 | 55.0116 |
| rs115064 | C | T | 7 | 2.4E+07 | -0.0161 | 0.00281 | ####### | 269867 | 32.7184 |
| rs11605348 | A | G | 11 | 4.8E+07 | -0.0166 | 0.0029 | ####### | 269867 | 32.8902 |
| rs11623436 | T | C | 14 | 3.7E+07 | -0.0158 | 0.00275 | ####### | 269867 | 32.9018 |
| rs11634187 | G | T | 15 | 4.1E+07 | -0.022 | 0.00386 | ####### | 269867 | 32.627 |
| rs11646221 | T | G | 16 | 7666088 | 0.01774 | 0.00277 | ####### | 269867 | 40.9472 |
| rs11678106 | T | C | 2 | 8.2E+07 | 0.01609 | 0.00275 | ####### | 269867 | 34.3395 |
| rs11720523 | A | C | 3 | 7.2E+07 | 0.01833 | 0.00277 | ####### | 269867 | 43.6656 |
| rs11793831 | T | G | 9 | 2.3E+07 | 0.02783 | 0.0028 | ####### | 269867 | 98.5058 |
| rs11898362 | A | G | 2 | 7.4E+07 | -0.018 | 0.00301 | ####### | 269867 | 35.7365 |
| rs12026245 | A | G | 1 | 1E+08 | -0.0179 | 0.00273 | ####### | 269867 | 42.811 |
| rs12035012 | A | C | 1 | 4.2E+07 | -0.027 | 0.00331 | ####### | 269867 | 66.4059 |
| rs12190777 | G | A | 6 | 9.8E+07 | -0.017 | 0.00309 | ####### | 269867 | 30.3379 |
| rs1233578 | G | A | 6 | 2.9E+07 | 0.0238 | 0.0039 | ####### | 269867 | 37.1854 |
| rs12470949 | C | T | 2 | 2.4E+07 | 0.01717 | 0.00302 | ####### | 269867 | 32.2965 |
| rs12535854 | G | C | 7 | 1.1E+08 | 0.01823 | 0.00295 | ####### | 269867 | 38.0938 |
| rs12646225 | T | C | 4 | 696848 | 0.02513 | 0.00422 | ####### | 269867 | 35.5335 |
| rs1280049 | C | A | 6 | 7.7E+07 | -0.015 | 0.00273 | ####### | 269867 | 30.1839 |
| rs12886584 | C | T | 14 | 4.1E+07 | -0.0205 | 0.00357 | ####### | 269867 | 32.9591 |
| rs13024268 | A | G | 2 | 2.3E+08 | -0.0167 | 0.00288 | ####### | 269867 | 33.4895 |
| rs13071190 | C | T | 3 | 1.4E+08 | -0.0181 | 0.00292 | ####### | 269867 | 38.4773 |
| rs13165296 | C | A | 5 | 6E+07 | -0.0196 | 0.00352 | ####### | 269867 | 31.1141 |
| rs13212044 | T | G | 6 | 1.3E+08 | -0.0184 | 0.00324 | ####### | 269867 | 32.1035 |
| rs13223152 | G | A | 7 | 7E+07 | -0.0176 | 0.00278 | ####### | 269867 | 40.1702 |
| rs13253386 | G | T | 8 | 1.4E+07 | 0.02013 | 0.00275 | ####### | 269867 | 53.6704 |
| rs13276212 | T | G | 8 | 6.6E+07 | 0.01507 | 0.00275 | ####### | 269867 | 29.9317 |
| rs13395129 | C | G | 2 | 6.4E+07 | 0.01687 | 0.003 | ####### | 269867 | 31.6744 |
| rs1362739 | A | C | 7 | 1.3E+08 | 0.02095 | 0.00273 | ####### | 269867 | 58.7061 |
| rs1369429 | C | T | 15 | 8.8E+07 | -0.0176 | 0.0029 | ####### | 269867 | 37.0639 |
| rs1408579 | T | C | 10 | 1E+08 | 0.01605 | 0.00275 | ####### | 269867 | 34.1055 |
| rs144026674 | T | C | 19 | 4E+07 | 0.04131 | 0.00747 | ####### | 269867 | 30.5919 |
| rs144246 | A | G | 4 | 1.7E+07 | 0.01549 | 0.00284 | ####### | 269867 | 29.7572 |
| rs1589652 | G | A | 3 | 3.6E+07 | -0.0171 | 0.00276 | ####### | 269867 | 38.3783 |
| rs166820 | A | G | 5 | 8.9E+07 | 0.02433 | 0.0036 | ####### | 269867 | 45.7113 |
| rs17002025 | A | G | 19 | 1.3E+07 | 0.0256 | 0.00426 | ####### | 269867 | 36.084 |
| rs17106817 | C | T | 14 | 7E+07 | -0.0169 | 0.00302 | ####### | 269867 | 31.2593 |
| rs17128425 | A | T | 11 | 1.2E+08 | 0.02556 | 0.00454 | ####### | 269867 | 31.6294 |
| rs17199964 | A | G | 4 | 1E+08 | -0.0392 | 0.00571 | ####### | 269867 | 47.1144 |
| rs1727307 | G | A | 12 | 1.2E+08 | -0.0178 | 0.00301 | ####### | 269867 | 35.1173 |
| rs17698176 | G | T | 17 | 4.5E+07 | 0.02011 | 0.00357 | ####### | 269867 | 31.8095 |
| rs1812587 | T | G | 5 | 6.3E+07 | -0.0173 | 0.00277 | ####### | 269867 | 39.2752 |
| rs1831539 | C | T | 1 | 6E+07 | 0.0172 | 0.00276 | ####### | 269867 | 38.7881 |
| rs1840847 | A | G | 5 | 1.3E+07 | 0.01634 | 0.00288 | ####### | 269867 | 32.1375 |
| rs1906252 | A | C | 6 | 9.9E+07 | 0.03166 | 0.00274 | ####### | 269867 | 133.38 |
| rs190925241 | A | T | 1 | 9.9E+07 | 0.03343 | 0.00547 | ####### | 269867 | 37.3688 |
| rs1962047 | A | G | 12 | 5.8E+07 | -0.0195 | 0.00286 | ####### | 269867 | 46.5534 |
| rs1972860 | A | G | 4 | 9.5E+07 | -0.0176 | 0.00293 | ####### | 269867 | 35.8921 |
| rs2007176 | C | T | 2 | 2E+08 | -0.0154 | 0.00277 | ####### | 269867 | 30.9583 |
| rs2008514 | A | G | 16 | 2.9E+07 | -0.0287 | 0.0028 | ####### | 269867 | 104.96 |
| rs2071407 | C | T | 14 | 1E+08 | 0.02197 | 0.00286 | ####### | 269867 | 59.0745 |
| rs2072490 | T | C | 19 | 1.8E+07 | 0.017 | 0.00274 | ####### | 269867 | 38.341 |
| rs2111490 | G | A | 8 | 1E+08 | -0.0155 | 0.00275 | ####### | 269867 | 31.6631 |
| rs2239647 | C | A | 14 | 3.3E+07 | 0.02054 | 0.00277 | ####### | 269867 | 55.1157 |
| rs2268894 | T | C | 2 | 1.6E+08 | 0.02078 | 0.00275 | ####### | 269867 | 57.1841 |
| rs2285640 | A | G | 17 | 3.5E+07 | 0.01751 | 0.00276 | ####### | 269867 | 40.1323 |
| rs2309812 | T | C | 2 | 1E+08 | 0.02284 | 0.00284 | ####### | 269867 | 64.4331 |
| rs2352974 | T | C | 3 | 5E+07 | -0.0308 | 0.00275 | ####### | 269867 | 125.642 |
| rs2373353 | G | A | 11 | 7.9E+07 | 0.01632 | 0.00289 | ####### | 269867 | 31.9792 |
| rs2393967 | C | A | 10 | 6.5E+07 | 0.01871 | 0.00296 | ####### | 269867 | 39.879 |
| rs2420551 | A | T | 1 | 6.9E+07 | -0.0286 | 0.00434 | ####### | 269867 | 43.3359 |
| rs2450333 | A | G | 5 | 1.8E+08 | -0.0188 | 0.0028 | ####### | 269867 | 45.2522 |
| rs2457192 | A | C | 16 | 1.2E+07 | -0.0198 | 0.00313 | ####### | 269867 | 39.7907 |
| rs2478286 | C | G | 13 | 1.1E+08 | -0.0258 | 0.00313 | ####### | 269867 | 67.9966 |
| rs2508713 | A | T | 11 | 9.6E+07 | 0.01653 | 0.00284 | ####### | 269867 | 33.8608 |
| rs2558096 | G | T | 2 | 1.4E+08 | 0.01563 | 0.00277 | ####### | 269867 | 31.7646 |
| rs2647995 | C | T | 16 | 5.2E+07 | 0.01975 | 0.00304 | ####### | 269867 | 42.0939 |
| rs2678210 | C | T | 1 | 2E+08 | -0.0188 | 0.00305 | ####### | 269867 | 38.0319 |
| rs2721173 | T | C | 8 | 1.5E+08 | -0.0162 | 0.00273 | ####### | 269867 | 35.2597 |
| rs2726491 | A | G | 4 | 1.1E+08 | -0.0283 | 0.00286 | ####### | 269867 | 98.0097 |
| rs2836921 | A | G | 21 | 4.1E+07 | 0.02035 | 0.00296 | ####### | 269867 | 47.156 |
| rs28620532 | G | A | 9 | 9.8E+07 | 0.01635 | 0.00289 | ####### | 269867 | 32.0357 |
| rs287879 | G | A | 6 | 1.6E+08 | 0.01887 | 0.00307 | ####### | 269867 | 37.6502 |
| rs2885208 | C | T | 11 | 1.1E+08 | -0.0189 | 0.00346 | ####### | 269867 | 29.8881 |
| rs2920940 | C | T | 8 | 9.3E+07 | 0.02474 | 0.00325 | ####### | 269867 | 57.8966 |
| rs2955280 | T | C | 2 | 4.4E+07 | -0.0149 | 0.00273 | ####### | 269867 | 29.7571 |
| rs297578 | A | G | 2 | 1.6E+08 | 0.0181 | 0.00301 | ####### | 269867 | 36.2525 |
| rs2987390 | G | C | 9 | 1.3E+08 | 0.0178 | 0.00312 | ####### | 269867 | 32.5016 |
| rs3128341 | C | T | 1 | 7.3E+07 | 0.03173 | 0.00342 | ####### | 269867 | 86.1924 |
| rs31768 | T | A | 5 | 1.7E+08 | -0.0182 | 0.00305 | ####### | 269867 | 35.4263 |
| rs329672 | T | C | 11 | 1.3E+08 | 0.01743 | 0.00285 | ####### | 269867 | 37.3199 |
| rs34316 | C | A | 5 | 8.8E+07 | -0.021 | 0.00277 | ####### | 269867 | 57.8508 |
| rs34320898 | C | G | 1 | 1.7E+08 | 0.02287 | 0.00384 | ####### | 269867 | 35.3905 |
| rs34811474 | A | G | 4 | 2.5E+07 | 0.029 | 0.00359 | ####### | 269867 | 65.0926 |
| rs35608616 | A | G | 10 | 1.3E+08 | -0.0181 | 0.00294 | ####### | 269867 | 37.9335 |
| rs35731967 | C | T | 2 | 2E+08 | -0.0218 | 0.00366 | ####### | 269867 | 35.6409 |
| rs36033 | C | T | 5 | 6.1E+07 | -0.016 | 0.00279 | ####### | 269867 | 32.8099 |
| rs3740422 | C | G | 10 | 1E+08 | -0.0241 | 0.00291 | ####### | 269867 | 68.5253 |
| rs3843954 | C | G | 13 | 5.9E+07 | -0.0208 | 0.00334 | ####### | 269867 | 38.5641 |
| rs3860537 | C | T | 3 | 1.1E+08 | -0.0189 | 0.0034 | ####### | 269867 | 30.7137 |
| rs405321 | A | G | 5 | 1.1E+08 | -0.0164 | 0.00297 | ####### | 269867 | 30.5145 |
| rs4463213 | A | G | 5 | 1.4E+08 | 0.01907 | 0.00273 | ####### | 269867 | 48.6925 |
| rs4484297 | C | G | 4 | 1.6E+07 | 0.01827 | 0.00316 | ####### | 269867 | 33.4086 |
| rs4667954 | C | T | 2 | 1.6E+08 | -0.0173 | 0.00304 | ####### | 269867 | 32.4898 |
| rs4725065 | G | A | 7 | 8109522 | 0.01653 | 0.00274 | ####### | 269867 | 36.5058 |
| rs4731392 | G | A | 7 | 1.3E+08 | 0.02174 | 0.00297 | ####### | 269867 | 53.4215 |
| rs4793161 | G | A | 17 | 4.3E+07 | 0.01772 | 0.00325 | ####### | 269867 | 29.7242 |
| rs4821995 | G | A | 22 | 4.1E+07 | -0.016 | 0.00287 | ####### | 269867 | 30.9692 |
| rs4852252 | C | T | 2 | 7.2E+07 | 0.02079 | 0.00275 | ####### | 269867 | 57.2443 |
| rs4976976 | A | G | 8 | 1.4E+08 | 0.01732 | 0.00278 | ####### | 269867 | 38.8752 |
| rs4981713 | G | T | 14 | 3E+07 | -0.0162 | 0.0028 | ####### | 269867 | 33.4776 |
| rs55754731 | C | T | 12 | 1.6E+07 | -0.0214 | 0.00367 | ####### | 269867 | 33.8142 |
| rs55763037 | G | A | 5 | 9.3E+07 | -0.0183 | 0.00333 | ####### | 269867 | 30.283 |
| rs56150095 | A | C | 7 | 7.2E+07 | -0.022 | 0.00275 | ####### | 269867 | 63.9518 |
| rs566237 | G | A | 6 | 1.2E+07 | 0.01872 | 0.00294 | ####### | 269867 | 40.6533 |
| rs5750830 | A | C | 22 | 4E+07 | 0.02289 | 0.00313 | ####### | 269867 | 53.5971 |
| rs58593843 | A | G | 2 | 6E+07 | -0.0277 | 0.00465 | ####### | 269867 | 35.4143 |
| rs59142272 | A | G | 3 | 1.4E+08 | 0.0227 | 0.00368 | ####### | 269867 | 37.9333 |
| rs600806 | A | G | 1 | 1.1E+08 | -0.0193 | 0.00308 | ####### | 269867 | 39.3381 |
| rs6019535 | A | G | 20 | 4.8E+07 | 0.02511 | 0.00298 | ####### | 269867 | 71.1658 |
| rs60262711 | T | C | 2 | 1.2E+08 | 0.01595 | 0.00283 | ####### | 269867 | 31.8662 |
| rs62181012 | C | T | 2 | 1.8E+08 | -0.0211 | 0.00351 | ####### | 269867 | 36.2644 |
| rs62198803 | A | G | 2 | 1.9E+08 | 0.01907 | 0.00323 | ####### | 269867 | 34.9397 |
| rs6508220 | G | A | 18 | 5.1E+07 | 0.02275 | 0.00274 | ####### | 269867 | 69.0558 |
| rs6535809 | G | A | 4 | 1.5E+08 | -0.0196 | 0.00273 | ####### | 269867 | 51.6384 |
| rs6539284 | C | T | 12 | 8E+07 | 0.01948 | 0.00283 | ####### | 269867 | 47.4723 |
| rs6550835 | A | G | 3 | 2.4E+07 | -0.0248 | 0.00293 | ####### | 269867 | 71.7579 |
| rs6668048 | T | C | 1 | 9.6E+07 | -0.0215 | 0.00273 | ####### | 269867 | 61.5911 |
| rs66954617 | G | A | 17 | 5.7E+07 | 0.02088 | 0.00283 | ####### | 269867 | 54.3023 |
| rs67482514 | G | C | 4 | 6.6E+07 | 0.01786 | 0.00323 | ####### | 269867 | 30.5809 |
| rs6770622 | A | G | 3 | 8.5E+07 | -0.045 | 0.00686 | ####### | 269867 | 42.9026 |
| rs6819372 | G | A | 4 | 6.8E+07 | 0.0198 | 0.00273 | ####### | 269867 | 52.6352 |
| rs6860963 | T | C | 5 | 1.7E+08 | 0.02026 | 0.00348 | ####### | 269867 | 33.9773 |
| rs6903716 | G | A | 6 | 2.2E+07 | -0.0178 | 0.00298 | ####### | 269867 | 35.6287 |
| rs702222 | T | C | 9 | 2.4E+07 | -0.0198 | 0.00287 | ####### | 269867 | 47.679 |
| rs7069887 | C | A | 10 | 3E+07 | -0.0225 | 0.0039 | ####### | 269867 | 33.4201 |
| rs7116046 | T | C | 11 | 1.1E+08 | 0.01571 | 0.00284 | ####### | 269867 | 30.5367 |
| rs7172979 | T | G | 15 | 5.2E+07 | 0.06063 | 0.00908 | ####### | 269867 | 44.5556 |
| rs7248006 | C | T | 19 | 3.2E+07 | 0.01918 | 0.00282 | ####### | 269867 | 46.2263 |
| rs72739469 | C | T | 15 | 6.6E+07 | 0.03436 | 0.00565 | ####### | 269867 | 37.0028 |
| rs72768642 | C | T | 16 | 2.5E+07 | 0.03062 | 0.0054 | ####### | 269867 | 32.1149 |
| rs73068339 | C | G | 19 | 5.9E+07 | 0.01886 | 0.00305 | ####### | 269867 | 38.341 |
| rs7312919 | G | C | 12 | 9.3E+07 | -0.0181 | 0.00291 | ####### | 269867 | 38.7505 |
| rs7357604 | G | A | 8 | 1.4E+08 | -0.0157 | 0.00282 | ####### | 269867 | 31.0139 |
| rs7573001 | C | G | 2 | 2E+08 | -0.0163 | 0.00286 | ####### | 269867 | 32.2966 |
| rs75973558 | G | A | 5 | 2.7E+07 | -0.0256 | 0.00447 | ####### | 269867 | 32.959 |
| rs7640196 | T | C | 3 | 5.4E+07 | -0.0175 | 0.00315 | ####### | 269867 | 30.6142 |
| rs7652296 | G | A | 3 | 9E+07 | -0.0165 | 0.0028 | ####### | 269867 | 34.8807 |
| rs7731260 | A | G | 5 | 1.1E+08 | 0.0153 | 0.00274 | ####### | 269867 | 31.0693 |
| rs78084033 | C | A | 20 | 3.4E+07 | 0.02288 | 0.00405 | ####### | 269867 | 31.9113 |
| rs7941785 | G | A | 11 | 6.4E+07 | -0.0155 | 0.00284 | ####### | 269867 | 29.8115 |
| rs799444 | C | T | 7 | 4.5E+07 | -0.0184 | 0.00276 | ####### | 269867 | 44.5424 |
| rs8006700 | A | T | 14 | 2.7E+07 | -0.0182 | 0.00293 | ####### | 269867 | 38.6881 |
| rs80170948 | G | T | 5 | 6.4E+07 | -0.0454 | 0.00738 | ####### | 269867 | 37.8348 |
| rs8025964 | A | G | 15 | 8.3E+07 | 0.01703 | 0.00275 | ####### | 269867 | 38.3903 |
| rs8051038 | A | G | 16 | 7.2E+07 | 0.01892 | 0.00315 | ####### | 269867 | 36.1923 |
| rs8054299 | G | C | 16 | 5.3E+07 | 0.02301 | 0.00293 | ####### | 269867 | 61.7795 |
| rs889169 | A | G | 19 | 4.8E+07 | 0.01607 | 0.00289 | ####### | 269867 | 30.8804 |
| rs913264 | T | C | 9 | 1.3E+08 | 0.01972 | 0.00303 | ####### | 269867 | 42.4972 |
| rs9384679 | T | C | 6 | 1.1E+08 | -0.0267 | 0.00278 | ####### | 269867 | 92.1786 |
| rs9503599 | C | T | 6 | 3451048 | 0.01711 | 0.00278 | ####### | 269867 | 37.7488 |
| rs9516855 | G | A | 13 | 9.8E+07 | -0.0334 | 0.0061 | ####### | 269867 | 30.0633 |
| rs9569206 | G | A | 13 | 5.6E+07 | 0.01541 | 0.00282 | ####### | 269867 | 29.7789 |
| rs967569 | T | C | 2 | 4.2E+07 | -0.018 | 0.00293 | ####### | 269867 | 37.7119 |
| rs9888986 | A | G | 16 | 6.8E+07 | -0.0235 | 0.00426 | ####### | 269867 | 30.4041 |

| **Supplementary table 3. Detailed information on educational level instrumental variables in outcomes** | | | | | | | | | | |  |  |  |  |  |  |  |  |  |  |  |  |  |  |  |  |  |  |  |  |  |  |  |  |  |  |  |  |  |  |  |  |  |  |  |  |  |  |  |  |  |  |  |  |  |  |  |  |  |  |  |  |
| --- | --- | --- | --- | --- | --- | --- | --- | --- | --- | --- | --- | --- | --- | --- | --- | --- | --- | --- | --- | --- | --- | --- | --- | --- | --- | --- | --- | --- | --- | --- | --- | --- | --- | --- | --- | --- | --- | --- | --- | --- | --- | --- | --- | --- | --- | --- | --- | --- | --- | --- | --- | --- | --- | --- | --- | --- | --- | --- | --- | --- | --- | --- |
| **Outcome** | **SNP** | **effect_allele.outcome** | **other_allele.outcome** | **beta.outcome** | **pval.outcome** | **se.outcome** | **Outcome** | **SNP** | **effect_allele.outcome** | **other_allele.outcome** | **beta.outcome** | **pval.outcome** | **se.outcome** | **Outcome** | **SNP** | **effect_allele.outcome** | **other_allele.outcome** | **beta.outcome** | **pval.outcome** | **se.outcome** | **Outcome** | **SNP** | **effect_allele.outcome** | **other_allele.outcome** | **beta.outcome** | **pval.outcome** | **se.outcome** | **Outcome** | **SNP** | **effect_allele.outcome** | **other_allele.outcome** | **beta.outcome** | **pval.outcome** | **se.outcome** | **Outcome** | **SNP** | **effect_allele.outcome** | **other_allele.outcome** | **beta.outcome** | **pval.outcome** | **se.outcome** | **Outcome** | **SNP** | **effect_allele.outcome** | **other_allele.outcome** | **beta.outcome** | **pval.outcome** | **se.outcome** | **Outcome** | **SNP** | **effect_allele.outcome** | **other_allele.outcome** | **beta.outcome** | **pval.outcome** | **se.outcome** | **Outcome** | **SNP** | **effect_allele.outcome** | **other_allele.outcome** | **beta.outcome** | **pval.outcome** | **se.outcome**Outcome SNP effect_allele.outcome other_allele.outcome beta.outcome pval.outcome se.outcome Outcome SNP effect_allele.outcome other_allele.outcome beta.outcome pval.outcome se.outcome Outcome SNP effect_allele.outcome other_allele.outcome beta.outcome pval.outcome se.outcome Outcome SNP effect_allele.outcome other_allele.outcome beta.outcome pval.outcome se.outcome Outcome SNP effect_allele.outcome other_allele.outcome beta.outcome pval.outcome se.outcome Outcome SNP effect_allele.outcome other_allele.outcome beta.outcome pval.outcome se.outcome Outcome SNP effect_allele.outcome other_allele.outcome beta.outcome pval.outcome se.outcome Outcome SNP effect_allele.outcome other_allele.outcome beta.outcome pval.outcome se.outcome Outcome SNP effect_allele.outcome other_allele.outcome beta.outcome pval.outcome se.outcome Outcome SNP effect_allele.outcome other_allele.outcome beta.outcome pval.outcome se.outcome Outcome SNP effect_allele.outcome other_allele.outcome beta.outcome pval.outcome se.outcome |
| Gastroesophageal reflux | rs10073890 | G | A | 0.00867 | 0.46539 | 0.01188 | Esophageal carcinoma | rs10073890 | G | A | -0.0076 | 0.92168 | 0.07702 | Acute gastritis | rs10073890 | G | A | 0.05248 | 0.16031 | 0.03737 | Chronic gastritis | rs10073890 | G | A | 0.0259 | 0.17278 | 0.01899 | Gastroduodenal ulcer | rs10073890 | G | A | 0.00495 | 0.79884 | 0.01941 | Gastric cancer | rs10073890 | G | A | 0.00177 | 0.97161 | 0.04969 | Fatty liver | rs10073890 | G | A | 0.0224 | 0.56939 | 0.03938 | Hepatic fibrosis | rs10073890 | G | A | 0.11183 | 0.45249 | 0.14885 | Cirrhosis | rs10073890 | G | A | 0.00797 | 0.78356 | 0.02902liver cancer rs10073890 G A -0.0524 0.43992 0.06789 Choleliths rs10073890 G A 0.02469 0.01407 0.01005 Acute pancreatitis rs10073890 G A 0.01523 0.51523 0.02341 Chronic pancreatitis rs10073890 G A 0.05126 0.10338 0.03147 Pancreatic cancer rs10073890 G A -0.1212 0.01296 0.04879 Ulcerative colitis rs10073890 G A -0.0203 0.43259 0.02591 Crohn's disease rs10073890 G A 0.06104 0.17157 0.04465 Irrtable bowel syndrome rs10073890 G A 0.00266 0.89092 0.0194 Acute appendicitis rs10073890 G A 0.01705 0.1262 0.01115 Colon cancer rs10073890 G A -0.0203 0.50657 0.03054 Rectal cancer rs10073890 G A -0.0795 0.04165 0.03901 |
| Gastroesophageal reflux | rs1008078 | T | C | -0.0076 | 0.44396 | 0.00992 | Esophageal carcinoma | rs1008078 | T | C | -0.1033 | 0.10808 | 0.06427 | Acute gastritis | rs1008078 | T | C | 0.00692 | 0.82494 | 0.03129 | Chronic gastritis | rs1008078 | T | C | -0.008 | 0.6126 | 0.01584 | Gastroduodenal ulcer | rs1008078 | T | C | 0.00293 | 0.85638 | 0.0162 | Gastric cancer | rs1008078 | T | C | 0.03124 | 0.44817 | 0.04119 | Fatty liver | rs1008078 | T | C | 0.01384 | 0.67462 | 0.03297 | Hepatic fibrosis | rs1008078 | T | C | 0.06002 | 0.63333 | 0.12581 | Cirrhosis | rs1008078 | T | C | 0.05707 | 0.01872 | 0.02428liver cancer rs1008078 T C 0.04611 0.41499 0.05657 Choleliths rs1008078 T C 0.01979 0.01822 0.00838 Acute pancreatitis rs1008078 T C 0.03722 0.05703 0.01956 Chronic pancreatitis rs1008078 T C 0.04588 0.08171 0.02635 Pancreatic cancer rs1008078 T C 0.00797 0.84488 0.04071 Ulcerative colitis rs1008078 T C 0.01739 0.42231 0.02168 Crohn's disease rs1008078 T C -0.0066 0.86001 0.03741 Irrtable bowel syndrome rs1008078 T C 0.02039 0.20863 0.01621 Acute appendicitis rs1008078 T C 0.00989 0.28869 0.00932 Colon cancer rs1008078 T C -0.0314 0.21669 0.02539 Rectal cancer rs1008078 T C 0.00517 0.87245 0.03222 |
| Gastroesophageal reflux | rs10189857 | G | A | -0.0015 | 0.87641 | 0.00982 | Esophageal carcinoma | rs10189857 | G | A | 0.16084 | 0.00948 | 0.062 | Acute gastritis | rs10189857 | G | A | -0.0203 | 0.5133 | 0.03098 | Chronic gastritis | rs10189857 | G | A | 0.01462 | 0.35165 | 0.01569 | Gastroduodenal ulcer | rs10189857 | G | A | 0.01336 | 0.40479 | 0.01604 | Gastric cancer | rs10189857 | G | A | 0.04047 | 0.32121 | 0.0408 | Fatty liver | rs10189857 | G | A | -0.0705 | 0.03068 | 0.03264 | Hepatic fibrosis | rs10189857 | G | A | 0.00572 | 0.96335 | 0.12439 | Cirrhosis | rs10189857 | G | A | -0.0016 | 0.94549 | 0.024liver cancer rs10189857 G A -0.0582 0.29927 0.05602 Choleliths rs10189857 G A 0.00133 0.87269 0.00829 Acute pancreatitis rs10189857 G A 0.00065 0.97318 0.01935 Chronic pancreatitis rs10189857 G A -0.0046 0.86027 0.02608 Pancreatic cancer rs10189857 G A 0.01769 0.66105 0.04035 Ulcerative colitis rs10189857 G A -0.0308 0.15038 0.02143 Crohn's disease rs10189857 G A -0.0635 0.08625 0.037 Irrtable bowel syndrome rs10189857 G A -0.0046 0.77307 0.01605 Acute appendicitis rs10189857 G A 0.01828 0.04759 0.00923 Colon cancer rs10189857 G A -0.0125 0.6192 0.0252 Rectal cancer rs10189857 G A -0.0058 0.85512 0.03199 |
| Gastroesophageal reflux | rs10205801 | A | G | 0.01586 | 0.10524 | 0.00979 | Esophageal carcinoma | rs10205801 | A | G | -0.0979 | 0.12284 | 0.06343 | Acute gastritis | rs10205801 | A | G | -0.0196 | 0.52464 | 0.03088 | Chronic gastritis | rs10205801 | A | G | 0.00182 | 0.90716 | 0.01562 | Gastroduodenal ulcer | rs10205801 | A | G | 0.01483 | 0.35401 | 0.016 | Gastric cancer | rs10205801 | A | G | -0.0669 | 0.10133 | 0.0408 | Fatty liver | rs10205801 | A | G | -0.0217 | 0.50387 | 0.03252 | Hepatic fibrosis | rs10205801 | A | G | -0.0594 | 0.6316 | 0.12391 | Cirrhosis | rs10205801 | A | G | -0.0048 | 0.84233 | 0.02396liver cancer rs10205801 A G -0.0267 0.63258 0.05595 Choleliths rs10205801 A G 0.00909 0.27154 0.00827 Acute pancreatitis rs10205801 A G 0.01257 0.51487 0.01931 Chronic pancreatitis rs10205801 A G 0.03271 0.20849 0.02601 Pancreatic cancer rs10205801 A G -0.0344 0.39316 0.04031 Ulcerative colitis rs10205801 A G 0.03201 0.13489 0.02141 Crohn's disease rs10205801 A G 0.00363 0.92164 0.03694 Irrtable bowel syndrome rs10205801 A G 0.00373 0.8155 0.016 Acute appendicitis rs10205801 A G -0.0142 0.12367 0.00921 Colon cancer rs10205801 A G 0.02739 0.27606 0.02514 Rectal cancer rs10205801 A G 0.05404 0.09028 0.0319 |
| Gastroesophageal reflux | rs10215082 | A | G | 0.01783 | 0.06932 | 0.00982 | Esophageal carcinoma | rs10215082 | A | G | 0.09244 | 0.14574 | 0.06355 | Acute gastritis | rs10215082 | A | G | 0.01376 | 0.65729 | 0.031 | Chronic gastritis | rs10215082 | A | G | 0.01119 | 0.47601 | 0.01569 | Gastroduodenal ulcer | rs10215082 | A | G | 0.02973 | 0.06418 | 0.01606 | Gastric cancer | rs10215082 | A | G | -0.0148 | 0.71744 | 0.04089 | Fatty liver | rs10215082 | A | G | 0.03483 | 0.2853 | 0.0326 | Hepatic fibrosis | rs10215082 | A | G | 0.01205 | 0.92274 | 0.12423 | Cirrhosis | rs10215082 | A | G | 0.02153 | 0.37 | 0.02402liver cancer rs10215082 A G 0.00327 0.9534 0.05597 Choleliths rs10215082 A G 0.00022 0.97843 0.0083 Acute pancreatitis rs10215082 A G 0.03512 0.06964 0.01936 Chronic pancreatitis rs10215082 A G 0.02027 0.43686 0.02607 Pancreatic cancer rs10215082 A G -0.0353 0.38311 0.04047 Ulcerative colitis rs10215082 A G 0.01132 0.59735 0.02143 Crohn's disease rs10215082 A G 0.04278 0.24697 0.03695 Irrtable bowel syndrome rs10215082 A G 0.0302 0.05993 0.01605 Acute appendicitis rs10215082 A G -0.001 0.91411 0.00922 Colon cancer rs10215082 A G -0.0232 0.35678 0.02521 Rectal cancer rs10215082 A G -0.0148 0.64219 0.03194 |
| Gastroesophageal reflux | rs10240905 | T | C | 0.00974 | 0.3625 | 0.0107 | Esophageal carcinoma | rs10240905 | T | C | -0.0222 | 0.74778 | 0.06918 | Acute gastritis | rs10240905 | T | C | 0.02381 | 0.48105 | 0.0338 | Chronic gastritis | rs10240905 | T | C | -0.0041 | 0.81073 | 0.0171 | Gastroduodenal ulcer | rs10240905 | T | C | 0.00323 | 0.85352 | 0.01752 | Gastric cancer | rs10240905 | T | C | 0.01929 | 0.66214 | 0.04416 | Fatty liver | rs10240905 | T | C | 0.05275 | 0.13778 | 0.03554 | Hepatic fibrosis | rs10240905 | T | C | 0.12894 | 0.34607 | 0.13684 | Cirrhosis | rs10240905 | T | C | 0.01126 | 0.6684 | 0.02628liver cancer rs10240905 T C -0.0805 0.18852 0.06123 Choleliths rs10240905 T C 0.02309 0.01063 0.00904 Acute pancreatitis rs10240905 T C 0.01973 0.35133 0.02117 Chronic pancreatitis rs10240905 T C 0.0449 0.11604 0.02857 Pancreatic cancer rs10240905 T C -0.0153 0.72799 0.04412 Ulcerative colitis rs10240905 T C 0.01293 0.58089 0.02342 Crohn's disease rs10240905 T C 0.05346 0.1855 0.04037 Irrtable bowel syndrome rs10240905 T C 0.00288 0.869 0.01749 Acute appendicitis rs10240905 T C 0.02804 0.00523 0.01004 Colon cancer rs10240905 T C 0.01228 0.65276 0.0273 Rectal cancer rs10240905 T C -0.0279 0.42127 0.03475 |
| Gastroesophageal reflux | rs10456918 | A | C | 0.01429 | 0.31564 | 0.01424 | Esophageal carcinoma | rs10456918 | A | C | 0.05745 | 0.53774 | 0.09323 | Acute gastritis | rs10456918 | A | C | -0.0195 | 0.66364 | 0.04493 | Chronic gastritis | rs10456918 | A | C | 0.0334 | 0.14222 | 0.02276 | Gastroduodenal ulcer | rs10456918 | A | C | 0.05428 | 0.01996 | 0.02333 | Gastric cancer | rs10456918 | A | C | 0.03203 | 0.58914 | 0.05931 | Fatty liver | rs10456918 | A | C | -0.0269 | 0.57021 | 0.04736 | Hepatic fibrosis | rs10456918 | A | C | 0.29589 | 0.10434 | 0.18218 | Cirrhosis | rs10456918 | A | C | 0.0571 | 0.10437 | 0.03516liver cancer rs10456918 A C -0.1128 0.16987 0.08215 Choleliths rs10456918 A C 0.00067 0.95597 0.01207 Acute pancreatitis rs10456918 A C 0.01602 0.56996 0.0282 Chronic pancreatitis rs10456918 A C -0.0064 0.8661 0.03815 Pancreatic cancer rs10456918 A C 0.03711 0.53152 0.05931 Ulcerative colitis rs10456918 A C 0.04743 0.12749 0.03112 Crohn's disease rs10456918 A C -0.0083 0.87736 0.05377 Irrtable bowel syndrome rs10456918 A C 0.01825 0.43371 0.02331 Acute appendicitis rs10456918 A C -0.0153 0.25326 0.01343 Colon cancer rs10456918 A C -0.038 0.29957 0.0366 Rectal cancer rs10456918 A C 0.01677 0.71845 0.0465 |
| Gastroesophageal reflux | rs10460095 | G | A | 0.00332 | 0.73577 | 0.00985 | Esophageal carcinoma | rs10460095 | G | A | 0.01741 | 0.78431 | 0.0636 | Acute gastritis | rs10460095 | G | A | -0.0361 | 0.24564 | 0.0311 | Chronic gastritis | rs10460095 | G | A | -0.0192 | 0.22237 | 0.01574 | Gastroduodenal ulcer | rs10460095 | G | A | -0.0139 | 0.38964 | 0.0161 | Gastric cancer | rs10460095 | G | A | 0.02723 | 0.50587 | 0.04093 | Fatty liver | rs10460095 | G | A | -0.0218 | 0.50531 | 0.0327 | Hepatic fibrosis | rs10460095 | G | A | -0.0467 | 0.70807 | 0.12473 | Cirrhosis | rs10460095 | G | A | -0.0247 | 0.30539 | 0.02406liver cancer rs10460095 G A -0.0637 0.25708 0.05616 Choleliths rs10460095 G A -0.0153 0.06669 0.00833 Acute pancreatitis rs10460095 G A -0.0237 0.22205 0.01941 Chronic pancreatitis rs10460095 G A -0.0171 0.51218 0.02616 Pancreatic cancer rs10460095 G A 0.03041 0.45248 0.04047 Ulcerative colitis rs10460095 G A 0.00889 0.67952 0.02151 Crohn's disease rs10460095 G A -0.0131 0.72508 0.03713 Irrtable bowel syndrome rs10460095 G A -0.0089 0.58205 0.0161 Acute appendicitis rs10460095 G A -0.0157 0.08944 0.00925 Colon cancer rs10460095 G A -0.0073 0.7738 0.02527 Rectal cancer rs10460095 G A -0.0008 0.98071 0.03204 |
| Gastroesophageal reflux | rs1051474 | T | C | -0.0064 | 0.55015 | 0.01068 | Esophageal carcinoma | rs1051474 | T | C | 0.0725 | 0.29253 | 0.06888 | Acute gastritis | rs1051474 | T | C | -0.0006 | 0.98484 | 0.03366 | Chronic gastritis | rs1051474 | T | C | -0.025 | 0.14212 | 0.01706 | Gastroduodenal ulcer | rs1051474 | T | C | -0.0105 | 0.54855 | 0.01743 | Gastric cancer | rs1051474 | T | C | -0.0677 | 0.12576 | 0.04421 | Fatty liver | rs1051474 | T | C | 0.02881 | 0.41632 | 0.03544 | Hepatic fibrosis | rs1051474 | T | C | 0.09948 | 0.46219 | 0.1353 | Cirrhosis | rs1051474 | T | C | -0.0248 | 0.34233 | 0.02609liver cancer rs1051474 T C -0.0618 0.30862 0.06068 Choleliths rs1051474 T C -0.0217 0.01631 0.00902 Acute pancreatitis rs1051474 T C -0.0037 0.86008 0.02101 Chronic pancreatitis rs1051474 T C -0.0022 0.93895 0.02828 Pancreatic cancer rs1051474 T C -0.0126 0.77382 0.04383 Ulcerative colitis rs1051474 T C 0.00204 0.93023 0.0233 Crohn's disease rs1051474 T C 0.03217 0.42445 0.04027 Irrtable bowel syndrome rs1051474 T C -0.0027 0.87888 0.01745 Acute appendicitis rs1051474 T C -0.0052 0.60604 0.01003 Colon cancer rs1051474 T C 7.1E-05 0.99792 0.02736 Rectal cancer rs1051474 T C -0.0012 0.97258 0.03465 |
| Gastroesophageal reflux | rs10760023 | C | G | -0.0116 | 0.27773 | 0.01072 | Esophageal carcinoma | rs10760023 | C | G | -0.1002 | 0.14997 | 0.0696 | Acute gastritis | rs10760023 | C | G | 0.05697 | 0.09224 | 0.03384 | Chronic gastritis | rs10760023 | C | G | -0.0031 | 0.8585 | 0.01711 | Gastroduodenal ulcer | rs10760023 | C | G | -0.007 | 0.68921 | 0.0175 | Gastric cancer | rs10760023 | C | G | -0.0488 | 0.27313 | 0.04457 | Fatty liver | rs10760023 | C | G | 0.0251 | 0.4805 | 0.03558 | Hepatic fibrosis | rs10760023 | C | G | 0.11727 | 0.38883 | 0.13609 | Cirrhosis | rs10760023 | C | G | 0.0035 | 0.8937 | 0.02618liver cancer rs10760023 C G -0.1529 0.00955 0.05899 Choleliths rs10760023 C G -0.0014 0.88083 0.00906 Acute pancreatitis rs10760023 C G 0.00534 0.80043 0.02111 Chronic pancreatitis rs10760023 C G -0.0167 0.55678 0.02847 Pancreatic cancer rs10760023 C G -0.0453 0.3039 0.04411 Ulcerative colitis rs10760023 C G 0.02951 0.20745 0.02341 Crohn's disease rs10760023 C G -0.0187 0.64375 0.04035 Irrtable bowel syndrome rs10760023 C G 0.00568 0.74585 0.01752 Acute appendicitis rs10760023 C G 0.00156 0.87682 0.01007 Colon cancer rs10760023 C G -0.0077 0.78077 0.02753 Rectal cancer rs10760023 C G 0.00827 0.81271 0.0349 |
| Gastroesophageal reflux | rs10765775 | A | G | -0.0106 | 0.33637 | 0.01105 | Esophageal carcinoma | rs10765775 | A | G | -0.1861 | 0.00984 | 0.07209 | Acute gastritis | rs10765775 | A | G | 0.00286 | 0.93471 | 0.0349 | Chronic gastritis | rs10765775 | A | G | -0.0136 | 0.44146 | 0.01762 | Gastroduodenal ulcer | rs10765775 | A | G | -0.0526 | 0.00387 | 0.01821 | Gastric cancer | rs10765775 | A | G | 0.00658 | 0.88616 | 0.04598 | Fatty liver | rs10765775 | A | G | 0.02356 | 0.52121 | 0.03673 | Hepatic fibrosis | rs10765775 | A | G | -0.0183 | 0.8967 | 0.14075 | Cirrhosis | rs10765775 | A | G | -0.022 | 0.41838 | 0.02715liver cancer rs10765775 A G -0.0606 0.33975 0.0635 Choleliths rs10765775 A G -0.0042 0.65013 0.00936 Acute pancreatitis rs10765775 A G -0.0107 0.62544 0.02185 Chronic pancreatitis rs10765775 A G -0.0269 0.36138 0.0295 Pancreatic cancer rs10765775 A G -0.0912 0.04603 0.0457 Ulcerative colitis rs10765775 A G 0.0203 0.40209 0.02422 Crohn's disease rs10765775 A G 0.01377 0.74152 0.04176 Irrtable bowel syndrome rs10765775 A G 0.01747 0.33286 0.01804 Acute appendicitis rs10765775 A G -0.0058 0.5779 0.01041 Colon cancer rs10765775 A G 0.01398 0.62293 0.02843 Rectal cancer rs10765775 A G 0.02536 0.4821 0.03608 |
| Gastroesophageal reflux | rs10772644 | G | C | -0.0295 | 0.02573 | 0.01322 | Esophageal carcinoma | rs10772644 | G | C | 0.06055 | 0.48091 | 0.0859 | Acute gastritis | rs10772644 | G | C | -0.0116 | 0.78125 | 0.04178 | Chronic gastritis | rs10772644 | G | C | -0.0254 | 0.22986 | 0.02113 | Gastroduodenal ulcer | rs10772644 | G | C | 0.01127 | 0.60169 | 0.0216 | Gastric cancer | rs10772644 | G | C | 0.08988 | 0.10285 | 0.0551 | Fatty liver | rs10772644 | G | C | -0.0272 | 0.53441 | 0.0438 | Hepatic fibrosis | rs10772644 | G | C | -0.1632 | 0.32621 | 0.16626 | Cirrhosis | rs10772644 | G | C | -0.0108 | 0.73825 | 0.03224liver cancer rs10772644 G C 0.03355 0.65557 0.07522 Choleliths rs10772644 G C 0.01432 0.20068 0.01119 Acute pancreatitis rs10772644 G C 0.02546 0.32775 0.02601 Chronic pancreatitis rs10772644 G C 0.06417 0.06648 0.03497 Pancreatic cancer rs10772644 G C 0.05577 0.30537 0.05441 Ulcerative colitis rs10772644 G C 0.03764 0.1913 0.02881 Crohn's disease rs10772644 G C 0.02547 0.60827 0.0497 Irrtable bowel syndrome rs10772644 G C -0.0054 0.80184 0.02158 Acute appendicitis rs10772644 G C -0.0113 0.3598 0.01239 Colon cancer rs10772644 G C -0.0423 0.21521 0.03412 Rectal cancer rs10772644 G C -0.0268 0.5362 0.0433 |
| Gastroesophageal reflux | rs10773002 | T | A | 0.00594 | 0.60482 | 0.01147 | Esophageal carcinoma | rs10773002 | T | A | -0.0584 | 0.43364 | 0.0746 | Acute gastritis | rs10773002 | T | A | 0.02271 | 0.53089 | 0.03625 | Chronic gastritis | rs10773002 | T | A | -0.0141 | 0.44371 | 0.01837 | Gastroduodenal ulcer | rs10773002 | T | A | -0.0154 | 0.41198 | 0.01879 | Gastric cancer | rs10773002 | T | A | 0.09796 | 0.04114 | 0.04797 | Fatty liver | rs10773002 | T | A | 0.00776 | 0.83859 | 0.03809 | Hepatic fibrosis | rs10773002 | T | A | 0.02539 | 0.86136 | 0.14536 | Cirrhosis | rs10773002 | T | A | 0.02294 | 0.41446 | 0.02811liver cancer rs10773002 T A -0.0245 0.70964 0.06586 Choleliths rs10773002 T A 0.01898 0.05072 0.00971 Acute pancreatitis rs10773002 T A 0.03309 0.14346 0.02262 Chronic pancreatitis rs10773002 T A 0.04656 0.12698 0.03051 Pancreatic cancer rs10773002 T A 0.06656 0.16112 0.04749 Ulcerative colitis rs10773002 T A 0.04632 0.06444 0.02505 Crohn's disease rs10773002 T A 0.0275 0.52398 0.04316 Irrtable bowel syndrome rs10773002 T A 0.03482 0.06284 0.01872 Acute appendicitis rs10773002 T A -0.0172 0.11009 0.01078 Colon cancer rs10773002 T A -0.0064 0.82926 0.02951 Rectal cancer rs10773002 T A 0.10458 0.0059 0.03798 |
| Gastroesophageal reflux | rs10856785 | T | C | 0.02732 | 0.01096 | 0.01074 | Esophageal carcinoma | rs10856785 | T | C | -0.0788 | 0.25678 | 0.0695 | Acute gastritis | rs10856785 | T | C | 0.03312 | 0.32978 | 0.03398 | Chronic gastritis | rs10856785 | T | C | 0.01856 | 0.27961 | 0.01716 | Gastroduodenal ulcer | rs10856785 | T | C | -0.0058 | 0.74292 | 0.01758 | Gastric cancer | rs10856785 | T | C | -0.0203 | 0.65058 | 0.04475 | Fatty liver | rs10856785 | T | C | -0.0122 | 0.73265 | 0.03569 | Hepatic fibrosis | rs10856785 | T | C | -0.14 | 0.30562 | 0.13667 | Cirrhosis | rs10856785 | T | C | -0.0203 | 0.44084 | 0.02629liver cancer rs10856785 T C -0.0658 0.28502 0.06152 Choleliths rs10856785 T C 0.00781 0.38916 0.00907 Acute pancreatitis rs10856785 T C -0.0231 0.27493 0.02118 Chronic pancreatitis rs10856785 T C 0.03047 0.28556 0.02853 Pancreatic cancer rs10856785 T C -0.0086 0.84548 0.04436 Ulcerative colitis rs10856785 T C -0.0252 0.28379 0.02347 Crohn's disease rs10856785 T C -0.0515 0.20323 0.04051 Irrtable bowel syndrome rs10856785 T C 0.01146 0.51409 0.01757 Acute appendicitis rs10856785 T C 0.00039 0.9691 0.01011 Colon cancer rs10856785 T C -0.0243 0.37658 0.02751 Rectal cancer rs10856785 T C -0.0569 0.10299 0.03492 |
| Gastroesophageal reflux | rs10862376 | T | A | 0.00317 | 0.80484 | 0.01284 | Esophageal carcinoma | rs10862376 | T | A | 0.00681 | 0.93476 | 0.08323 | Acute gastritis | rs10862376 | T | A | 0.04833 | 0.23168 | 0.04041 | Chronic gastritis | rs10862376 | T | A | 0.00767 | 0.70871 | 0.02052 | Gastroduodenal ulcer | rs10862376 | T | A | 0.02447 | 0.2436 | 0.02099 | Gastric cancer | rs10862376 | T | A | 0.01311 | 0.80597 | 0.05339 | Fatty liver | rs10862376 | T | A | 0.02648 | 0.53383 | 0.04256 | Hepatic fibrosis | rs10862376 | T | A | 0.26321 | 0.10537 | 0.16254 | Cirrhosis | rs10862376 | T | A | 0.10744 | 0.00076 | 0.03192liver cancer rs10862376 T A 0.11732 0.10922 0.07325 Choleliths rs10862376 T A -0.0071 0.5152 0.01084 Acute pancreatitis rs10862376 T A -0.0047 0.85252 0.02526 Chronic pancreatitis rs10862376 T A 0.01531 0.65334 0.03408 Pancreatic cancer rs10862376 T A 0.00492 0.92578 0.05283 Ulcerative colitis rs10862376 T A 0.00631 0.82124 0.02794 Crohn's disease rs10862376 T A 0.03217 0.50435 0.04819 Irrtable bowel syndrome rs10862376 T A 0.02081 0.32138 0.02098 Acute appendicitis rs10862376 T A -0.0048 0.69233 0.01203 Colon cancer rs10862376 T A -0.0321 0.32884 0.03284 Rectal cancer rs10862376 T A -0.0514 0.21759 0.04172 |
| Gastroesophageal reflux | rs10875121 | G | C | ####### | 0.9933 | 0.0116 | Esophageal carcinoma | rs10875121 | G | C | -0.0962 | 0.20092 | 0.07524 | Acute gastritis | rs10875121 | G | C | 0.02475 | 0.5002 | 0.03672 | Chronic gastritis | rs10875121 | G | C | 0.01176 | 0.5266 | 0.01858 | Gastroduodenal ulcer | rs10875121 | G | C | 0.00059 | 0.97505 | 0.01902 | Gastric cancer | rs10875121 | G | C | 0.09061 | 0.06066 | 0.0483 | Fatty liver | rs10875121 | G | C | 0.01712 | 0.65667 | 0.03852 | Hepatic fibrosis | rs10875121 | G | C | -0.0901 | 0.53911 | 0.14674 | Cirrhosis | rs10875121 | G | C | 0.02662 | 0.34781 | 0.02836liver cancer rs10875121 G C -0.0967 0.14488 0.06633 Choleliths rs10875121 G C -0.0041 0.67326 0.0098 Acute pancreatitis rs10875121 G C 0.03866 0.09091 0.02287 Chronic pancreatitis rs10875121 G C 0.02174 0.48023 0.0308 Pancreatic cancer rs10875121 G C 0.02608 0.58488 0.04774 Ulcerative colitis rs10875121 G C 0.01766 0.48488 0.02528 Crohn's disease rs10875121 G C 0.05195 0.23387 0.04364 Irrtable bowel syndrome rs10875121 G C -0.0104 0.58382 0.01898 Acute appendicitis rs10875121 G C 0.01008 0.35471 0.01089 Colon cancer rs10875121 G C 0.00355 0.90485 0.02969 Rectal cancer rs10875121 G C 0.00725 0.8477 0.03773 |
| Gastroesophageal reflux | rs10887801 | G | T | 0.01013 | 0.30263 | 0.00982 | Esophageal carcinoma | rs10887801 | G | T | -0.0192 | 0.76358 | 0.06371 | Acute gastritis | rs10887801 | G | T | -0.018 | 0.56231 | 0.03102 | Chronic gastritis | rs10887801 | G | T | -0.002 | 0.89757 | 0.0157 | Gastroduodenal ulcer | rs10887801 | G | T | 0.01958 | 0.22328 | 0.01608 | Gastric cancer | rs10887801 | G | T | 0.02311 | 0.57171 | 0.04086 | Fatty liver | rs10887801 | G | T | -0.0613 | 0.06016 | 0.03262 | Hepatic fibrosis | rs10887801 | G | T | 0.01915 | 0.87764 | 0.1244 | Cirrhosis | rs10887801 | G | T | -0.0008 | 0.97433 | 0.02402liver cancer rs10887801 G T 0.0638 0.25591 0.05616 Choleliths rs10887801 G T 0.01297 0.11845 0.00831 Acute pancreatitis rs10887801 G T -0.0041 0.8307 0.01937 Chronic pancreatitis rs10887801 G T 0.01553 0.55219 0.02612 Pancreatic cancer rs10887801 G T 0.00457 0.91011 0.04044 Ulcerative colitis rs10887801 G T 0.01188 0.57924 0.02143 Crohn's disease rs10887801 G T 0.00767 0.83558 0.03698 Irrtable bowel syndrome rs10887801 G T 0.01185 0.46025 0.01605 Acute appendicitis rs10887801 G T -0.0032 0.73133 0.00922 Colon cancer rs10887801 G T -0.0256 0.30936 0.02522 Rectal cancer rs10887801 G T -0.0259 0.41897 0.03203 |
| Gastroesophageal reflux | rs10940921 | G | T | 0.02027 | 0.03966 | 0.00985 | Esophageal carcinoma | rs10940921 | G | T | 0.02078 | 0.7459 | 0.06414 | Acute gastritis | rs10940921 | G | T | 0.03528 | 0.25706 | 0.03113 | Chronic gastritis | rs10940921 | G | T | 0.00859 | 0.58512 | 0.01574 | Gastroduodenal ulcer | rs10940921 | G | T | 0.0052 | 0.74694 | 0.01612 | Gastric cancer | rs10940921 | G | T | -0.0094 | 0.81864 | 0.04108 | Fatty liver | rs10940921 | G | T | 0.01735 | 0.59584 | 0.03271 | Hepatic fibrosis | rs10940921 | G | T | 0.23198 | 0.06286 | 0.12471 | Cirrhosis | rs10940921 | G | T | 0.03713 | 0.12359 | 0.02411liver cancer rs10940921 G T 0.05758 0.30658 0.05631 Choleliths rs10940921 G T 0.01259 0.13086 0.00834 Acute pancreatitis rs10940921 G T -0.0051 0.79313 0.01943 Chronic pancreatitis rs10940921 G T 0.03286 0.20983 0.0262 Pancreatic cancer rs10940921 G T -0.0569 0.16224 0.04068 Ulcerative colitis rs10940921 G T -0.0091 0.67233 0.02151 Crohn's disease rs10940921 G T -0.0113 0.75993 0.03706 Irrtable bowel syndrome rs10940921 G T 0.01805 0.26216 0.0161 Acute appendicitis rs10940921 G T 0.00569 0.5384 0.00925 Colon cancer rs10940921 G T -0.0145 0.56699 0.02532 Rectal cancer rs10940921 G T -0.0195 0.54347 0.03212 |
| Gastroesophageal reflux | rs10963297 | G | C | -0.0138 | 0.21437 | 0.01113 | Esophageal carcinoma | rs10963297 | G | C | -0.0604 | 0.40379 | 0.0723 | Acute gastritis | rs10963297 | G | C | 0.00797 | 0.82052 | 0.03515 | Chronic gastritis | rs10963297 | G | C | -0.0319 | 0.07237 | 0.01778 | Gastroduodenal ulcer | rs10963297 | G | C | -0.0108 | 0.55439 | 0.0182 | Gastric cancer | rs10963297 | G | C | 0.02864 | 0.5376 | 0.04647 | Fatty liver | rs10963297 | G | C | -0.015 | 0.68367 | 0.03693 | Hepatic fibrosis | rs10963297 | G | C | -0.1515 | 0.28119 | 0.14057 | Cirrhosis | rs10963297 | G | C | -0.0176 | 0.51639 | 0.0272liver cancer rs10963297 G C 0.01151 0.8569 0.06382 Choleliths rs10963297 G C -0.0141 0.13269 0.0094 Acute pancreatitis rs10963297 G C -0.0505 0.02111 0.02191 Chronic pancreatitis rs10963297 G C -0.0213 0.47045 0.02955 Pancreatic cancer rs10963297 G C 0.11794 0.0103 0.04597 Ulcerative colitis rs10963297 G C 0.05781 0.01737 0.0243 Crohn's disease rs10963297 G C -0.0658 0.11639 0.04189 Irrtable bowel syndrome rs10963297 G C -0.0036 0.84291 0.01817 Acute appendicitis rs10963297 G C 0.0079 0.44947 0.01045 Colon cancer rs10963297 G C 0.03175 0.26754 0.02864 Rectal cancer rs10963297 G C 0.03627 0.31922 0.03641 |
| Gastroesophageal reflux | rs10994777 | G | A | 0.02423 | 0.11428 | 0.01534 | Esophageal carcinoma | rs10994777 | G | A | 0.0185 | 0.85099 | 0.0985 | Acute gastritis | rs10994777 | G | A | -0.0323 | 0.5038 | 0.04829 | Chronic gastritis | rs10994777 | G | A | 0.00277 | 0.90974 | 0.02445 | Gastroduodenal ulcer | rs10994777 | G | A | 0.01074 | 0.66827 | 0.02505 | Gastric cancer | rs10994777 | G | A | -0.022 | 0.73079 | 0.06388 | Fatty liver | rs10994777 | G | A | -0.0193 | 0.70498 | 0.05106 | Hepatic fibrosis | rs10994777 | G | A | -0.3606 | 0.06676 | 0.19667 | Cirrhosis | rs10994777 | G | A | -0.0524 | 0.16476 | 0.03769liver cancer rs10994777 G A -0.0036 0.96748 0.088 Choleliths rs10994777 G A 0.01517 0.24193 0.01296 Acute pancreatitis rs10994777 G A -0.02 0.51045 0.03033 Chronic pancreatitis rs10994777 G A -0.0665 0.10424 0.04093 Pancreatic cancer rs10994777 G A 0.00898 0.88712 0.06323 Ulcerative colitis rs10994777 G A -0.0068 0.83941 0.0336 Crohn's disease rs10994777 G A -0.0807 0.16511 0.05812 Irrtable bowel syndrome rs10994777 G A -0.0051 0.8382 0.02508 Acute appendicitis rs10994777 G A 0.01598 0.26988 0.01448 Colon cancer rs10994777 G A -0.0362 0.35574 0.03918 Rectal cancer rs10994777 G A 0.00678 0.89167 0.04982 |
| Gastroesophageal reflux | rs11023749 | A | G | -0.0112 | 0.27465 | 0.01027 | Esophageal carcinoma | rs11023749 | A | G | -0.0636 | 0.3384 | 0.06643 | Acute gastritis | rs11023749 | A | G | 0.02751 | 0.39614 | 0.03242 | Chronic gastritis | rs11023749 | A | G | -0.0022 | 0.89291 | 0.0164 | Gastroduodenal ulcer | rs11023749 | A | G | 0.02143 | 0.20174 | 0.01679 | Gastric cancer | rs11023749 | A | G | -0.0815 | 0.05629 | 0.04271 | Fatty liver | rs11023749 | A | G | 0.03084 | 0.36519 | 0.03406 | Hepatic fibrosis | rs11023749 | A | G | -0.0998 | 0.4433 | 0.13022 | Cirrhosis | rs11023749 | A | G | -0.0235 | 0.34917 | 0.02513liver cancer rs11023749 A G -0.0615 0.29299 0.05844 Choleliths rs11023749 A G 0.02397 0.00581 0.00869 Acute pancreatitis rs11023749 A G 0.01621 0.42332 0.02024 Chronic pancreatitis rs11023749 A G 0.02662 0.32977 0.02731 Pancreatic cancer rs11023749 A G -0.0402 0.34093 0.04221 Ulcerative colitis rs11023749 A G 0.00845 0.70639 0.02244 Crohn's disease rs11023749 A G -0.0146 0.70613 0.03868 Irrtable bowel syndrome rs11023749 A G 0.02288 0.17241 0.01677 Acute appendicitis rs11023749 A G 0.00132 0.89114 0.00964 Colon cancer rs11023749 A G 0.04927 0.06154 0.02635 Rectal cancer rs11023749 A G 0.04234 0.2059 0.03347 |
| Gastroesophageal reflux | rs1106090 | A | G | 0.04311 | ####### | 0.00991 | Esophageal carcinoma | rs1106090 | A | G | -0.0871 | 0.17351 | 0.06399 | Acute gastritis | rs1106090 | A | G | 0.01624 | 0.60328 | 0.03124 | Chronic gastritis | rs1106090 | A | G | 0.01739 | 0.27164 | 0.01582 | Gastroduodenal ulcer | rs1106090 | A | G | -0.0171 | 0.29176 | 0.01619 | Gastric cancer | rs1106090 | A | G | -0.0254 | 0.53752 | 0.04122 | Fatty liver | rs1106090 | A | G | -0.0154 | 0.63863 | 0.03284 | Hepatic fibrosis | rs1106090 | A | G | -0.0716 | 0.56763 | 0.12526 | Cirrhosis | rs1106090 | A | G | -0.0059 | 0.8083 | 0.02419liver cancer rs1106090 A G 0.00774 0.89082 0.05641 Choleliths rs1106090 A G -0.0177 0.03412 0.00836 Acute pancreatitis rs1106090 A G 0.02017 0.30107 0.0195 Chronic pancreatitis rs1106090 A G -0.01 0.70443 0.0263 Pancreatic cancer rs1106090 A G 0.01723 0.67207 0.04071 Ulcerative colitis rs1106090 A G -0.0061 0.77721 0.0216 Crohn's disease rs1106090 A G 0.03651 0.3266 0.03722 Irrtable bowel syndrome rs1106090 A G 0.03925 0.01523 0.01618 Acute appendicitis rs1106090 A G -0.0043 0.64344 0.00929 Colon cancer rs1106090 A G -0.003 0.90689 0.0254 Rectal cancer rs1106090 A G 0.01018 0.75235 0.03225 |
| Gastroesophageal reflux | rs11081529 | T | C | -0.0135 | 0.19862 | 0.0105 | Esophageal carcinoma | rs11081529 | T | C | 0.0782 | 0.25082 | 0.0681 | Acute gastritis | rs11081529 | T | C | 0.00339 | 0.91874 | 0.03318 | Chronic gastritis | rs11081529 | T | C | -0.0019 | 0.91098 | 0.01677 | Gastroduodenal ulcer | rs11081529 | T | C | 0.00024 | 0.98878 | 0.01718 | Gastric cancer | rs11081529 | T | C | 0.0017 | 0.96898 | 0.0438 | Fatty liver | rs11081529 | T | C | 0.02375 | 0.49499 | 0.03481 | Hepatic fibrosis | rs11081529 | T | C | 0.04167 | 0.75351 | 0.1327 | Cirrhosis | rs11081529 | T | C | 0.01733 | 0.49973 | 0.02567liver cancer rs11081529 T C 0.13569 0.02388 0.06006 Choleliths rs11081529 T C -0.0026 0.76913 0.00887 Acute pancreatitis rs11081529 T C 0.02884 0.16323 0.02068 Chronic pancreatitis rs11081529 T C -0.0053 0.85018 0.02786 Pancreatic cancer rs11081529 T C 0.00029 0.9947 0.04332 Ulcerative colitis rs11081529 T C -0.0166 0.46768 0.02289 Crohn's disease rs11081529 T C -0.0326 0.40813 0.03946 Irrtable bowel syndrome rs11081529 T C -0.0119 0.48644 0.01714 Acute appendicitis rs11081529 T C -0.0033 0.7357 0.00985 Colon cancer rs11081529 T C -0.014 0.6035 0.02699 Rectal cancer rs11081529 T C -0.008 0.81575 0.03422 |
| Gastroesophageal reflux | rs11123818 | G | A | 0.01799 | 0.07308 | 0.01004 | Esophageal carcinoma | rs11123818 | G | A | 0.10423 | 0.1085 | 0.06494 | Acute gastritis | rs11123818 | G | A | -0.0124 | 0.69462 | 0.03167 | Chronic gastritis | rs11123818 | G | A | 0.00172 | 0.91482 | 0.01605 | Gastroduodenal ulcer | rs11123818 | G | A | -0.0032 | 0.84568 | 0.01641 | Gastric cancer | rs11123818 | G | A | -0.0337 | 0.42031 | 0.04182 | Fatty liver | rs11123818 | G | A | -0.0053 | 0.87449 | 0.03334 | Hepatic fibrosis | rs11123818 | G | A | -0.1289 | 0.31027 | 0.12703 | Cirrhosis | rs11123818 | G | A | -0.0011 | 0.96301 | 0.02455liver cancer rs11123818 G A -0.0374 0.51444 0.05739 Choleliths rs11123818 G A 0.01741 0.04005 0.00848 Acute pancreatitis rs11123818 G A 0.03395 0.08611 0.01978 Chronic pancreatitis rs11123818 G A 0.05061 0.05778 0.02667 Pancreatic cancer rs11123818 G A 0.08168 0.04856 0.04141 Ulcerative colitis rs11123818 G A -0.014 0.52423 0.02193 Crohn's disease rs11123818 G A -0.0159 0.67348 0.03781 Irrtable bowel syndrome rs11123818 G A 0.02209 0.17816 0.01641 Acute appendicitis rs11123818 G A 0.00584 0.53566 0.00942 Colon cancer rs11123818 G A -0.023 0.3735 0.02581 Rectal cancer rs11123818 G A -0.0829 0.01136 0.03277 |
| Gastroesophageal reflux | rs111821073 | C | T | 0.00988 | 0.4954 | 0.0145 | Esophageal carcinoma | rs111821073 | C | T | 0.14885 | 0.11301 | 0.09392 | Acute gastritis | rs111821073 | C | T | 0.01517 | 0.74021 | 0.04574 | Chronic gastritis | rs111821073 | C | T | -0.0142 | 0.5387 | 0.0231 | Gastroduodenal ulcer | rs111821073 | C | T | 0.02442 | 0.30436 | 0.02377 | Gastric cancer | rs111821073 | C | T | 0.08143 | 0.18115 | 0.06089 | Fatty liver | rs111821073 | C | T | -0.0387 | 0.4227 | 0.04823 | Hepatic fibrosis | rs111821073 | C | T | -0.0242 | 0.89521 | 0.1837 | Cirrhosis | rs111821073 | C | T | -0.0307 | 0.38714 | 0.03551liver cancer rs111821073 C T 0.0115 0.88994 0.08312 Choleliths rs111821073 C T 0.02104 0.087 0.01229 Acute pancreatitis rs111821073 C T 0.03103 0.27929 0.02868 Chronic pancreatitis rs111821073 C T -0.002 0.95942 0.03871 Pancreatic cancer rs111821073 C T -0.0536 0.37254 0.06008 Ulcerative colitis rs111821073 C T 0.04461 0.15983 0.03173 Crohn's disease rs111821073 C T -0.0558 0.30783 0.05471 Irrtable bowel syndrome rs111821073 C T 0.00279 0.90641 0.02375 Acute appendicitis rs111821073 C T 0.00769 0.57396 0.01368 Colon cancer rs111821073 C T -0.0929 0.01269 0.03729 Rectal cancer rs111821073 C T -0.0332 0.48161 0.04724 |
| Gastroesophageal reflux | rs11222609 | G | A | 0.01116 | 0.31952 | 0.01122 | Esophageal carcinoma | rs11222609 | G | A | -0.0339 | 0.638 | 0.07215 | Acute gastritis | rs11222609 | G | A | 0.04766 | 0.17895 | 0.03546 | Chronic gastritis | rs11222609 | G | A | 0.02522 | 0.15892 | 0.0179 | Gastroduodenal ulcer | rs11222609 | G | A | 0.03064 | 0.09513 | 0.01836 | Gastric cancer | rs11222609 | G | A | 0.01227 | 0.79203 | 0.04652 | Fatty liver | rs11222609 | G | A | 0.039 | 0.29546 | 0.03728 | Hepatic fibrosis | rs11222609 | G | A | 0.00081 | 0.99546 | 0.14283 | Cirrhosis | rs11222609 | G | A | -0.0027 | 0.92046 | 0.02746liver cancer rs11222609 G A 0.12573 0.04836 0.06369 Choleliths rs11222609 G A 0.01953 0.03934 0.00948 Acute pancreatitis rs11222609 G A 0.03123 0.15828 0.02213 Chronic pancreatitis rs11222609 G A 0.0041 0.89091 0.02989 Pancreatic cancer rs11222609 G A 0.00161 0.97215 0.04604 Ulcerative colitis rs11222609 G A -0.0396 0.10729 0.02457 Crohn's disease rs11222609 G A -0.029 0.49324 0.04229 Irrtable bowel syndrome rs11222609 G A 0.01678 0.36059 0.01835 Acute appendicitis rs11222609 G A 0.01061 0.31519 0.01056 Colon cancer rs11222609 G A 0.01709 0.55114 0.02867 Rectal cancer rs11222609 G A 0.03225 0.37522 0.03637 |
| Gastroesophageal reflux | rs112687095 | G | A | 0.00082 | 0.96528 | 0.01894 | Esophageal carcinoma | rs112687095 | G | A | -0.0978 | 0.4221 | 0.12189 | Acute gastritis | rs112687095 | G | A | -0.0493 | 0.41342 | 0.06023 | Chronic gastritis | rs112687095 | G | A | -0.0311 | 0.30094 | 0.0301 | Gastroduodenal ulcer | rs112687095 | G | A | 0.04782 | 0.12301 | 0.03101 | Gastric cancer | rs112687095 | G | A | 0.0554 | 0.47988 | 0.07842 | Fatty liver | rs112687095 | G | A | 0.0325 | 0.60547 | 0.06291 | Hepatic fibrosis | rs112687095 | G | A | 0.05874 | 0.8113 | 0.24602 | Cirrhosis | rs112687095 | G | A | 0.03752 | 0.42214 | 0.04675liver cancer rs112687095 G A 0.10461 0.32955 0.10729 Choleliths rs112687095 G A -0.0198 0.21886 0.01607 Acute pancreatitis rs112687095 G A -0.0072 0.84844 0.03761 Chronic pancreatitis rs112687095 G A 0.0083 0.8705 0.05093 Pancreatic cancer rs112687095 G A -0.0856 0.27276 0.07807 Ulcerative colitis rs112687095 G A -0.0419 0.31534 0.04174 Crohn's disease rs112687095 G A 0.12155 0.08965 0.07162 Irrtable bowel syndrome rs112687095 G A -0.0006 0.98541 0.03106 Acute appendicitis rs112687095 G A -0.0061 0.73416 0.01798 Colon cancer rs112687095 G A 0.06974 0.14864 0.04828 Rectal cancer rs112687095 G A 0.06645 0.2753 0.06091 |
| Gastroesophageal reflux | rs112806496 | G | C | -0.0236 | 0.27589 | 0.02165 | Esophageal carcinoma | rs112806496 | G | C | -0.0056 | 0.96802 | 0.13923 | Acute gastritis | rs112806496 | G | C | 0.01618 | 0.81316 | 0.06846 | Chronic gastritis | rs112806496 | G | C | -0.0308 | 0.37269 | 0.0345 | Gastroduodenal ulcer | rs112806496 | G | C | -0.0062 | 0.86142 | 0.03533 | Gastric cancer | rs112806496 | G | C | -0.0328 | 0.71017 | 0.08834 | Fatty liver | rs112806496 | G | C | -0.0096 | 0.89346 | 0.07187 | Hepatic fibrosis | rs112806496 | G | C | -0.1097 | 0.69535 | 0.28023 | Cirrhosis | rs112806496 | G | C | -0.0809 | 0.12961 | 0.05337liver cancer rs112806496 G C -0.118 0.33323 0.122 Choleliths rs112806496 G C -0.0018 0.91985 0.01824 Acute pancreatitis rs112806496 G C -0.0042 0.92113 0.04279 Chronic pancreatitis rs112806496 G C 0.02388 0.68033 0.05797 Pancreatic cancer rs112806496 G C -0.1214 0.17015 0.08853 Ulcerative colitis rs112806496 G C -0.051 0.28373 0.04757 Crohn's disease rs112806496 G C -0.0354 0.66537 0.08193 Irrtable bowel syndrome rs112806496 G C -0.0551 0.12086 0.03554 Acute appendicitis rs112806496 G C 3.9E-05 0.99847 0.02044 Colon cancer rs112806496 G C -0.057 0.29887 0.05482 Rectal cancer rs112806496 G C -0.0012 0.9857 0.06965 |
| Gastroesophageal reflux | rs113182709 | G | A | -0.0135 | 0.75803 | 0.04395 | Esophageal carcinoma | rs113182709 | G | A | -0.202 | 0.4814 | 0.28698 | Acute gastritis | rs113182709 | G | A | 0.02869 | 0.83759 | 0.13994 | Chronic gastritis | rs113182709 | G | A | 0.02461 | 0.72579 | 0.07015 | Gastroduodenal ulcer | rs113182709 | G | A | -0.0452 | 0.5342 | 0.07269 | Gastric cancer | rs113182709 | G | A | -0.1126 | 0.54315 | 0.18518 | Fatty liver | rs113182709 | G | A | -0.1511 | 0.31005 | 0.14882 | Hepatic fibrosis | rs113182709 | G | A | 0.39755 | 0.48392 | 0.56792 | Cirrhosis | rs113182709 | G | A | 0.13731 | 0.21004 | 0.10955liver cancer rs113182709 G A 0.41916 0.10384 0.2577 Choleliths rs113182709 G A -0.009 0.81128 0.03785 Acute pancreatitis rs113182709 G A 0.00061 0.99449 0.08836 Chronic pancreatitis rs113182709 G A 0.16597 0.16747 0.12023 Pancreatic cancer rs113182709 G A 0.26621 0.15156 0.18564 Ulcerative colitis rs113182709 G A 0.11008 0.258 0.09732 Crohn's disease rs113182709 G A -0.3028 0.07046 0.16738 Irrtable bowel syndrome rs113182709 G A -0.0456 0.53174 0.07299 Acute appendicitis rs113182709 G A -0.0714 0.09132 0.04229 Colon cancer rs113182709 G A -0.0562 0.62121 0.11363 Rectal cancer rs113182709 G A -0.0714 0.61933 0.14374 |
| Gastroesophageal reflux | rs113520408 | A | G | 0.01332 | 0.26029 | 0.01184 | Esophageal carcinoma | rs113520408 | A | G | 0.07961 | 0.29781 | 0.07647 | Acute gastritis | rs113520408 | A | G | 0.06373 | 0.08869 | 0.03744 | Chronic gastritis | rs113520408 | A | G | -0.021 | 0.26557 | 0.0189 | Gastroduodenal ulcer | rs113520408 | A | G | 0.01784 | 0.35589 | 0.01933 | Gastric cancer | rs113520408 | A | G | 0.00614 | 0.9006 | 0.04912 | Fatty liver | rs113520408 | A | G | 0.01375 | 0.72684 | 0.03938 | Hepatic fibrosis | rs113520408 | A | G | -0.2164 | 0.15083 | 0.15062 | Cirrhosis | rs113520408 | A | G | 0.03876 | 0.18108 | 0.02898liver cancer rs113520408 A G 0.01483 0.82605 0.06747 Choleliths rs113520408 A G 0.00191 0.84868 0.01001 Acute pancreatitis rs113520408 A G 0.03126 0.18072 0.02335 Chronic pancreatitis rs113520408 A G 0.04295 0.17333 0.03154 Pancreatic cancer rs113520408 A G 0.0351 0.47216 0.04881 Ulcerative colitis rs113520408 A G -0.0036 0.88999 0.02589 Crohn's disease rs113520408 A G -0.0471 0.29061 0.04461 Irrtable bowel syndrome rs113520408 A G -0.0142 0.46304 0.01935 Acute appendicitis rs113520408 A G -0.0085 0.44311 0.01113 Colon cancer rs113520408 A G -0.0008 0.97784 0.03023 Rectal cancer rs113520408 A G 0.02041 0.59532 0.03842 |
| Gastroesophageal reflux | rs113615161 | T | C | 0.01346 | 0.38137 | 0.01538 | Esophageal carcinoma | rs113615161 | T | C | -0.0978 | 0.32485 | 0.09934 | Acute gastritis | rs113615161 | T | C | -0.012 | 0.80413 | 0.04853 | Chronic gastritis | rs113615161 | T | C | 0.06173 | 0.01181 | 0.02452 | Gastroduodenal ulcer | rs113615161 | T | C | -0.0069 | 0.78432 | 0.02518 | Gastric cancer | rs113615161 | T | C | -0.0743 | 0.24226 | 0.06352 | Fatty liver | rs113615161 | T | C | -0.0849 | 0.09774 | 0.05125 | Hepatic fibrosis | rs113615161 | T | C | -0.0812 | 0.68061 | 0.19736 | Cirrhosis | rs113615161 | T | C | -0.0027 | 0.94352 | 0.03791liver cancer rs113615161 T C -0.0733 0.40481 0.08795 Choleliths rs113615161 T C 0.01551 0.23242 0.01299 Acute pancreatitis rs113615161 T C 0.01602 0.59986 0.03054 Chronic pancreatitis rs113615161 T C 0.02849 0.49085 0.04135 Pancreatic cancer rs113615161 T C 0.00277 0.96529 0.0636 Ulcerative colitis rs113615161 T C -0.021 0.53517 0.03379 Crohn's disease rs113615161 T C -0.0859 0.13937 0.05811 Irrtable bowel syndrome rs113615161 T C -0.0282 0.26373 0.02522 Acute appendicitis rs113615161 T C 0.0148 0.30781 0.01452 Colon cancer rs113615161 T C 0.02941 0.45356 0.03923 Rectal cancer rs113615161 T C -0.054 0.27809 0.04983 |
| Gastroesophageal reflux | rs1143770 | C | T | 0.01109 | 0.28668 | 0.01041 | Esophageal carcinoma | rs1143770 | C | T | 0.02173 | 0.74718 | 0.06741 | Acute gastritis | rs1143770 | C | T | -0.0106 | 0.74717 | 0.03289 | Chronic gastritis | rs1143770 | C | T | 0.00378 | 0.81997 | 0.01662 | Gastroduodenal ulcer | rs1143770 | C | T | 0.01417 | 0.40546 | 0.01704 | Gastric cancer | rs1143770 | C | T | 0.1209 | 0.0043 | 0.04235 | Fatty liver | rs1143770 | C | T | 0.0108 | 0.75505 | 0.03462 | Hepatic fibrosis | rs1143770 | C | T | 0.09151 | 0.49017 | 0.13261 | Cirrhosis | rs1143770 | C | T | -0.029 | 0.25684 | 0.02553liver cancer rs1143770 C T -0.0154 0.79619 0.05952 Choleliths rs1143770 C T 0.02261 0.01025 0.00881 Acute pancreatitis rs1143770 C T -0.0212 0.30269 0.02056 Chronic pancreatitis rs1143770 C T -0.0178 0.52216 0.02779 Pancreatic cancer rs1143770 C T -0.0434 0.31264 0.04295 Ulcerative colitis rs1143770 C T 0.00831 0.71494 0.02275 Crohn's disease rs1143770 C T -0.0271 0.48961 0.03923 Irrtable bowel syndrome rs1143770 C T 0.00683 0.68792 0.01701 Acute appendicitis rs1143770 C T -0.0027 0.78058 0.0098 Colon cancer rs1143770 C T 0.06243 0.01935 0.0267 Rectal cancer rs1143770 C T -0.0175 0.60488 0.03389 |
| Gastroesophageal reflux | rs115000530 | T | A | 0.01514 | 0.55984 | 0.02597 | Esophageal carcinoma | rs115000530 | T | A | -0.1439 | 0.38648 | 0.16619 | Acute gastritis | rs115000530 | T | A | 0.02567 | 0.75411 | 0.08197 | Chronic gastritis | rs115000530 | T | A | -0.0686 | 0.09735 | 0.0414 | Gastroduodenal ulcer | rs115000530 | T | A | -0.0199 | 0.6373 | 0.04224 | Gastric cancer | rs115000530 | T | A | -0.0485 | 0.65329 | 0.108 | Fatty liver | rs115000530 | T | A | -0.0959 | 0.26775 | 0.08651 | Hepatic fibrosis | rs115000530 | T | A | 0.66566 | 0.04225 | 0.32774 | Cirrhosis | rs115000530 | T | A | 0.06846 | 0.27902 | 0.06324liver cancer rs115000530 T A 0.0456 0.75445 0.1458 Choleliths rs115000530 T A 0.01119 0.61192 0.02206 Acute pancreatitis rs115000530 T A -0.0185 0.71805 0.05122 Chronic pancreatitis rs115000530 T A -0.0193 0.77984 0.06919 Pancreatic cancer rs115000530 T A -0.1542 0.14766 0.10649 Ulcerative colitis rs115000530 T A 0.01509 0.79067 0.05685 Crohn's disease rs115000530 T A -0.0694 0.48089 0.09848 Irrtable bowel syndrome rs115000530 T A -0.0089 0.83429 0.04262 Acute appendicitis rs115000530 T A -0.0101 0.6819 0.02453 Colon cancer rs115000530 T A 0.04212 0.52713 0.0666 Rectal cancer rs115000530 T A 0.03946 0.63977 0.08432 |
| Gastroesophageal reflux | rs11601122 | G | A | 0.00539 | 0.63305 | 0.01129 | Esophageal carcinoma | rs11601122 | G | A | 0.07066 | 0.33541 | 0.07335 | Acute gastritis | rs11601122 | G | A | -0.0404 | 0.2591 | 0.03578 | Chronic gastritis | rs11601122 | G | A | -0.01 | 0.58016 | 0.0181 | Gastroduodenal ulcer | rs11601122 | G | A | 0.00451 | 0.80742 | 0.01852 | Gastric cancer | rs11601122 | G | A | -0.0403 | 0.39147 | 0.04704 | Fatty liver | rs11601122 | G | A | 0.00642 | 0.86389 | 0.03748 | Hepatic fibrosis | rs11601122 | G | A | -0.0367 | 0.79768 | 0.14324 | Cirrhosis | rs11601122 | G | A | 0.03199 | 0.24797 | 0.02769liver cancer rs11601122 G A -0.0011 0.98642 0.06466 Choleliths rs11601122 G A -0.0073 0.44601 0.00956 Acute pancreatitis rs11601122 G A 0.0034 0.87888 0.02228 Chronic pancreatitis rs11601122 G A 0.0084 0.77949 0.03 Pancreatic cancer rs11601122 G A 0.01931 0.679 0.04667 Ulcerative colitis rs11601122 G A 0.01859 0.45047 0.02464 Crohn's disease rs11601122 G A 0.05877 0.16651 0.04248 Irrtable bowel syndrome rs11601122 G A 0.0208 0.25912 0.01843 Acute appendicitis rs11601122 G A -0.0153 0.14877 0.0106 Colon cancer rs11601122 G A -0.0296 0.30731 0.02903 Rectal cancer rs11601122 G A 0.00423 0.90865 0.03686 |
| Gastroesophageal reflux | rs11620355 | G | A | -0.0332 | 0.0761 | 0.01872 | Esophageal carcinoma | rs11620355 | G | A | -0.3124 | 0.00388 | 0.10818 | Acute gastritis | rs11620355 | G | A | -0.0853 | 0.14821 | 0.059 | Chronic gastritis | rs11620355 | G | A | -0.033 | 0.26946 | 0.02992 | Gastroduodenal ulcer | rs11620355 | G | A | 0.04791 | 0.11798 | 0.03065 | Gastric cancer | rs11620355 | G | A | -0.1456 | 0.06136 | 0.07785 | Fatty liver | rs11620355 | G | A | -0.0426 | 0.49082 | 0.06184 | Hepatic fibrosis | rs11620355 | G | A | 0.06901 | 0.77156 | 0.2377 | Cirrhosis | rs11620355 | G | A | 0.02701 | 0.55426 | 0.04566liver cancer rs11620355 G A 0.10226 0.33462 0.10598 Choleliths rs11620355 G A -0.0207 0.19097 0.01583 Acute pancreatitis rs11620355 G A -0.0351 0.34246 0.03693 Chronic pancreatitis rs11620355 G A -0.118 0.01763 0.0497 Pancreatic cancer rs11620355 G A -0.0834 0.27768 0.07684 Ulcerative colitis rs11620355 G A -0.0377 0.35581 0.04088 Crohn's disease rs11620355 G A -0.0531 0.45044 0.0704 Irrtable bowel syndrome rs11620355 G A 0.00306 0.92028 0.03054 Acute appendicitis rs11620355 G A -0.0437 0.01265 0.01754 Colon cancer rs11620355 G A 0.07972 0.09694 0.04803 Rectal cancer rs11620355 G A -0.0021 0.97202 0.06067 |
| Gastroesophageal reflux | rs11627087 | A | G | 0.01253 | 0.57898 | 0.02258 | Esophageal carcinoma | rs11627087 | A | G | -0.1291 | 0.37097 | 0.14429 | Acute gastritis | rs11627087 | A | G | 0.09659 | 0.1755 | 0.0713 | Chronic gastritis | rs11627087 | A | G | -0.0178 | 0.62043 | 0.03591 | Gastroduodenal ulcer | rs11627087 | A | G | -0.012 | 0.74547 | 0.03683 | Gastric cancer | rs11627087 | A | G | -0.0897 | 0.32988 | 0.09206 | Fatty liver | rs11627087 | A | G | -0.0793 | 0.28945 | 0.07482 | Hepatic fibrosis | rs11627087 | A | G | 0.12517 | 0.66782 | 0.29167 | Cirrhosis | rs11627087 | A | G | 0.01011 | 0.85609 | 0.05574liver cancer rs11627087 A G -0.0642 0.61675 0.12831 Choleliths rs11627087 A G 0.01111 0.55795 0.01897 Acute pancreatitis rs11627087 A G 0.00649 0.88455 0.04467 Chronic pancreatitis rs11627087 A G 0.0235 0.69945 0.06088 Pancreatic cancer rs11627087 A G -0.0004 0.99671 0.09245 Ulcerative colitis rs11627087 A G -0.0753 0.12806 0.04947 Crohn's disease rs11627087 A G -0.0566 0.50948 0.08583 Irrtable bowel syndrome rs11627087 A G 0.01852 0.61508 0.03683 Acute appendicitis rs11627087 A G -0.0278 0.19158 0.02133 Colon cancer rs11627087 A G 0.0227 0.69294 0.05749 Rectal cancer rs11627087 A G 0.08672 0.23417 0.0729 |
| Gastroesophageal reflux | rs11635092 | A | G | 0.00055 | 0.96078 | 0.01111 | Esophageal carcinoma | rs11635092 | A | G | 0.02275 | 0.75097 | 0.07168 | Acute gastritis | rs11635092 | A | G | -0.0071 | 0.83974 | 0.03512 | Chronic gastritis | rs11635092 | A | G | -0.0074 | 0.67477 | 0.01775 | Gastroduodenal ulcer | rs11635092 | A | G | 0.02807 | 0.12224 | 0.01816 | Gastric cancer | rs11635092 | A | G | 0.07215 | 0.11836 | 0.0462 | Fatty liver | rs11635092 | A | G | 0.04807 | 0.1928 | 0.03691 | Hepatic fibrosis | rs11635092 | A | G | -0.0332 | 0.81414 | 0.14142 | Cirrhosis | rs11635092 | A | G | 0.01771 | 0.51557 | 0.02723liver cancer rs11635092 A G -0.0378 0.55147 0.06353 Choleliths rs11635092 A G 0.0116 0.21648 0.00939 Acute pancreatitis rs11635092 A G 0.02442 0.26548 0.02193 Chronic pancreatitis rs11635092 A G -0.0175 0.55449 0.02962 Pancreatic cancer rs11635092 A G 0.05696 0.2146 0.0459 Ulcerative colitis rs11635092 A G 0.00285 0.90668 0.0243 Crohn's disease rs11635092 A G 0.07915 0.05901 0.04192 Irrtable bowel syndrome rs11635092 A G 0.01026 0.57275 0.01819 Acute appendicitis rs11635092 A G 0.00561 0.59173 0.01046 Colon cancer rs11635092 A G 0.01977 0.48667 0.02842 Rectal cancer rs11635092 A G 0.04644 0.19791 0.03607 |
| Gastroesophageal reflux | rs11657342 | A | G | -0.0009 | 0.92882 | 0.0103 | Esophageal carcinoma | rs11657342 | A | G | 0.07654 | 0.25297 | 0.06695 | Acute gastritis | rs11657342 | A | G | -0.0445 | 0.17078 | 0.03252 | Chronic gastritis | rs11657342 | A | G | 0.00609 | 0.71145 | 0.01647 | Gastroduodenal ulcer | rs11657342 | A | G | 0.00708 | 0.67467 | 0.01687 | Gastric cancer | rs11657342 | A | G | 0.02995 | 0.48581 | 0.04297 | Fatty liver | rs11657342 | A | G | -0.0182 | 0.59507 | 0.03421 | Hepatic fibrosis | rs11657342 | A | G | -0.0228 | 0.86158 | 0.13083 | Cirrhosis | rs11657342 | A | G | -0.016 | 0.52535 | 0.02527liver cancer rs11657342 A G -0.1192 0.04376 0.0591 Choleliths rs11657342 A G 0.01214 0.16376 0.00872 Acute pancreatitis rs11657342 A G 0.04157 0.04114 0.02036 Chronic pancreatitis rs11657342 A G 0.00572 0.83489 0.02743 Pancreatic cancer rs11657342 A G -0.0554 0.19463 0.04269 Ulcerative colitis rs11657342 A G -0.0485 0.03097 0.02248 Crohn's disease rs11657342 A G 0.02137 0.58108 0.03872 Irrtable bowel syndrome rs11657342 A G 0.00867 0.60679 0.01684 Acute appendicitis rs11657342 A G -0.0178 0.06568 0.00969 Colon cancer rs11657342 A G 0.03881 0.14294 0.02649 Rectal cancer rs11657342 A G 0.04347 0.19578 0.0336 |
| Gastroesophageal reflux | rs11663602 | A | C | 0.01478 | 0.15216 | 0.01032 | Esophageal carcinoma | rs11663602 | A | C | -0.069 | 0.30207 | 0.0669 | Acute gastritis | rs11663602 | A | C | 0.04827 | 0.13891 | 0.03262 | Chronic gastritis | rs11663602 | A | C | 0.00912 | 0.58084 | 0.01652 | Gastroduodenal ulcer | rs11663602 | A | C | 0.03332 | 0.04841 | 0.01688 | Gastric cancer | rs11663602 | A | C | 0.11026 | 0.01037 | 0.04301 | Fatty liver | rs11663602 | A | C | 0.02921 | 0.39256 | 0.03416 | Hepatic fibrosis | rs11663602 | A | C | 0.04118 | 0.75196 | 0.13028 | Cirrhosis | rs11663602 | A | C | 0.03385 | 0.1786 | 0.02517liver cancer rs11663602 A C -0.0084 0.88684 0.05897 Choleliths rs11663602 A C 0.01518 0.08123 0.00871 Acute pancreatitis rs11663602 A C 0.04121 0.04227 0.02029 Chronic pancreatitis rs11663602 A C 0.04932 0.07154 0.02737 Pancreatic cancer rs11663602 A C 0.05619 0.18548 0.04244 Ulcerative colitis rs11663602 A C 0.00338 0.88049 0.02246 Crohn's disease rs11663602 A C 0.01261 0.74478 0.03872 Irrtable bowel syndrome rs11663602 A C -0.0062 0.71433 0.01684 Acute appendicitis rs11663602 A C 0.01495 0.12168 0.00966 Colon cancer rs11663602 A C -0.0281 0.28902 0.02654 Rectal cancer rs11663602 A C 0.02341 0.48681 0.03367 |
| Gastroesophageal reflux | rs11678980 | G | A | 0.0014 | 0.88969 | 0.01009 | Esophageal carcinoma | rs11678980 | G | A | -0.0588 | 0.36634 | 0.06512 | Acute gastritis | rs11678980 | G | A | -0.0007 | 0.98364 | 0.03188 | Chronic gastritis | rs11678980 | G | A | -0.0042 | 0.7955 | 0.01612 | Gastroduodenal ulcer | rs11678980 | G | A | -0.0389 | 0.01848 | 0.01649 | Gastric cancer | rs11678980 | G | A | 0.02685 | 0.52238 | 0.04197 | Fatty liver | rs11678980 | G | A | -0.0685 | 0.04084 | 0.03351 | Hepatic fibrosis | rs11678980 | G | A | -0.1867 | 0.14438 | 0.12787 | Cirrhosis | rs11678980 | G | A | -0.0182 | 0.46139 | 0.02468liver cancer rs11678980 G A -0.0538 0.35063 0.05761 Choleliths rs11678980 G A 0.00867 0.30872 0.00851 Acute pancreatitis rs11678980 G A -0.0003 0.98786 0.0199 Chronic pancreatitis rs11678980 G A -0.0403 0.1334 0.02683 Pancreatic cancer rs11678980 G A -0.0077 0.8533 0.04153 Ulcerative colitis rs11678980 G A 0.02516 0.25383 0.02205 Crohn's disease rs11678980 G A -0.0382 0.31464 0.03802 Irrtable bowel syndrome rs11678980 G A -0.0438 0.00772 0.01643 Acute appendicitis rs11678980 G A -0.0037 0.69785 0.00948 Colon cancer rs11678980 G A -0.0344 0.18289 0.02586 Rectal cancer rs11678980 G A 0.02056 0.53129 0.03284 |
| Gastroesophageal reflux | rs11681861 | G | T | -0.0079 | 0.63475 | 0.01661 | Esophageal carcinoma | rs11681861 | G | T | -0.0129 | 0.90338 | 0.10652 | Acute gastritis | rs11681861 | G | T | -0.0612 | 0.24253 | 0.05239 | Chronic gastritis | rs11681861 | G | T | -0.0006 | 0.98117 | 0.0265 | Gastroduodenal ulcer | rs11681861 | G | T | 0.02917 | 0.28297 | 0.02717 | Gastric cancer | rs11681861 | G | T | -0.0754 | 0.2719 | 0.06865 | Fatty liver | rs11681861 | G | T | 0.08591 | 0.11945 | 0.05518 | Hepatic fibrosis | rs11681861 | G | T | 0.17093 | 0.41999 | 0.21196 | Cirrhosis | rs11681861 | G | T | -0.0086 | 0.83219 | 0.04062liver cancer rs11681861 G T 0.08994 0.33796 0.09386 Choleliths rs11681861 G T 0.01342 0.33864 0.01402 Acute pancreatitis rs11681861 G T -0.0178 0.5862 0.03278 Chronic pancreatitis rs11681861 G T 0.1015 0.02214 0.04436 Pancreatic cancer rs11681861 G T -0.0512 0.45197 0.06806 Ulcerative colitis rs11681861 G T 0.00107 0.97657 0.0363 Crohn's disease rs11681861 G T -0.0783 0.21136 0.06266 Irrtable bowel syndrome rs11681861 G T -0.0006 0.98323 0.02717 Acute appendicitis rs11681861 G T 0.00864 0.58035 0.01562 Colon cancer rs11681861 G T -0.023 0.58601 0.04229 Rectal cancer rs11681861 G T -0.0626 0.24219 0.05356 |
| Gastroesophageal reflux | rs11694904 | T | C | -0.0044 | 0.70857 | 0.01184 | Esophageal carcinoma | rs11694904 | T | C | -0.0402 | 0.59993 | 0.0766 | Acute gastritis | rs11694904 | T | C | -0.0451 | 0.22716 | 0.03737 | Chronic gastritis | rs11694904 | T | C | -0.0112 | 0.55519 | 0.01894 | Gastroduodenal ulcer | rs11694904 | T | C | -0.0218 | 0.26123 | 0.01936 | Gastric cancer | rs11694904 | T | C | -0.0386 | 0.42999 | 0.04893 | Fatty liver | rs11694904 | T | C | -0.0296 | 0.45146 | 0.03936 | Hepatic fibrosis | rs11694904 | T | C | -0.0553 | 0.71366 | 0.1508 | Cirrhosis | rs11694904 | T | C | 0.00203 | 0.94428 | 0.02909liver cancer rs11694904 T C -0.0725 0.28429 0.06766 Choleliths rs11694904 T C -0.0203 0.04246 0.01 Acute pancreatitis rs11694904 T C -0.0027 0.90719 0.02334 Chronic pancreatitis rs11694904 T C -0.0309 0.32669 0.03153 Pancreatic cancer rs11694904 T C -0.0012 0.97992 0.04872 Ulcerative colitis rs11694904 T C -0.0178 0.4916 0.02592 Crohn's disease rs11694904 T C 0.02545 0.569 0.04468 Irrtable bowel syndrome rs11694904 T C 0.01447 0.45452 0.01935 Acute appendicitis rs11694904 T C 0.01916 0.0853 0.01114 Colon cancer rs11694904 T C -0.0465 0.12612 0.03038 Rectal cancer rs11694904 T C -0.0481 0.21194 0.03853 |
| Gastroesophageal reflux | rs11732657 | A | G | 0.01171 | 0.31714 | 0.0117 | Esophageal carcinoma | rs11732657 | A | G | 0.05978 | 0.42991 | 0.07574 | Acute gastritis | rs11732657 | A | G | 0.01441 | 0.69605 | 0.03689 | Chronic gastritis | rs11732657 | A | G | 0.04291 | 0.02182 | 0.01871 | Gastroduodenal ulcer | rs11732657 | A | G | 0.03171 | 0.09711 | 0.01911 | Gastric cancer | rs11732657 | A | G | -0.0234 | 0.63077 | 0.04864 | Fatty liver | rs11732657 | A | G | 0.03616 | 0.35229 | 0.03887 | Hepatic fibrosis | rs11732657 | A | G | -0.1528 | 0.30347 | 0.14851 | Cirrhosis | rs11732657 | A | G | -0.0199 | 0.48671 | 0.02864liver cancer rs11732657 A G -0.017 0.79984 0.06701 Choleliths rs11732657 A G -0.0079 0.42544 0.0099 Acute pancreatitis rs11732657 A G 6.6E-05 0.99772 0.02308 Chronic pancreatitis rs11732657 A G 0.01696 0.58579 0.03112 Pancreatic cancer rs11732657 A G -0.0138 0.77561 0.04825 Ulcerative colitis rs11732657 A G 0.00846 0.74055 0.02555 Crohn's disease rs11732657 A G 0.08788 0.04641 0.04412 Irrtable bowel syndrome rs11732657 A G 0.0482 0.01184 0.01915 Acute appendicitis rs11732657 A G -0.0009 0.93156 0.01098 Colon cancer rs11732657 A G -0.0378 0.20817 0.03002 Rectal cancer rs11732657 A G 0.06926 0.06966 0.03818 |
| Gastroesophageal reflux | rs117468730 | G | A | 0.00222 | 0.95464 | 0.03901 | Esophageal carcinoma | rs117468730 | G | A | 0.42941 | 0.09549 | 0.25758 | Acute gastritis | rs117468730 | G | A | 0.26982 | 0.02804 | 0.12283 | Chronic gastritis | rs117468730 | G | A | -0.0015 | 0.98158 | 0.06288 | Gastroduodenal ulcer | rs117468730 | G | A | -0.0697 | 0.27737 | 0.06418 | Gastric cancer | rs117468730 | G | A | -0.2319 | 0.16245 | 0.16599 | Fatty liver | rs117468730 | G | A | -0.0321 | 0.80588 | 0.13063 | Hepatic fibrosis | rs117468730 | G | A | -1.1621 | 0.0182 | 0.4921 | Cirrhosis | rs117468730 | G | A | 0.01139 | 0.90557 | 0.09597liver cancer rs117468730 G A -0.074 0.74114 0.22404 Choleliths rs117468730 G A 0.01298 0.69609 0.03324 Acute pancreatitis rs117468730 G A 0.12231 0.11601 0.07782 Chronic pancreatitis rs117468730 G A 0.18202 0.08219 0.10472 Pancreatic cancer rs117468730 G A -0.0335 0.83749 0.16331 Ulcerative colitis rs117468730 G A -0.0341 0.69178 0.08596 Crohn's disease rs117468730 G A 0.04247 0.77666 0.14971 Irrtable bowel syndrome rs117468730 G A 0.09145 0.1543 0.0642 Acute appendicitis rs117468730 G A 0.0016 0.96532 0.03681 Colon cancer rs117468730 G A 0.00046 0.99644 0.10224 Rectal cancer rs117468730 G A 0.03645 0.77845 0.12955 |
| Gastroesophageal reflux | rs11752914 | T | C | -0.0211 | 0.1111 | 0.01323 | Esophageal carcinoma | rs11752914 | T | C | 0.01838 | 0.8314 | 0.08635 | Acute gastritis | rs11752914 | T | C | 0.09374 | 0.02509 | 0.04185 | Chronic gastritis | rs11752914 | T | C | -0.034 | 0.1077 | 0.02116 | Gastroduodenal ulcer | rs11752914 | T | C | 0.04595 | 0.03391 | 0.02166 | Gastric cancer | rs11752914 | T | C | 0.10937 | 0.04784 | 0.05527 | Fatty liver | rs11752914 | T | C | -0.0004 | 0.99186 | 0.04399 | Hepatic fibrosis | rs11752914 | T | C | 0.22337 | 0.1841 | 0.16817 | Cirrhosis | rs11752914 | T | C | 0.04771 | 0.14181 | 0.03248liver cancer rs11752914 T C -0.0241 0.75151 0.07616 Choleliths rs11752914 T C -0.0056 0.61861 0.0112 Acute pancreatitis rs11752914 T C 0.00716 0.78442 0.02616 Chronic pancreatitis rs11752914 T C -0.0558 0.11382 0.03527 Pancreatic cancer rs11752914 T C -0.0917 0.09417 0.0548 Ulcerative colitis rs11752914 T C 0.00979 0.73548 0.02897 Crohn's disease rs11752914 T C 0.01051 0.83327 0.04994 Irrtable bowel syndrome rs11752914 T C -0.0196 0.36494 0.02161 Acute appendicitis rs11752914 T C 0.02081 0.09443 0.01244 Colon cancer rs11752914 T C -0.0203 0.55048 0.03396 Rectal cancer rs11752914 T C 0.01997 0.64463 0.0433 |
| Gastroesophageal reflux | rs11772580 | G | T | -0.0176 | 0.12177 | 0.01135 | Esophageal carcinoma | rs11772580 | G | T | 0.18568 | 0.0118 | 0.07374 | Acute gastritis | rs11772580 | G | T | 0.0238 | 0.50621 | 0.03581 | Chronic gastritis | rs11772580 | G | T | -0.0242 | 0.18281 | 0.01815 | Gastroduodenal ulcer | rs11772580 | G | T | 0.01085 | 0.5591 | 0.01858 | Gastric cancer | rs11772580 | G | T | -0.0315 | 0.50362 | 0.04709 | Fatty liver | rs11772580 | G | T | -0.0153 | 0.68412 | 0.03767 | Hepatic fibrosis | rs11772580 | G | T | -0.0755 | 0.60004 | 0.14396 | Cirrhosis | rs11772580 | G | T | 0.00533 | 0.84776 | 0.02778liver cancer rs11772580 G T -0.0279 0.66588 0.0647 Choleliths rs11772580 G T -0.0094 0.32863 0.00961 Acute pancreatitis rs11772580 G T -0.0076 0.735 0.02241 Chronic pancreatitis rs11772580 G T -0.0224 0.45813 0.0302 Pancreatic cancer rs11772580 G T -0.0654 0.16124 0.04671 Ulcerative colitis rs11772580 G T -0.0363 0.14252 0.02476 Crohn's disease rs11772580 G T 0.04222 0.32264 0.04269 Irrtable bowel syndrome rs11772580 G T 0.00455 0.80646 0.01855 Acute appendicitis rs11772580 G T -0.0069 0.51846 0.01067 Colon cancer rs11772580 G T -0.0203 0.48754 0.02921 Rectal cancer rs11772580 G T 0.00047 0.98982 0.03697 |
| Gastroesophageal reflux | rs117799466 | G | C | 0.00582 | 0.5638 | 0.01009 | Esophageal carcinoma | rs117799466 | G | C | -0.0175 | 0.78846 | 0.06524 | Acute gastritis | rs117799466 | G | C | 0.05382 | 0.09129 | 0.03187 | Chronic gastritis | rs117799466 | G | C | -0.0308 | 0.05595 | 0.01614 | Gastroduodenal ulcer | rs117799466 | G | C | 0.00408 | 0.80465 | 0.0165 | Gastric cancer | rs117799466 | G | C | -0.0194 | 0.64402 | 0.04197 | Fatty liver | rs117799466 | G | C | -0.007 | 0.83428 | 0.03346 | Hepatic fibrosis | rs117799466 | G | C | -0.0147 | 0.90816 | 0.12727 | Cirrhosis | rs117799466 | G | C | -0.025 | 0.30993 | 0.02465liver cancer rs117799466 G C -0.1073 0.06254 0.05764 Choleliths rs117799466 G C 0.0167 0.05034 0.00854 Acute pancreatitis rs117799466 G C 0.02438 0.21939 0.01985 Chronic pancreatitis rs117799466 G C -0.0084 0.75453 0.02676 Pancreatic cancer rs117799466 G C 0.02524 0.54435 0.04164 Ulcerative colitis rs117799466 G C 0.00521 0.81253 0.02197 Crohn's disease rs117799466 G C -0.0002 0.99679 0.03791 Irrtable bowel syndrome rs117799466 G C 0.01234 0.4542 0.01649 Acute appendicitis rs117799466 G C 0.00154 0.8711 0.00947 Colon cancer rs117799466 G C -0.0292 0.26025 0.02594 Rectal cancer rs117799466 G C -0.05 0.12825 0.03286 |
| Gastroesophageal reflux | rs11871429 | A | G | ####### | 0.99599 | 0.01151 | Esophageal carcinoma | rs11871429 | A | G | -0.0344 | 0.6434 | 0.07436 | Acute gastritis | rs11871429 | A | G | 0.02152 | 0.55282 | 0.03626 | Chronic gastritis | rs11871429 | A | G | 0.03597 | 0.05079 | 0.01842 | Gastroduodenal ulcer | rs11871429 | A | G | -0.0103 | 0.58257 | 0.01878 | Gastric cancer | rs11871429 | A | G | 0.06118 | 0.20278 | 0.04803 | Fatty liver | rs11871429 | A | G | -0.0961 | 0.01176 | 0.03815 | Hepatic fibrosis | rs11871429 | A | G | 0.0366 | 0.8005 | 0.14483 | Cirrhosis | rs11871429 | A | G | -0.0081 | 0.77187 | 0.02806liver cancer rs11871429 A G 0.04624 0.48053 0.06555 Choleliths rs11871429 A G 0.01571 0.10624 0.00973 Acute pancreatitis rs11871429 A G -0.0045 0.8429 0.02265 Chronic pancreatitis rs11871429 A G -0.0111 0.71453 0.03045 Pancreatic cancer rs11871429 A G -0.0601 0.20322 0.04725 Ulcerative colitis rs11871429 A G 0.04481 0.07371 0.02506 Crohn's disease rs11871429 A G -0.07 0.10568 0.04327 Irrtable bowel syndrome rs11871429 A G 0.00073 0.96917 0.0188 Acute appendicitis rs11871429 A G -0.0084 0.43738 0.01077 Colon cancer rs11871429 A G 0.03046 0.30365 0.02961 Rectal cancer rs11871429 A G -0.0036 0.92447 0.03757 |
| Gastroesophageal reflux | rs12028010 | C | T | 0.01888 | 0.10738 | 0.01173 | Esophageal carcinoma | rs12028010 | C | T | 0.01571 | 0.83631 | 0.07601 | Acute gastritis | rs12028010 | C | T | -0.0128 | 0.72912 | 0.03707 | Chronic gastritis | rs12028010 | C | T | 0.00843 | 0.65283 | 0.01874 | Gastroduodenal ulcer | rs12028010 | C | T | 0.00285 | 0.88208 | 0.01919 | Gastric cancer | rs12028010 | C | T | -0.0138 | 0.7784 | 0.04893 | Fatty liver | rs12028010 | C | T | -0.0459 | 0.2391 | 0.03898 | Hepatic fibrosis | rs12028010 | C | T | 0.04813 | 0.74587 | 0.14851 | Cirrhosis | rs12028010 | C | T | 0.01386 | 0.62859 | 0.02865liver cancer rs12028010 C T -0.0563 0.40232 0.06725 Choleliths rs12028010 C T -0.0214 0.0314 0.00993 Acute pancreatitis rs12028010 C T -0.0154 0.504 0.02312 Chronic pancreatitis rs12028010 C T -0.0005 0.98641 0.03116 Pancreatic cancer rs12028010 C T 0.0066 0.89119 0.04827 Ulcerative colitis rs12028010 C T -0.0252 0.32649 0.02563 Crohn's disease rs12028010 C T -0.0079 0.85885 0.04421 Irrtable bowel syndrome rs12028010 C T 0.01201 0.53198 0.01922 Acute appendicitis rs12028010 C T -0.0139 0.2077 0.01104 Colon cancer rs12028010 C T 0.05056 0.09258 0.03006 Rectal cancer rs12028010 C T -0.0112 0.76903 0.03825 |
| Gastroesophageal reflux | rs12134151 | C | G | 0.01014 | 0.30471 | 0.00988 | Esophageal carcinoma | rs12134151 | C | G | 0.13986 | 0.02871 | 0.06394 | Acute gastritis | rs12134151 | C | G | 0.04336 | 0.16503 | 0.03123 | Chronic gastritis | rs12134151 | C | G | -0.002 | 0.89965 | 0.01579 | Gastroduodenal ulcer | rs12134151 | C | G | 0.00295 | 0.85538 | 0.01617 | Gastric cancer | rs12134151 | C | G | -0.0429 | 0.29592 | 0.04102 | Fatty liver | rs12134151 | C | G | -0.0065 | 0.84388 | 0.03281 | Hepatic fibrosis | rs12134151 | C | G | 0.17234 | 0.16893 | 0.12528 | Cirrhosis | rs12134151 | C | G | 0.02647 | 0.27336 | 0.02416liver cancer rs12134151 C G -0.047 0.40445 0.05643 Choleliths rs12134151 C G 0.00522 0.53298 0.00837 Acute pancreatitis rs12134151 C G -0.0244 0.2104 0.01948 Chronic pancreatitis rs12134151 C G -0.0205 0.43498 0.02624 Pancreatic cancer rs12134151 C G 0.03409 0.40259 0.04073 Ulcerative colitis rs12134151 C G 0.00723 0.73728 0.02155 Crohn's disease rs12134151 C G -0.0215 0.56244 0.0372 Irrtable bowel syndrome rs12134151 C G 0.01469 0.36326 0.01615 Acute appendicitis rs12134151 C G -0.0179 0.05398 0.00928 Colon cancer rs12134151 C G 0.00048 0.98498 0.02535 Rectal cancer rs12134151 C G 0.00319 0.9209 0.03216 |
| Gastroesophageal reflux | rs12332731 | A | T | -0.0502 | 0.01394 | 0.02041 | Esophageal carcinoma | rs12332731 | A | T | 0.01762 | 0.89321 | 0.13122 | Acute gastritis | rs12332731 | A | T | 0.04651 | 0.4693 | 0.06428 | Chronic gastritis | rs12332731 | A | T | -0.0662 | 0.04119 | 0.03242 | Gastroduodenal ulcer | rs12332731 | A | T | -0.0643 | 0.05542 | 0.03358 | Gastric cancer | rs12332731 | A | T | -0.1294 | 0.12389 | 0.08411 | Fatty liver | rs12332731 | A | T | -0.0078 | 0.90935 | 0.06831 | Hepatic fibrosis | rs12332731 | A | T | -0.2939 | 0.26936 | 0.26608 | Cirrhosis | rs12332731 | A | T | -0.043 | 0.39859 | 0.05098liver cancer rs12332731 A T 0.01906 0.86962 0.11612 Choleliths rs12332731 A T 0.01275 0.4615 0.01732 Acute pancreatitis rs12332731 A T -0.0535 0.19139 0.04097 Chronic pancreatitis rs12332731 A T -0.1061 0.05679 0.0557 Pancreatic cancer rs12332731 A T -0.0506 0.54717 0.08405 Ulcerative colitis rs12332731 A T 0.01607 0.72238 0.04522 Crohn's disease rs12332731 A T -0.0469 0.54664 0.07772 Irrtable bowel syndrome rs12332731 A T -0.0447 0.18235 0.03349 Acute appendicitis rs12332731 A T 0.00614 0.7525 0.01949 Colon cancer rs12332731 A T -0.0475 0.36004 0.05188 Rectal cancer rs12332731 A T -0.0781 0.23501 0.06574 |
| Gastroesophageal reflux | rs12375949 | T | C | -0.0043 | 0.66937 | 0.00997 | Esophageal carcinoma | rs12375949 | T | C | -0.0378 | 0.5582 | 0.06457 | Acute gastritis | rs12375949 | T | C | -0.0384 | 0.22269 | 0.03152 | Chronic gastritis | rs12375949 | T | C | -0.0136 | 0.39472 | 0.01595 | Gastroduodenal ulcer | rs12375949 | T | C | -0.0212 | 0.19359 | 0.01631 | Gastric cancer | rs12375949 | T | C | 0.00218 | 0.95801 | 0.04144 | Fatty liver | rs12375949 | T | C | 0.03914 | 0.23719 | 0.03311 | Hepatic fibrosis | rs12375949 | T | C | -0.1426 | 0.25919 | 0.12637 | Cirrhosis | rs12375949 | T | C | -0.016 | 0.51268 | 0.0244liver cancer rs12375949 T C 0.08737 0.12445 0.05687 Choleliths rs12375949 T C -0.003 0.7224 0.00843 Acute pancreatitis rs12375949 T C -0.0099 0.61535 0.01967 Chronic pancreatitis rs12375949 T C -0.0246 0.35428 0.02655 Pancreatic cancer rs12375949 T C -0.0251 0.54125 0.04102 Ulcerative colitis rs12375949 T C -0.0139 0.52282 0.02181 Crohn's disease rs12375949 T C -0.0162 0.66604 0.0376 Irrtable bowel syndrome rs12375949 T C -0.0157 0.33591 0.01629 Acute appendicitis rs12375949 T C 0.00328 0.72652 0.00938 Colon cancer rs12375949 T C -0.0297 0.24518 0.02554 Rectal cancer rs12375949 T C 0.01227 0.70518 0.03243 |
| Gastroesophageal reflux | rs12468040 | G | T | -0.0036 | 0.71907 | 0.00989 | Esophageal carcinoma | rs12468040 | G | T | -0.056 | 0.38079 | 0.06393 | Acute gastritis | rs12468040 | G | T | -0.0254 | 0.41673 | 0.03125 | Chronic gastritis | rs12468040 | G | T | -0.0185 | 0.24146 | 0.01582 | Gastroduodenal ulcer | rs12468040 | G | T | 0.01458 | 0.36753 | 0.01618 | Gastric cancer | rs12468040 | G | T | -0.02 | 0.62587 | 0.04107 | Fatty liver | rs12468040 | G | T | 0.01705 | 0.60335 | 0.03281 | Hepatic fibrosis | rs12468040 | G | T | -0.0644 | 0.60757 | 0.12531 | Cirrhosis | rs12468040 | G | T | 0.03055 | 0.20668 | 0.02419liver cancer rs12468040 G T 0.06096 0.27921 0.05634 Choleliths rs12468040 G T -0.0121 0.14837 0.00835 Acute pancreatitis rs12468040 G T 0.03223 0.09832 0.0195 Chronic pancreatitis rs12468040 G T 0.03951 0.13282 0.02629 Pancreatic cancer rs12468040 G T -0.0136 0.73771 0.0407 Ulcerative colitis rs12468040 G T -0.0046 0.82963 0.02157 Crohn's disease rs12468040 G T 0.01984 0.594 0.03723 Irrtable bowel syndrome rs12468040 G T -0.0399 0.01356 0.01616 Acute appendicitis rs12468040 G T 0.00423 0.64831 0.00928 Colon cancer rs12468040 G T -0.0195 0.44297 0.02535 Rectal cancer rs12468040 G T -0.0322 0.31789 0.03222 |
| Gastroesophageal reflux | rs12503522 | C | T | 0.00519 | 0.62555 | 0.01064 | Esophageal carcinoma | rs12503522 | C | T | -0.1416 | 0.04119 | 0.06937 | Acute gastritis | rs12503522 | C | T | -0.0277 | 0.4091 | 0.03358 | Chronic gastritis | rs12503522 | C | T | 0.0312 | 0.06666 | 0.01701 | Gastroduodenal ulcer | rs12503522 | C | T | -0.0113 | 0.5171 | 0.01744 | Gastric cancer | rs12503522 | C | T | 0.01775 | 0.6891 | 0.04437 | Fatty liver | rs12503522 | C | T | -0.0214 | 0.54591 | 0.03537 | Hepatic fibrosis | rs12503522 | C | T | 0.0193 | 0.88669 | 0.13541 | Cirrhosis | rs12503522 | C | T | -0.0286 | 0.27438 | 0.02613liver cancer rs12503522 C T 0.10755 0.07938 0.0613 Choleliths rs12503522 C T -0.0106 0.24064 0.00899 Acute pancreatitis rs12503522 C T 0.0156 0.45812 0.02103 Chronic pancreatitis rs12503522 C T 0.02002 0.48073 0.02839 Pancreatic cancer rs12503522 C T -0.0208 0.63818 0.04413 Ulcerative colitis rs12503522 C T 0.01273 0.5841 0.02326 Crohn's disease rs12503522 C T -0.0537 0.18108 0.04016 Irrtable bowel syndrome rs12503522 C T 0.01606 0.35502 0.01737 Acute appendicitis rs12503522 C T -0.0092 0.35713 0.01001 Colon cancer rs12503522 C T -0.0629 0.02185 0.02742 Rectal cancer rs12503522 C T -0.0526 0.13107 0.03481 |
| Gastroesophageal reflux | rs12519073 | C | T | -0.0268 | 0.02349 | 0.01184 | Esophageal carcinoma | rs12519073 | C | T | -0.0861 | 0.26201 | 0.07678 | Acute gastritis | rs12519073 | C | T | -0.0647 | 0.08426 | 0.03749 | Chronic gastritis | rs12519073 | C | T | 0.00462 | 0.80704 | 0.01892 | Gastroduodenal ulcer | rs12519073 | C | T | -0.0179 | 0.35643 | 0.01941 | Gastric cancer | rs12519073 | C | T | -0.0221 | 0.65437 | 0.0493 | Fatty liver | rs12519073 | C | T | -0.0313 | 0.4268 | 0.03937 | Hepatic fibrosis | rs12519073 | C | T | 0.09732 | 0.51834 | 0.15067 | Cirrhosis | rs12519073 | C | T | 0.00256 | 0.92991 | 0.02905liver cancer rs12519073 C T 0.08422 0.21475 0.06788 Choleliths rs12519073 C T 0.00785 0.43343 0.01002 Acute pancreatitis rs12519073 C T -0.0102 0.66386 0.02339 Chronic pancreatitis rs12519073 C T -0.0426 0.17723 0.03159 Pancreatic cancer rs12519073 C T 0.04747 0.33259 0.04899 Ulcerative colitis rs12519073 C T 0.00304 0.90652 0.02591 Crohn's disease rs12519073 C T 0.00978 0.82706 0.04475 Irrtable bowel syndrome rs12519073 C T 0.01172 0.54514 0.01936 Acute appendicitis rs12519073 C T 0.0045 0.68659 0.01115 Colon cancer rs12519073 C T -0.0428 0.15866 0.03035 Rectal cancer rs12519073 C T 0.05086 0.18767 0.0386 |
| Gastroesophageal reflux | rs12574281 | A | C | 0.0094 | 0.36465 | 0.01037 | Esophageal carcinoma | rs12574281 | A | C | 0.11354 | 0.09034 | 0.06704 | Acute gastritis | rs12574281 | A | C | 0.03232 | 0.32311 | 0.0327 | Chronic gastritis | rs12574281 | A | C | 0.02169 | 0.19069 | 0.01657 | Gastroduodenal ulcer | rs12574281 | A | C | 0.00821 | 0.62759 | 0.01693 | Gastric cancer | rs12574281 | A | C | -0.0809 | 0.06002 | 0.04301 | Fatty liver | rs12574281 | A | C | 0.02755 | 0.42352 | 0.03442 | Hepatic fibrosis | rs12574281 | A | C | 0.05726 | 0.66292 | 0.13136 | Cirrhosis | rs12574281 | A | C | -0.0136 | 0.59174 | 0.02532liver cancer rs12574281 A C -0.0704 0.23285 0.059 Choleliths rs12574281 A C -0.0125 0.15408 0.00876 Acute pancreatitis rs12574281 A C 0.05479 0.00753 0.0205 Chronic pancreatitis rs12574281 A C -0.0295 0.28382 0.02751 Pancreatic cancer rs12574281 A C -0.0387 0.36447 0.04263 Ulcerative colitis rs12574281 A C 0.00289 0.89816 0.02262 Crohn's disease rs12574281 A C 0.08271 0.03431 0.03908 Irrtable bowel syndrome rs12574281 A C -0.0094 0.57674 0.01691 Acute appendicitis rs12574281 A C 0.00164 0.86623 0.00973 Colon cancer rs12574281 A C -0.0283 0.28753 0.02658 Rectal cancer rs12574281 A C 0.04169 0.21713 0.03378 |
| Gastroesophageal reflux | rs12602286 | T | G | -0.031 | 0.01752 | 0.01303 | Esophageal carcinoma | rs12602286 | T | G | -0.2062 | 0.01471 | 0.08454 | Acute gastritis | rs12602286 | T | G | -0.0132 | 0.74964 | 0.04125 | Chronic gastritis | rs12602286 | T | G | -0.0119 | 0.5684 | 0.02087 | Gastroduodenal ulcer | rs12602286 | T | G | -0.0143 | 0.50327 | 0.02133 | Gastric cancer | rs12602286 | T | G | -0.0253 | 0.64317 | 0.05451 | Fatty liver | rs12602286 | T | G | 0.04661 | 0.28147 | 0.04328 | Hepatic fibrosis | rs12602286 | T | G | 0.12009 | 0.46324 | 0.16372 | Cirrhosis | rs12602286 | T | G | 0.01393 | 0.66032 | 0.0317liver cancer rs12602286 T G -0.0881 0.23455 0.07412 Choleliths rs12602286 T G -0.0002 0.98483 0.01104 Acute pancreatitis rs12602286 T G 0.00229 0.92895 0.02563 Chronic pancreatitis rs12602286 T G -0.0227 0.50933 0.03446 Pancreatic cancer rs12602286 T G -0.0451 0.4005 0.05364 Ulcerative colitis rs12602286 T G 0.04721 0.0961 0.02837 Crohn's disease rs12602286 T G -0.0112 0.81866 0.04905 Irrtable bowel syndrome rs12602286 T G -0.0158 0.45749 0.02132 Acute appendicitis rs12602286 T G 0.00346 0.77675 0.01222 Colon cancer rs12602286 T G -0.0243 0.46762 0.03351 Rectal cancer rs12602286 T G -0.0358 0.40098 0.04262 |
| Gastroesophageal reflux | rs12643771 | C | T | 0.00176 | 0.87726 | 0.01137 | Esophageal carcinoma | rs12643771 | C | T | -0.0169 | 0.81815 | 0.07362 | Acute gastritis | rs12643771 | C | T | 0.00439 | 0.90264 | 0.03585 | Chronic gastritis | rs12643771 | C | T | 0.00245 | 0.89239 | 0.01814 | Gastroduodenal ulcer | rs12643771 | C | T | 0.00503 | 0.78666 | 0.01858 | Gastric cancer | rs12643771 | C | T | 0.05053 | 0.28574 | 0.04733 | Fatty liver | rs12643771 | C | T | 0.02088 | 0.58013 | 0.03775 | Hepatic fibrosis | rs12643771 | C | T | 0.09081 | 0.52774 | 0.14381 | Cirrhosis | rs12643771 | C | T | 0.00063 | 0.9818 | 0.02781liver cancer rs12643771 C T 0.01659 0.7983 0.06494 Choleliths rs12643771 C T 0.02315 0.01611 0.00962 Acute pancreatitis rs12643771 C T -0.01 0.65608 0.02244 Chronic pancreatitis rs12643771 C T -0.0316 0.29619 0.03025 Pancreatic cancer rs12643771 C T -0.0157 0.73721 0.0469 Ulcerative colitis rs12643771 C T -0.0188 0.45008 0.02486 Crohn's disease rs12643771 C T -0.0128 0.76513 0.04291 Irrtable bowel syndrome rs12643771 C T 0.0221 0.23395 0.01857 Acute appendicitis rs12643771 C T -0.0197 0.06501 0.01069 Colon cancer rs12643771 C T 0.01774 0.54418 0.02926 Rectal cancer rs12643771 C T 0.01889 0.61047 0.03708 |
| Gastroesophageal reflux | rs12647336 | A | G | -0.0183 | 0.20685 | 0.01453 | Esophageal carcinoma | rs12647336 | A | G | 0.01621 | 0.86331 | 0.09414 | Acute gastritis | rs12647336 | A | G | 0.02414 | 0.59937 | 0.04595 | Chronic gastritis | rs12647336 | A | G | -0.0182 | 0.43328 | 0.02326 | Gastroduodenal ulcer | rs12647336 | A | G | -0.0232 | 0.32999 | 0.02382 | Gastric cancer | rs12647336 | A | G | -0.0464 | 0.44369 | 0.06059 | Fatty liver | rs12647336 | A | G | -0.0822 | 0.08874 | 0.04828 | Hepatic fibrosis | rs12647336 | A | G | -0.2119 | 0.25086 | 0.18452 | Cirrhosis | rs12647336 | A | G | -0.0049 | 0.89119 | 0.03562liver cancer rs12647336 A G 0.05422 0.5127 0.08282 Choleliths rs12647336 A G -0.015 0.22441 0.01231 Acute pancreatitis rs12647336 A G 0.03144 0.27393 0.02874 Chronic pancreatitis rs12647336 A G 0.04872 0.20846 0.03873 Pancreatic cancer rs12647336 A G 0.06025 0.31374 0.05981 Ulcerative colitis rs12647336 A G -0.0495 0.1191 0.03176 Crohn's disease rs12647336 A G 0.00397 0.94219 0.05478 Irrtable bowel syndrome rs12647336 A G -0.0098 0.67962 0.02372 Acute appendicitis rs12647336 A G -0.0284 0.03733 0.01366 Colon cancer rs12647336 A G 0.06789 0.06878 0.03731 Rectal cancer rs12647336 A G 0.11334 0.01656 0.0473 |
| Gastroesophageal reflux | rs12655753 | A | G | 0.0264 | 0.18617 | 0.01997 | Esophageal carcinoma | rs12655753 | A | G | 0.06127 | 0.63729 | 0.12996 | Acute gastritis | rs12655753 | A | G | -0.0105 | 0.8684 | 0.06323 | Chronic gastritis | rs12655753 | A | G | -0.0054 | 0.86734 | 0.03206 | Gastroduodenal ulcer | rs12655753 | A | G | -0.0362 | 0.27041 | 0.03282 | Gastric cancer | rs12655753 | A | G | -0.0766 | 0.35742 | 0.08321 | Fatty liver | rs12655753 | A | G | -0.0165 | 0.80421 | 0.06645 | Hepatic fibrosis | rs12655753 | A | G | -0.1071 | 0.67474 | 0.25517 | Cirrhosis | rs12655753 | A | G | 0.07456 | 0.12863 | 0.04907liver cancer rs12655753 A G -0.011 0.92305 0.11423 Choleliths rs12655753 A G -0.0533 0.00182 0.01708 Acute pancreatitis rs12655753 A G -0.0167 0.6711 0.03936 Chronic pancreatitis rs12655753 A G 0.07125 0.18058 0.05321 Pancreatic cancer rs12655753 A G 0.03895 0.63794 0.08276 Ulcerative colitis rs12655753 A G -0.0275 0.5301 0.04373 Crohn's disease rs12655753 A G 0.14779 0.04923 0.07515 Irrtable bowel syndrome rs12655753 A G 0.0431 0.18744 0.0327 Acute appendicitis rs12655753 A G 0.03609 0.05489 0.0188 Colon cancer rs12655753 A G 0.02139 0.67599 0.05118 Rectal cancer rs12655753 A G -0.1981 0.0037 0.06825 |
| Gastroesophageal reflux | rs12682775 | T | C | 0.01932 | 0.0731 | 0.01078 | Esophageal carcinoma | rs12682775 | T | C | 0.15186 | 0.02976 | 0.06988 | Acute gastritis | rs12682775 | T | C | 0.0498 | 0.14369 | 0.03406 | Chronic gastritis | rs12682775 | T | C | 0.01372 | 0.42603 | 0.01723 | Gastroduodenal ulcer | rs12682775 | T | C | 0.01147 | 0.51512 | 0.01763 | Gastric cancer | rs12682775 | T | C | 0.03943 | 0.38148 | 0.04505 | Fatty liver | rs12682775 | T | C | -0.0126 | 0.72434 | 0.03586 | Hepatic fibrosis | rs12682775 | T | C | 0.26793 | 0.0486 | 0.13586 | Cirrhosis | rs12682775 | T | C | 0.04605 | 0.08009 | 0.02631liver cancer rs12682775 T C -0.0317 0.60685 0.06164 Choleliths rs12682775 T C 0.00562 0.53783 0.00913 Acute pancreatitis rs12682775 T C 0.01605 0.44968 0.02124 Chronic pancreatitis rs12682775 T C 0.02262 0.42924 0.02861 Pancreatic cancer rs12682775 T C 0.05086 0.25266 0.04446 Ulcerative colitis rs12682775 T C -0.0168 0.47514 0.02354 Crohn's disease rs12682775 T C -0.0106 0.79484 0.04059 Irrtable bowel syndrome rs12682775 T C -0.0244 0.16672 0.01763 Acute appendicitis rs12682775 T C -0.0007 0.94851 0.01013 Colon cancer rs12682775 T C -0.0412 0.13841 0.02778 Rectal cancer rs12682775 T C -0.0041 0.90785 0.03525 |
| Gastroesophageal reflux | rs12804787 | A | G | -0.0444 | 0.06267 | 0.02385 | Esophageal carcinoma | rs12804787 | A | G | 0.04355 | 0.78052 | 0.15628 | Acute gastritis | rs12804787 | A | G | -0.0132 | 0.86138 | 0.07533 | Chronic gastritis | rs12804787 | A | G | -0.0245 | 0.52351 | 0.03836 | Gastroduodenal ulcer | rs12804787 | A | G | -0.0074 | 0.84988 | 0.03933 | Gastric cancer | rs12804787 | A | G | 0.11143 | 0.2649 | 0.09995 | Fatty liver | rs12804787 | A | G | -0.0567 | 0.47419 | 0.07918 | Hepatic fibrosis | rs12804787 | A | G | -0.2158 | 0.47426 | 0.30155 | Cirrhosis | rs12804787 | A | G | -0.1148 | 0.05056 | 0.05871liver cancer rs12804787 A G -0.1042 0.45089 0.13826 Choleliths rs12804787 A G 0.00267 0.89476 0.02019 Acute pancreatitis rs12804787 A G -0.0263 0.57744 0.04719 Chronic pancreatitis rs12804787 A G -0.0409 0.52091 0.06375 Pancreatic cancer rs12804787 A G -0.0365 0.71333 0.09935 Ulcerative colitis rs12804787 A G -0.0807 0.11971 0.05185 Crohn's disease rs12804787 A G 0.03805 0.66978 0.08922 Irrtable bowel syndrome rs12804787 A G -0.028 0.47234 0.03897 Acute appendicitis rs12804787 A G 0.01443 0.51934 0.02239 Colon cancer rs12804787 A G 0.08584 0.16403 0.06168 Rectal cancer rs12804787 A G 0.09328 0.23307 0.07822 |
| Gastroesophageal reflux | rs1291818 | C | T | 0.01651 | 0.09454 | 0.00988 | Esophageal carcinoma | rs1291818 | C | T | 0.00196 | 0.9756 | 0.06395 | Acute gastritis | rs1291818 | C | T | -0.0321 | 0.30315 | 0.03117 | Chronic gastritis | rs1291818 | C | T | 0.01244 | 0.43032 | 0.01577 | Gastroduodenal ulcer | rs1291818 | C | T | 0.03423 | 0.03397 | 0.01614 | Gastric cancer | rs1291818 | C | T | -0.0551 | 0.17981 | 0.04107 | Fatty liver | rs1291818 | C | T | -0.0176 | 0.5915 | 0.03277 | Hepatic fibrosis | rs1291818 | C | T | -0.0656 | 0.60003 | 0.12516 | Cirrhosis | rs1291818 | C | T | 0.00595 | 0.80519 | 0.02411liver cancer rs1291818 C T 0.04141 0.46244 0.05635 Choleliths rs1291818 C T 0.01301 0.11922 0.00835 Acute pancreatitis rs1291818 C T 0.0277 0.15425 0.01944 Chronic pancreatitis rs1291818 C T -0.0117 0.65416 0.02619 Pancreatic cancer rs1291818 C T -0.0406 0.31806 0.04067 Ulcerative colitis rs1291818 C T -0.0188 0.38264 0.02154 Crohn's disease rs1291818 C T 0.02899 0.43535 0.03716 Irrtable bowel syndrome rs1291818 C T 0.00281 0.8617 0.01613 Acute appendicitis rs1291818 C T 0.00645 0.4869 0.00927 Colon cancer rs1291818 C T 0.00222 0.93025 0.02533 Rectal cancer rs1291818 C T 0.0342 0.28779 0.03217 |
| Gastroesophageal reflux | rs12940014 | C | T | 0.00804 | 0.41427 | 0.00985 | Esophageal carcinoma | rs12940014 | C | T | -0.0462 | 0.4682 | 0.06372 | Acute gastritis | rs12940014 | C | T | -0.0158 | 0.6108 | 0.03108 | Chronic gastritis | rs12940014 | C | T | -0.0205 | 0.19294 | 0.01576 | Gastroduodenal ulcer | rs12940014 | C | T | 0.01298 | 0.42023 | 0.01611 | Gastric cancer | rs12940014 | C | T | -0.01 | 0.80664 | 0.04098 | Fatty liver | rs12940014 | C | T | -0.0495 | 0.1304 | 0.03272 | Hepatic fibrosis | rs12940014 | C | T | 0.15507 | 0.21392 | 0.12477 | Cirrhosis | rs12940014 | C | T | -0.0085 | 0.72353 | 0.02407liver cancer rs12940014 C T 0.06665 0.23549 0.05618 Choleliths rs12940014 C T 0.01834 0.02786 0.00834 Acute pancreatitis rs12940014 C T 0.03143 0.10542 0.01941 Chronic pancreatitis rs12940014 C T -0.0236 0.36734 0.02618 Pancreatic cancer rs12940014 C T 0.01198 0.76768 0.04055 Ulcerative colitis rs12940014 C T 0.00219 0.91893 0.02149 Crohn's disease rs12940014 C T 0.01503 0.68513 0.03707 Irrtable bowel syndrome rs12940014 C T -0.0054 0.73978 0.01611 Acute appendicitis rs12940014 C T 0.007 0.44938 0.00925 Colon cancer rs12940014 C T 0.02257 0.37237 0.0253 Rectal cancer rs12940014 C T 0.0185 0.56367 0.03205 |
| Gastroesophageal reflux | rs13010566 | A | C | 0.00618 | 0.5327 | 0.0099 | Esophageal carcinoma | rs13010566 | A | C | -0.0799 | 0.21058 | 0.06384 | Acute gastritis | rs13010566 | A | C | 0.02937 | 0.34723 | 0.03125 | Chronic gastritis | rs13010566 | A | C | 0.01018 | 0.51995 | 0.01582 | Gastroduodenal ulcer | rs13010566 | A | C | 0.00258 | 0.87325 | 0.01619 | Gastric cancer | rs13010566 | A | C | 0.01452 | 0.72421 | 0.04115 | Fatty liver | rs13010566 | A | C | 0.02714 | 0.40871 | 0.03286 | Hepatic fibrosis | rs13010566 | A | C | -0.1082 | 0.38811 | 0.12534 | Cirrhosis | rs13010566 | A | C | 0.01472 | 0.54332 | 0.02422liver cancer rs13010566 A C 0.0255 0.65063 0.0563 Choleliths rs13010566 A C 0.00638 0.4452 0.00836 Acute pancreatitis rs13010566 A C 0.02195 0.26084 0.01952 Chronic pancreatitis rs13010566 A C 0.04733 0.07207 0.02632 Pancreatic cancer rs13010566 A C 0.01928 0.63607 0.04074 Ulcerative colitis rs13010566 A C 0.00922 0.6694 0.02161 Crohn's disease rs13010566 A C -0.0345 0.35509 0.03727 Irrtable bowel syndrome rs13010566 A C -0.0011 0.94363 0.01616 Acute appendicitis rs13010566 A C -0.0052 0.57301 0.0093 Colon cancer rs13010566 A C -0.008 0.75164 0.02535 Rectal cancer rs13010566 A C -0.0306 0.34098 0.03219 |
| Gastroesophageal reflux | rs13029509 | A | G | -0.0007 | 0.9416 | 0.00993 | Esophageal carcinoma | rs13029509 | A | G | 0.05348 | 0.40451 | 0.06416 | Acute gastritis | rs13029509 | A | G | -0.0076 | 0.80821 | 0.03136 | Chronic gastritis | rs13029509 | A | G | -0.0124 | 0.43343 | 0.01585 | Gastroduodenal ulcer | rs13029509 | A | G | -0.0144 | 0.37414 | 0.01625 | Gastric cancer | rs13029509 | A | G | -0.0296 | 0.47453 | 0.04134 | Fatty liver | rs13029509 | A | G | -0.012 | 0.71506 | 0.03298 | Hepatic fibrosis | rs13029509 | A | G | -0.0382 | 0.7612 | 0.12574 | Cirrhosis | rs13029509 | A | G | -0.0122 | 0.61532 | 0.02426liver cancer rs13029509 A G -0.0582 0.30329 0.05657 Choleliths rs13029509 A G 0.01284 0.12562 0.00838 Acute pancreatitis rs13029509 A G -0.0053 0.78581 0.01957 Chronic pancreatitis rs13029509 A G -0.0077 0.7707 0.02638 Pancreatic cancer rs13029509 A G -0.0633 0.12067 0.04081 Ulcerative colitis rs13029509 A G 0.00963 0.6573 0.0217 Crohn's disease rs13029509 A G 0.0079 0.8327 0.03739 Irrtable bowel syndrome rs13029509 A G 0.02317 0.15311 0.01622 Acute appendicitis rs13029509 A G 0.00267 0.77518 0.00933 Colon cancer rs13029509 A G 0.05859 0.02125 0.02544 Rectal cancer rs13029509 A G -0.0089 0.78321 0.03231 |
| Gastroesophageal reflux | rs13090388 | C | T | 0.0086 | 0.38647 | 0.00993 | Esophageal carcinoma | rs13090388 | C | T | 0.05445 | 0.39515 | 0.06404 | Acute gastritis | rs13090388 | C | T | -0.0585 | 0.06208 | 0.03133 | Chronic gastritis | rs13090388 | C | T | 0.01061 | 0.5035 | 0.01587 | Gastroduodenal ulcer | rs13090388 | C | T | -0.0074 | 0.64656 | 0.01622 | Gastric cancer | rs13090388 | C | T | 0.04212 | 0.30612 | 0.04116 | Fatty liver | rs13090388 | C | T | -0.019 | 0.56418 | 0.03298 | Hepatic fibrosis | rs13090388 | C | T | 0.06946 | 0.5808 | 0.12579 | Cirrhosis | rs13090388 | C | T | 0.00486 | 0.84103 | 0.02425liver cancer rs13090388 C T -0.1187 0.03539 0.05641 Choleliths rs13090388 C T 0.00344 0.68242 0.0084 Acute pancreatitis rs13090388 C T -0.013 0.50438 0.01954 Chronic pancreatitis rs13090388 C T 0.00264 0.92004 0.02633 Pancreatic cancer rs13090388 C T 0.01987 0.62606 0.04077 Ulcerative colitis rs13090388 C T -0.1747 3E-16 0.02137 Crohn's disease rs13090388 C T -0.1349 0.00025 0.03686 Irrtable bowel syndrome rs13090388 C T 0.0246 0.1298 0.01624 Acute appendicitis rs13090388 C T 0.03244 0.0005 0.00932 Colon cancer rs13090388 C T -0.0283 0.26466 0.02541 Rectal cancer rs13090388 C T 0.0233 0.47065 0.0323 |
| Gastroesophageal reflux | rs13130765 | C | G | 0.0079 | 0.42548 | 0.00992 | Esophageal carcinoma | rs13130765 | C | G | -0.073 | 0.25455 | 0.06403 | Acute gastritis | rs13130765 | C | G | 0.0177 | 0.57165 | 0.0313 | Chronic gastritis | rs13130765 | C | G | 0.00426 | 0.78779 | 0.01584 | Gastroduodenal ulcer | rs13130765 | C | G | 0.01349 | 0.4052 | 0.01621 | Gastric cancer | rs13130765 | C | G | -0.0004 | 0.99185 | 0.04121 | Fatty liver | rs13130765 | C | G | 0.01658 | 0.61467 | 0.03293 | Hepatic fibrosis | rs13130765 | C | G | -0.1121 | 0.37324 | 0.12586 | Cirrhosis | rs13130765 | C | G | -0.0186 | 0.44202 | 0.02425liver cancer rs13130765 C G 0.01912 0.73541 0.05657 Choleliths rs13130765 C G 0.01569 0.06119 0.00838 Acute pancreatitis rs13130765 C G 0.05564 0.00444 0.01956 Chronic pancreatitis rs13130765 C G 0.0423 0.10861 0.02636 Pancreatic cancer rs13130765 C G 0.00434 0.9153 0.04081 Ulcerative colitis rs13130765 C G 0.03779 0.08089 0.02165 Crohn's disease rs13130765 C G -0.0181 0.62702 0.03731 Irrtable bowel syndrome rs13130765 C G -0.013 0.42373 0.01621 Acute appendicitis rs13130765 C G 0.00186 0.84142 0.00931 Colon cancer rs13130765 C G 0.0131 0.60586 0.02539 Rectal cancer rs13130765 C G -0.0246 0.4451 0.03224 |
| Gastroesophageal reflux | rs13141210 | C | T | 0.01978 | 0.04411 | 0.00983 | Esophageal carcinoma | rs13141210 | C | T | 0.03747 | 0.55604 | 0.06365 | Acute gastritis | rs13141210 | C | T | -0.0397 | 0.2002 | 0.03102 | Chronic gastritis | rs13141210 | C | T | 0.00793 | 0.61374 | 0.01572 | Gastroduodenal ulcer | rs13141210 | C | T | 0.00054 | 0.97338 | 0.01606 | Gastric cancer | rs13141210 | C | T | -0.0555 | 0.17436 | 0.04088 | Fatty liver | rs13141210 | C | T | 0.0095 | 0.77091 | 0.03261 | Hepatic fibrosis | rs13141210 | C | T | -0.051 | 0.68129 | 0.12422 | Cirrhosis | rs13141210 | C | T | -0.031 | 0.19707 | 0.024liver cancer rs13141210 C T 0.03752 0.50284 0.056 Choleliths rs13141210 C T 0.00094 0.91032 0.00831 Acute pancreatitis rs13141210 C T 0.02841 0.14224 0.01936 Chronic pancreatitis rs13141210 C T -0.0053 0.83891 0.02607 Pancreatic cancer rs13141210 C T 0.02089 0.6054 0.04044 Ulcerative colitis rs13141210 C T -0.0071 0.73878 0.02144 Crohn's disease rs13141210 C T 0.01621 0.66108 0.03697 Irrtable bowel syndrome rs13141210 C T 0.01908 0.23428 0.01605 Acute appendicitis rs13141210 C T -0.0046 0.6194 0.00922 Colon cancer rs13141210 C T -0.0266 0.29101 0.02524 Rectal cancer rs13141210 C T -0.0148 0.64271 0.03199 |
| Gastroesophageal reflux | rs13145650 | C | T | 0.0355 | 0.04744 | 0.01791 | Esophageal carcinoma | rs13145650 | C | T | 0.1089 | 0.34572 | 0.1155 | Acute gastritis | rs13145650 | C | T | 0.05791 | 0.30257 | 0.05617 | Chronic gastritis | rs13145650 | C | T | -0.0415 | 0.1477 | 0.02868 | Gastroduodenal ulcer | rs13145650 | C | T | 0.00582 | 0.8421 | 0.02924 | Gastric cancer | rs13145650 | C | T | 0.05295 | 0.47399 | 0.07396 | Fatty liver | rs13145650 | C | T | -0.0387 | 0.51419 | 0.05934 | Hepatic fibrosis | rs13145650 | C | T | -0.3437 | 0.13033 | 0.22719 | Cirrhosis | rs13145650 | C | T | 0.02674 | 0.54057 | 0.0437liver cancer rs13145650 C T -0.0527 0.60555 0.10202 Choleliths rs13145650 C T -0.0019 0.90019 0.01511 Acute pancreatitis rs13145650 C T 0.05777 0.10069 0.03519 Chronic pancreatitis rs13145650 C T 0.06597 0.16486 0.0475 Pancreatic cancer rs13145650 C T 0.03536 0.63042 0.07349 Ulcerative colitis rs13145650 C T 0.06713 0.0854 0.03902 Crohn's disease rs13145650 C T 0.00611 0.92784 0.06747 Irrtable bowel syndrome rs13145650 C T 0.02288 0.4341 0.02925 Acute appendicitis rs13145650 C T 0.02479 0.13926 0.01677 Colon cancer rs13145650 C T -0.0036 0.93687 0.04594 Rectal cancer rs13145650 C T 0.0225 0.6988 0.05815 |
| Gastroesophageal reflux | rs1334297 | A | G | 0.00194 | 0.86012 | 0.01103 | Esophageal carcinoma | rs1334297 | A | G | 0.08454 | 0.23652 | 0.07142 | Acute gastritis | rs1334297 | A | G | -0.0238 | 0.4933 | 0.03477 | Chronic gastritis | rs1334297 | A | G | -0.0013 | 0.93989 | 0.01768 | Gastroduodenal ulcer | rs1334297 | A | G | -0.0254 | 0.15887 | 0.01804 | Gastric cancer | rs1334297 | A | G | 0.01691 | 0.71192 | 0.04579 | Fatty liver | rs1334297 | A | G | 0.0487 | 0.18374 | 0.03663 | Hepatic fibrosis | rs1334297 | A | G | 0.03124 | 0.82335 | 0.13994 | Cirrhosis | rs1334297 | A | G | -0.0072 | 0.78899 | 0.02707liver cancer rs1334297 A G 0.0896 0.15626 0.0632 Choleliths rs1334297 A G -0.0128 0.17192 0.00934 Acute pancreatitis rs1334297 A G 0.00262 0.90425 0.02179 Chronic pancreatitis rs1334297 A G 0.01953 0.50638 0.02939 Pancreatic cancer rs1334297 A G 0.00828 0.85554 0.04548 Ulcerative colitis rs1334297 A G 0.04011 0.09599 0.02409 Crohn's disease rs1334297 A G -0.0242 0.56064 0.04157 Irrtable bowel syndrome rs1334297 A G -0.0129 0.47388 0.01802 Acute appendicitis rs1334297 A G 0.00318 0.75859 0.01036 Colon cancer rs1334297 A G 0.01789 0.52798 0.02836 Rectal cancer rs1334297 A G -0.007 0.84538 0.03597 |
| Gastroesophageal reflux | rs13422673 | C | T | 0.00692 | 0.48489 | 0.00991 | Esophageal carcinoma | rs13422673 | C | T | 0.04155 | 0.51888 | 0.06442 | Acute gastritis | rs13422673 | C | T | 0.01453 | 0.64212 | 0.03126 | Chronic gastritis | rs13422673 | C | T | -0.03 | 0.05838 | 0.01585 | Gastroduodenal ulcer | rs13422673 | C | T | -0.029 | 0.07376 | 0.01619 | Gastric cancer | rs13422673 | C | T | -0.0269 | 0.51503 | 0.04126 | Fatty liver | rs13422673 | C | T | -0.0354 | 0.28172 | 0.03293 | Hepatic fibrosis | rs13422673 | C | T | -0.1572 | 0.21227 | 0.12606 | Cirrhosis | rs13422673 | C | T | 0.02297 | 0.34367 | 0.02426liver cancer rs13422673 C T 0.05197 0.35982 0.05676 Choleliths rs13422673 C T 0.01005 0.22973 0.00837 Acute pancreatitis rs13422673 C T -0.012 0.54013 0.01953 Chronic pancreatitis rs13422673 C T -0.0221 0.4024 0.02637 Pancreatic cancer rs13422673 C T 0.00472 0.90815 0.04088 Ulcerative colitis rs13422673 C T -0.0475 0.02805 0.02164 Crohn's disease rs13422673 C T -0.0105 0.77875 0.03735 Irrtable bowel syndrome rs13422673 C T 0.00128 0.93695 0.01619 Acute appendicitis rs13422673 C T -0.0099 0.28562 0.00931 Colon cancer rs13422673 C T 0.00816 0.74836 0.02543 Rectal cancer rs13422673 C T -0.0059 0.85414 0.0323 |
| Gastroesophageal reflux | rs13428598 | T | C | -0.0251 | 0.01545 | 0.01037 | Esophageal carcinoma | rs13428598 | T | C | 0.01089 | 0.87122 | 0.06716 | Acute gastritis | rs13428598 | T | C | -0.0398 | 0.22383 | 0.03271 | Chronic gastritis | rs13428598 | T | C | -0.0118 | 0.47596 | 0.01655 | Gastroduodenal ulcer | rs13428598 | T | C | -0.0142 | 0.40214 | 0.01694 | Gastric cancer | rs13428598 | T | C | 0.03131 | 0.46908 | 0.04324 | Fatty liver | rs13428598 | T | C | -0.0285 | 0.40915 | 0.03448 | Hepatic fibrosis | rs13428598 | T | C | -0.268 | 0.04056 | 0.13085 | Cirrhosis | rs13428598 | T | C | -0.0607 | 0.01678 | 0.02536liver cancer rs13428598 T C 0.04785 0.41907 0.05922 Choleliths rs13428598 T C -0.0128 0.14404 0.00876 Acute pancreatitis rs13428598 T C -0.0275 0.1783 0.02044 Chronic pancreatitis rs13428598 T C -0.0364 0.18685 0.02754 Pancreatic cancer rs13428598 T C -0.0621 0.14582 0.04267 Ulcerative colitis rs13428598 T C 0.02618 0.24723 0.02263 Crohn's disease rs13428598 T C -0.0135 0.72916 0.03905 Irrtable bowel syndrome rs13428598 T C -0.0074 0.66396 0.01694 Acute appendicitis rs13428598 T C -0.01 0.30657 0.00974 Colon cancer rs13428598 T C -0.0071 0.78872 0.02663 Rectal cancer rs13428598 T C 0.01081 0.74875 0.03374 |
| Gastroesophageal reflux | rs1363862 | G | A | -0.0082 | 0.49009 | 0.01186 | Esophageal carcinoma | rs1363862 | G | A | 0.06442 | 0.40209 | 0.07688 | Acute gastritis | rs1363862 | G | A | -0.0472 | 0.20765 | 0.03745 | Chronic gastritis | rs1363862 | G | A | 0.01273 | 0.50212 | 0.01897 | Gastroduodenal ulcer | rs1363862 | G | A | 0.02445 | 0.20814 | 0.01942 | Gastric cancer | rs1363862 | G | A | -0.0066 | 0.89382 | 0.04963 | Fatty liver | rs1363862 | G | A | 0.05378 | 0.1722 | 0.03939 | Hepatic fibrosis | rs1363862 | G | A | 0.08848 | 0.55646 | 0.15045 | Cirrhosis | rs1363862 | G | A | 0.08198 | 0.0053 | 0.0294liver cancer rs1363862 G A 0.02954 0.66384 0.06797 Choleliths rs1363862 G A -0.0066 0.50841 0.01003 Acute pancreatitis rs1363862 G A -0.0238 0.30852 0.02339 Chronic pancreatitis rs1363862 G A 0.01691 0.59198 0.03155 Pancreatic cancer rs1363862 G A 0.00452 0.92635 0.04895 Ulcerative colitis rs1363862 G A 0.01891 0.46456 0.02586 Crohn's disease rs1363862 G A 0.01762 0.6924 0.04455 Irrtable bowel syndrome rs1363862 G A 0.00868 0.6539 0.01936 Acute appendicitis rs1363862 G A 0.00249 0.82299 0.01114 Colon cancer rs1363862 G A 0.0159 0.60345 0.03061 Rectal cancer rs1363862 G A 0.04603 0.23546 0.0388 |
| Gastroesophageal reflux | rs1381247 | C | T | 0.00381 | 0.69939 | 0.00987 | Esophageal carcinoma | rs1381247 | C | T | -0.0936 | 0.14293 | 0.06392 | Acute gastritis | rs1381247 | C | T | -0.023 | 0.45998 | 0.03115 | Chronic gastritis | rs1381247 | C | T | 0.00675 | 0.66927 | 0.01579 | Gastroduodenal ulcer | rs1381247 | C | T | 0.01053 | 0.51415 | 0.01613 | Gastric cancer | rs1381247 | C | T | -0.0316 | 0.4423 | 0.0411 | Fatty liver | rs1381247 | C | T | -0.0071 | 0.82926 | 0.03276 | Hepatic fibrosis | rs1381247 | C | T | 0.06397 | 0.60848 | 0.12489 | Cirrhosis | rs1381247 | C | T | 0.03668 | 0.12844 | 0.02413liver cancer rs1381247 C T 0.02321 0.68048 0.05636 Choleliths rs1381247 C T 0.00333 0.68994 0.00835 Acute pancreatitis rs1381247 C T 0.00115 0.95275 0.01946 Chronic pancreatitis rs1381247 C T 0.04301 0.10086 0.02621 Pancreatic cancer rs1381247 C T -0.0313 0.44214 0.04071 Ulcerative colitis rs1381247 C T -0.0142 0.50871 0.02154 Crohn's disease rs1381247 C T -0.0767 0.03888 0.03715 Irrtable bowel syndrome rs1381247 C T 0.02089 0.19487 0.01612 Acute appendicitis rs1381247 C T -0.0006 0.94649 0.00927 Colon cancer rs1381247 C T -0.0027 0.91391 0.02535 Rectal cancer rs1381247 C T -0.0165 0.6083 0.03216 |
| Gastroesophageal reflux | rs1391438 | T | C | -0.0007 | 0.94306 | 0.01034 | Esophageal carcinoma | rs1391438 | T | C | -0.0146 | 0.82691 | 0.06697 | Acute gastritis | rs1391438 | T | C | 0.05822 | 0.07418 | 0.03261 | Chronic gastritis | rs1391438 | T | C | -0.0156 | 0.34685 | 0.01655 | Gastroduodenal ulcer | rs1391438 | T | C | -0.0005 | 0.97667 | 0.01691 | Gastric cancer | rs1391438 | T | C | 0.02473 | 0.56641 | 0.04313 | Fatty liver | rs1391438 | T | C | 0.00897 | 0.79382 | 0.03431 | Hepatic fibrosis | rs1391438 | T | C | -0.1655 | 0.20541 | 0.1307 | Cirrhosis | rs1391438 | T | C | -0.0395 | 0.11847 | 0.02527liver cancer rs1391438 T C 0.02978 0.61427 0.05909 Choleliths rs1391438 T C -0.0109 0.21282 0.00874 Acute pancreatitis rs1391438 T C -0.0179 0.37984 0.02036 Chronic pancreatitis rs1391438 T C -0.0371 0.1763 0.02744 Pancreatic cancer rs1391438 T C -0.0098 0.81851 0.04257 Ulcerative colitis rs1391438 T C -0.0227 0.31377 0.02255 Crohn's disease rs1391438 T C -0.0165 0.67082 0.03891 Irrtable bowel syndrome rs1391438 T C 0.00723 0.6687 0.0169 Acute appendicitis rs1391438 T C -0.0032 0.74307 0.0097 Colon cancer rs1391438 T C -0.0265 0.31786 0.02658 Rectal cancer rs1391438 T C -0.0235 0.4866 0.03382 |
| Gastroesophageal reflux | rs1427298 | C | T | -0.0038 | 0.70438 | 0.00999 | Esophageal carcinoma | rs1427298 | C | T | 0.05855 | 0.36475 | 0.0646 | Acute gastritis | rs1427298 | C | T | -0.0073 | 0.81566 | 0.0315 | Chronic gastritis | rs1427298 | C | T | 0.01216 | 0.44619 | 0.01596 | Gastroduodenal ulcer | rs1427298 | C | T | 0.03065 | 0.06039 | 0.01632 | Gastric cancer | rs1427298 | C | T | 0.06849 | 0.09963 | 0.04159 | Fatty liver | rs1427298 | C | T | -0.0115 | 0.72915 | 0.03318 | Hepatic fibrosis | rs1427298 | C | T | -0.0161 | 0.89885 | 0.12639 | Cirrhosis | rs1427298 | C | T | 0.04729 | 0.05292 | 0.02443liver cancer rs1427298 C T 0.00334 0.95313 0.05679 Choleliths rs1427298 C T 0.00088 0.91728 0.00844 Acute pancreatitis rs1427298 C T 0.01738 0.37758 0.0197 Chronic pancreatitis rs1427298 C T -0.0041 0.87726 0.02658 Pancreatic cancer rs1427298 C T -0.0155 0.70561 0.04109 Ulcerative colitis rs1427298 C T -0.0049 0.82397 0.02181 Crohn's disease rs1427298 C T 0.00989 0.79265 0.03764 Irrtable bowel syndrome rs1427298 C T -0.0273 0.09514 0.01633 Acute appendicitis rs1427298 C T -0.0174 0.06333 0.00938 Colon cancer rs1427298 C T 0.01442 0.57318 0.0256 Rectal cancer rs1427298 C T 0.02288 0.4811 0.03248 |
| Gastroesophageal reflux | rs1450782 | G | T | 0.01112 | 0.25743 | 0.00982 | Esophageal carcinoma | rs1450782 | G | T | -0.0696 | 0.27327 | 0.06349 | Acute gastritis | rs1450782 | G | T | 0.02465 | 0.42621 | 0.03097 | Chronic gastritis | rs1450782 | G | T | -0.0044 | 0.777 | 0.01568 | Gastroduodenal ulcer | rs1450782 | G | T | 0.02047 | 0.20233 | 0.01605 | Gastric cancer | rs1450782 | G | T | -0.041 | 0.31527 | 0.04084 | Fatty liver | rs1450782 | G | T | 0.00959 | 0.7682 | 0.03255 | Hepatic fibrosis | rs1450782 | G | T | -0.1779 | 0.15199 | 0.12416 | Cirrhosis | rs1450782 | G | T | 0.00385 | 0.87242 | 0.024liver cancer rs1450782 G T 0.03953 0.48156 0.05616 Choleliths rs1450782 G T -0.0009 0.91105 0.0083 Acute pancreatitis rs1450782 G T 0.00817 0.67259 0.01933 Chronic pancreatitis rs1450782 G T -0.0296 0.25585 0.02605 Pancreatic cancer rs1450782 G T -0.0118 0.77088 0.04043 Ulcerative colitis rs1450782 G T 0.00736 0.73071 0.02139 Crohn's disease rs1450782 G T 0.00227 0.95091 0.03692 Irrtable bowel syndrome rs1450782 G T 0.00509 0.75088 0.01603 Acute appendicitis rs1450782 G T -0.0015 0.86725 0.00921 Colon cancer rs1450782 G T -0.0482 0.05579 0.0252 Rectal cancer rs1450782 G T 0.05248 0.1007 0.03197 |
| Gastroesophageal reflux | rs1455350 | T | A | -0.0035 | 0.72394 | 0.00985 | Esophageal carcinoma | rs1455350 | T | A | 0.00906 | 0.88703 | 0.06374 | Acute gastritis | rs1455350 | T | A | -0.0168 | 0.58927 | 0.03108 | Chronic gastritis | rs1455350 | T | A | 0.00172 | 0.91284 | 0.01573 | Gastroduodenal ulcer | rs1455350 | T | A | -0.023 | 0.15358 | 0.0161 | Gastric cancer | rs1455350 | T | A | 0.01738 | 0.67105 | 0.04091 | Fatty liver | rs1455350 | T | A | -0.0022 | 0.94601 | 0.0327 | Hepatic fibrosis | rs1455350 | T | A | -0.0328 | 0.79306 | 0.12516 | Cirrhosis | rs1455350 | T | A | 0.01678 | 0.48641 | 0.02411liver cancer rs1455350 T A -0.0364 0.51606 0.05612 Choleliths rs1455350 T A -0.0136 0.10306 0.00832 Acute pancreatitis rs1455350 T A 0.00386 0.84258 0.01943 Chronic pancreatitis rs1455350 T A 0.00057 0.98279 0.0262 Pancreatic cancer rs1455350 T A -0.0676 0.09576 0.04057 Ulcerative colitis rs1455350 T A -0.0939 1.3E-05 0.02152 Crohn's disease rs1455350 T A -0.07 0.0592 0.03713 Irrtable bowel syndrome rs1455350 T A 0.01179 0.46397 0.0161 Acute appendicitis rs1455350 T A 0.01066 0.24938 0.00926 Colon cancer rs1455350 T A -0.045 0.07492 0.02526 Rectal cancer rs1455350 T A -0.0344 0.28379 0.03207 |
| Gastroesophageal reflux | rs152603 | G | A | -0.0033 | 0.76902 | 0.01128 | Esophageal carcinoma | rs152603 | G | A | -0.0472 | 0.51846 | 0.07305 | Acute gastritis | rs152603 | G | A | -0.0206 | 0.56377 | 0.03571 | Chronic gastritis | rs152603 | G | A | 0.0305 | 0.09054 | 0.01802 | Gastroduodenal ulcer | rs152603 | G | A | -0.0115 | 0.53504 | 0.0185 | Gastric cancer | rs152603 | G | A | 0.06755 | 0.15025 | 0.04695 | Fatty liver | rs152603 | G | A | -0.0072 | 0.84825 | 0.0375 | Hepatic fibrosis | rs152603 | G | A | 0.17683 | 0.21839 | 0.14367 | Cirrhosis | rs152603 | G | A | -0.0469 | 0.09046 | 0.02772liver cancer rs152603 G A -0.0374 0.56259 0.06458 Choleliths rs152603 G A 0.00556 0.56088 0.00956 Acute pancreatitis rs152603 G A -0.0094 0.67339 0.02234 Chronic pancreatitis rs152603 G A -0.0448 0.13772 0.03015 Pancreatic cancer rs152603 G A -0.0021 0.96446 0.04666 Ulcerative colitis rs152603 G A -0.0223 0.36691 0.02468 Crohn's disease rs152603 G A -0.0455 0.28534 0.04259 Irrtable bowel syndrome rs152603 G A -0.024 0.19299 0.01847 Acute appendicitis rs152603 G A -0.0022 0.83532 0.01064 Colon cancer rs152603 G A 0.0195 0.50041 0.02894 Rectal cancer rs152603 G A -0.0219 0.55084 0.03676 |
| Gastroesophageal reflux | rs1558727 | C | T | 0.01606 | 0.11111 | 0.01008 | Esophageal carcinoma | rs1558727 | C | T | 0.0069 | 0.91572 | 0.0652 | Acute gastritis | rs1558727 | C | T | -0.0369 | 0.24599 | 0.03184 | Chronic gastritis | rs1558727 | C | T | 0.01854 | 0.24981 | 0.01611 | Gastroduodenal ulcer | rs1558727 | C | T | -0.0007 | 0.96761 | 0.01649 | Gastric cancer | rs1558727 | C | T | -0.002 | 0.96163 | 0.04193 | Fatty liver | rs1558727 | C | T | -0.0253 | 0.44966 | 0.03347 | Hepatic fibrosis | rs1558727 | C | T | -0.0113 | 0.92943 | 0.12768 | Cirrhosis | rs1558727 | C | T | 0.04152 | 0.09238 | 0.02467liver cancer rs1558727 C T 0.05469 0.34205 0.05756 Choleliths rs1558727 C T 0.00135 0.87389 0.00852 Acute pancreatitis rs1558727 C T -0.0124 0.53182 0.01988 Chronic pancreatitis rs1558727 C T -0.0004 0.9892 0.02682 Pancreatic cancer rs1558727 C T 0.00834 0.84077 0.04151 Ulcerative colitis rs1558727 C T 0.00787 0.72049 0.02201 Crohn's disease rs1558727 C T -0.0145 0.70255 0.03792 Irrtable bowel syndrome rs1558727 C T 0.00337 0.83757 0.01646 Acute appendicitis rs1558727 C T 0.00453 0.63256 0.00947 Colon cancer rs1558727 C T 0.02663 0.30293 0.02585 Rectal cancer rs1558727 C T 0.02898 0.37661 0.03278 |
| Gastroesophageal reflux | rs1566085 | T | G | -0.001 | 0.91533 | 0.00981 | Esophageal carcinoma | rs1566085 | T | G | 0.05594 | 0.37913 | 0.0636 | Acute gastritis | rs1566085 | T | G | 0.00676 | 0.82717 | 0.03094 | Chronic gastritis | rs1566085 | T | G | 0.00735 | 0.63923 | 0.01567 | Gastroduodenal ulcer | rs1566085 | T | G | 0.01681 | 0.29419 | 0.01603 | Gastric cancer | rs1566085 | T | G | -0.0171 | 0.67487 | 0.04083 | Fatty liver | rs1566085 | T | G | 0.02724 | 0.4028 | 0.03256 | Hepatic fibrosis | rs1566085 | T | G | -0.1687 | 0.17454 | 0.12424 | Cirrhosis | rs1566085 | T | G | -0.0056 | 0.81597 | 0.02399liver cancer rs1566085 T G 0.01123 0.84128 0.05606 Choleliths rs1566085 T G -0.0149 0.07238 0.0083 Acute pancreatitis rs1566085 T G 0.03372 0.08125 0.01934 Chronic pancreatitis rs1566085 T G 0.01728 0.50723 0.02606 Pancreatic cancer rs1566085 T G -0.0145 0.71891 0.04033 Ulcerative colitis rs1566085 T G -0.0204 0.33974 0.02141 Crohn's disease rs1566085 T G -0.0379 0.30488 0.03689 Irrtable bowel syndrome rs1566085 T G -0.0193 0.22755 0.01602 Acute appendicitis rs1566085 T G 0.00068 0.94158 0.00921 Colon cancer rs1566085 T G 0.01126 0.65476 0.02519 Rectal cancer rs1566085 T G 0.01693 0.59633 0.03196 |
| Gastroesophageal reflux | rs1569092 | A | G | 0.01732 | 0.25494 | 0.01522 | Esophageal carcinoma | rs1569092 | A | G | 0.0567 | 0.57038 | 0.09992 | Acute gastritis | rs1569092 | A | G | 0.06266 | 0.1924 | 0.04807 | Chronic gastritis | rs1569092 | A | G | -0.0267 | 0.27265 | 0.02435 | Gastroduodenal ulcer | rs1569092 | A | G | 0.02248 | 0.3683 | 0.02499 | Gastric cancer | rs1569092 | A | G | 0.0635 | 0.32162 | 0.06407 | Fatty liver | rs1569092 | A | G | 0.07008 | 0.16693 | 0.0507 | Hepatic fibrosis | rs1569092 | A | G | -0.2781 | 0.14956 | 0.19301 | Cirrhosis | rs1569092 | A | G | -0.0124 | 0.73933 | 0.03738liver cancer rs1569092 A G 0.07371 0.40005 0.08759 Choleliths rs1569092 A G 0.00876 0.49693 0.0129 Acute pancreatitis rs1569092 A G -0.0007 0.9819 0.03016 Chronic pancreatitis rs1569092 A G -0.0041 0.92049 0.04074 Pancreatic cancer rs1569092 A G 0.06162 0.33017 0.06328 Ulcerative colitis rs1569092 A G 0.01727 0.60423 0.03331 Crohn's disease rs1569092 A G -0.0097 0.86637 0.05747 Irrtable bowel syndrome rs1569092 A G 0.01066 0.66813 0.02487 Acute appendicitis rs1569092 A G -0.0014 0.923 0.01433 Colon cancer rs1569092 A G -0.0085 0.8287 0.03946 Rectal cancer rs1569092 A G 0.05556 0.26668 0.05002 |
| Gastroesophageal reflux | rs1582173 | C | T | 0.01198 | 0.29922 | 0.01154 | Esophageal carcinoma | rs1582173 | C | T | -0.0989 | 0.18739 | 0.07499 | Acute gastritis | rs1582173 | C | T | -0.0084 | 0.81685 | 0.03638 | Chronic gastritis | rs1582173 | C | T | -0.0186 | 0.31348 | 0.01842 | Gastroduodenal ulcer | rs1582173 | C | T | 0.00669 | 0.7228 | 0.01886 | Gastric cancer | rs1582173 | C | T | 0.04123 | 0.38927 | 0.04789 | Fatty liver | rs1582173 | C | T | -0.0274 | 0.47485 | 0.03835 | Hepatic fibrosis | rs1582173 | C | T | 0.02307 | 0.87512 | 0.14678 | Cirrhosis | rs1582173 | C | T | -0.0029 | 0.9197 | 0.02833liver cancer rs1582173 C T -0.053 0.42071 0.06584 Choleliths rs1582173 C T 0.00929 0.34198 0.00977 Acute pancreatitis rs1582173 C T -0.0175 0.4434 0.02283 Chronic pancreatitis rs1582173 C T -0.0307 0.3188 0.03083 Pancreatic cancer rs1582173 C T -0.0094 0.84418 0.04765 Ulcerative colitis rs1582173 C T -0.0003 0.99094 0.02525 Crohn's disease rs1582173 C T -0.0126 0.77239 0.04349 Irrtable bowel syndrome rs1582173 C T -0.0082 0.66495 0.01886 Acute appendicitis rs1582173 C T -0.0138 0.20435 0.01087 Colon cancer rs1582173 C T 0.02352 0.42639 0.02958 Rectal cancer rs1582173 C T 0.0252 0.50145 0.03748 |
| Gastroesophageal reflux | rs1584469 | T | C | 0.01057 | 0.303 | 0.01026 | Esophageal carcinoma | rs1584469 | T | C | 0.0899 | 0.17608 | 0.06645 | Acute gastritis | rs1584469 | T | C | -0.0245 | 0.44934 | 0.03238 | Chronic gastritis | rs1584469 | T | C | 0.00707 | 0.66646 | 0.01639 | Gastroduodenal ulcer | rs1584469 | T | C | 0.01009 | 0.54687 | 0.01675 | Gastric cancer | rs1584469 | T | C | 0.03349 | 0.43305 | 0.04272 | Fatty liver | rs1584469 | T | C | 0.02178 | 0.52288 | 0.03409 | Hepatic fibrosis | rs1584469 | T | C | 0.11785 | 0.36385 | 0.12978 | Cirrhosis | rs1584469 | T | C | -0.0098 | 0.69542 | 0.02504liver cancer rs1584469 T C -0.0551 0.34541 0.05844 Choleliths rs1584469 T C -0.0075 0.39014 0.00867 Acute pancreatitis rs1584469 T C 0.00044 0.9828 0.02018 Chronic pancreatitis rs1584469 T C -0.0023 0.93339 0.02721 Pancreatic cancer rs1584469 T C -0.0378 0.37072 0.04223 Ulcerative colitis rs1584469 T C 0.00318 0.88734 0.02241 Crohn's disease rs1584469 T C -0.0119 0.75805 0.03866 Irrtable bowel syndrome rs1584469 T C 0.003 0.85809 0.01678 Acute appendicitis rs1584469 T C 0.01264 0.18912 0.00963 Colon cancer rs1584469 T C 0.0017 0.94861 0.02632 Rectal cancer rs1584469 T C -0.0156 0.64118 0.03344 |
| Gastroesophageal reflux | rs1592757 | C | G | 0.00892 | 0.38658 | 0.01031 | Esophageal carcinoma | rs1592757 | C | G | 0.05828 | 0.38174 | 0.06663 | Acute gastritis | rs1592757 | C | G | -0.0042 | 0.89764 | 0.03256 | Chronic gastritis | rs1592757 | C | G | 0.00607 | 0.71283 | 0.01649 | Gastroduodenal ulcer | rs1592757 | C | G | 0.04766 | 0.0045 | 0.01678 | Gastric cancer | rs1592757 | C | G | 0.01214 | 0.77704 | 0.04286 | Fatty liver | rs1592757 | C | G | 0.04208 | 0.21836 | 0.03419 | Hepatic fibrosis | rs1592757 | C | G | -0.0565 | 0.66489 | 0.13048 | Cirrhosis | rs1592757 | C | G | 0.00446 | 0.85958 | 0.02518liver cancer rs1592757 C G 0.00842 0.88645 0.05895 Choleliths rs1592757 C G 0.00995 0.25355 0.00871 Acute pancreatitis rs1592757 C G 0.02831 0.16306 0.0203 Chronic pancreatitis rs1592757 C G 0.01552 0.57026 0.02735 Pancreatic cancer rs1592757 C G -0.0153 0.71733 0.04238 Ulcerative colitis rs1592757 C G 0.01939 0.38842 0.02248 Crohn's disease rs1592757 C G -0.037 0.34089 0.0388 Irrtable bowel syndrome rs1592757 C G 0.03448 0.04053 0.01684 Acute appendicitis rs1592757 C G 0.00914 0.34464 0.00967 Colon cancer rs1592757 C G -0.0645 0.01469 0.02642 Rectal cancer rs1592757 C G -0.0264 0.43155 0.03361 |
| Gastroesophageal reflux | rs1595973 | C | T | -0.0246 | 0.01494 | 0.01009 | Esophageal carcinoma | rs1595973 | C | T | 0.06353 | 0.32919 | 0.06511 | Acute gastritis | rs1595973 | C | T | -0.0067 | 0.83318 | 0.03188 | Chronic gastritis | rs1595973 | C | T | -0.0031 | 0.8458 | 0.01611 | Gastroduodenal ulcer | rs1595973 | C | T | -0.0254 | 0.12329 | 0.0165 | Gastric cancer | rs1595973 | C | T | 0.0191 | 0.64817 | 0.04185 | Fatty liver | rs1595973 | C | T | -0.0224 | 0.50487 | 0.03354 | Hepatic fibrosis | rs1595973 | C | T | -0.1002 | 0.4337 | 0.12799 | Cirrhosis | rs1595973 | C | T | -0.0164 | 0.50504 | 0.02467liver cancer rs1595973 C T -0.0644 0.26232 0.05743 Choleliths rs1595973 C T -0.0111 0.19309 0.00853 Acute pancreatitis rs1595973 C T -0.021 0.29159 0.01989 Chronic pancreatitis rs1595973 C T -0.0384 0.15199 0.02682 Pancreatic cancer rs1595973 C T 0.06092 0.14173 0.04146 Ulcerative colitis rs1595973 C T -0.0039 0.8602 0.02206 Crohn's disease rs1595973 C T 0.00808 0.8321 0.03809 Irrtable bowel syndrome rs1595973 C T -0.0124 0.45106 0.0165 Acute appendicitis rs1595973 C T -0.0172 0.06922 0.00949 Colon cancer rs1595973 C T 0.01892 0.46462 0.02587 Rectal cancer rs1595973 C T -0.0155 0.63634 0.03279 |
| Gastroesophageal reflux | rs1620977 | G | A | -0.0166 | 0.13478 | 0.01112 | Esophageal carcinoma | rs1620977 | G | A | 0.03373 | 0.6407 | 0.07227 | Acute gastritis | rs1620977 | G | A | -0.0134 | 0.7014 | 0.03504 | Chronic gastritis | rs1620977 | G | A | -0.028 | 0.11541 | 0.01779 | Gastroduodenal ulcer | rs1620977 | G | A | -0.0038 | 0.83561 | 0.01819 | Gastric cancer | rs1620977 | G | A | -0.0204 | 0.66022 | 0.04635 | Fatty liver | rs1620977 | G | A | 0.008 | 0.82839 | 0.03689 | Hepatic fibrosis | rs1620977 | G | A | 0.19455 | 0.16631 | 0.14055 | Cirrhosis | rs1620977 | G | A | 0.04097 | 0.13173 | 0.02718liver cancer rs1620977 G A -0.0064 0.91982 0.06372 Choleliths rs1620977 G A 0.0097 0.30202 0.0094 Acute pancreatitis rs1620977 G A 0.00761 0.72829 0.02189 Chronic pancreatitis rs1620977 G A 0.00999 0.73451 0.02946 Pancreatic cancer rs1620977 G A 0.03182 0.48817 0.04591 Ulcerative colitis rs1620977 G A 0.00391 0.87193 0.02425 Crohn's disease rs1620977 G A 0.00979 0.81514 0.04188 Irrtable bowel syndrome rs1620977 G A 0.00988 0.58647 0.01817 Acute appendicitis rs1620977 G A 0.00265 0.79977 0.01043 Colon cancer rs1620977 G A -0.0153 0.59214 0.02859 Rectal cancer rs1620977 G A -0.0061 0.86709 0.03625 |
| Gastroesophageal reflux | rs1671770 | C | A | 0.01483 | 0.27082 | 0.01347 | Esophageal carcinoma | rs1671770 | C | A | -0.0233 | 0.78949 | 0.08717 | Acute gastritis | rs1671770 | C | A | 0.07927 | 0.06316 | 0.04266 | Chronic gastritis | rs1671770 | C | A | 0.02525 | 0.24109 | 0.02154 | Gastroduodenal ulcer | rs1671770 | C | A | 0.03436 | 0.1205 | 0.02213 | Gastric cancer | rs1671770 | C | A | 0.03733 | 0.50565 | 0.05608 | Fatty liver | rs1671770 | C | A | 0.07516 | 0.09438 | 0.04493 | Hepatic fibrosis | rs1671770 | C | A | -0.2782 | 0.10773 | 0.17297 | Cirrhosis | rs1671770 | C | A | 0.03815 | 0.25008 | 0.03317liver cancer rs1671770 C A 0.14782 0.05669 0.07757 Choleliths rs1671770 C A -0.0032 0.77883 0.01142 Acute pancreatitis rs1671770 C A -0.0191 0.47543 0.0267 Chronic pancreatitis rs1671770 C A 0.01705 0.63639 0.03606 Pancreatic cancer rs1671770 C A 0.01472 0.7922 0.05588 Ulcerative colitis rs1671770 C A 0.01795 0.54398 0.02958 Crohn's disease rs1671770 C A -0.0303 0.5522 0.05098 Irrtable bowel syndrome rs1671770 C A -0.0276 0.21151 0.02208 Acute appendicitis rs1671770 C A -0.0335 0.00805 0.01265 Colon cancer rs1671770 C A -0.0108 0.75515 0.03448 Rectal cancer rs1671770 C A 0.0269 0.53925 0.04382 |
| Gastroesophageal reflux | rs16846463 | A | G | -0.0052 | 0.77131 | 0.01805 | Esophageal carcinoma | rs16846463 | A | G | -0.0044 | 0.96913 | 0.11493 | Acute gastritis | rs16846463 | A | G | 0.026 | 0.64706 | 0.05679 | Chronic gastritis | rs16846463 | A | G | -0.0113 | 0.696 | 0.02881 | Gastroduodenal ulcer | rs16846463 | A | G | -0.0016 | 0.95647 | 0.02957 | Gastric cancer | rs16846463 | A | G | 0.01101 | 0.88075 | 0.07339 | Fatty liver | rs16846463 | A | G | 0.09946 | 0.09843 | 0.06019 | Hepatic fibrosis | rs16846463 | A | G | -0.3139 | 0.18498 | 0.23677 | Cirrhosis | rs16846463 | A | G | -0.0287 | 0.52166 | 0.04474liver cancer rs16846463 A G 0.11154 0.27687 0.10258 Choleliths rs16846463 A G -0.0048 0.75448 0.01521 Acute pancreatitis rs16846463 A G -0.0218 0.54252 0.03587 Chronic pancreatitis rs16846463 A G -0.0476 0.32824 0.04869 Pancreatic cancer rs16846463 A G 0.02051 0.78248 0.0743 Ulcerative colitis rs16846463 A G 0.05813 0.14335 0.03972 Crohn's disease rs16846463 A G -0.0092 0.89353 0.06841 Irrtable bowel syndrome rs16846463 A G -0.0692 0.01908 0.02953 Acute appendicitis rs16846463 A G -0.0223 0.19082 0.01704 Colon cancer rs16846463 A G 0.10323 0.02381 0.04567 Rectal cancer rs16846463 A G 0.01339 0.81718 0.0579 |
| Gastroesophageal reflux | rs16854920 | T | C | -0.0084 | 0.4404 | 0.01083 | Esophageal carcinoma | rs16854920 | T | C | 0.22802 | 0.00129 | 0.07088 | Acute gastritis | rs16854920 | T | C | -0.0131 | 0.70092 | 0.03419 | Chronic gastritis | rs16854920 | T | C | 0.00744 | 0.66727 | 0.01731 | Gastroduodenal ulcer | rs16854920 | T | C | -0.0026 | 0.88259 | 0.01773 | Gastric cancer | rs16854920 | T | C | -0.0362 | 0.42176 | 0.045 | Fatty liver | rs16854920 | T | C | 0.00306 | 0.93245 | 0.03604 | Hepatic fibrosis | rs16854920 | T | C | 0.11154 | 0.42022 | 0.13838 | Cirrhosis | rs16854920 | T | C | -0.0139 | 0.60115 | 0.02659liver cancer rs16854920 T C 0.08185 0.18785 0.06215 Choleliths rs16854920 T C 0.00979 0.28492 0.00915 Acute pancreatitis rs16854920 T C -0.0072 0.73795 0.02142 Chronic pancreatitis rs16854920 T C -0.0214 0.45991 0.02891 Pancreatic cancer rs16854920 T C 0.01432 0.7492 0.04479 Ulcerative colitis rs16854920 T C 0.01299 0.58372 0.0237 Crohn's disease rs16854920 T C 0.02633 0.51925 0.04085 Irrtable bowel syndrome rs16854920 T C -0.0108 0.54083 0.01772 Acute appendicitis rs16854920 T C 0.01869 0.06673 0.0102 Colon cancer rs16854920 T C -0.0038 0.89171 0.02782 Rectal cancer rs16854920 T C -0.0319 0.36644 0.03531 |
| Gastroesophageal reflux | rs1689510 | G | C | 0.02146 | 0.04108 | 0.01051 | Esophageal carcinoma | rs1689510 | G | C | -0.0938 | 0.16727 | 0.06791 | Acute gastritis | rs1689510 | G | C | -0.0225 | 0.49767 | 0.03315 | Chronic gastritis | rs1689510 | G | C | -0.0092 | 0.58542 | 0.01679 | Gastroduodenal ulcer | rs1689510 | G | C | -0.0031 | 0.85853 | 0.01718 | Gastric cancer | rs1689510 | G | C | 0.01663 | 0.70286 | 0.0436 | Fatty liver | rs1689510 | G | C | 0.04068 | 0.24325 | 0.03486 | Hepatic fibrosis | rs1689510 | G | C | -0.0318 | 0.81152 | 0.13316 | Cirrhosis | rs1689510 | G | C | -0.0134 | 0.60141 | 0.0257liver cancer rs1689510 G C 0.0595 0.32019 0.05986 Choleliths rs1689510 G C 0.01729 0.05135 0.00887 Acute pancreatitis rs1689510 G C -0.004 0.84748 0.0207 Chronic pancreatitis rs1689510 G C -0.0156 0.577 0.02792 Pancreatic cancer rs1689510 G C 0.05989 0.16573 0.04321 Ulcerative colitis rs1689510 G C 0.02606 0.25611 0.02295 Crohn's disease rs1689510 G C 0.02447 0.53623 0.03956 Irrtable bowel syndrome rs1689510 G C -0.0018 0.91462 0.01716 Acute appendicitis rs1689510 G C 0.00877 0.37416 0.00987 Colon cancer rs1689510 G C -0.0177 0.50995 0.02687 Rectal cancer rs1689510 G C 0.04516 0.18526 0.03409 |
| Gastroesophageal reflux | rs16995054 | C | T | -0.0027 | 0.83277 | 0.01278 | Esophageal carcinoma | rs16995054 | C | T | -0.0113 | 0.89156 | 0.08253 | Acute gastritis | rs16995054 | C | T | 0.03406 | 0.39821 | 0.04032 | Chronic gastritis | rs16995054 | C | T | -0.0058 | 0.77448 | 0.02041 | Gastroduodenal ulcer | rs16995054 | C | T | -0.0099 | 0.6367 | 0.02093 | Gastric cancer | rs16995054 | C | T | 0.03166 | 0.55508 | 0.05364 | Fatty liver | rs16995054 | C | T | 0.01944 | 0.64699 | 0.04244 | Hepatic fibrosis | rs16995054 | C | T | -0.3004 | 0.06359 | 0.16191 | Cirrhosis | rs16995054 | C | T | -0.0483 | 0.12252 | 0.0313liver cancer rs16995054 C T -0.0414 0.57019 0.0729 Choleliths rs16995054 C T 0.02973 0.0062 0.01086 Acute pancreatitis rs16995054 C T 0.01436 0.5695 0.02524 Chronic pancreatitis rs16995054 C T 0.02328 0.49362 0.03401 Pancreatic cancer rs16995054 C T 0.0355 0.50044 0.05269 Ulcerative colitis rs16995054 C T -0.0244 0.38228 0.02798 Crohn's disease rs16995054 C T -0.0662 0.16878 0.04814 Irrtable bowel syndrome rs16995054 C T 0.01439 0.49106 0.02089 Acute appendicitis rs16995054 C T -0.0191 0.11264 0.01202 Colon cancer rs16995054 C T 0.01113 0.73459 0.03284 Rectal cancer rs16995054 C T -0.027 0.51637 0.04166 |
| Gastroesophageal reflux | rs17048855 | A | G | -0.0093 | 0.34973 | 0.00995 | Esophageal carcinoma | rs17048855 | A | G | 0.03773 | 0.55917 | 0.06459 | Acute gastritis | rs17048855 | A | G | -0.0262 | 0.40444 | 0.0314 | Chronic gastritis | rs17048855 | A | G | 0.00185 | 0.90729 | 0.0159 | Gastroduodenal ulcer | rs17048855 | A | G | -0.0127 | 0.43625 | 0.01628 | Gastric cancer | rs17048855 | A | G | 0.01361 | 0.74256 | 0.04144 | Fatty liver | rs17048855 | A | G | -0.0537 | 0.10404 | 0.03305 | Hepatic fibrosis | rs17048855 | A | G | -0.0985 | 0.43409 | 0.1259 | Cirrhosis | rs17048855 | A | G | -0.0364 | 0.13494 | 0.02434liver cancer rs17048855 A G -0.0053 0.92565 0.05697 Choleliths rs17048855 A G -0.0105 0.21303 0.00842 Acute pancreatitis rs17048855 A G -0.0123 0.53067 0.01963 Chronic pancreatitis rs17048855 A G -0.0528 0.0462 0.02648 Pancreatic cancer rs17048855 A G -0.1115 0.00646 0.04093 Ulcerative colitis rs17048855 A G 0.01522 0.4836 0.02172 Crohn's disease rs17048855 A G 0.02154 0.56555 0.03749 Irrtable bowel syndrome rs17048855 A G -0.0333 0.04075 0.01625 Acute appendicitis rs17048855 A G -0.0065 0.49006 0.00935 Colon cancer rs17048855 A G -0.0111 0.66514 0.02558 Rectal cancer rs17048855 A G 0.00857 0.79172 0.03246 |
| Gastroesophageal reflux | rs17110109 | C | T | -0.0075 | 0.44752 | 0.00993 | Esophageal carcinoma | rs17110109 | C | T | 0.03477 | 0.58912 | 0.06439 | Acute gastritis | rs17110109 | C | T | -0.0099 | 0.75088 | 0.0313 | Chronic gastritis | rs17110109 | C | T | -0.0034 | 0.82842 | 0.01587 | Gastroduodenal ulcer | rs17110109 | C | T | -0.0315 | 0.05229 | 0.01622 | Gastric cancer | rs17110109 | C | T | 0.10457 | 0.01126 | 0.04126 | Fatty liver | rs17110109 | C | T | -0.0073 | 0.82501 | 0.03299 | Hepatic fibrosis | rs17110109 | C | T | 0.06899 | 0.58217 | 0.12538 | Cirrhosis | rs17110109 | C | T | -0.0136 | 0.57447 | 0.02426liver cancer rs17110109 C T -0.0068 0.90389 0.05665 Choleliths rs17110109 C T -0.0172 0.0401 0.0084 Acute pancreatitis rs17110109 C T -0.0237 0.22495 0.01957 Chronic pancreatitis rs17110109 C T -0.0462 0.07995 0.02639 Pancreatic cancer rs17110109 C T -0.0273 0.50283 0.04082 Ulcerative colitis rs17110109 C T 0.00153 0.94366 0.02167 Crohn's disease rs17110109 C T -0.0409 0.27384 0.03742 Irrtable bowel syndrome rs17110109 C T 0.02633 0.10499 0.01624 Acute appendicitis rs17110109 C T 0.01955 0.03598 0.00932 Colon cancer rs17110109 C T -0.0116 0.64835 0.0255 Rectal cancer rs17110109 C T 0.0342 0.29083 0.03237 |
| Gastroesophageal reflux | rs17126938 | T | C | -0.0002 | 0.99298 | 0.01738 | Esophageal carcinoma | rs17126938 | T | C | -0.1683 | 0.13334 | 0.11212 | Acute gastritis | rs17126938 | T | C | 0.00367 | 0.9466 | 0.05483 | Chronic gastritis | rs17126938 | T | C | 0.00122 | 0.965 | 0.02775 | Gastroduodenal ulcer | rs17126938 | T | C | 0.02629 | 0.35514 | 0.02843 | Gastric cancer | rs17126938 | T | C | 0.02058 | 0.77456 | 0.07184 | Fatty liver | rs17126938 | T | C | 0.03479 | 0.5477 | 0.05786 | Hepatic fibrosis | rs17126938 | T | C | -0.2402 | 0.28033 | 0.22251 | Cirrhosis | rs17126938 | T | C | -0.0326 | 0.44411 | 0.04261liver cancer rs17126938 T C -0.1825 0.06574 0.09917 Choleliths rs17126938 T C 0.04137 0.00527 0.01483 Acute pancreatitis rs17126938 T C 0.05348 0.11834 0.03424 Chronic pancreatitis rs17126938 T C -0.003 0.94894 0.04627 Pancreatic cancer rs17126938 T C -0.0294 0.6814 0.07164 Ulcerative colitis rs17126938 T C -0.0504 0.18472 0.03803 Crohn's disease rs17126938 T C -0.0051 0.93742 0.06556 Irrtable bowel syndrome rs17126938 T C -0.0428 0.13194 0.02843 Acute appendicitis rs17126938 T C 0.01314 0.42192 0.01636 Colon cancer rs17126938 T C 0.04803 0.28071 0.04452 Rectal cancer rs17126938 T C 0.03522 0.53222 0.05638 |
| Gastroesophageal reflux | rs17425572 | G | A | -0.0031 | 0.75531 | 0.0099 | Esophageal carcinoma | rs17425572 | G | A | -0.0465 | 0.46898 | 0.06419 | Acute gastritis | rs17425572 | G | A | -0.0117 | 0.70917 | 0.03126 | Chronic gastritis | rs17425572 | G | A | 0.0175 | 0.26849 | 0.01581 | Gastroduodenal ulcer | rs17425572 | G | A | 0.01276 | 0.43063 | 0.0162 | Gastric cancer | rs17425572 | G | A | -0.0771 | 0.06157 | 0.04124 | Fatty liver | rs17425572 | G | A | -0.0017 | 0.95856 | 0.03288 | Hepatic fibrosis | rs17425572 | G | A | -0.1736 | 0.16623 | 0.12537 | Cirrhosis | rs17425572 | G | A | 0.0043 | 0.85899 | 0.02421liver cancer rs17425572 G A -0.0291 0.60671 0.05647 Choleliths rs17425572 G A -0.0116 0.16417 0.00837 Acute pancreatitis rs17425572 G A 0.02048 0.29449 0.01953 Chronic pancreatitis rs17425572 G A -0.0171 0.5174 0.02636 Pancreatic cancer rs17425572 G A 0.03933 0.33488 0.04078 Ulcerative colitis rs17425572 G A -0.0077 0.72151 0.02164 Crohn's disease rs17425572 G A 0.00955 0.79796 0.0373 Irrtable bowel syndrome rs17425572 G A -0.0122 0.45079 0.01619 Acute appendicitis rs17425572 G A 0.00086 0.92666 0.0093 Colon cancer rs17425572 G A 0.00344 0.89228 0.0254 Rectal cancer rs17425572 G A -0.0162 0.61444 0.03224 |
| Gastroesophageal reflux | rs17489649 | A | G | -0.0065 | 0.54554 | 0.01069 | Esophageal carcinoma | rs17489649 | A | G | -0.0559 | 0.4162 | 0.0688 | Acute gastritis | rs17489649 | A | G | 0.00058 | 0.98619 | 0.03373 | Chronic gastritis | rs17489649 | A | G | 0.00384 | 0.82205 | 0.01708 | Gastroduodenal ulcer | rs17489649 | A | G | -0.0162 | 0.35478 | 0.01747 | Gastric cancer | rs17489649 | A | G | 0.04876 | 0.27179 | 0.04437 | Fatty liver | rs17489649 | A | G | 0.04738 | 0.18158 | 0.03547 | Hepatic fibrosis | rs17489649 | A | G | -0.2302 | 0.09 | 0.1358 | Cirrhosis | rs17489649 | A | G | -0.0249 | 0.34046 | 0.02611liver cancer rs17489649 A G -0.0062 0.91929 0.06072 Choleliths rs17489649 A G 0.00877 0.33066 0.00902 Acute pancreatitis rs17489649 A G 0.01193 0.57103 0.02106 Chronic pancreatitis rs17489649 A G 0.02495 0.37992 0.02842 Pancreatic cancer rs17489649 A G 0.03931 0.36971 0.04383 Ulcerative colitis rs17489649 A G 0.05878 0.01186 0.02336 Crohn's disease rs17489649 A G -0.0509 0.20691 0.04029 Irrtable bowel syndrome rs17489649 A G -0.0089 0.61061 0.01746 Acute appendicitis rs17489649 A G -0.0044 0.65922 0.01003 Colon cancer rs17489649 A G 0.01399 0.60892 0.02734 Rectal cancer rs17489649 A G -0.003 0.93039 0.03471 |
| Gastroesophageal reflux | rs175325 | A | T | 0.01012 | 0.30924 | 0.00996 | Esophageal carcinoma | rs175325 | A | T | 0.00464 | 0.94258 | 0.06442 | Acute gastritis | rs175325 | A | T | -0.0169 | 0.59 | 0.03145 | Chronic gastritis | rs175325 | A | T | -0.0102 | 0.52135 | 0.01592 | Gastroduodenal ulcer | rs175325 | A | T | -0.0119 | 0.46341 | 0.01628 | Gastric cancer | rs175325 | A | T | 0.04518 | 0.27609 | 0.04148 | Fatty liver | rs175325 | A | T | -0.0058 | 0.8597 | 0.03308 | Hepatic fibrosis | rs175325 | A | T | 0.16987 | 0.17695 | 0.12581 | Cirrhosis | rs175325 | A | T | -0.0058 | 0.81102 | 0.02436liver cancer rs175325 A T -0.0755 0.18381 0.05679 Choleliths rs175325 A T 0.00137 0.87098 0.00843 Acute pancreatitis rs175325 A T -0.0053 0.78708 0.01964 Chronic pancreatitis rs175325 A T -0.0195 0.46077 0.02648 Pancreatic cancer rs175325 A T 0.01198 0.77023 0.04101 Ulcerative colitis rs175325 A T -0.0046 0.83222 0.02174 Crohn's disease rs175325 A T 0.04038 0.28137 0.03748 Irrtable bowel syndrome rs175325 A T 0.02413 0.13857 0.01629 Acute appendicitis rs175325 A T -0.0075 0.42434 0.00936 Colon cancer rs175325 A T -0.0082 0.74766 0.0256 Rectal cancer rs175325 A T -0.0072 0.82502 0.03241 |
| Gastroesophageal reflux | rs17551064 | G | A | 0.02777 | 0.03942 | 0.01348 | Esophageal carcinoma | rs17551064 | G | A | 0.14696 | 0.09255 | 0.08736 | Acute gastritis | rs17551064 | G | A | -0.0566 | 0.18185 | 0.04237 | Chronic gastritis | rs17551064 | G | A | 0.02292 | 0.28601 | 0.02149 | Gastroduodenal ulcer | rs17551064 | G | A | 0.04519 | 0.03944 | 0.02194 | Gastric cancer | rs17551064 | G | A | -0.105 | 0.05929 | 0.05567 | Fatty liver | rs17551064 | G | A | 0.14708 | 0.00065 | 0.04312 | Hepatic fibrosis | rs17551064 | G | A | 0.2436 | 0.15545 | 0.17148 | Cirrhosis | rs17551064 | G | A | 0.0569 | 0.0857 | 0.03311liver cancer rs17551064 G A 0.04963 0.51785 0.07675 Choleliths rs17551064 G A -0.0048 0.67522 0.01137 Acute pancreatitis rs17551064 G A -0.0284 0.28523 0.02662 Chronic pancreatitis rs17551064 G A -0.0007 0.98399 0.036 Pancreatic cancer rs17551064 G A 0.06036 0.275 0.05529 Ulcerative colitis rs17551064 G A 0.05114 0.08364 0.02956 Crohn's disease rs17551064 G A 0.05003 0.32601 0.05094 Irrtable bowel syndrome rs17551064 G A 0.0068 0.75757 0.02202 Acute appendicitis rs17551064 G A -0.0019 0.88031 0.01269 Colon cancer rs17551064 G A -0.0433 0.21145 0.03462 Rectal cancer rs17551064 G A 0.04512 0.30482 0.04397 |
| Gastroesophageal reflux | rs17563464 | A | C | 0.01635 | 0.24055 | 0.01393 | Esophageal carcinoma | rs17563464 | A | C | 0.04032 | 0.65289 | 0.08965 | Acute gastritis | rs17563464 | A | C | -0.0551 | 0.21342 | 0.04432 | Chronic gastritis | rs17563464 | A | C | 0.00362 | 0.87086 | 0.02228 | Gastroduodenal ulcer | rs17563464 | A | C | -0.0032 | 0.88871 | 0.0229 | Gastric cancer | rs17563464 | A | C | -0.0827 | 0.1492 | 0.05732 | Fatty liver | rs17563464 | A | C | 0.02499 | 0.58939 | 0.04631 | Hepatic fibrosis | rs17563464 | A | C | -0.1187 | 0.51165 | 0.18087 | Cirrhosis | rs17563464 | A | C | -0.0605 | 0.07868 | 0.03438liver cancer rs17563464 A C 0.04472 0.57388 0.07952 Choleliths rs17563464 A C 0.01784 0.13021 0.01179 Acute pancreatitis rs17563464 A C -0.0027 0.92109 0.02762 Chronic pancreatitis rs17563464 A C -0.0473 0.20617 0.0374 Pancreatic cancer rs17563464 A C -0.0316 0.58427 0.05768 Ulcerative colitis rs17563464 A C 0.02777 0.36444 0.03062 Crohn's disease rs17563464 A C 0.01775 0.73598 0.05265 Irrtable bowel syndrome rs17563464 A C -0.0068 0.76613 0.0228 Acute appendicitis rs17563464 A C -0.0204 0.12119 0.01316 Colon cancer rs17563464 A C -0.0016 0.96368 0.03537 Rectal cancer rs17563464 A C -0.0155 0.73058 0.04487 |
| Gastroesophageal reflux | rs17565975 | A | G | 0.00877 | 0.37257 | 0.00984 | Esophageal carcinoma | rs17565975 | A | G | -0.069 | 0.27912 | 0.06376 | Acute gastritis | rs17565975 | A | G | -0.0046 | 0.88114 | 0.031 | Chronic gastritis | rs17565975 | A | G | -0.0036 | 0.81891 | 0.0157 | Gastroduodenal ulcer | rs17565975 | A | G | 0.00549 | 0.73278 | 0.01607 | Gastric cancer | rs17565975 | A | G | 0.00564 | 0.89011 | 0.04082 | Fatty liver | rs17565975 | A | G | -0.0722 | 0.02716 | 0.0327 | Hepatic fibrosis | rs17565975 | A | G | 0.01067 | 0.93183 | 0.12471 | Cirrhosis | rs17565975 | A | G | 0.00479 | 0.84218 | 0.02405liver cancer rs17565975 A G -0.0369 0.51072 0.05611 Choleliths rs17565975 A G 0.00628 0.44964 0.00831 Acute pancreatitis rs17565975 A G 0.01702 0.38019 0.01939 Chronic pancreatitis rs17565975 A G 0.01637 0.53132 0.02615 Pancreatic cancer rs17565975 A G -0.055 0.17442 0.04047 Ulcerative colitis rs17565975 A G -0.0297 0.16676 0.02149 Crohn's disease rs17565975 A G 0.00233 0.94981 0.03709 Irrtable bowel syndrome rs17565975 A G 0.01436 0.37213 0.01609 Acute appendicitis rs17565975 A G 0.00938 0.31035 0.00924 Colon cancer rs17565975 A G -0.0709 0.00471 0.0251 Rectal cancer rs17565975 A G -0.0326 0.30796 0.03202 |
| Gastroesophageal reflux | rs17598675 | C | T | -0.0067 | 0.49994 | 0.01 | Esophageal carcinoma | rs17598675 | C | T | 0.01496 | 0.81753 | 0.06484 | Acute gastritis | rs17598675 | C | T | 0.03157 | 0.31768 | 0.0316 | Chronic gastritis | rs17598675 | C | T | -0.0357 | 0.02571 | 0.01599 | Gastroduodenal ulcer | rs17598675 | C | T | -0.0191 | 0.24265 | 0.01638 | Gastric cancer | rs17598675 | C | T | -0.0045 | 0.91466 | 0.04154 | Fatty liver | rs17598675 | C | T | -0.0089 | 0.78936 | 0.03326 | Hepatic fibrosis | rs17598675 | C | T | 0.06043 | 0.63422 | 0.127 | Cirrhosis | rs17598675 | C | T | -0.0051 | 0.83577 | 0.02451liver cancer rs17598675 C T -0.0932 0.10323 0.05718 Choleliths rs17598675 C T -0.0058 0.48989 0.00846 Acute pancreatitis rs17598675 C T 0.0108 0.58414 0.01974 Chronic pancreatitis rs17598675 C T -0.0056 0.8344 0.02662 Pancreatic cancer rs17598675 C T -0.0165 0.6895 0.04125 Ulcerative colitis rs17598675 C T 0.03092 0.15691 0.02184 Crohn's disease rs17598675 C T -0.0411 0.27552 0.03766 Irrtable bowel syndrome rs17598675 C T -0.0013 0.93632 0.01635 Acute appendicitis rs17598675 C T -0.0039 0.67641 0.0094 Colon cancer rs17598675 C T 0.02346 0.36147 0.0257 Rectal cancer rs17598675 C T 0.01075 0.74157 0.03261 |
| Gastroesophageal reflux | rs176218 | G | T | -0.0016 | 0.8861 | 0.0113 | Esophageal carcinoma | rs176218 | G | T | -0.0524 | 0.47256 | 0.07299 | Acute gastritis | rs176218 | G | T | 0.04256 | 0.23414 | 0.03577 | Chronic gastritis | rs176218 | G | T | -0.0039 | 0.82937 | 0.01804 | Gastroduodenal ulcer | rs176218 | G | T | -0.0007 | 0.96927 | 0.0185 | Gastric cancer | rs176218 | G | T | -0.0161 | 0.73157 | 0.04706 | Fatty liver | rs176218 | G | T | 0.03133 | 0.40269 | 0.03743 | Hepatic fibrosis | rs176218 | G | T | 0.00493 | 0.97246 | 0.14272 | Cirrhosis | rs176218 | G | T | 0.03942 | 0.15301 | 0.02759liver cancer rs176218 G T 0.0651 0.31343 0.06458 Choleliths rs176218 G T 0.01932 0.04336 0.00956 Acute pancreatitis rs176218 G T 0.01521 0.4946 0.02228 Chronic pancreatitis rs176218 G T 0.00458 0.8785 0.02997 Pancreatic cancer rs176218 G T -0.0001 0.99775 0.04659 Ulcerative colitis rs176218 G T -0.0085 0.73174 0.02465 Crohn's disease rs176218 G T -0.0342 0.42068 0.04246 Irrtable bowel syndrome rs176218 G T 0.00108 0.95318 0.01844 Acute appendicitis rs176218 G T 0.00807 0.44683 0.01061 Colon cancer rs176218 G T 0.01297 0.65491 0.02902 Rectal cancer rs176218 G T -0.006 0.87076 0.03677 |
| Gastroesophageal reflux | rs1827540 | G | A | -0.0168 | 0.09197 | 0.00996 | Esophageal carcinoma | rs1827540 | G | A | -0.0694 | 0.28234 | 0.06458 | Acute gastritis | rs1827540 | G | A | 0.05338 | 0.08964 | 0.03145 | Chronic gastritis | rs1827540 | G | A | 0.01368 | 0.39019 | 0.01592 | Gastroduodenal ulcer | rs1827540 | G | A | 0.0086 | 0.59747 | 0.0163 | Gastric cancer | rs1827540 | G | A | -0.0148 | 0.72117 | 0.04146 | Fatty liver | rs1827540 | G | A | 0.02178 | 0.51047 | 0.0331 | Hepatic fibrosis | rs1827540 | G | A | -0.0109 | 0.93147 | 0.12639 | Cirrhosis | rs1827540 | G | A | -0.0514 | 0.03517 | 0.02439liver cancer rs1827540 G A 0.12109 0.0337 0.05702 Choleliths rs1827540 G A -0.0027 0.75169 0.00842 Acute pancreatitis rs1827540 G A 0.04557 0.02048 0.01966 Chronic pancreatitis rs1827540 G A 0.01077 0.68448 0.0265 Pancreatic cancer rs1827540 G A 0.05224 0.20391 0.04112 Ulcerative colitis rs1827540 G A 0.00288 0.89493 0.02178 Crohn's disease rs1827540 G A -0.0045 0.90395 0.03754 Irrtable bowel syndrome rs1827540 G A -0.0254 0.11808 0.01628 Acute appendicitis rs1827540 G A 0.01196 0.2014 0.00937 Colon cancer rs1827540 G A 0.03887 0.1284 0.02556 Rectal cancer rs1827540 G A 0.0677 0.03715 0.03249 |
| Gastroesophageal reflux | rs1866823 | A | G | -0.0123 | 0.22657 | 0.0102 | Esophageal carcinoma | rs1866823 | A | G | -0.0659 | 0.31689 | 0.06581 | Acute gastritis | rs1866823 | A | G | -0.0172 | 0.59303 | 0.0322 | Chronic gastritis | rs1866823 | A | G | 0.00777 | 0.63341 | 0.0163 | Gastroduodenal ulcer | rs1866823 | A | G | -0.0339 | 0.04198 | 0.01666 | Gastric cancer | rs1866823 | A | G | -0.0355 | 0.40312 | 0.04249 | Fatty liver | rs1866823 | A | G | -0.0016 | 0.96268 | 0.03388 | Hepatic fibrosis | rs1866823 | A | G | -0.3243 | 0.01225 | 0.12947 | Cirrhosis | rs1866823 | A | G | -0.0816 | 0.00097 | 0.02473liver cancer rs1866823 A G 0.11719 0.04426 0.05826 Choleliths rs1866823 A G 0.00018 0.9834 0.00862 Acute pancreatitis rs1866823 A G -0.0416 0.0387 0.02015 Chronic pancreatitis rs1866823 A G -0.0526 0.05286 0.02718 Pancreatic cancer rs1866823 A G 0.07236 0.08482 0.04199 Ulcerative colitis rs1866823 A G 0.04867 0.02894 0.02228 Crohn's disease rs1866823 A G 0.0122 0.75092 0.03844 Irrtable bowel syndrome rs1866823 A G 0.02301 0.16762 0.01667 Acute appendicitis rs1866823 A G -0.002 0.83339 0.00958 Colon cancer rs1866823 A G 0.00091 0.97231 0.02617 Rectal cancer rs1866823 A G -0.0478 0.15016 0.03324 |
| Gastroesophageal reflux | rs1882273 | G | C | -0.024 | 0.02235 | 0.01051 | Esophageal carcinoma | rs1882273 | G | C | -0.0454 | 0.50454 | 0.06797 | Acute gastritis | rs1882273 | G | C | 0.01376 | 0.67853 | 0.0332 | Chronic gastritis | rs1882273 | G | C | -0.027 | 0.10857 | 0.01681 | Gastroduodenal ulcer | rs1882273 | G | C | -0.0046 | 0.79083 | 0.01719 | Gastric cancer | rs1882273 | G | C | 0.06446 | 0.14019 | 0.0437 | Fatty liver | rs1882273 | G | C | -0.0143 | 0.682 | 0.03491 | Hepatic fibrosis | rs1882273 | G | C | -0.0283 | 0.83239 | 0.13369 | Cirrhosis | rs1882273 | G | C | -0.0182 | 0.48072 | 0.02574liver cancer rs1882273 G C 0.0372 0.53516 0.05999 Choleliths rs1882273 G C -0.0016 0.86058 0.00888 Acute pancreatitis rs1882273 G C -0.0019 0.92539 0.02074 Chronic pancreatitis rs1882273 G C -0.0306 0.27375 0.02794 Pancreatic cancer rs1882273 G C 0.00857 0.84289 0.04322 Ulcerative colitis rs1882273 G C 0.01093 0.63416 0.02298 Crohn's disease rs1882273 G C -0.059 0.13609 0.03962 Irrtable bowel syndrome rs1882273 G C -0.0186 0.27879 0.01719 Acute appendicitis rs1882273 G C -0.0115 0.2432 0.00988 Colon cancer rs1882273 G C -0.0247 0.35811 0.02693 Rectal cancer rs1882273 G C -0.0222 0.51539 0.03416 |
| Gastroesophageal reflux | rs192436652 | C | T | -0.0465 | 0.04055 | 0.0227 | Esophageal carcinoma | rs192436652 | C | T | -0.0383 | 0.79517 | 0.14765 | Acute gastritis | rs192436652 | C | T | 0.11489 | 0.11065 | 0.07202 | Chronic gastritis | rs192436652 | C | T | 0.02289 | 0.52963 | 0.03641 | Gastroduodenal ulcer | rs192436652 | C | T | -0.0862 | 0.02061 | 0.03722 | Gastric cancer | rs192436652 | C | T | -0.0943 | 0.31228 | 0.09333 | Fatty liver | rs192436652 | C | T | -0.0201 | 0.79083 | 0.07587 | Hepatic fibrosis | rs192436652 | C | T | -0.405 | 0.16655 | 0.29273 | Cirrhosis | rs192436652 | C | T | -0.0265 | 0.63579 | 0.05603liver cancer rs192436652 C T 0.16431 0.21228 0.13173 Choleliths rs192436652 C T 0.00042 0.98266 0.01916 Acute pancreatitis rs192436652 C T 0.02772 0.53691 0.04489 Chronic pancreatitis rs192436652 C T 0.05517 0.36518 0.06092 Pancreatic cancer rs192436652 C T 0.00612 0.94836 0.09449 Ulcerative colitis rs192436652 C T -0.0674 0.17458 0.04964 Crohn's disease rs192436652 C T 0.29704 0.00149 0.09353 Irrtable bowel syndrome rs192436652 C T -0.0387 0.29897 0.03725 Acute appendicitis rs192436652 C T -0.0058 0.78609 0.02145 Colon cancer rs192436652 C T -0.0241 0.67747 0.05785 Rectal cancer rs192436652 C T 0.02811 0.70341 0.07385 |
| Gastroesophageal reflux | rs1925576 | A | G | -0.0056 | 0.57312 | 0.00985 | Esophageal carcinoma | rs1925576 | A | G | -0.0413 | 0.51677 | 0.06367 | Acute gastritis | rs1925576 | A | G | -0.0289 | 0.35169 | 0.03108 | Chronic gastritis | rs1925576 | A | G | 0.02246 | 0.15402 | 0.01576 | Gastroduodenal ulcer | rs1925576 | A | G | -0.0084 | 0.60306 | 0.01609 | Gastric cancer | rs1925576 | A | G | 0.05314 | 0.19509 | 0.04101 | Fatty liver | rs1925576 | A | G | 0.01453 | 0.65682 | 0.03271 | Hepatic fibrosis | rs1925576 | A | G | -0.0005 | 0.99657 | 0.12493 | Cirrhosis | rs1925576 | A | G | 0.00519 | 0.82907 | 0.02406liver cancer rs1925576 A G 0.06443 0.25103 0.05614 Choleliths rs1925576 A G 0.00956 0.25067 0.00832 Acute pancreatitis rs1925576 A G 0.01642 0.39736 0.01941 Chronic pancreatitis rs1925576 A G -0.0181 0.48768 0.02615 Pancreatic cancer rs1925576 A G -0.0314 0.43884 0.04053 Ulcerative colitis rs1925576 A G -0.0306 0.15519 0.02151 Crohn's disease rs1925576 A G -0.0296 0.42602 0.03717 Irrtable bowel syndrome rs1925576 A G 0.01541 0.33863 0.01611 Acute appendicitis rs1925576 A G 0.0146 0.11471 0.00925 Colon cancer rs1925576 A G 0.01824 0.47061 0.02528 Rectal cancer rs1925576 A G -0.0012 0.9713 0.03213 |
| Gastroesophageal reflux | rs1947114 | G | A | -0.0077 | 0.4698 | 0.01063 | Esophageal carcinoma | rs1947114 | G | A | -0.0793 | 0.24974 | 0.06891 | Acute gastritis | rs1947114 | G | A | -0.031 | 0.35459 | 0.03353 | Chronic gastritis | rs1947114 | G | A | -0.007 | 0.6787 | 0.01699 | Gastroduodenal ulcer | rs1947114 | G | A | -0.0114 | 0.51077 | 0.01739 | Gastric cancer | rs1947114 | G | A | 0.00463 | 0.91691 | 0.04433 | Fatty liver | rs1947114 | G | A | -0.0424 | 0.22897 | 0.03524 | Hepatic fibrosis | rs1947114 | G | A | 0.03968 | 0.768 | 0.1345 | Cirrhosis | rs1947114 | G | A | -0.0098 | 0.70531 | 0.02596liver cancer rs1947114 G A -0.0697 0.25031 0.06063 Choleliths rs1947114 G A -0.0255 0.00455 0.009 Acute pancreatitis rs1947114 G A -0.0619 0.00326 0.02103 Chronic pancreatitis rs1947114 G A -0.0025 0.92916 0.02818 Pancreatic cancer rs1947114 G A -0.0037 0.93332 0.04379 Ulcerative colitis rs1947114 G A 0.04912 0.03363 0.02312 Crohn's disease rs1947114 G A -0.0617 0.12203 0.0399 Irrtable bowel syndrome rs1947114 G A 0.03041 0.07963 0.01735 Acute appendicitis rs1947114 G A 0.00088 0.92931 0.00996 Colon cancer rs1947114 G A -0.0536 0.0503 0.02738 Rectal cancer rs1947114 G A -0.0124 0.72041 0.0347 |
| Gastroesophageal reflux | rs1964927 | G | A | 0.00301 | 0.76531 | 0.01008 | Esophageal carcinoma | rs1964927 | G | A | 0.07893 | 0.22458 | 0.065 | Acute gastritis | rs1964927 | G | A | 0.0682 | 0.03186 | 0.03178 | Chronic gastritis | rs1964927 | G | A | -0.0015 | 0.92692 | 0.01611 | Gastroduodenal ulcer | rs1964927 | G | A | 0.00875 | 0.59537 | 0.01647 | Gastric cancer | rs1964927 | G | A | -0.0235 | 0.5753 | 0.04187 | Fatty liver | rs1964927 | G | A | -0.0135 | 0.68686 | 0.03344 | Hepatic fibrosis | rs1964927 | G | A | -0.2825 | 0.02648 | 0.12731 | Cirrhosis | rs1964927 | G | A | -0.0127 | 0.60652 | 0.02461liver cancer rs1964927 G A 0.00514 0.92859 0.05736 Choleliths rs1964927 G A 0.01012 0.23512 0.00852 Acute pancreatitis rs1964927 G A 0.01227 0.5367 0.01985 Chronic pancreatitis rs1964927 G A 0.04479 0.09397 0.02674 Pancreatic cancer rs1964927 G A 0.00102 0.98039 0.0415 Ulcerative colitis rs1964927 G A -0.0089 0.68463 0.022 Crohn's disease rs1964927 G A -0.0158 0.67697 0.03793 Irrtable bowel syndrome rs1964927 G A -0.0275 0.09567 0.01648 Acute appendicitis rs1964927 G A 0.00504 0.59398 0.00946 Colon cancer rs1964927 G A -0.0112 0.664 0.02584 Rectal cancer rs1964927 G A -0.0178 0.58783 0.0328 |
| Gastroesophageal reflux | rs2016392 | A | G | 0.0059 | 0.59514 | 0.0111 | Esophageal carcinoma | rs2016392 | A | G | -0.013 | 0.85749 | 0.07224 | Acute gastritis | rs2016392 | A | G | 0.00971 | 0.78184 | 0.03506 | Chronic gastritis | rs2016392 | A | G | 0.01465 | 0.40916 | 0.01775 | Gastroduodenal ulcer | rs2016392 | A | G | -0.0209 | 0.24944 | 0.01815 | Gastric cancer | rs2016392 | A | G | 0.03053 | 0.51097 | 0.04645 | Fatty liver | rs2016392 | A | G | 0.05469 | 0.13755 | 0.03683 | Hepatic fibrosis | rs2016392 | A | G | 0.07762 | 0.57989 | 0.14023 | Cirrhosis | rs2016392 | A | G | -0.0187 | 0.49017 | 0.02706liver cancer rs2016392 A G -0.0899 0.1574 0.06357 Choleliths rs2016392 A G 0.01275 0.17445 0.00939 Acute pancreatitis rs2016392 A G 0.02336 0.28472 0.02183 Chronic pancreatitis rs2016392 A G 0.03276 0.26528 0.0294 Pancreatic cancer rs2016392 A G 0.00133 0.97687 0.04578 Ulcerative colitis rs2016392 A G 0.00534 0.82514 0.02417 Crohn's disease rs2016392 A G 0.00899 0.8296 0.04177 Irrtable bowel syndrome rs2016392 A G 0.01818 0.31597 0.01813 Acute appendicitis rs2016392 A G -0.0058 0.57664 0.0104 Colon cancer rs2016392 A G 0.05204 0.06861 0.02858 Rectal cancer rs2016392 A G 0.06153 0.08993 0.03628 |
| Gastroesophageal reflux | rs2052285 | A | G | -0.0195 | 0.04863 | 0.00991 | Esophageal carcinoma | rs2052285 | A | G | 0.04699 | 0.46506 | 0.06433 | Acute gastritis | rs2052285 | A | G | -0.0041 | 0.89567 | 0.03128 | Chronic gastritis | rs2052285 | A | G | -0.0175 | 0.26969 | 0.01584 | Gastroduodenal ulcer | rs2052285 | A | G | -0.0106 | 0.51419 | 0.0162 | Gastric cancer | rs2052285 | A | G | -0.0381 | 0.35717 | 0.04134 | Fatty liver | rs2052285 | A | G | 0.02252 | 0.49418 | 0.03294 | Hepatic fibrosis | rs2052285 | A | G | 0.22452 | 0.07326 | 0.12535 | Cirrhosis | rs2052285 | A | G | 0.07051 | 0.00361 | 0.02423liver cancer rs2052285 A G 0.04792 0.39723 0.0566 Choleliths rs2052285 A G -0.0126 0.13172 0.00838 Acute pancreatitis rs2052285 A G 0.02975 0.1277 0.01953 Chronic pancreatitis rs2052285 A G 0.02156 0.4124 0.02631 Pancreatic cancer rs2052285 A G 0.00148 0.97118 0.04085 Ulcerative colitis rs2052285 A G 0.04896 0.02354 0.02162 Crohn's disease rs2052285 A G 0.01554 0.67687 0.03729 Irrtable bowel syndrome rs2052285 A G -0.0202 0.21259 0.01619 Acute appendicitis rs2052285 A G 0.00494 0.59579 0.00931 Colon cancer rs2052285 A G -0.015 0.55484 0.02548 Rectal cancer rs2052285 A G 0.03368 0.2976 0.03234 |
| Gastroesophageal reflux | rs2067854 | A | G | 0.00373 | 0.73682 | 0.0111 | Esophageal carcinoma | rs2067854 | A | G | -0.0277 | 0.69881 | 0.07163 | Acute gastritis | rs2067854 | A | G | -0.0198 | 0.57222 | 0.03497 | Chronic gastritis | rs2067854 | A | G | 0.00537 | 0.76224 | 0.01775 | Gastroduodenal ulcer | rs2067854 | A | G | 0.01768 | 0.3289 | 0.01811 | Gastric cancer | rs2067854 | A | G | 0.01835 | 0.69034 | 0.04607 | Fatty liver | rs2067854 | A | G | 0.01489 | 0.68592 | 0.03682 | Hepatic fibrosis | rs2067854 | A | G | -0.2332 | 0.09699 | 0.14053 | Cirrhosis | rs2067854 | A | G | -0.0102 | 0.70557 | 0.02709liver cancer rs2067854 A G -0.0416 0.51003 0.06307 Choleliths rs2067854 A G -0.0015 0.8731 0.00938 Acute pancreatitis rs2067854 A G 0.01892 0.38661 0.02185 Chronic pancreatitis rs2067854 A G 0.01292 0.6613 0.02948 Pancreatic cancer rs2067854 A G -0.0179 0.6952 0.04559 Ulcerative colitis rs2067854 A G 0.0012 0.96061 0.02421 Crohn's disease rs2067854 A G -0.0276 0.50824 0.04177 Irrtable bowel syndrome rs2067854 A G -0.0138 0.4464 0.01812 Acute appendicitis rs2067854 A G 0.02459 0.01811 0.0104 Colon cancer rs2067854 A G 0.04158 0.14462 0.0285 Rectal cancer rs2067854 A G 0.02514 0.48637 0.03612 |
| Gastroesophageal reflux | rs2179152 | C | T | -0.0297 | 0.00472 | 0.01052 | Esophageal carcinoma | rs2179152 | C | T | -0.036 | 0.59957 | 0.06862 | Acute gastritis | rs2179152 | C | T | -0.0409 | 0.2208 | 0.03338 | Chronic gastritis | rs2179152 | C | T | -0.01 | 0.55444 | 0.01689 | Gastroduodenal ulcer | rs2179152 | C | T | -0.0173 | 0.31734 | 0.01729 | Gastric cancer | rs2179152 | C | T | -0.0525 | 0.23223 | 0.04393 | Fatty liver | rs2179152 | C | T | -0.0228 | 0.51587 | 0.03509 | Hepatic fibrosis | rs2179152 | C | T | -0.1434 | 0.28711 | 0.1347 | Cirrhosis | rs2179152 | C | T | -0.0071 | 0.78394 | 0.02589liver cancer rs2179152 C T -0.0392 0.5175 0.06057 Choleliths rs2179152 C T -0.0268 0.00259 0.0089 Acute pancreatitis rs2179152 C T 0.00577 0.78202 0.02087 Chronic pancreatitis rs2179152 C T -0.0261 0.35466 0.02816 Pancreatic cancer rs2179152 C T 0.10405 0.01717 0.04366 Ulcerative colitis rs2179152 C T -0.0868 0.00014 0.02278 Crohn's disease rs2179152 C T -0.0115 0.77372 0.03985 Irrtable bowel syndrome rs2179152 C T 0.01974 0.25203 0.01724 Acute appendicitis rs2179152 C T 0.01315 0.18543 0.00993 Colon cancer rs2179152 C T 0.04337 0.10957 0.0271 Rectal cancer rs2179152 C T -0.0254 0.46095 0.03441 |
| Gastroesophageal reflux | rs2182505 | T | C | -0.002 | 0.87409 | 0.01259 | Esophageal carcinoma | rs2182505 | T | C | 0.03004 | 0.7118 | 0.08131 | Acute gastritis | rs2182505 | T | C | 0.00506 | 0.89869 | 0.03978 | Chronic gastritis | rs2182505 | T | C | -0.0094 | 0.6381 | 0.02007 | Gastroduodenal ulcer | rs2182505 | T | C | -0.0128 | 0.53452 | 0.02054 | Gastric cancer | rs2182505 | T | C | 0.07258 | 0.16518 | 0.05229 | Fatty liver | rs2182505 | T | C | 0.01314 | 0.75388 | 0.04191 | Hepatic fibrosis | rs2182505 | T | C | -0.1776 | 0.26797 | 0.16035 | Cirrhosis | rs2182505 | T | C | -0.021 | 0.4956 | 0.03088liver cancer rs2182505 T C -0.029 0.68728 0.07205 Choleliths rs2182505 T C -0.002 0.85114 0.01064 Acute pancreatitis rs2182505 T C -0.0391 0.11632 0.02487 Chronic pancreatitis rs2182505 T C -0.0676 0.04405 0.03358 Pancreatic cancer rs2182505 T C -0.0153 0.76835 0.0518 Ulcerative colitis rs2182505 T C 0.07035 0.01087 0.02762 Crohn's disease rs2182505 T C 0.00188 0.96851 0.04763 Irrtable bowel syndrome rs2182505 T C -0.0062 0.76337 0.02061 Acute appendicitis rs2182505 T C -0.0094 0.42989 0.01188 Colon cancer rs2182505 T C 0.01223 0.70449 0.03223 Rectal cancer rs2182505 T C -0.0131 0.74852 0.04096 |
| Gastroesophageal reflux | rs2256965 | G | A | ####### | 0.99339 | 0.00988 | Esophageal carcinoma | rs2256965 | G | A | -0.1141 | 0.07381 | 0.06382 | Acute gastritis | rs2256965 | G | A | 0.01816 | 0.56075 | 0.03122 | Chronic gastritis | rs2256965 | G | A | 0.03629 | 0.02163 | 0.0158 | Gastroduodenal ulcer | rs2256965 | G | A | -0.001 | 0.94841 | 0.01615 | Gastric cancer | rs2256965 | G | A | -0.0083 | 0.84031 | 0.04096 | Fatty liver | rs2256965 | G | A | 0.04508 | 0.16887 | 0.03276 | Hepatic fibrosis | rs2256965 | G | A | 0.06854 | 0.58326 | 0.12493 | Cirrhosis | rs2256965 | G | A | -0.0487 | 0.04327 | 0.0241liver cancer rs2256965 G A 0.03605 0.52178 0.05627 Choleliths rs2256965 G A 0.01802 0.03095 0.00835 Acute pancreatitis rs2256965 G A 0.02641 0.17423 0.01944 Chronic pancreatitis rs2256965 G A 0.05159 0.04875 0.02618 Pancreatic cancer rs2256965 G A -0.0256 0.52909 0.04066 Ulcerative colitis rs2256965 G A 0.05341 0.01312 0.02153 Crohn's disease rs2256965 G A 0.05415 0.14543 0.03719 Irrtable bowel syndrome rs2256965 G A 0.02481 0.1244 0.01615 Acute appendicitis rs2256965 G A 0.0406 1.2E-05 0.00928 Colon cancer rs2256965 G A -0.0254 0.31678 0.02534 Rectal cancer rs2256965 G A 0.00261 0.93513 0.0321 |
| Gastroesophageal reflux | rs2283076 | A | G | -0.0225 | 0.09962 | 0.01368 | Esophageal carcinoma | rs2283076 | A | G | -0.022 | 0.8024 | 0.08807 | Acute gastritis | rs2283076 | A | G | -0.0503 | 0.24245 | 0.043 | Chronic gastritis | rs2283076 | A | G | 0.01078 | 0.62179 | 0.02184 | Gastroduodenal ulcer | rs2283076 | A | G | -0.0311 | 0.16439 | 0.02238 | Gastric cancer | rs2283076 | A | G | 0.01046 | 0.85432 | 0.05699 | Fatty liver | rs2283076 | A | G | -0.0664 | 0.14261 | 0.04531 | Hepatic fibrosis | rs2283076 | A | G | 0.25032 | 0.15062 | 0.17416 | Cirrhosis | rs2283076 | A | G | 0.00983 | 0.76939 | 0.03354liver cancer rs2283076 A G 0.0248 0.75023 0.07789 Choleliths rs2283076 A G 0.00082 0.94344 0.01158 Acute pancreatitis rs2283076 A G -0.0199 0.4618 0.02707 Chronic pancreatitis rs2283076 A G -0.0731 0.04455 0.03641 Pancreatic cancer rs2283076 A G 0.0234 0.67795 0.05636 Ulcerative colitis rs2283076 A G -0.0064 0.83127 0.02995 Crohn's disease rs2283076 A G 0.03858 0.45386 0.05151 Irrtable bowel syndrome rs2283076 A G 0.02939 0.18764 0.0223 Acute appendicitis rs2283076 A G -0.0001 0.99239 0.01286 Colon cancer rs2283076 A G -0.0016 0.9633 0.03525 Rectal cancer rs2283076 A G -0.0025 0.95576 0.04457 |
| Gastroesophageal reflux | rs2287838 | A | G | -0.0181 | 0.06743 | 0.00987 | Esophageal carcinoma | rs2287838 | A | G | -0.0229 | 0.71987 | 0.06382 | Acute gastritis | rs2287838 | A | G | -0.0318 | 0.30808 | 0.03118 | Chronic gastritis | rs2287838 | A | G | -0.0052 | 0.74292 | 0.01578 | Gastroduodenal ulcer | rs2287838 | A | G | -0.0066 | 0.6823 | 0.01616 | Gastric cancer | rs2287838 | A | G | -0.0207 | 0.61447 | 0.04109 | Fatty liver | rs2287838 | A | G | -0.0067 | 0.83738 | 0.03276 | Hepatic fibrosis | rs2287838 | A | G | -0.2798 | 0.02513 | 0.12494 | Cirrhosis | rs2287838 | A | G | -0.0367 | 0.12881 | 0.02417liver cancer rs2287838 A G 0.0167 0.7677 0.05653 Choleliths rs2287838 A G 0.00236 0.77688 0.00834 Acute pancreatitis rs2287838 A G -0.0072 0.70976 0.01946 Chronic pancreatitis rs2287838 A G -0.0305 0.24525 0.02625 Pancreatic cancer rs2287838 A G -0.0439 0.28053 0.04067 Ulcerative colitis rs2287838 A G -0.0273 0.20518 0.02155 Crohn's disease rs2287838 A G -0.0043 0.90733 0.03711 Irrtable bowel syndrome rs2287838 A G 0.00254 0.87469 0.01613 Acute appendicitis rs2287838 A G 0.01028 0.26764 0.00927 Colon cancer rs2287838 A G -0.0268 0.2905 0.02536 Rectal cancer rs2287838 A G 0.04735 0.14104 0.03217 |
| Gastroesophageal reflux | rs2302761 | T | C | -0.0178 | 0.16504 | 0.01282 | Esophageal carcinoma | rs2302761 | T | C | -0.086 | 0.30027 | 0.08298 | Acute gastritis | rs2302761 | T | C | -0.0215 | 0.595 | 0.0404 | Chronic gastritis | rs2302761 | T | C | -0.0364 | 0.0757 | 0.02049 | Gastroduodenal ulcer | rs2302761 | T | C | -0.0157 | 0.45355 | 0.02093 | Gastric cancer | rs2302761 | T | C | 0.08148 | 0.12623 | 0.05329 | Fatty liver | rs2302761 | T | C | 0.02696 | 0.52636 | 0.04255 | Hepatic fibrosis | rs2302761 | T | C | 0.17625 | 0.27893 | 0.16278 | Cirrhosis | rs2302761 | T | C | 0.03903 | 0.21473 | 0.03146liver cancer rs2302761 T C -0.0645 0.37961 0.07342 Choleliths rs2302761 T C -0.0209 0.0542 0.01085 Acute pancreatitis rs2302761 T C -0.0126 0.61939 0.02532 Chronic pancreatitis rs2302761 T C 0.03069 0.36933 0.03419 Pancreatic cancer rs2302761 T C 0.02653 0.61475 0.05272 Ulcerative colitis rs2302761 T C -0.0417 0.13653 0.02801 Crohn's disease rs2302761 T C -0.0251 0.60413 0.04832 Irrtable bowel syndrome rs2302761 T C -0.0114 0.58558 0.02092 Acute appendicitis rs2302761 T C 0.03295 0.00594 0.01198 Colon cancer rs2302761 T C -0.0033 0.91954 0.03292 Rectal cancer rs2302761 T C -0.0718 0.08554 0.04177 |
| Gastroesophageal reflux | rs2347526 | T | C | 0.01472 | 0.18125 | 0.01101 | Esophageal carcinoma | rs2347526 | T | C | -0.0019 | 0.97872 | 0.07114 | Acute gastritis | rs2347526 | T | C | -0.0029 | 0.93268 | 0.03483 | Chronic gastritis | rs2347526 | T | C | -0.0067 | 0.70564 | 0.01762 | Gastroduodenal ulcer | rs2347526 | T | C | 0.01317 | 0.46501 | 0.01802 | Gastric cancer | rs2347526 | T | C | 0.0175 | 0.70177 | 0.0457 | Fatty liver | rs2347526 | T | C | -0.0294 | 0.42126 | 0.03657 | Hepatic fibrosis | rs2347526 | T | C | -0.0826 | 0.55644 | 0.14047 | Cirrhosis | rs2347526 | T | C | -0.0257 | 0.34104 | 0.02699liver cancer rs2347526 T C -0.0799 0.20483 0.06303 Choleliths rs2347526 T C -0.0199 0.0321 0.00931 Acute pancreatitis rs2347526 T C -0.0191 0.37844 0.02169 Chronic pancreatitis rs2347526 T C -0.0056 0.84871 0.02928 Pancreatic cancer rs2347526 T C 0.01239 0.78494 0.0454 Ulcerative colitis rs2347526 T C 0.01589 0.50875 0.02405 Crohn's disease rs2347526 T C 0.03403 0.41161 0.04145 Irrtable bowel syndrome rs2347526 T C 0.02221 0.21725 0.018 Acute appendicitis rs2347526 T C 0.01216 0.23987 0.01034 Colon cancer rs2347526 T C -0.0088 0.75393 0.02816 Rectal cancer rs2347526 T C 0.0128 0.72078 0.0358 |
| Gastroesophageal reflux | rs2414072 | A | T | -0.0132 | 0.18279 | 0.00992 | Esophageal carcinoma | rs2414072 | A | T | 0.05309 | 0.4076 | 0.06411 | Acute gastritis | rs2414072 | A | T | -0.044 | 0.16052 | 0.03135 | Chronic gastritis | rs2414072 | A | T | -0.0176 | 0.26785 | 0.01585 | Gastroduodenal ulcer | rs2414072 | A | T | -0.0004 | 0.98276 | 0.01621 | Gastric cancer | rs2414072 | A | T | 0.02425 | 0.55574 | 0.04117 | Fatty liver | rs2414072 | A | T | 0.02735 | 0.40614 | 0.03293 | Hepatic fibrosis | rs2414072 | A | T | -0.0625 | 0.61882 | 0.12561 | Cirrhosis | rs2414072 | A | T | -0.0155 | 0.52147 | 0.02425liver cancer rs2414072 A T 0.05069 0.36983 0.05652 Choleliths rs2414072 A T 0.00181 0.8294 0.00839 Acute pancreatitis rs2414072 A T -0.0271 0.16554 0.01956 Chronic pancreatitis rs2414072 A T 0.01182 0.65406 0.02638 Pancreatic cancer rs2414072 A T 0.01968 0.62962 0.04081 Ulcerative colitis rs2414072 A T -0.0255 0.23962 0.02166 Crohn's disease rs2414072 A T 0.06722 0.07184 0.03734 Irrtable bowel syndrome rs2414072 A T 0.02118 0.19159 0.01622 Acute appendicitis rs2414072 A T -0.0045 0.62681 0.00932 Colon cancer rs2414072 A T 0.00937 0.71283 0.02545 Rectal cancer rs2414072 A T 0.02156 0.50407 0.03227 |
| Gastroesophageal reflux | rs242093 | A | G | 0.01302 | 0.19313 | 0.01001 | Esophageal carcinoma | rs242093 | A | G | -0.0535 | 0.40913 | 0.06479 | Acute gastritis | rs242093 | A | G | 0.064 | 0.04236 | 0.03153 | Chronic gastritis | rs242093 | A | G | 0.01395 | 0.38304 | 0.01599 | Gastroduodenal ulcer | rs242093 | A | G | -0.0139 | 0.3942 | 0.01636 | Gastric cancer | rs242093 | A | G | -0.0398 | 0.33852 | 0.04161 | Fatty liver | rs242093 | A | G | -0.0292 | 0.37908 | 0.03322 | Hepatic fibrosis | rs242093 | A | G | 0.18266 | 0.1492 | 0.12664 | Cirrhosis | rs242093 | A | G | 0.02314 | 0.34428 | 0.02447liver cancer rs242093 A G -0.0143 0.8011 0.05693 Choleliths rs242093 A G -0.007 0.4076 0.00846 Acute pancreatitis rs242093 A G 0.00217 0.91213 0.0197 Chronic pancreatitis rs242093 A G -0.0257 0.33412 0.02656 Pancreatic cancer rs242093 A G -0.0121 0.76787 0.04116 Ulcerative colitis rs242093 A G 0.00543 0.8033 0.02181 Crohn's disease rs242093 A G 0.0254 0.49959 0.03763 Irrtable bowel syndrome rs242093 A G 0.02767 0.09082 0.01636 Acute appendicitis rs242093 A G 0.01354 0.1493 0.00939 Colon cancer rs242093 A G 0.00055 0.98291 0.02564 Rectal cancer rs242093 A G -0.0047 0.88536 0.03255 |
| Gastroesophageal reflux | rs2447535 | A | G | -0.0039 | 0.69564 | 0.00995 | Esophageal carcinoma | rs2447535 | A | G | 0.01914 | 0.76696 | 0.06458 | Acute gastritis | rs2447535 | A | G | 0.03132 | 0.31955 | 0.03146 | Chronic gastritis | rs2447535 | A | G | -0.013 | 0.41359 | 0.01591 | Gastroduodenal ulcer | rs2447535 | A | G | 0.00605 | 0.7103 | 0.01628 | Gastric cancer | rs2447535 | A | G | -0.0082 | 0.84395 | 0.04145 | Fatty liver | rs2447535 | A | G | 0.02001 | 0.54513 | 0.03307 | Hepatic fibrosis | rs2447535 | A | G | 0.20144 | 0.1099 | 0.12601 | Cirrhosis | rs2447535 | A | G | 0.0021 | 0.9312 | 0.02435liver cancer rs2447535 A G 0.02241 0.69385 0.05692 Choleliths rs2447535 A G 0.00193 0.81811 0.00841 Acute pancreatitis rs2447535 A G -0.0149 0.44755 0.01963 Chronic pancreatitis rs2447535 A G 0.02163 0.41385 0.02647 Pancreatic cancer rs2447535 A G -0.0043 0.91676 0.04106 Ulcerative colitis rs2447535 A G 0.00792 0.71536 0.02173 Crohn's disease rs2447535 A G -0.0321 0.39148 0.03746 Irrtable bowel syndrome rs2447535 A G 0.00379 0.81585 0.01626 Acute appendicitis rs2447535 A G 0.00822 0.37911 0.00935 Colon cancer rs2447535 A G -0.0193 0.45121 0.0256 Rectal cancer rs2447535 A G 0.00865 0.78979 0.03247 |
| Gastroesophageal reflux | rs2478208 | C | G | 0.01082 | 0.27551 | 0.00993 | Esophageal carcinoma | rs2478208 | C | G | -0.0337 | 0.60063 | 0.06439 | Acute gastritis | rs2478208 | C | G | -0.0481 | 0.12535 | 0.03137 | Chronic gastritis | rs2478208 | C | G | 0.0008 | 0.95972 | 0.01586 | Gastroduodenal ulcer | rs2478208 | C | G | -0.0035 | 0.83005 | 0.01626 | Gastric cancer | rs2478208 | C | G | 0.00049 | 0.99054 | 0.04129 | Fatty liver | rs2478208 | C | G | 0.00608 | 0.85371 | 0.03299 | Hepatic fibrosis | rs2478208 | C | G | -0.0485 | 0.69996 | 0.12588 | Cirrhosis | rs2478208 | C | G | 0.02476 | 0.30827 | 0.02431liver cancer rs2478208 C G -0.0389 0.4934 0.05679 Choleliths rs2478208 C G 0.00773 0.35767 0.0084 Acute pancreatitis rs2478208 C G 0.01841 0.34745 0.01959 Chronic pancreatitis rs2478208 C G 0.04168 0.11439 0.0264 Pancreatic cancer rs2478208 C G 0.00661 0.87176 0.04094 Ulcerative colitis rs2478208 C G 0.01582 0.46557 0.02168 Crohn's disease rs2478208 C G -0.0466 0.21274 0.03739 Irrtable bowel syndrome rs2478208 C G -0.0056 0.72862 0.01621 Acute appendicitis rs2478208 C G 0.00224 0.81001 0.00933 Colon cancer rs2478208 C G -0.0122 0.63138 0.02545 Rectal cancer rs2478208 C G 0.02134 0.50957 0.03235 |
| Gastroesophageal reflux | rs2545798 | T | A | -0.0066 | 0.50574 | 0.00991 | Esophageal carcinoma | rs2545798 | T | A | -0.0618 | 0.3337 | 0.06398 | Acute gastritis | rs2545798 | T | A | 0.01008 | 0.74801 | 0.03137 | Chronic gastritis | rs2545798 | T | A | -0.0202 | 0.20197 | 0.01584 | Gastroduodenal ulcer | rs2545798 | T | A | -0.0058 | 0.72195 | 0.01621 | Gastric cancer | rs2545798 | T | A | 0.09374 | 0.02292 | 0.04121 | Fatty liver | rs2545798 | T | A | -0.0132 | 0.68777 | 0.03291 | Hepatic fibrosis | rs2545798 | T | A | 0.17425 | 0.16706 | 0.12611 | Cirrhosis | rs2545798 | T | A | -0.0361 | 0.13625 | 0.02424liver cancer rs2545798 T A 0.01366 0.80937 0.05664 Choleliths rs2545798 T A -0.0064 0.44404 0.00838 Acute pancreatitis rs2545798 T A -0.0032 0.86791 0.01953 Chronic pancreatitis rs2545798 T A -0.0168 0.52386 0.02635 Pancreatic cancer rs2545798 T A 0.02124 0.60351 0.04089 Ulcerative colitis rs2545798 T A -0.0212 0.3272 0.02164 Crohn's disease rs2545798 T A -0.047 0.20845 0.03733 Irrtable bowel syndrome rs2545798 T A -0.008 0.62026 0.01621 Acute appendicitis rs2545798 T A -0.0068 0.46432 0.00932 Colon cancer rs2545798 T A 0.02003 0.42991 0.02538 Rectal cancer rs2545798 T A 0.06371 0.04785 0.0322 |
| Gastroesophageal reflux | rs2554835 | G | A | 0.00416 | 0.67317 | 0.00985 | Esophageal carcinoma | rs2554835 | G | A | 0.02858 | 0.65452 | 0.06386 | Acute gastritis | rs2554835 | G | A | 0.04212 | 0.17592 | 0.03112 | Chronic gastritis | rs2554835 | G | A | 0.01495 | 0.34277 | 0.01576 | Gastroduodenal ulcer | rs2554835 | G | A | -0.0413 | 0.01024 | 0.0161 | Gastric cancer | rs2554835 | G | A | 0.10491 | 0.01069 | 0.0411 | Fatty liver | rs2554835 | G | A | 0.08763 | 0.00704 | 0.03252 | Hepatic fibrosis | rs2554835 | G | A | -0.0537 | 0.66674 | 0.12461 | Cirrhosis | rs2554835 | G | A | 0.00122 | 0.95965 | 0.0241liver cancer rs2554835 G A -0.0268 0.63393 0.05625 Choleliths rs2554835 G A 0.00888 0.28623 0.00833 Acute pancreatitis rs2554835 G A -0.0081 0.67613 0.01941 Chronic pancreatitis rs2554835 G A -0.0115 0.65973 0.02618 Pancreatic cancer rs2554835 G A -0.0405 0.31937 0.04066 Ulcerative colitis rs2554835 G A 0.03836 0.07437 0.0215 Crohn's disease rs2554835 G A 0.01394 0.70695 0.03708 Irrtable bowel syndrome rs2554835 G A 0.00573 0.72161 0.01608 Acute appendicitis rs2554835 G A -0.0145 0.11796 0.00925 Colon cancer rs2554835 G A -0.017 0.50333 0.02539 Rectal cancer rs2554835 G A 0.0135 0.67448 0.03215 |
| Gastroesophageal reflux | rs2570497 | C | T | -0.0099 | 0.33524 | 0.01029 | Esophageal carcinoma | rs2570497 | C | T | 0.04193 | 0.52996 | 0.06675 | Acute gastritis | rs2570497 | C | T | 0.01027 | 0.75176 | 0.03248 | Chronic gastritis | rs2570497 | C | T | -0.0356 | 0.03031 | 0.01642 | Gastroduodenal ulcer | rs2570497 | C | T | -0.0065 | 0.69736 | 0.01681 | Gastric cancer | rs2570497 | C | T | 0.05877 | 0.17086 | 0.04292 | Fatty liver | rs2570497 | C | T | -0.0222 | 0.51722 | 0.03422 | Hepatic fibrosis | rs2570497 | C | T | -0.1283 | 0.32573 | 0.13059 | Cirrhosis | rs2570497 | C | T | -0.0004 | 0.98842 | 0.02522liver cancer rs2570497 C T 0.05588 0.34251 0.05887 Choleliths rs2570497 C T 0.00151 0.86237 0.00869 Acute pancreatitis rs2570497 C T 0.01248 0.539 0.02031 Chronic pancreatitis rs2570497 C T -0.0125 0.64894 0.02742 Pancreatic cancer rs2570497 C T -0.0122 0.77331 0.04252 Ulcerative colitis rs2570497 C T 0.01467 0.51439 0.02251 Crohn's disease rs2570497 C T 0.06542 0.09203 0.03883 Irrtable bowel syndrome rs2570497 C T 0.02165 0.19798 0.01682 Acute appendicitis rs2570497 C T -0.0002 0.98305 0.00968 Colon cancer rs2570497 C T 0.01076 0.68365 0.02641 Rectal cancer rs2570497 C T 0.01994 0.55145 0.03348 |
| Gastroesophageal reflux | rs2725370 | T | C | 0.00378 | 0.73478 | 0.01115 | Esophageal carcinoma | rs2725370 | T | C | 0.09171 | 0.20457 | 0.07229 | Acute gastritis | rs2725370 | T | C | -0.0079 | 0.82201 | 0.03521 | Chronic gastritis | rs2725370 | T | C | 0.01737 | 0.32927 | 0.0178 | Gastroduodenal ulcer | rs2725370 | T | C | 0.00181 | 0.92109 | 0.01825 | Gastric cancer | rs2725370 | T | C | -0.0332 | 0.47324 | 0.04634 | Fatty liver | rs2725370 | T | C | 0.01992 | 0.59151 | 0.03712 | Hepatic fibrosis | rs2725370 | T | C | -0.034 | 0.81094 | 0.14223 | Cirrhosis | rs2725370 | T | C | 0.04641 | 0.09118 | 0.02747liver cancer rs2725370 T C -0.0026 0.96734 0.06414 Choleliths rs2725370 T C 0.00579 0.53894 0.00942 Acute pancreatitis rs2725370 T C 0.00854 0.69871 0.02207 Chronic pancreatitis rs2725370 T C -0.0032 0.91446 0.02982 Pancreatic cancer rs2725370 T C -0.0367 0.42693 0.04614 Ulcerative colitis rs2725370 T C -0.0019 0.93772 0.02444 Crohn's disease rs2725370 T C -0.0541 0.19901 0.04215 Irrtable bowel syndrome rs2725370 T C -0.0013 0.94087 0.0182 Acute appendicitis rs2725370 T C 0.00626 0.55109 0.01051 Colon cancer rs2725370 T C 0.01361 0.63422 0.02861 Rectal cancer rs2725370 T C -0.0249 0.49363 0.03634 |
| Gastroesophageal reflux | rs277828 | C | A | -0.0131 | 0.31631 | 0.01308 | Esophageal carcinoma | rs277828 | C | A | -0.0475 | 0.57423 | 0.08446 | Acute gastritis | rs277828 | C | A | -0.0624 | 0.13153 | 0.04139 | Chronic gastritis | rs277828 | C | A | 0.00203 | 0.92279 | 0.02093 | Gastroduodenal ulcer | rs277828 | C | A | -0.0116 | 0.59004 | 0.02146 | Gastric cancer | rs277828 | C | A | -0.0424 | 0.4362 | 0.05446 | Fatty liver | rs277828 | C | A | 0.00102 | 0.9813 | 0.04341 | Hepatic fibrosis | rs277828 | C | A | 0.0386 | 0.81657 | 0.16639 | Cirrhosis | rs277828 | C | A | -0.0543 | 0.09017 | 0.03205liver cancer rs277828 C A 0.21645 0.00506 0.07722 Choleliths rs277828 C A -0.0169 0.12705 0.01108 Acute pancreatitis rs277828 C A -0.0275 0.28671 0.02583 Chronic pancreatitis rs277828 C A -0.0405 0.24487 0.03485 Pancreatic cancer rs277828 C A 0.01559 0.77222 0.05386 Ulcerative colitis rs277828 C A -0.0176 0.53818 0.02851 Crohn's disease rs277828 C A -0.0102 0.83592 0.04907 Irrtable bowel syndrome rs277828 C A -0.0129 0.54654 0.02136 Acute appendicitis rs277828 C A -0.0057 0.64413 0.01229 Colon cancer rs277828 C A 0.019 0.57121 0.03356 Rectal cancer rs277828 C A 0.00019 0.99643 0.04265 |
| Gastroesophageal reflux | rs2819336 | T | C | 0.01956 | 0.05282 | 0.0101 | Esophageal carcinoma | rs2819336 | T | C | -0.0096 | 0.88379 | 0.06561 | Acute gastritis | rs2819336 | T | C | 0.00205 | 0.94865 | 0.03185 | Chronic gastritis | rs2819336 | T | C | -0.0133 | 0.40976 | 0.01615 | Gastroduodenal ulcer | rs2819336 | T | C | -0.002 | 0.90365 | 0.01653 | Gastric cancer | rs2819336 | T | C | -0.0157 | 0.70848 | 0.04202 | Fatty liver | rs2819336 | T | C | 0.00247 | 0.94136 | 0.03357 | Hepatic fibrosis | rs2819336 | T | C | 0.03528 | 0.78348 | 0.12838 | Cirrhosis | rs2819336 | T | C | -0.0435 | 0.07852 | 0.0247liver cancer rs2819336 T C 0.08038 0.16428 0.0578 Choleliths rs2819336 T C 0.002 0.81503 0.00854 Acute pancreatitis rs2819336 T C -0.0271 0.17441 0.01992 Chronic pancreatitis rs2819336 T C -0.0388 0.14869 0.02685 Pancreatic cancer rs2819336 T C 0.00811 0.84565 0.04164 Ulcerative colitis rs2819336 T C -0.0117 0.59662 0.02204 Crohn's disease rs2819336 T C 0.01642 0.66566 0.03801 Irrtable bowel syndrome rs2819336 T C -0.007 0.672 0.01651 Acute appendicitis rs2819336 T C -0.0042 0.65581 0.00949 Colon cancer rs2819336 T C 0.03648 0.16042 0.02599 Rectal cancer rs2819336 T C -0.0202 0.54005 0.03292 |
| Gastroesophageal reflux | rs2820314 | A | C | 0.00758 | 0.48067 | 0.01075 | Esophageal carcinoma | rs2820314 | A | C | -0.0407 | 0.56018 | 0.06981 | Acute gastritis | rs2820314 | A | C | 0.01114 | 0.74214 | 0.03387 | Chronic gastritis | rs2820314 | A | C | -0.0096 | 0.57738 | 0.01719 | Gastroduodenal ulcer | rs2820314 | A | C | -0.0118 | 0.50212 | 0.01757 | Gastric cancer | rs2820314 | A | C | 0.04615 | 0.30354 | 0.04485 | Fatty liver | rs2820314 | A | C | -0.024 | 0.50043 | 0.03562 | Hepatic fibrosis | rs2820314 | A | C | 0.07393 | 0.58631 | 0.13585 | Cirrhosis | rs2820314 | A | C | -0.0121 | 0.64424 | 0.02628liver cancer rs2820314 A C -0.1059 0.08562 0.0616 Choleliths rs2820314 A C -0.0093 0.30636 0.00909 Acute pancreatitis rs2820314 A C 0.00876 0.67952 0.02119 Chronic pancreatitis rs2820314 A C 0.03887 0.17374 0.02857 Pancreatic cancer rs2820314 A C -0.0067 0.88011 0.04436 Ulcerative colitis rs2820314 A C 0.0042 0.85777 0.02341 Crohn's disease rs2820314 A C -0.0883 0.0287 0.04037 Irrtable bowel syndrome rs2820314 A C 0.01264 0.47166 0.01756 Acute appendicitis rs2820314 A C 0.00398 0.69263 0.01008 Colon cancer rs2820314 A C -0.0242 0.38218 0.02764 Rectal cancer rs2820314 A C -0.0152 0.6648 0.03509 |
| Gastroesophageal reflux | rs28373063 | G | C | 0.02458 | 0.10812 | 0.0153 | Esophageal carcinoma | rs28373063 | G | C | -0.0307 | 0.75562 | 0.09858 | Acute gastritis | rs28373063 | G | C | -0.0708 | 0.14413 | 0.04844 | Chronic gastritis | rs28373063 | G | C | 0.00036 | 0.98809 | 0.02444 | Gastroduodenal ulcer | rs28373063 | G | C | 0.01061 | 0.67182 | 0.02503 | Gastric cancer | rs28373063 | G | C | -0.0498 | 0.43284 | 0.06354 | Fatty liver | rs28373063 | G | C | -0.0159 | 0.75345 | 0.05072 | Hepatic fibrosis | rs28373063 | G | C | 0.12123 | 0.53325 | 0.19458 | Cirrhosis | rs28373063 | G | C | 0.01789 | 0.63243 | 0.03741liver cancer rs28373063 G C -0.0275 0.75303 0.08754 Choleliths rs28373063 G C -0.0145 0.26161 0.01296 Acute pancreatitis rs28373063 G C 0.04098 0.17438 0.03017 Chronic pancreatitis rs28373063 G C -0.0057 0.88889 0.04071 Pancreatic cancer rs28373063 G C -0.0729 0.24734 0.06303 Ulcerative colitis rs28373063 G C -0.0104 0.75611 0.03335 Crohn's disease rs28373063 G C 0.03995 0.48664 0.05742 Irrtable bowel syndrome rs28373063 G C -0.0074 0.76843 0.0251 Acute appendicitis rs28373063 G C 0.00354 0.80558 0.0144 Colon cancer rs28373063 G C 0.02235 0.56769 0.0391 Rectal cancer rs28373063 G C 0.07055 0.15537 0.04965 |
| Gastroesophageal reflux | rs28513670 | G | A | -0.0241 | 0.03264 | 0.01129 | Esophageal carcinoma | rs28513670 | G | A | 0.00911 | 0.90067 | 0.07297 | Acute gastritis | rs28513670 | G | A | -0.0347 | 0.32852 | 0.03555 | Chronic gastritis | rs28513670 | G | A | -0.0418 | 0.02062 | 0.01804 | Gastroduodenal ulcer | rs28513670 | G | A | -0.0153 | 0.40706 | 0.0184 | Gastric cancer | rs28513670 | G | A | -0.0144 | 0.75844 | 0.04696 | Fatty liver | rs28513670 | G | A | -0.0704 | 0.06039 | 0.03749 | Hepatic fibrosis | rs28513670 | G | A | -0.05 | 0.72512 | 0.14231 | Cirrhosis | rs28513670 | G | A | -0.0208 | 0.45033 | 0.02749liver cancer rs28513670 G A 0.07532 0.24123 0.06427 Choleliths rs28513670 G A -0.0282 0.00323 0.00957 Acute pancreatitis rs28513670 G A -0.0431 0.05191 0.02216 Chronic pancreatitis rs28513670 G A -0.0079 0.79122 0.02989 Pancreatic cancer rs28513670 G A -0.0845 0.06801 0.0463 Ulcerative colitis rs28513670 G A 0.01294 0.59908 0.02461 Crohn's disease rs28513670 G A 0.01359 0.749 0.04247 Irrtable bowel syndrome rs28513670 G A -0.0251 0.17434 0.01846 Acute appendicitis rs28513670 G A -0.0248 0.01915 0.01059 Colon cancer rs28513670 G A 0.01009 0.72736 0.02893 Rectal cancer rs28513670 G A 0.04746 0.19646 0.03674 |
| Gastroesophageal reflux | rs2885198 | A | G | 0.0198 | 0.04374 | 0.00982 | Esophageal carcinoma | rs2885198 | A | G | -0.0217 | 0.73232 | 0.06345 | Acute gastritis | rs2885198 | A | G | 0.0165 | 0.59457 | 0.03099 | Chronic gastritis | rs2885198 | A | G | 0.03255 | 0.03782 | 0.01568 | Gastroduodenal ulcer | rs2885198 | A | G | 0.01483 | 0.35533 | 0.01604 | Gastric cancer | rs2885198 | A | G | -0.0781 | 0.0558 | 0.04085 | Fatty liver | rs2885198 | A | G | 0.00442 | 0.89199 | 0.03256 | Hepatic fibrosis | rs2885198 | A | G | -0.036 | 0.77184 | 0.12426 | Cirrhosis | rs2885198 | A | G | -0.0258 | 0.28216 | 0.02397liver cancer rs2885198 A G -0.0241 0.66605 0.05585 Choleliths rs2885198 A G 0.00954 0.25001 0.00829 Acute pancreatitis rs2885198 A G -0.0054 0.78114 0.01932 Chronic pancreatitis rs2885198 A G -0.0358 0.16995 0.02606 Pancreatic cancer rs2885198 A G -0.0123 0.75991 0.04039 Ulcerative colitis rs2885198 A G -0.0061 0.77501 0.02143 Crohn's disease rs2885198 A G -0.0185 0.61752 0.037 Irrtable bowel syndrome rs2885198 A G 0.00222 0.88976 0.01605 Acute appendicitis rs2885198 A G -0.0072 0.43518 0.00922 Colon cancer rs2885198 A G 0.02782 0.26839 0.02514 Rectal cancer rs2885198 A G 0.03918 0.21981 0.03193 |
| Gastroesophageal reflux | rs2901616 | A | G | -0.0216 | 0.02818 | 0.00983 | Esophageal carcinoma | rs2901616 | A | G | -0.0875 | 0.16871 | 0.06354 | Acute gastritis | rs2901616 | A | G | -0.0188 | 0.5453 | 0.03102 | Chronic gastritis | rs2901616 | A | G | -0.018 | 0.25033 | 0.0157 | Gastroduodenal ulcer | rs2901616 | A | G | -0.0051 | 0.7498 | 0.01606 | Gastric cancer | rs2901616 | A | G | 0.01915 | 0.63927 | 0.04085 | Fatty liver | rs2901616 | A | G | -0.0163 | 0.61763 | 0.03266 | Hepatic fibrosis | rs2901616 | A | G | 0.09428 | 0.44866 | 0.12443 | Cirrhosis | rs2901616 | A | G | -0.0197 | 0.41249 | 0.02404liver cancer rs2901616 A G 0.02862 0.60892 0.05593 Choleliths rs2901616 A G -0.013 0.11672 0.00831 Acute pancreatitis rs2901616 A G -0.0379 0.05028 0.01937 Chronic pancreatitis rs2901616 A G -0.0154 0.55623 0.02611 Pancreatic cancer rs2901616 A G 0.02062 0.61001 0.04042 Ulcerative colitis rs2901616 A G -0.0169 0.43036 0.02148 Crohn's disease rs2901616 A G 0.00709 0.8481 0.03703 Irrtable bowel syndrome rs2901616 A G -0.0077 0.63058 0.01608 Acute appendicitis rs2901616 A G 0.0073 0.42943 0.00924 Colon cancer rs2901616 A G -0.0007 0.97845 0.02516 Rectal cancer rs2901616 A G -0.0008 0.97992 0.03195 |
| Gastroesophageal reflux | rs2905426 | G | T | -0.0006 | 0.95891 | 0.01092 | Esophageal carcinoma | rs2905426 | G | T | -0.0946 | 0.18228 | 0.07094 | Acute gastritis | rs2905426 | G | T | -0.0026 | 0.94006 | 0.03443 | Chronic gastritis | rs2905426 | G | T | 0.03577 | 0.04028 | 0.01744 | Gastroduodenal ulcer | rs2905426 | G | T | -0.0154 | 0.38794 | 0.01785 | Gastric cancer | rs2905426 | G | T | 0.08563 | 0.06005 | 0.04554 | Fatty liver | rs2905426 | G | T | 0.0992 | 0.00525 | 0.03554 | Hepatic fibrosis | rs2905426 | G | T | -0.1863 | 0.17907 | 0.13862 | Cirrhosis | rs2905426 | G | T | 0.00158 | 0.9529 | 0.02674liver cancer rs2905426 G T 0.15126 0.01545 0.06246 Choleliths rs2905426 G T -0.0038 0.68264 0.00923 Acute pancreatitis rs2905426 G T -0.0194 0.36802 0.02156 Chronic pancreatitis rs2905426 G T -0.007 0.81057 0.02909 Pancreatic cancer rs2905426 G T 0.02312 0.60883 0.04518 Ulcerative colitis rs2905426 G T 0.05617 0.01869 0.02389 Crohn's disease rs2905426 G T -0.0061 0.88199 0.0412 Irrtable bowel syndrome rs2905426 G T 0.01952 0.27431 0.01786 Acute appendicitis rs2905426 G T 0.00686 0.50396 0.01027 Colon cancer rs2905426 G T -0.0378 0.17806 0.0281 Rectal cancer rs2905426 G T 0.0025 0.94398 0.0356 |
| Gastroesophageal reflux | rs2923431 | C | G | -0.0004 | 0.97198 | 0.01018 | Esophageal carcinoma | rs2923431 | C | G | 0.05382 | 0.41444 | 0.06595 | Acute gastritis | rs2923431 | C | G | 0.00963 | 0.76489 | 0.03219 | Chronic gastritis | rs2923431 | C | G | -0.0142 | 0.38291 | 0.01628 | Gastroduodenal ulcer | rs2923431 | C | G | 0.00317 | 0.84902 | 0.01667 | Gastric cancer | rs2923431 | C | G | -0.0365 | 0.38822 | 0.04231 | Fatty liver | rs2923431 | C | G | -0.007 | 0.83537 | 0.03379 | Hepatic fibrosis | rs2923431 | C | G | -0.1549 | 0.23047 | 0.12919 | Cirrhosis | rs2923431 | C | G | -0.0212 | 0.3948 | 0.02494liver cancer rs2923431 C G 0.0904 0.12096 0.05829 Choleliths rs2923431 C G 0.00656 0.44642 0.00861 Acute pancreatitis rs2923431 C G -0.0069 0.73065 0.02011 Chronic pancreatitis rs2923431 C G -0.0111 0.68156 0.02709 Pancreatic cancer rs2923431 C G -0.0015 0.97107 0.042 Ulcerative colitis rs2923431 C G -0.0183 0.41058 0.02223 Crohn's disease rs2923431 C G -0.0555 0.14751 0.03835 Irrtable bowel syndrome rs2923431 C G 0.01722 0.30108 0.01665 Acute appendicitis rs2923431 C G 0.00179 0.85137 0.00957 Colon cancer rs2923431 C G -0.003 0.91013 0.02616 Rectal cancer rs2923431 C G -0.0017 0.95981 0.03317 |
| Gastroesophageal reflux | rs2971970 | T | G | 0.00093 | 0.94423 | 0.0133 | Esophageal carcinoma | rs2971970 | T | G | -0.1283 | 0.13626 | 0.08609 | Acute gastritis | rs2971970 | T | G | 0.04243 | 0.31323 | 0.04207 | Chronic gastritis | rs2971970 | T | G | 0.0428 | 0.04373 | 0.02122 | Gastroduodenal ulcer | rs2971970 | T | G | 0.01845 | 0.39587 | 0.02173 | Gastric cancer | rs2971970 | T | G | -0.057 | 0.3024 | 0.05528 | Fatty liver | rs2971970 | T | G | 0.08604 | 0.05118 | 0.04412 | Hepatic fibrosis | rs2971970 | T | G | 0.06789 | 0.68764 | 0.16885 | Cirrhosis | rs2971970 | T | G | 0.00907 | 0.78035 | 0.03253liver cancer rs2971970 T G 0.00346 0.96349 0.07558 Choleliths rs2971970 T G 0.00695 0.53627 0.01124 Acute pancreatitis rs2971970 T G 0.0185 0.48089 0.02625 Chronic pancreatitis rs2971970 T G 0.052 0.14149 0.03537 Pancreatic cancer rs2971970 T G -0.094 0.08552 0.05464 Ulcerative colitis rs2971970 T G -0.0152 0.60247 0.0291 Crohn's disease rs2971970 T G -0.0312 0.53258 0.05007 Irrtable bowel syndrome rs2971970 T G 0.01341 0.53785 0.02177 Acute appendicitis rs2971970 T G -0.0142 0.25593 0.0125 Colon cancer rs2971970 T G -0.0266 0.43507 0.03403 Rectal cancer rs2971970 T G 0.06599 0.12627 0.04316 |
| Gastroesophageal reflux | rs2998315 | G | A | -0.0016 | 0.87148 | 0.00987 | Esophageal carcinoma | rs2998315 | G | A | 0.01969 | 0.75762 | 0.0638 | Acute gastritis | rs2998315 | G | A | -0.019 | 0.54254 | 0.03113 | Chronic gastritis | rs2998315 | G | A | 0.00663 | 0.67394 | 0.01575 | Gastroduodenal ulcer | rs2998315 | G | A | 0.02979 | 0.06481 | 0.01613 | Gastric cancer | rs2998315 | G | A | 0.08722 | 0.0338 | 0.0411 | Fatty liver | rs2998315 | G | A | -0.0129 | 0.69504 | 0.0328 | Hepatic fibrosis | rs2998315 | G | A | 0.11021 | 0.37739 | 0.12485 | Cirrhosis | rs2998315 | G | A | 0.00149 | 0.95072 | 0.02413liver cancer rs2998315 G A 0.05838 0.30001 0.05633 Choleliths rs2998315 G A 0.00677 0.4169 0.00834 Acute pancreatitis rs2998315 G A 0.02814 0.14786 0.01944 Chronic pancreatitis rs2998315 G A -0.0133 0.6126 0.0262 Pancreatic cancer rs2998315 G A 0.08737 0.03169 0.04067 Ulcerative colitis rs2998315 G A 0.02939 0.17271 0.02155 Crohn's disease rs2998315 G A -0.0066 0.85852 0.03724 Irrtable bowel syndrome rs2998315 G A 0.01877 0.24397 0.01611 Acute appendicitis rs2998315 G A -0.0097 0.29581 0.00927 Colon cancer rs2998315 G A 0.01496 0.55458 0.02533 Rectal cancer rs2998315 G A 0.02708 0.39979 0.03216 |
| Gastroesophageal reflux | rs3013014 | G | A | 0.00195 | 0.84652 | 0.01006 | Esophageal carcinoma | rs3013014 | G | A | 0.05847 | 0.36865 | 0.06504 | Acute gastritis | rs3013014 | G | A | -0.0111 | 0.7255 | 0.03174 | Chronic gastritis | rs3013014 | G | A | 0.00385 | 0.81081 | 0.01608 | Gastroduodenal ulcer | rs3013014 | G | A | -0.0002 | 0.98934 | 0.01644 | Gastric cancer | rs3013014 | G | A | -0.0478 | 0.253 | 0.04185 | Fatty liver | rs3013014 | G | A | -0.0407 | 0.2231 | 0.03338 | Hepatic fibrosis | rs3013014 | G | A | 0.17909 | 0.16005 | 0.12747 | Cirrhosis | rs3013014 | G | A | -0.0401 | 0.10328 | 0.0246liver cancer rs3013014 G A -0.0321 0.57617 0.05741 Choleliths rs3013014 G A 0.0059 0.48778 0.00851 Acute pancreatitis rs3013014 G A -0.0091 0.64506 0.01984 Chronic pancreatitis rs3013014 G A 0.01718 0.52066 0.02675 Pancreatic cancer rs3013014 G A 0.07807 0.05954 0.04143 Ulcerative colitis rs3013014 G A 0.01018 0.64256 0.02194 Crohn's disease rs3013014 G A -0.0209 0.58119 0.03785 Irrtable bowel syndrome rs3013014 G A -0.0101 0.53788 0.01643 Acute appendicitis rs3013014 G A 0.00037 0.96843 0.00944 Colon cancer rs3013014 G A 0.02854 0.26861 0.0258 Rectal cancer rs3013014 G A -0.0063 0.84795 0.03273 |
| Gastroesophageal reflux | rs301800 | T | C | -0.0104 | 0.42559 | 0.01308 | Esophageal carcinoma | rs301800 | T | C | -0.0495 | 0.55645 | 0.08422 | Acute gastritis | rs301800 | T | C | 0.00102 | 0.98026 | 0.04121 | Chronic gastritis | rs301800 | T | C | 0.01889 | 0.36597 | 0.02089 | Gastroduodenal ulcer | rs301800 | T | C | 0.01445 | 0.49874 | 0.02137 | Gastric cancer | rs301800 | T | C | -0.0297 | 0.58349 | 0.05413 | Fatty liver | rs301800 | T | C | 0.05679 | 0.19022 | 0.04335 | Hepatic fibrosis | rs301800 | T | C | 0.07066 | 0.67137 | 0.16655 | Cirrhosis | rs301800 | T | C | -0.0302 | 0.3463 | 0.03204liver cancer rs301800 T C -0.0431 0.56312 0.07449 Choleliths rs301800 T C 0.0141 0.20136 0.01104 Acute pancreatitis rs301800 T C 0.04317 0.09387 0.02577 Chronic pancreatitis rs301800 T C 0.05333 0.12536 0.0348 Pancreatic cancer rs301800 T C -0.0037 0.94542 0.05362 Ulcerative colitis rs301800 T C 0.05041 0.07763 0.02857 Crohn's disease rs301800 T C -0.1059 0.03142 0.04922 Irrtable bowel syndrome rs301800 T C 0.03749 0.0794 0.02137 Acute appendicitis rs301800 T C -0.0126 0.30502 0.01227 Colon cancer rs301800 T C 0.03017 0.36807 0.03351 Rectal cancer rs301800 T C 0.00246 0.95381 0.04248 |
| Gastroesophageal reflux | rs3026996 | A | C | 0.01243 | 0.29338 | 0.01183 | Esophageal carcinoma | rs3026996 | A | C | -0.1095 | 0.15263 | 0.07656 | Acute gastritis | rs3026996 | A | C | -0.058 | 0.12077 | 0.03737 | Chronic gastritis | rs3026996 | A | C | 0.00241 | 0.89816 | 0.01885 | Gastroduodenal ulcer | rs3026996 | A | C | 0.00921 | 0.63381 | 0.01934 | Gastric cancer | rs3026996 | A | C | -0.0476 | 0.33077 | 0.04894 | Fatty liver | rs3026996 | A | C | -0.0055 | 0.88963 | 0.03937 | Hepatic fibrosis | rs3026996 | A | C | -0.0487 | 0.74765 | 0.15138 | Cirrhosis | rs3026996 | A | C | 0.00464 | 0.87294 | 0.02902liver cancer rs3026996 A C 0.01313 0.84553 0.06742 Choleliths rs3026996 A C -0.0248 0.01296 0.00999 Acute pancreatitis rs3026996 A C -0.0554 0.01765 0.02336 Chronic pancreatitis rs3026996 A C -0.0182 0.56578 0.03161 Pancreatic cancer rs3026996 A C 0.01609 0.74145 0.04877 Ulcerative colitis rs3026996 A C 0.01352 0.60239 0.02595 Crohn's disease rs3026996 A C -0.0375 0.4024 0.04479 Irrtable bowel syndrome rs3026996 A C 0.0093 0.63005 0.01931 Acute appendicitis rs3026996 A C 0.0108 0.33304 0.01115 Colon cancer rs3026996 A C -0.0177 0.55628 0.03008 Rectal cancer rs3026996 A C -0.0171 0.65535 0.03822 |
| Gastroesophageal reflux | rs31940 | G | A | 0.01587 | 0.31961 | 0.01595 | Esophageal carcinoma | rs31940 | G | A | -0.06 | 0.56526 | 0.10426 | Acute gastritis | rs31940 | G | A | 0.03552 | 0.48055 | 0.05035 | Chronic gastritis | rs31940 | G | A | -0.0257 | 0.31279 | 0.02545 | Gastroduodenal ulcer | rs31940 | G | A | 0.07316 | 0.00601 | 0.02663 | Gastric cancer | rs31940 | G | A | -0.0069 | 0.9178 | 0.06722 | Fatty liver | rs31940 | G | A | 0.06823 | 0.19669 | 0.05285 | Hepatic fibrosis | rs31940 | G | A | 0.26084 | 0.19325 | 0.20048 | Cirrhosis | rs31940 | G | A | -0.0409 | 0.29498 | 0.03905liver cancer rs31940 G A -0.1882 0.03969 0.09148 Choleliths rs31940 G A 0.00338 0.80266 0.01354 Acute pancreatitis rs31940 G A -0.0685 0.02974 0.03153 Chronic pancreatitis rs31940 G A -0.0342 0.42067 0.04251 Pancreatic cancer rs31940 G A 0.00728 0.91194 0.06581 Ulcerative colitis rs31940 G A 0.06834 0.04927 0.03475 Crohn's disease rs31940 G A 0.07004 0.24324 0.06002 Irrtable bowel syndrome rs31940 G A 0.04193 0.10814 0.0261 Acute appendicitis rs31940 G A -0.003 0.84231 0.015 Colon cancer rs31940 G A -0.0147 0.72086 0.04126 Rectal cancer rs31940 G A 0.03867 0.46066 0.05241 |
| Gastroesophageal reflux | rs320693 | C | G | -0.0065 | 0.50529 | 0.00981 | Esophageal carcinoma | rs320693 | C | G | -0.0854 | 0.17835 | 0.06345 | Acute gastritis | rs320693 | C | G | 0.00571 | 0.85352 | 0.03095 | Chronic gastritis | rs320693 | C | G | -0.0004 | 0.9799 | 0.01567 | Gastroduodenal ulcer | rs320693 | C | G | -0.0183 | 0.2547 | 0.01603 | Gastric cancer | rs320693 | C | G | -0.0313 | 0.44282 | 0.04082 | Fatty liver | rs320693 | C | G | -0.0461 | 0.15678 | 0.03258 | Hepatic fibrosis | rs320693 | C | G | -0.107 | 0.38939 | 0.12431 | Cirrhosis | rs320693 | C | G | -0.0544 | 0.02343 | 0.02401liver cancer rs320693 C G -0.0545 0.32962 0.05592 Choleliths rs320693 C G -0.0152 0.06679 0.00829 Acute pancreatitis rs320693 C G 0.02827 0.1439 0.01935 Chronic pancreatitis rs320693 C G 0.04178 0.10918 0.02608 Pancreatic cancer rs320693 C G 0.02819 0.48512 0.04038 Ulcerative colitis rs320693 C G 0.00537 0.80207 0.02143 Crohn's disease rs320693 C G -0.0046 0.89984 0.03693 Irrtable bowel syndrome rs320693 C G 0.01098 0.49321 0.01603 Acute appendicitis rs320693 C G 0.00796 0.38775 0.00922 Colon cancer rs320693 C G -0.0031 0.90056 0.02514 Rectal cancer rs320693 C G 0.01155 0.71729 0.03191 |
| Gastroesophageal reflux | rs337637 | G | A | 0.00384 | 0.70439 | 0.01011 | Esophageal carcinoma | rs337637 | G | A | 0.03821 | 0.55816 | 0.06526 | Acute gastritis | rs337637 | G | A | -0.0085 | 0.7904 | 0.03184 | Chronic gastritis | rs337637 | G | A | -0.0014 | 0.92964 | 0.01615 | Gastroduodenal ulcer | rs337637 | G | A | -0.0014 | 0.93144 | 0.01652 | Gastric cancer | rs337637 | G | A | -0.0025 | 0.95235 | 0.04196 | Fatty liver | rs337637 | G | A | -0.0025 | 0.94091 | 0.03357 | Hepatic fibrosis | rs337637 | G | A | 0.02569 | 0.84104 | 0.12809 | Cirrhosis | rs337637 | G | A | 0.00087 | 0.97204 | 0.02472liver cancer rs337637 G A -0.0409 0.47691 0.05749 Choleliths rs337637 G A 0.01013 0.23559 0.00854 Acute pancreatitis rs337637 G A 0.04385 0.02781 0.01993 Chronic pancreatitis rs337637 G A 0.00723 0.7876 0.02685 Pancreatic cancer rs337637 G A -0.036 0.38585 0.04146 Ulcerative colitis rs337637 G A -0.0022 0.92131 0.02208 Crohn's disease rs337637 G A 0.01821 0.63308 0.03815 Irrtable bowel syndrome rs337637 G A -0.005 0.76172 0.01652 Acute appendicitis rs337637 G A 0.00717 0.44995 0.0095 Colon cancer rs337637 G A 0.01342 0.60416 0.02588 Rectal cancer rs337637 G A 0.0033 0.91992 0.03283 |
| Gastroesophageal reflux | rs34316 | C | A | 0.00682 | 0.49173 | 0.00992 | Esophageal carcinoma | rs34316 | C | A | -0.0273 | 0.67011 | 0.0642 | Acute gastritis | rs34316 | C | A | -0.0439 | 0.16107 | 0.03134 | Chronic gastritis | rs34316 | C | A | -0.0069 | 0.66243 | 0.01586 | Gastroduodenal ulcer | rs34316 | C | A | -0.0138 | 0.39479 | 0.01623 | Gastric cancer | rs34316 | C | A | 0.06095 | 0.14014 | 0.04131 | Fatty liver | rs34316 | C | A | -0.0139 | 0.6733 | 0.03297 | Hepatic fibrosis | rs34316 | C | A | -0.0913 | 0.46908 | 0.12608 | Cirrhosis | rs34316 | C | A | 0.00943 | 0.69773 | 0.02429liver cancer rs34316 C A 0.00688 0.90336 0.05663 Choleliths rs34316 C A -0.0032 0.7054 0.00839 Acute pancreatitis rs34316 C A -0.0113 0.56477 0.01956 Chronic pancreatitis rs34316 C A 0.01227 0.64222 0.02641 Pancreatic cancer rs34316 C A 0.0272 0.50516 0.04081 Ulcerative colitis rs34316 C A 0.01794 0.40811 0.02168 Crohn's disease rs34316 C A 0.02431 0.51508 0.03735 Irrtable bowel syndrome rs34316 C A 0.01415 0.38271 0.01621 Acute appendicitis rs34316 C A 0.0048 0.60669 0.00933 Colon cancer rs34316 C A 0.00678 0.78972 0.02543 Rectal cancer rs34316 C A -0.0231 0.47445 0.03228 |
| Gastroesophageal reflux | rs34485537 | T | C | 0.00224 | 0.8207 | 0.00987 | Esophageal carcinoma | rs34485537 | T | C | -0.0192 | 0.76279 | 0.06377 | Acute gastritis | rs34485537 | T | C | -0.0586 | 0.06044 | 0.0312 | Chronic gastritis | rs34485537 | T | C | -0.017 | 0.28195 | 0.01578 | Gastroduodenal ulcer | rs34485537 | T | C | -0.0109 | 0.49969 | 0.01615 | Gastric cancer | rs34485537 | T | C | -0.0183 | 0.65607 | 0.04113 | Fatty liver | rs34485537 | T | C | -0.0527 | 0.10743 | 0.03276 | Hepatic fibrosis | rs34485537 | T | C | -0.0651 | 0.60252 | 0.12504 | Cirrhosis | rs34485537 | T | C | -0.0388 | 0.10726 | 0.02412liver cancer rs34485537 T C 0.08363 0.13706 0.05625 Choleliths rs34485537 T C -0.0166 0.04719 0.00835 Acute pancreatitis rs34485537 T C -0.0311 0.10962 0.01944 Chronic pancreatitis rs34485537 T C -0.0225 0.3917 0.02621 Pancreatic cancer rs34485537 T C -0.0059 0.885 0.04059 Ulcerative colitis rs34485537 T C -0.0394 0.06759 0.02155 Crohn's disease rs34485537 T C 0.01146 0.75796 0.03719 Irrtable bowel syndrome rs34485537 T C -0.0255 0.1129 0.01611 Acute appendicitis rs34485537 T C -0.0037 0.68901 0.00927 Colon cancer rs34485537 T C 0.01517 0.5492 0.02533 Rectal cancer rs34485537 T C 0.01406 0.66219 0.03218 |
| Gastroesophageal reflux | rs34853711 | C | G | -0.0012 | 0.92863 | 0.01343 | Esophageal carcinoma | rs34853711 | C | G | -0.0747 | 0.38796 | 0.08658 | Acute gastritis | rs34853711 | C | G | -0.0385 | 0.36525 | 0.04258 | Chronic gastritis | rs34853711 | C | G | 0.01089 | 0.61136 | 0.02143 | Gastroduodenal ulcer | rs34853711 | C | G | -0.0254 | 0.24946 | 0.02202 | Gastric cancer | rs34853711 | C | G | 0.01048 | 0.85065 | 0.05567 | Fatty liver | rs34853711 | C | G | 0.01717 | 0.70013 | 0.04459 | Hepatic fibrosis | rs34853711 | C | G | 0.09804 | 0.56667 | 0.17111 | Cirrhosis | rs34853711 | C | G | -0.0188 | 0.56775 | 0.03294liver cancer rs34853711 C G -0.0254 0.7396 0.07655 Choleliths rs34853711 C G -0.0163 0.15018 0.01136 Acute pancreatitis rs34853711 C G -8E-05 0.99769 0.02651 Chronic pancreatitis rs34853711 C G -0.0205 0.56814 0.03585 Pancreatic cancer rs34853711 C G -0.0693 0.21048 0.05533 Ulcerative colitis rs34853711 C G 0.04659 0.11255 0.02936 Crohn's disease rs34853711 C G 0.07046 0.16311 0.05052 Irrtable bowel syndrome rs34853711 C G -0.0084 0.70221 0.02198 Acute appendicitis rs34853711 C G 0.00498 0.69411 0.01266 Colon cancer rs34853711 C G -0.0716 0.03685 0.03431 Rectal cancer rs34853711 C G 0.0186 0.6693 0.04354 |
| Gastroesophageal reflux | rs35309068 | G | T | -0.0169 | 0.08617 | 0.00986 | Esophageal carcinoma | rs35309068 | G | T | 0.01764 | 0.78187 | 0.06371 | Acute gastritis | rs35309068 | G | T | 0.03119 | 0.31635 | 0.03113 | Chronic gastritis | rs35309068 | G | T | 0.01503 | 0.33999 | 0.01575 | Gastroduodenal ulcer | rs35309068 | G | T | 0.02193 | 0.1736 | 0.01612 | Gastric cancer | rs35309068 | G | T | -0.0216 | 0.59868 | 0.04099 | Fatty liver | rs35309068 | G | T | -0.0156 | 0.63304 | 0.03274 | Hepatic fibrosis | rs35309068 | G | T | -0.2864 | 0.01236 | 0.11447 | Cirrhosis | rs35309068 | G | T | -0.0245 | 0.30986 | 0.02411liver cancer rs35309068 G T -0.0031 0.95603 0.05625 Choleliths rs35309068 G T 0.01557 0.06175 0.00833 Acute pancreatitis rs35309068 G T -0.0037 0.84986 0.01944 Chronic pancreatitis rs35309068 G T 0.00365 0.88909 0.0262 Pancreatic cancer rs35309068 G T -0.0031 0.93851 0.04064 Ulcerative colitis rs35309068 G T -0.0162 0.45129 0.02153 Crohn's disease rs35309068 G T -0.0038 0.9176 0.03715 Irrtable bowel syndrome rs35309068 G T 0.00792 0.62307 0.01612 Acute appendicitis rs35309068 G T 0.00203 0.82671 0.00926 Colon cancer rs35309068 G T 0.04334 0.08648 0.02528 Rectal cancer rs35309068 G T 0.0123 0.70174 0.03211 |
| Gastroesophageal reflux | rs35316276 | C | T | -0.0094 | 0.37883 | 0.01067 | Esophageal carcinoma | rs35316276 | C | T | -0.06 | 0.38396 | 0.06897 | Acute gastritis | rs35316276 | C | T | -0.0055 | 0.87019 | 0.03368 | Chronic gastritis | rs35316276 | C | T | -0.0217 | 0.2032 | 0.01705 | Gastroduodenal ulcer | rs35316276 | C | T | 0.00898 | 0.60672 | 0.01745 | Gastric cancer | rs35316276 | C | T | -0.0211 | 0.6344 | 0.04446 | Fatty liver | rs35316276 | C | T | 0.02311 | 0.51393 | 0.03541 | Hepatic fibrosis | rs35316276 | C | T | 0.0458 | 0.7337 | 0.13463 | Cirrhosis | rs35316276 | C | T | 0.00747 | 0.77438 | 0.02607liver cancer rs35316276 C T 0.06831 0.2614 0.06082 Choleliths rs35316276 C T -0.005 0.57975 0.00903 Acute pancreatitis rs35316276 C T -0.0108 0.60609 0.02102 Chronic pancreatitis rs35316276 C T 0.00056 0.9842 0.02832 Pancreatic cancer rs35316276 C T -0.0428 0.33026 0.04399 Ulcerative colitis rs35316276 C T 0.04205 0.07012 0.02322 Crohn's disease rs35316276 C T -0.0818 0.04045 0.03994 Irrtable bowel syndrome rs35316276 C T -0.0028 0.87286 0.01742 Acute appendicitis rs35316276 C T -0.001 0.91914 0.01001 Colon cancer rs35316276 C T 0.0262 0.3386 0.02738 Rectal cancer rs35316276 C T 0.015 0.66568 0.03471 |
| Gastroesophageal reflux | rs35417702 | C | T | -0.0073 | 0.45393 | 0.0098 | Esophageal carcinoma | rs35417702 | C | T | -0.0428 | 0.50056 | 0.06356 | Acute gastritis | rs35417702 | C | T | 0.00207 | 0.94673 | 0.03095 | Chronic gastritis | rs35417702 | C | T | 0.00202 | 0.89755 | 0.01567 | Gastroduodenal ulcer | rs35417702 | C | T | -0.0141 | 0.37812 | 0.01604 | Gastric cancer | rs35417702 | C | T | -0.0058 | 0.88721 | 0.0408 | Fatty liver | rs35417702 | C | T | -0.0277 | 0.39547 | 0.03255 | Hepatic fibrosis | rs35417702 | C | T | 0.11159 | 0.36812 | 0.12399 | Cirrhosis | rs35417702 | C | T | -0.0179 | 0.45586 | 0.02398liver cancer rs35417702 C T 0.00214 0.96953 0.05608 Choleliths rs35417702 C T -0.0257 0.00194 0.00828 Acute pancreatitis rs35417702 C T -0.0227 0.24042 0.01935 Chronic pancreatitis rs35417702 C T -0.0037 0.88866 0.02609 Pancreatic cancer rs35417702 C T 0.02823 0.48496 0.04042 Ulcerative colitis rs35417702 C T -0.0259 0.22696 0.02141 Crohn's disease rs35417702 C T -0.02 0.58744 0.03692 Irrtable bowel syndrome rs35417702 C T 0.02141 0.18117 0.01601 Acute appendicitis rs35417702 C T 0.00214 0.81643 0.00921 Colon cancer rs35417702 C T -0.0549 0.02891 0.02515 Rectal cancer rs35417702 C T -0.0174 0.58506 0.03192 |
| Gastroesophageal reflux | rs35475880 | G | T | -0.0138 | 0.2044 | 0.01085 | Esophageal carcinoma | rs35475880 | G | T | -0.1076 | 0.12497 | 0.07012 | Acute gastritis | rs35475880 | G | T | 0.05917 | 0.084 | 0.03424 | Chronic gastritis | rs35475880 | G | T | 0.00104 | 0.95229 | 0.01733 | Gastroduodenal ulcer | rs35475880 | G | T | 0.05481 | 0.00212 | 0.01784 | Gastric cancer | rs35475880 | G | T | 0.04614 | 0.30829 | 0.04528 | Fatty liver | rs35475880 | G | T | -0.0027 | 0.94051 | 0.03597 | Hepatic fibrosis | rs35475880 | G | T | 0.0062 | 0.96373 | 0.13641 | Cirrhosis | rs35475880 | G | T | 0.00772 | 0.77028 | 0.02644liver cancer rs35475880 G T 0.01585 0.79748 0.06176 Choleliths rs35475880 G T -0.0174 0.0582 0.00917 Acute pancreatitis rs35475880 G T -0.0113 0.5968 0.02135 Chronic pancreatitis rs35475880 G T 0.0045 0.87567 0.02875 Pancreatic cancer rs35475880 G T 0.05546 0.21294 0.04452 Ulcerative colitis rs35475880 G T -0.0127 0.59208 0.02365 Crohn's disease rs35475880 G T -0.0108 0.79108 0.04082 Irrtable bowel syndrome rs35475880 G T -0.0013 0.94215 0.01773 Acute appendicitis rs35475880 G T 0.00069 0.94624 0.01017 Colon cancer rs35475880 G T 0.00072 0.97921 0.02777 Rectal cancer rs35475880 G T -0.0231 0.51249 0.03532 |
| Gastroesophageal reflux | rs35532491 | T | A | 0.02401 | 0.37181 | 0.02688 | Esophageal carcinoma | rs35532491 | T | A | -0.2629 | 0.12692 | 0.17223 | Acute gastritis | rs35532491 | T | A | 0.04056 | 0.6337 | 0.08513 | Chronic gastritis | rs35532491 | T | A | 0.02611 | 0.54272 | 0.04289 | Gastroduodenal ulcer | rs35532491 | T | A | -0.0103 | 0.81475 | 0.04417 | Gastric cancer | rs35532491 | T | A | -0.0762 | 0.48975 | 0.11039 | Fatty liver | rs35532491 | T | A | -0.1014 | 0.25794 | 0.08964 | Hepatic fibrosis | rs35532491 | T | A | -0.0644 | 0.85405 | 0.34986 | Cirrhosis | rs35532491 | T | A | 0.05797 | 0.3849 | 0.06672liver cancer rs35532491 T A 0.10849 0.47631 0.15232 Choleliths rs35532491 T A 0.00244 0.9148 0.02276 Acute pancreatitis rs35532491 T A 0.01333 0.80389 0.05367 Chronic pancreatitis rs35532491 T A -0.0155 0.83163 0.07278 Pancreatic cancer rs35532491 T A -0.1095 0.32215 0.11063 Ulcerative colitis rs35532491 T A 0.04644 0.43638 0.05967 Crohn's disease rs35532491 T A -0.2448 0.01742 0.10296 Irrtable bowel syndrome rs35532491 T A -0.0344 0.43772 0.04427 Acute appendicitis rs35532491 T A 0.02 0.43677 0.02572 Colon cancer rs35532491 T A 0.05679 0.40244 0.06782 Rectal cancer rs35532491 T A -0.0049 0.95493 0.08613 |
| Gastroesophageal reflux | rs36083520 | C | T | -0.0084 | 0.54731 | 0.01391 | Esophageal carcinoma | rs36083520 | C | T | -0.026 | 0.77248 | 0.08993 | Acute gastritis | rs36083520 | C | T | -0.0166 | 0.70662 | 0.04406 | Chronic gastritis | rs36083520 | C | T | -0.005 | 0.82348 | 0.0222 | Gastroduodenal ulcer | rs36083520 | C | T | -0.0128 | 0.57489 | 0.02276 | Gastric cancer | rs36083520 | C | T | -0.001 | 0.98665 | 0.0575 | Fatty liver | rs36083520 | C | T | -0.0497 | 0.28169 | 0.04612 | Hepatic fibrosis | rs36083520 | C | T | 0.33898 | 0.05615 | 0.17749 | Cirrhosis | rs36083520 | C | T | 0.01485 | 0.66342 | 0.03411liver cancer rs36083520 C T 0.02542 0.74761 0.079 Choleliths rs36083520 C T 0.01691 0.1511 0.01178 Acute pancreatitis rs36083520 C T 0.05294 0.0538 0.02745 Chronic pancreatitis rs36083520 C T -0.0343 0.35478 0.0371 Pancreatic cancer rs36083520 C T -0.0978 0.08777 0.05726 Ulcerative colitis rs36083520 C T 0.02253 0.45807 0.03036 Crohn's disease rs36083520 C T -0.0378 0.46904 0.05217 Irrtable bowel syndrome rs36083520 C T -0.0191 0.40146 0.02272 Acute appendicitis rs36083520 C T 0.00037 0.97752 0.01307 Colon cancer rs36083520 C T -0.0177 0.61941 0.03566 Rectal cancer rs36083520 C T 0.02524 0.57514 0.04504 |
| Gastroesophageal reflux | rs36119825 | G | A | 0.00728 | 0.45797 | 0.00981 | Esophageal carcinoma | rs36119825 | G | A | -0.0332 | 0.60083 | 0.06353 | Acute gastritis | rs36119825 | G | A | 0.07033 | 0.02334 | 0.03101 | Chronic gastritis | rs36119825 | G | A | 0.04371 | 0.00529 | 0.01568 | Gastroduodenal ulcer | rs36119825 | G | A | 0.02482 | 0.12194 | 0.01604 | Gastric cancer | rs36119825 | G | A | -0.0865 | 0.03398 | 0.0408 | Fatty liver | rs36119825 | G | A | 0.01606 | 0.62177 | 0.03256 | Hepatic fibrosis | rs36119825 | G | A | 0.16129 | 0.19446 | 0.12431 | Cirrhosis | rs36119825 | G | A | 0.01628 | 0.4969 | 0.02397liver cancer rs36119825 G A -0.029 0.60345 0.05588 Choleliths rs36119825 G A 0.00769 0.35377 0.00829 Acute pancreatitis rs36119825 G A 0.00904 0.64019 0.01933 Chronic pancreatitis rs36119825 G A 0.02122 0.41585 0.02608 Pancreatic cancer rs36119825 G A -0.0129 0.74807 0.04028 Ulcerative colitis rs36119825 G A 0.02185 0.30789 0.02143 Crohn's disease rs36119825 G A -0.0016 0.96618 0.03698 Irrtable bowel syndrome rs36119825 G A 0.00726 0.65086 0.01604 Acute appendicitis rs36119825 G A 0.00422 0.6471 0.00921 Colon cancer rs36119825 G A 0.02376 0.34523 0.02518 Rectal cancer rs36119825 G A -0.0331 0.29965 0.03195 |
| Gastroesophageal reflux | rs363096 | C | T | -0.0208 | 0.03944 | 0.01008 | Esophageal carcinoma | rs363096 | C | T | 0.05299 | 0.4163 | 0.06519 | Acute gastritis | rs363096 | C | T | -0.0506 | 0.11264 | 0.03186 | Chronic gastritis | rs363096 | C | T | 0.00485 | 0.76354 | 0.01613 | Gastroduodenal ulcer | rs363096 | C | T | -0.0311 | 0.05882 | 0.01647 | Gastric cancer | rs363096 | C | T | 0.02452 | 0.55769 | 0.04182 | Fatty liver | rs363096 | C | T | -0.0266 | 0.42709 | 0.03345 | Hepatic fibrosis | rs363096 | C | T | 0.11236 | 0.38056 | 0.12814 | Cirrhosis | rs363096 | C | T | -0.0088 | 0.72002 | 0.02465liver cancer rs363096 C T -0.0435 0.44798 0.05736 Choleliths rs363096 C T -0.0212 0.01303 0.00852 Acute pancreatitis rs363096 C T -0.0108 0.58769 0.01988 Chronic pancreatitis rs363096 C T 0.0009 0.97326 0.02679 Pancreatic cancer rs363096 C T -0.0441 0.28824 0.04149 Ulcerative colitis rs363096 C T 0.0028 0.89892 0.02201 Crohn's disease rs363096 C T -0.0379 0.31847 0.03796 Irrtable bowel syndrome rs363096 C T -0.0327 0.04701 0.01647 Acute appendicitis rs363096 C T -0.0013 0.89487 0.00947 Colon cancer rs363096 C T 0.00735 0.77608 0.02583 Rectal cancer rs363096 C T 0.02535 0.43882 0.03274 |
| Gastroesophageal reflux | rs3747631 | G | C | -0.0094 | 0.42199 | 0.01172 | Esophageal carcinoma | rs3747631 | G | C | 0.17787 | 0.01916 | 0.07594 | Acute gastritis | rs3747631 | G | C | -0.0202 | 0.58564 | 0.03705 | Chronic gastritis | rs3747631 | G | C | -0.0235 | 0.20925 | 0.01875 | Gastroduodenal ulcer | rs3747631 | G | C | -0.0053 | 0.78248 | 0.01921 | Gastric cancer | rs3747631 | G | C | 0.08971 | 0.06651 | 0.04889 | Fatty liver | rs3747631 | G | C | -0.0095 | 0.80612 | 0.03887 | Hepatic fibrosis | rs3747631 | G | C | 0.37543 | 0.01067 | 0.14704 | Cirrhosis | rs3747631 | G | C | 0.06425 | 0.02516 | 0.02869liver cancer rs3747631 G C 0.05186 0.43962 0.0671 Choleliths rs3747631 G C -0.0017 0.86221 0.00992 Acute pancreatitis rs3747631 G C 0.05116 0.02699 0.02313 Chronic pancreatitis rs3747631 G C -0.0274 0.38004 0.03118 Pancreatic cancer rs3747631 G C 0.05264 0.27563 0.04829 Ulcerative colitis rs3747631 G C -0.009 0.72469 0.02553 Crohn's disease rs3747631 G C -0.0258 0.55772 0.04403 Irrtable bowel syndrome rs3747631 G C -0.0434 0.02316 0.01913 Acute appendicitis rs3747631 G C -0.0046 0.67903 0.011 Colon cancer rs3747631 G C 0.05224 0.08395 0.03023 Rectal cancer rs3747631 G C 0.02987 0.43571 0.03832 |
| Gastroesophageal reflux | rs3788556 | T | C | 0.0072 | 0.47416 | 0.01006 | Esophageal carcinoma | rs3788556 | T | C | -0.0024 | 0.97054 | 0.06513 | Acute gastritis | rs3788556 | T | C | 0.05849 | 0.06528 | 0.03173 | Chronic gastritis | rs3788556 | T | C | 0.00194 | 0.90402 | 0.01608 | Gastroduodenal ulcer | rs3788556 | T | C | 0.00146 | 0.92917 | 0.01646 | Gastric cancer | rs3788556 | T | C | 0.01411 | 0.73612 | 0.04188 | Fatty liver | rs3788556 | T | C | 0.00118 | 0.97175 | 0.03344 | Hepatic fibrosis | rs3788556 | T | C | -0.1099 | 0.38881 | 0.12754 | Cirrhosis | rs3788556 | T | C | 0.02454 | 0.31945 | 0.02465liver cancer rs3788556 T C 0.04924 0.39149 0.05746 Choleliths rs3788556 T C -0.0077 0.36503 0.00851 Acute pancreatitis rs3788556 T C -0.0256 0.19708 0.01986 Chronic pancreatitis rs3788556 T C -0.0342 0.20228 0.02679 Pancreatic cancer rs3788556 T C 0.03801 0.35957 0.04148 Ulcerative colitis rs3788556 T C 0.01651 0.45276 0.02198 Crohn's disease rs3788556 T C -0.036 0.34239 0.03791 Irrtable bowel syndrome rs3788556 T C -0.0242 0.14122 0.01644 Acute appendicitis rs3788556 T C -0.0067 0.47607 0.00946 Colon cancer rs3788556 T C 0.01902 0.46198 0.02586 Rectal cancer rs3788556 T C 0.02469 0.45122 0.03277 |
| Gastroesophageal reflux | rs3800546 | G | C | 0.01589 | 0.13943 | 0.01075 | Esophageal carcinoma | rs3800546 | G | C | -0.0276 | 0.69157 | 0.06952 | Acute gastritis | rs3800546 | G | C | 0.05588 | 0.10031 | 0.034 | Chronic gastritis | rs3800546 | G | C | 0.01605 | 0.35079 | 0.0172 | Gastroduodenal ulcer | rs3800546 | G | C | -0.0024 | 0.89256 | 0.01762 | Gastric cancer | rs3800546 | G | C | 0.01657 | 0.7101 | 0.04458 | Fatty liver | rs3800546 | G | C | -0.0541 | 0.12973 | 0.03573 | Hepatic fibrosis | rs3800546 | G | C | -0.0277 | 0.83955 | 0.13691 | Cirrhosis | rs3800546 | G | C | -0.0024 | 0.92627 | 0.02633liver cancer rs3800546 G C 0.00575 0.92548 0.06144 Choleliths rs3800546 G C 0.00682 0.45326 0.0091 Acute pancreatitis rs3800546 G C 0.02185 0.30346 0.02123 Chronic pancreatitis rs3800546 G C 0.00613 0.83052 0.02865 Pancreatic cancer rs3800546 G C 0.00858 0.84611 0.04422 Ulcerative colitis rs3800546 G C -0.0196 0.40396 0.02347 Crohn's disease rs3800546 G C 0.03305 0.4137 0.04043 Irrtable bowel syndrome rs3800546 G C -0.0195 0.26631 0.01759 Acute appendicitis rs3800546 G C 0.02042 0.04315 0.0101 Colon cancer rs3800546 G C -0.0258 0.3476 0.02748 Rectal cancer rs3800546 G C -0.0234 0.50335 0.03492 |
| Gastroesophageal reflux | rs3809634 | A | G | 0.01834 | 0.09836 | 0.01109 | Esophageal carcinoma | rs3809634 | A | G | 0.01001 | 0.8894 | 0.07199 | Acute gastritis | rs3809634 | A | G | 0.02684 | 0.44354 | 0.03503 | Chronic gastritis | rs3809634 | A | G | 0.01094 | 0.53637 | 0.01769 | Gastroduodenal ulcer | rs3809634 | A | G | -0.0078 | 0.66721 | 0.01809 | Gastric cancer | rs3809634 | A | G | 0.0326 | 0.48011 | 0.04617 | Fatty liver | rs3809634 | A | G | 0.01331 | 0.71805 | 0.03686 | Hepatic fibrosis | rs3809634 | A | G | -0.1763 | 0.21115 | 0.14101 | Cirrhosis | rs3809634 | A | G | -0.0108 | 0.6914 | 0.02712liver cancer rs3809634 A G 0.01201 0.84941 0.06326 Choleliths rs3809634 A G 0.00843 0.36827 0.00937 Acute pancreatitis rs3809634 A G 0.05494 0.01193 0.02185 Chronic pancreatitis rs3809634 A G 0.04951 0.09352 0.02952 Pancreatic cancer rs3809634 A G -0.0116 0.79989 0.04558 Ulcerative colitis rs3809634 A G -0.0271 0.26252 0.02422 Crohn's disease rs3809634 A G -0.045 0.28241 0.04184 Irrtable bowel syndrome rs3809634 A G 0.04014 0.02681 0.01813 Acute appendicitis rs3809634 A G -0.0022 0.83013 0.01043 Colon cancer rs3809634 A G -0.0359 0.20587 0.02837 Rectal cancer rs3809634 A G -0.0011 0.97657 0.03606 |
| Gastroesophageal reflux | rs3890802 | A | G | 0.00247 | 0.83263 | 0.01167 | Esophageal carcinoma | rs3890802 | A | G | -0.1598 | 0.03413 | 0.07544 | Acute gastritis | rs3890802 | A | G | -0.0157 | 0.6702 | 0.03686 | Chronic gastritis | rs3890802 | A | G | -0.0183 | 0.32548 | 0.01864 | Gastroduodenal ulcer | rs3890802 | A | G | -0.0282 | 0.13987 | 0.01909 | Gastric cancer | rs3890802 | A | G | -0.0109 | 0.82087 | 0.04833 | Fatty liver | rs3890802 | A | G | 0.01406 | 0.71713 | 0.0388 | Hepatic fibrosis | rs3890802 | A | G | -0.0611 | 0.68318 | 0.14961 | Cirrhosis | rs3890802 | A | G | -0.0364 | 0.20357 | 0.02866liver cancer rs3890802 A G -0.066 0.32222 0.06669 Choleliths rs3890802 A G -0.0241 0.01445 0.00986 Acute pancreatitis rs3890802 A G -0.0036 0.87707 0.02307 Chronic pancreatitis rs3890802 A G 0.02913 0.34962 0.03115 Pancreatic cancer rs3890802 A G -0.0145 0.7627 0.04808 Ulcerative colitis rs3890802 A G -0.0039 0.88018 0.02557 Crohn's disease rs3890802 A G 0.00079 0.98576 0.04404 Irrtable bowel syndrome rs3890802 A G -0.0217 0.25514 0.01909 Acute appendicitis rs3890802 A G -0.0001 0.99252 0.011 Colon cancer rs3890802 A G 0.01959 0.51188 0.02987 Rectal cancer rs3890802 A G 0.01259 0.7399 0.03791 |
| Gastroesophageal reflux | rs3897821 | A | G | -0.0127 | 0.23394 | 0.01066 | Esophageal carcinoma | rs3897821 | A | G | -0.0936 | 0.17668 | 0.06931 | Acute gastritis | rs3897821 | A | G | -0.0643 | 0.05664 | 0.03372 | Chronic gastritis | rs3897821 | A | G | -0.0048 | 0.77702 | 0.01703 | Gastroduodenal ulcer | rs3897821 | A | G | -0.0378 | 0.03014 | 0.01745 | Gastric cancer | rs3897821 | A | G | 0.00555 | 0.90078 | 0.04451 | Fatty liver | rs3897821 | A | G | -0.0336 | 0.34299 | 0.03539 | Hepatic fibrosis | rs3897821 | A | G | 0.06136 | 0.65109 | 0.13569 | Cirrhosis | rs3897821 | A | G | 0.00378 | 0.88511 | 0.02613liver cancer rs3897821 A G -0.0527 0.38901 0.06119 Choleliths rs3897821 A G -0.0047 0.60489 0.00901 Acute pancreatitis rs3897821 A G -0.0395 0.06088 0.02106 Chronic pancreatitis rs3897821 A G -0.0004 0.98832 0.0284 Pancreatic cancer rs3897821 A G -0.0446 0.31228 0.04414 Ulcerative colitis rs3897821 A G 0.01015 0.66297 0.02329 Crohn's disease rs3897821 A G -0.0485 0.22677 0.04009 Irrtable bowel syndrome rs3897821 A G -0.0274 0.11553 0.01739 Acute appendicitis rs3897821 A G 0.00849 0.3973 0.01003 Colon cancer rs3897821 A G -0.0113 0.67923 0.02741 Rectal cancer rs3897821 A G -0.0499 0.15055 0.03472 |
| Gastroesophageal reflux | rs401687 | G | C | 0.0101 | 0.3079 | 0.0099 | Esophageal carcinoma | rs401687 | G | C | 0.11082 | 0.0842 | 0.06417 | Acute gastritis | rs401687 | G | C | 0.0116 | 0.7109 | 0.0313 | Chronic gastritis | rs401687 | G | C | -0.0028 | 0.85846 | 0.01585 | Gastroduodenal ulcer | rs401687 | G | C | -0.0164 | 0.31267 | 0.01621 | Gastric cancer | rs401687 | G | C | -0.042 | 0.30897 | 0.04127 | Fatty liver | rs401687 | G | C | -0.0052 | 0.87504 | 0.03287 | Hepatic fibrosis | rs401687 | G | C | 0.09898 | 0.43072 | 0.12562 | Cirrhosis | rs401687 | G | C | 0.02555 | 0.29187 | 0.02424liver cancer rs401687 G C 0.0131 0.81694 0.05659 Choleliths rs401687 G C -3E-06 0.99967 0.00837 Acute pancreatitis rs401687 G C 0.00809 0.67886 0.01954 Chronic pancreatitis rs401687 G C 0.02104 0.42461 0.02635 Pancreatic cancer rs401687 G C 0.00867 0.83151 0.04077 Ulcerative colitis rs401687 G C 0.0156 0.47051 0.02162 Crohn's disease rs401687 G C 0.0161 0.66563 0.03726 Irrtable bowel syndrome rs401687 G C -0.0058 0.71987 0.01618 Acute appendicitis rs401687 G C -0.0089 0.33792 0.0093 Colon cancer rs401687 G C -0.0122 0.6323 0.02543 Rectal cancer rs401687 G C 0.02209 0.49422 0.03231 |
| Gastroesophageal reflux | rs406413 | A | T | -0.0095 | 0.43376 | 0.0121 | Esophageal carcinoma | rs406413 | A | T | -0.023 | 0.7676 | 0.07798 | Acute gastritis | rs406413 | A | T | -0.0325 | 0.39367 | 0.03813 | Chronic gastritis | rs406413 | A | T | 0.01034 | 0.59314 | 0.01935 | Gastroduodenal ulcer | rs406413 | A | T | 0.01118 | 0.57116 | 0.01974 | Gastric cancer | rs406413 | A | T | 0.00311 | 0.9507 | 0.05032 | Fatty liver | rs406413 | A | T | 0.03814 | 0.34234 | 0.04017 | Hepatic fibrosis | rs406413 | A | T | -0.192 | 0.20969 | 0.15304 | Cirrhosis | rs406413 | A | T | 0.06936 | 0.01872 | 0.0295liver cancer rs406413 A T 0.11267 0.10256 0.06901 Choleliths rs406413 A T -0.0048 0.6407 0.01021 Acute pancreatitis rs406413 A T 0.01102 0.64371 0.02383 Chronic pancreatitis rs406413 A T 0.00317 0.92136 0.03211 Pancreatic cancer rs406413 A T -0.0503 0.31073 0.04959 Ulcerative colitis rs406413 A T -0.0205 0.43819 0.02638 Crohn's disease rs406413 A T 0.01826 0.68818 0.04551 Irrtable bowel syndrome rs406413 A T -0.018 0.36173 0.01976 Acute appendicitis rs406413 A T -0.0038 0.73863 0.01134 Colon cancer rs406413 A T -0.0536 0.0846 0.0311 Rectal cancer rs406413 A T 0.02995 0.44839 0.0395 |
| Gastroesophageal reflux | rs4073894 | A | G | -0.0134 | 0.2669 | 0.01211 | Esophageal carcinoma | rs4073894 | A | G | 0.0723 | 0.35664 | 0.07844 | Acute gastritis | rs4073894 | A | G | -0.0503 | 0.18997 | 0.03836 | Chronic gastritis | rs4073894 | A | G | 0.02416 | 0.21185 | 0.01935 | Gastroduodenal ulcer | rs4073894 | A | G | 0.0222 | 0.26274 | 0.01982 | Gastric cancer | rs4073894 | A | G | 0.03002 | 0.54899 | 0.05009 | Fatty liver | rs4073894 | A | G | 0.02319 | 0.56468 | 0.04027 | Hepatic fibrosis | rs4073894 | A | G | -0.0218 | 0.88781 | 0.15439 | Cirrhosis | rs4073894 | A | G | -0.0622 | 0.03649 | 0.02972liver cancer rs4073894 A G 0.04284 0.53492 0.06903 Choleliths rs4073894 A G 0.01463 0.15353 0.01025 Acute pancreatitis rs4073894 A G 0.00827 0.72967 0.02394 Chronic pancreatitis rs4073894 A G -0.007 0.82938 0.03227 Pancreatic cancer rs4073894 A G -0.018 0.71955 0.05002 Ulcerative colitis rs4073894 A G -0.0129 0.62674 0.02647 Crohn's disease rs4073894 A G 0.01255 0.7837 0.04572 Irrtable bowel syndrome rs4073894 A G -0.0279 0.15884 0.0198 Acute appendicitis rs4073894 A G 0.00083 0.94173 0.0114 Colon cancer rs4073894 A G -0.0119 0.70111 0.03107 Rectal cancer rs4073894 A G -0.0042 0.91533 0.03944 |
| Gastroesophageal reflux | rs4328757 | T | C | -0.0001 | 0.98852 | 0.00986 | Esophageal carcinoma | rs4328757 | T | C | 0.06658 | 0.29781 | 0.06395 | Acute gastritis | rs4328757 | T | C | 0.02839 | 0.36175 | 0.03112 | Chronic gastritis | rs4328757 | T | C | -0.0148 | 0.34623 | 0.01576 | Gastroduodenal ulcer | rs4328757 | T | C | -0.0377 | 0.01924 | 0.01612 | Gastric cancer | rs4328757 | T | C | 0.00253 | 0.95081 | 0.04102 | Fatty liver | rs4328757 | T | C | -0.0228 | 0.4862 | 0.0327 | Hepatic fibrosis | rs4328757 | T | C | -0.1067 | 0.39154 | 0.12456 | Cirrhosis | rs4328757 | T | C | 0.00747 | 0.75662 | 0.02409liver cancer rs4328757 T C -0.0696 0.21619 0.05629 Choleliths rs4328757 T C 0.00734 0.37801 0.00833 Acute pancreatitis rs4328757 T C -0.0074 0.70201 0.01942 Chronic pancreatitis rs4328757 T C -0.0235 0.3688 0.02618 Pancreatic cancer rs4328757 T C -0.0107 0.79273 0.04064 Ulcerative colitis rs4328757 T C 0.04429 0.03943 0.0215 Crohn's disease rs4328757 T C 0.04693 0.2052 0.03704 Irrtable bowel syndrome rs4328757 T C 0.0007 0.96515 0.01611 Acute appendicitis rs4328757 T C 0.0051 0.58108 0.00925 Colon cancer rs4328757 T C -0.064 0.01144 0.02531 Rectal cancer rs4328757 T C -0.0239 0.45726 0.03219 |
| Gastroesophageal reflux | rs4352658 | T | C | 0.01791 | 0.26281 | 0.01599 | Esophageal carcinoma | rs4352658 | T | C | -0.0523 | 0.61566 | 0.10422 | Acute gastritis | rs4352658 | T | C | -0.0079 | 0.87539 | 0.05048 | Chronic gastritis | rs4352658 | T | C | 0.01248 | 0.62639 | 0.02564 | Gastroduodenal ulcer | rs4352658 | T | C | 0.01693 | 0.518 | 0.02619 | Gastric cancer | rs4352658 | T | C | 0.06886 | 0.2957 | 0.06585 | Fatty liver | rs4352658 | T | C | 0.03576 | 0.50146 | 0.0532 | Hepatic fibrosis | rs4352658 | T | C | 0.02899 | 0.8882 | 0.20622 | Cirrhosis | rs4352658 | T | C | 0.05477 | 0.1656 | 0.03951liver cancer rs4352658 T C -0.1593 0.08456 0.09234 Choleliths rs4352658 T C 0.00928 0.49134 0.01349 Acute pancreatitis rs4352658 T C 0.01687 0.59307 0.03157 Chronic pancreatitis rs4352658 T C 0.04231 0.32453 0.04294 Pancreatic cancer rs4352658 T C -0.0154 0.81633 0.0661 Ulcerative colitis rs4352658 T C 0.12953 0.00012 0.03371 Crohn's disease rs4352658 T C 0.05797 0.33695 0.06038 Irrtable bowel syndrome rs4352658 T C 0.02927 0.26409 0.02621 Acute appendicitis rs4352658 T C 0.01184 0.42998 0.01501 Colon cancer rs4352658 T C 0.00865 0.83214 0.04081 Rectal cancer rs4352658 T C -0.0075 0.8852 0.05187 |
| Gastroesophageal reflux | rs4369924 | A | G | 0.00291 | 0.84844 | 0.01522 | Esophageal carcinoma | rs4369924 | A | G | 0.02623 | 0.78946 | 0.09824 | Acute gastritis | rs4369924 | A | G | -0.0208 | 0.66483 | 0.04805 | Chronic gastritis | rs4369924 | A | G | 0.03777 | 0.12034 | 0.02431 | Gastroduodenal ulcer | rs4369924 | A | G | -0.006 | 0.80973 | 0.02493 | Gastric cancer | rs4369924 | A | G | 0.08838 | 0.16316 | 0.06338 | Fatty liver | rs4369924 | A | G | 0.04814 | 0.34046 | 0.05051 | Hepatic fibrosis | rs4369924 | A | G | 0.05709 | 0.76755 | 0.19313 | Cirrhosis | rs4369924 | A | G | 0.01734 | 0.64201 | 0.0373liver cancer rs4369924 A G -0.0748 0.3903 0.08705 Choleliths rs4369924 A G 0.00778 0.54564 0.01287 Acute pancreatitis rs4369924 A G 0.01196 0.69079 0.03006 Chronic pancreatitis rs4369924 A G -0.0236 0.55987 0.04048 Pancreatic cancer rs4369924 A G 0.05515 0.38141 0.06301 Ulcerative colitis rs4369924 A G 0.02217 0.50443 0.03321 Crohn's disease rs4369924 A G -0.0809 0.1583 0.05732 Irrtable bowel syndrome rs4369924 A G -0.0091 0.71435 0.02488 Acute appendicitis rs4369924 A G -0.0115 0.42213 0.01429 Colon cancer rs4369924 A G -0.0088 0.82156 0.03919 Rectal cancer rs4369924 A G -0.103 0.03829 0.04972 |
| Gastroesophageal reflux | rs4382592 | T | G | 0.01756 | 0.10971 | 0.01098 | Esophageal carcinoma | rs4382592 | T | G | -0.0476 | 0.50398 | 0.07125 | Acute gastritis | rs4382592 | T | G | 0.03028 | 0.3827 | 0.03468 | Chronic gastritis | rs4382592 | T | G | 0.01266 | 0.47146 | 0.01758 | Gastroduodenal ulcer | rs4382592 | T | G | 0.03025 | 0.09281 | 0.018 | Gastric cancer | rs4382592 | T | G | -0.0002 | 0.99695 | 0.04582 | Fatty liver | rs4382592 | T | G | 0.02744 | 0.45158 | 0.03645 | Hepatic fibrosis | rs4382592 | T | G | -0.0473 | 0.73385 | 0.13903 | Cirrhosis | rs4382592 | T | G | 0.0221 | 0.41157 | 0.02692liver cancer rs4382592 T G 0.03553 0.57162 0.0628 Choleliths rs4382592 T G 0.01003 0.28102 0.0093 Acute pancreatitis rs4382592 T G 0.00211 0.92255 0.02172 Chronic pancreatitis rs4382592 T G -1E-05 0.99964 0.02924 Pancreatic cancer rs4382592 T G -0.0511 0.25915 0.04529 Ulcerative colitis rs4382592 T G -0.0075 0.75406 0.02394 Crohn's disease rs4382592 T G -0.0049 0.90551 0.04129 Irrtable bowel syndrome rs4382592 T G -0.0091 0.61155 0.01793 Acute appendicitis rs4382592 T G -0.004 0.69805 0.01031 Colon cancer rs4382592 T G -0.0295 0.29853 0.02839 Rectal cancer rs4382592 T G 0.00581 0.87165 0.03594 |
| Gastroesophageal reflux | rs4384309 | G | A | 0.00511 | 0.60754 | 0.00995 | Esophageal carcinoma | rs4384309 | G | A | -0.0301 | 0.64036 | 0.06446 | Acute gastritis | rs4384309 | G | A | -0.006 | 0.84811 | 0.0315 | Chronic gastritis | rs4384309 | G | A | -0.0052 | 0.7448 | 0.01593 | Gastroduodenal ulcer | rs4384309 | G | A | -0.0098 | 0.54628 | 0.01631 | Gastric cancer | rs4384309 | G | A | 0.02582 | 0.53303 | 0.04141 | Fatty liver | rs4384309 | G | A | -0.0771 | 0.01968 | 0.03306 | Hepatic fibrosis | rs4384309 | G | A | -0.0108 | 0.93167 | 0.12596 | Cirrhosis | rs4384309 | G | A | -0.0298 | 0.22227 | 0.02438liver cancer rs4384309 G A -0.0254 0.65546 0.05683 Choleliths rs4384309 G A 0.01716 0.04152 0.00842 Acute pancreatitis rs4384309 G A 0.03105 0.11377 0.01963 Chronic pancreatitis rs4384309 G A 0.02818 0.28715 0.02648 Pancreatic cancer rs4384309 G A -0.0229 0.57583 0.04097 Ulcerative colitis rs4384309 G A -0.0067 0.75881 0.02173 Crohn's disease rs4384309 G A -0.0116 0.75778 0.03747 Irrtable bowel syndrome rs4384309 G A 0.03372 0.03809 0.01626 Acute appendicitis rs4384309 G A 0.0002 0.98299 0.00936 Colon cancer rs4384309 G A -0.0251 0.32521 0.02554 Rectal cancer rs4384309 G A -0.0287 0.37551 0.03243 |
| Gastroesophageal reflux | rs4442732 | G | A | 0.00238 | 0.82626 | 0.01084 | Esophageal carcinoma | rs4442732 | G | A | 0.09453 | 0.17848 | 0.07026 | Acute gastritis | rs4442732 | G | A | -0.0811 | 0.01763 | 0.03417 | Chronic gastritis | rs4442732 | G | A | -0.0289 | 0.09461 | 0.01732 | Gastroduodenal ulcer | rs4442732 | G | A | -0.0217 | 0.22052 | 0.01773 | Gastric cancer | rs4442732 | G | A | 0.00475 | 0.91595 | 0.04503 | Fatty liver | rs4442732 | G | A | 0.02807 | 0.43615 | 0.03605 | Hepatic fibrosis | rs4442732 | G | A | -0.0883 | 0.52376 | 0.13846 | Cirrhosis | rs4442732 | G | A | 0.00112 | 0.96638 | 0.02666liver cancer rs4442732 G A -0.0159 0.7977 0.06199 Choleliths rs4442732 G A 0.0107 0.24241 0.00916 Acute pancreatitis rs4442732 G A -0.0218 0.30893 0.02142 Chronic pancreatitis rs4442732 G A 5.6E-05 0.99846 0.02894 Pancreatic cancer rs4442732 G A -0.0373 0.40513 0.04477 Ulcerative colitis rs4442732 G A 0.03613 0.12798 0.02374 Crohn's disease rs4442732 G A -0.0797 0.05146 0.04094 Irrtable bowel syndrome rs4442732 G A -0.003 0.86468 0.01772 Acute appendicitis rs4442732 G A 0.00237 0.81659 0.01021 Colon cancer rs4442732 G A 0.02441 0.37992 0.0278 Rectal cancer rs4442732 G A -0.0088 0.80325 0.03526 |
| Gastroesophageal reflux | rs466047 | A | T | -0.0082 | 0.41047 | 0.00991 | Esophageal carcinoma | rs466047 | A | T | 0.00596 | 0.92607 | 0.06422 | Acute gastritis | rs466047 | A | T | -0.0064 | 0.83718 | 0.03132 | Chronic gastritis | rs466047 | A | T | 0.02185 | 0.16795 | 0.01585 | Gastroduodenal ulcer | rs466047 | A | T | 0.02317 | 0.15288 | 0.01621 | Gastric cancer | rs466047 | A | T | -0.0574 | 0.16451 | 0.04129 | Fatty liver | rs466047 | A | T | -0.0069 | 0.83419 | 0.03291 | Hepatic fibrosis | rs466047 | A | T | -0.0317 | 0.80081 | 0.12565 | Cirrhosis | rs466047 | A | T | 0.04545 | 0.06088 | 0.02425liver cancer rs466047 A T 0.07336 0.19575 0.0567 Choleliths rs466047 A T 0.01834 0.02866 0.00838 Acute pancreatitis rs466047 A T 0.02411 0.21751 0.01955 Chronic pancreatitis rs466047 A T 0.01855 0.48166 0.02636 Pancreatic cancer rs466047 A T 0.01899 0.64238 0.0409 Ulcerative colitis rs466047 A T -0.0278 0.1992 0.02163 Crohn's disease rs466047 A T 0.03607 0.3335 0.03729 Irrtable bowel syndrome rs466047 A T -0.0079 0.62706 0.0162 Acute appendicitis rs466047 A T 0.00642 0.4903 0.0093 Colon cancer rs466047 A T 0.00439 0.86324 0.02547 Rectal cancer rs466047 A T 0.06224 0.05426 0.03234 |
| Gastroesophageal reflux | rs4667029 | G | C | 0.00018 | 0.98635 | 0.0104 | Esophageal carcinoma | rs4667029 | G | C | -0.0556 | 0.40909 | 0.06734 | Acute gastritis | rs4667029 | G | C | -0.045 | 0.17062 | 0.03281 | Chronic gastritis | rs4667029 | G | C | -0.019 | 0.25179 | 0.01661 | Gastroduodenal ulcer | rs4667029 | G | C | -0.0039 | 0.82049 | 0.017 | Gastric cancer | rs4667029 | G | C | 0.02173 | 0.61482 | 0.04319 | Fatty liver | rs4667029 | G | C | 0.06291 | 0.06842 | 0.03452 | Hepatic fibrosis | rs4667029 | G | C | -0.0248 | 0.85054 | 0.13182 | Cirrhosis | rs4667029 | G | C | 0.01585 | 0.53351 | 0.02546liver cancer rs4667029 G C -0.0345 0.5616 0.05936 Choleliths rs4667029 G C 0.00322 0.71384 0.00877 Acute pancreatitis rs4667029 G C -0.0415 0.04307 0.0205 Chronic pancreatitis rs4667029 G C -0.0311 0.26046 0.02768 Pancreatic cancer rs4667029 G C -0.0749 0.0806 0.04287 Ulcerative colitis rs4667029 G C -0.0148 0.51395 0.02273 Crohn's disease rs4667029 G C 0.01289 0.74207 0.03917 Irrtable bowel syndrome rs4667029 G C 0.00912 0.59158 0.017 Acute appendicitis rs4667029 G C -0.0041 0.67604 0.00977 Colon cancer rs4667029 G C -0.0436 0.10225 0.02667 Rectal cancer rs4667029 G C 0.03416 0.3124 0.03382 |
| Gastroesophageal reflux | rs4700393 | G | A | -0.011 | 0.26187 | 0.00981 | Esophageal carcinoma | rs4700393 | G | A | 0.06881 | 0.27896 | 0.06356 | Acute gastritis | rs4700393 | G | A | 0.05441 | 0.07926 | 0.031 | Chronic gastritis | rs4700393 | G | A | -0.017 | 0.27778 | 0.01568 | Gastroduodenal ulcer | rs4700393 | G | A | -0.0251 | 0.11773 | 0.01606 | Gastric cancer | rs4700393 | G | A | 0.0409 | 0.31621 | 0.04081 | Fatty liver | rs4700393 | G | A | -0.0026 | 0.93716 | 0.03254 | Hepatic fibrosis | rs4700393 | G | A | 0.05217 | 0.67464 | 0.12427 | Cirrhosis | rs4700393 | G | A | -0.0344 | 0.15167 | 0.024liver cancer rs4700393 G A -0.077 0.16913 0.05599 Choleliths rs4700393 G A -0.002 0.80847 0.0083 Acute pancreatitis rs4700393 G A 0.01365 0.48037 0.01934 Chronic pancreatitis rs4700393 G A 0.01328 0.61068 0.02609 Pancreatic cancer rs4700393 G A 0.00824 0.83873 0.04049 Ulcerative colitis rs4700393 G A 0.03083 0.14989 0.02141 Crohn's disease rs4700393 G A -0.0012 0.97347 0.03684 Irrtable bowel syndrome rs4700393 G A 0.01821 0.25571 0.01602 Acute appendicitis rs4700393 G A 0.00892 0.33261 0.00921 Colon cancer rs4700393 G A 0.02446 0.33121 0.02517 Rectal cancer rs4700393 G A 0.00193 0.95174 0.03192 |
| Gastroesophageal reflux | rs4726070 | G | A | -0.0025 | 0.80228 | 0.00983 | Esophageal carcinoma | rs4726070 | G | A | 0.0727 | 0.25331 | 0.06364 | Acute gastritis | rs4726070 | G | A | -0.018 | 0.5622 | 0.03105 | Chronic gastritis | rs4726070 | G | A | 0.00221 | 0.88789 | 0.01571 | Gastroduodenal ulcer | rs4726070 | G | A | -0.0026 | 0.87354 | 0.01605 | Gastric cancer | rs4726070 | G | A | -0.032 | 0.43366 | 0.04091 | Fatty liver | rs4726070 | G | A | 0.02375 | 0.46638 | 0.03261 | Hepatic fibrosis | rs4726070 | G | A | -0.0199 | 0.87342 | 0.12478 | Cirrhosis | rs4726070 | G | A | 0.0161 | 0.50252 | 0.02402liver cancer rs4726070 G A 0.02478 0.65861 0.05607 Choleliths rs4726070 G A -0.0195 0.01889 0.0083 Acute pancreatitis rs4726070 G A 0.01214 0.53069 0.01936 Chronic pancreatitis rs4726070 G A 0.00821 0.75348 0.02613 Pancreatic cancer rs4726070 G A 0.04625 0.25283 0.04045 Ulcerative colitis rs4726070 G A 0.01315 0.53992 0.02146 Crohn's disease rs4726070 G A 0.0016 0.96557 0.03699 Irrtable bowel syndrome rs4726070 G A -0.0027 0.86572 0.01605 Acute appendicitis rs4726070 G A 0.00041 0.9649 0.00923 Colon cancer rs4726070 G A -0.017 0.50084 0.02521 Rectal cancer rs4726070 G A -0.0075 0.81425 0.03199 |
| Gastroesophageal reflux | rs4733264 | C | G | -0.0152 | 0.12948 | 0.01005 | Esophageal carcinoma | rs4733264 | C | G | -0.1423 | 0.02831 | 0.0649 | Acute gastritis | rs4733264 | C | G | 0.02252 | 0.47766 | 0.03172 | Chronic gastritis | rs4733264 | C | G | -0.0249 | 0.12086 | 0.01605 | Gastroduodenal ulcer | rs4733264 | C | G | -0.0003 | 0.98301 | 0.01642 | Gastric cancer | rs4733264 | C | G | -0.0618 | 0.13918 | 0.04176 | Fatty liver | rs4733264 | C | G | 0.02399 | 0.47206 | 0.03336 | Hepatic fibrosis | rs4733264 | C | G | -0.0303 | 0.81169 | 0.12722 | Cirrhosis | rs4733264 | C | G | -0.0079 | 0.74776 | 0.02454liver cancer rs4733264 C G -0.0116 0.83986 0.05736 Choleliths rs4733264 C G 0.00334 0.69454 0.0085 Acute pancreatitis rs4733264 C G -0.0129 0.51438 0.01979 Chronic pancreatitis rs4733264 C G -0.0371 0.16438 0.0267 Pancreatic cancer rs4733264 C G -0.0255 0.53668 0.04128 Ulcerative colitis rs4733264 C G -0.0268 0.22142 0.02194 Crohn's disease rs4733264 C G 0.05786 0.1263 0.03785 Irrtable bowel syndrome rs4733264 C G -0.0205 0.21189 0.01642 Acute appendicitis rs4733264 C G -0.0032 0.73338 0.00943 Colon cancer rs4733264 C G -0.0408 0.11366 0.02578 Rectal cancer rs4733264 C G 0.00847 0.79606 0.03276 |
| Gastroesophageal reflux | rs4757957 | C | G | -0.001 | 0.93176 | 0.0114 | Esophageal carcinoma | rs4757957 | C | G | -0.0635 | 0.39106 | 0.07409 | Acute gastritis | rs4757957 | C | G | -0.0547 | 0.12906 | 0.03603 | Chronic gastritis | rs4757957 | C | G | -0.0088 | 0.62946 | 0.01822 | Gastroduodenal ulcer | rs4757957 | C | G | -0.0121 | 0.5163 | 0.01868 | Gastric cancer | rs4757957 | C | G | -0.0648 | 0.1722 | 0.0475 | Fatty liver | rs4757957 | C | G | -0.0452 | 0.23311 | 0.03788 | Hepatic fibrosis | rs4757957 | C | G | 0.2557 | 0.07621 | 0.14421 | Cirrhosis | rs4757957 | C | G | -0.0128 | 0.64688 | 0.02795liver cancer rs4757957 C G 0.01003 0.87845 0.06556 Choleliths rs4757957 C G -0.0272 0.00466 0.00961 Acute pancreatitis rs4757957 C G -0.0211 0.34883 0.02253 Chronic pancreatitis rs4757957 C G -0.0158 0.60279 0.03041 Pancreatic cancer rs4757957 C G -0.1476 0.00121 0.04558 Ulcerative colitis rs4757957 C G -0.0107 0.66637 0.02489 Crohn's disease rs4757957 C G -0.0091 0.83202 0.04298 Irrtable bowel syndrome rs4757957 C G -0.011 0.55407 0.01863 Acute appendicitis rs4757957 C G 7.9E-05 0.99415 0.01072 Colon cancer rs4757957 C G -0.0123 0.67389 0.02931 Rectal cancer rs4757957 C G 0.00143 0.9694 0.0372 |
| Gastroesophageal reflux | rs4766424 | G | C | 0.02988 | 0.15154 | 0.02083 | Esophageal carcinoma | rs4766424 | G | C | -0.0248 | 0.85499 | 0.13549 | Acute gastritis | rs4766424 | G | C | 0.01975 | 0.76381 | 0.06573 | Chronic gastritis | rs4766424 | G | C | 0.00298 | 0.92837 | 0.03318 | Gastroduodenal ulcer | rs4766424 | G | C | 0.00707 | 0.83632 | 0.0342 | Gastric cancer | rs4766424 | G | C | 0.01979 | 0.81912 | 0.08653 | Fatty liver | rs4766424 | G | C | 0.05355 | 0.44099 | 0.0695 | Hepatic fibrosis | rs4766424 | G | C | -0.0827 | 0.75963 | 0.27044 | Cirrhosis | rs4766424 | G | C | -0.0397 | 0.44355 | 0.05184liver cancer rs4766424 G C -0.1042 0.38421 0.11976 Choleliths rs4766424 G C 0.04275 0.01552 0.01766 Acute pancreatitis rs4766424 G C 0.0447 0.2817 0.04153 Chronic pancreatitis rs4766424 G C 0.02169 0.70043 0.05637 Pancreatic cancer rs4766424 G C -0.0725 0.40093 0.08631 Ulcerative colitis rs4766424 G C 0.02725 0.55411 0.04606 Crohn's disease rs4766424 G C 0.01979 0.80299 0.07931 Irrtable bowel syndrome rs4766424 G C 0.0563 0.09938 0.03417 Acute appendicitis rs4766424 G C 0.01718 0.38458 0.01976 Colon cancer rs4766424 G C -0.0237 0.65585 0.05323 Rectal cancer rs4766424 G C 0.03953 0.55861 0.06758 |
| Gastroesophageal reflux | rs4778058 | C | T | 0.02215 | 0.02736 | 0.01004 | Esophageal carcinoma | rs4778058 | C | T | -0.0337 | 0.60452 | 0.06504 | Acute gastritis | rs4778058 | C | T | -0.0073 | 0.81859 | 0.03169 | Chronic gastritis | rs4778058 | C | T | -0.0124 | 0.44104 | 0.01604 | Gastroduodenal ulcer | rs4778058 | C | T | 0.01248 | 0.44695 | 0.01641 | Gastric cancer | rs4778058 | C | T | -0.0012 | 0.9778 | 0.04173 | Fatty liver | rs4778058 | C | T | 0.05363 | 0.10747 | 0.03332 | Hepatic fibrosis | rs4778058 | C | T | 0.20662 | 0.10488 | 0.12741 | Cirrhosis | rs4778058 | C | T | 0.00868 | 0.72377 | 0.02457liver cancer rs4778058 C T 0.03944 0.4916 0.05734 Choleliths rs4778058 C T 0.00602 0.47845 0.00849 Acute pancreatitis rs4778058 C T 0.02942 0.13727 0.0198 Chronic pancreatitis rs4778058 C T 0.01502 0.57346 0.02669 Pancreatic cancer rs4778058 C T 0.02599 0.52997 0.04138 Ulcerative colitis rs4778058 C T 0.02244 0.30552 0.0219 Crohn's disease rs4778058 C T 0.07532 0.0462 0.03778 Irrtable bowel syndrome rs4778058 C T 0.0087 0.59596 0.0164 Acute appendicitis rs4778058 C T 0.01128 0.23117 0.00942 Colon cancer rs4778058 C T 0.00234 0.92781 0.02581 Rectal cancer rs4778058 C T 0.03119 0.34057 0.03272 |
| Gastroesophageal reflux | rs4787457 | G | A | -0.0149 | 0.13202 | 0.00993 | Esophageal carcinoma | rs4787457 | G | A | 0.03438 | 0.59315 | 0.06436 | Acute gastritis | rs4787457 | G | A | -0.0008 | 0.97896 | 0.03139 | Chronic gastritis | rs4787457 | G | A | -0.0277 | 0.08143 | 0.01587 | Gastroduodenal ulcer | rs4787457 | G | A | 0.02333 | 0.15061 | 0.01623 | Gastric cancer | rs4787457 | G | A | 0.03798 | 0.35859 | 0.04137 | Fatty liver | rs4787457 | G | A | 0.06087 | 0.06468 | 0.03295 | Hepatic fibrosis | rs4787457 | G | A | -0.0727 | 0.5639 | 0.12596 | Cirrhosis | rs4787457 | G | A | 0.00935 | 0.70017 | 0.02428liver cancer rs4787457 G A -0.0451 0.42712 0.05674 Choleliths rs4787457 G A 0.01033 0.21828 0.00839 Acute pancreatitis rs4787457 G A -0.0281 0.15014 0.01956 Chronic pancreatitis rs4787457 G A -0.0532 0.04345 0.02634 Pancreatic cancer rs4787457 G A 0.05262 0.19784 0.04086 Ulcerative colitis rs4787457 G A 0.05323 0.01392 0.02164 Crohn's disease rs4787457 G A 0.06207 0.09662 0.03736 Irrtable bowel syndrome rs4787457 G A 0.00727 0.65356 0.0162 Acute appendicitis rs4787457 G A -0.0212 0.02314 0.00932 Colon cancer rs4787457 G A -0.0482 0.05852 0.0255 Rectal cancer rs4787457 G A 0.01573 0.62716 0.03238 |
| Gastroesophageal reflux | rs4810227 | A | G | -0.0152 | 0.12919 | 0.01002 | Esophageal carcinoma | rs4810227 | A | G | 0.03309 | 0.61073 | 0.06501 | Acute gastritis | rs4810227 | A | G | -0.006 | 0.84825 | 0.03161 | Chronic gastritis | rs4810227 | A | G | 0.00257 | 0.87269 | 0.01603 | Gastroduodenal ulcer | rs4810227 | A | G | -0.0096 | 0.55983 | 0.01639 | Gastric cancer | rs4810227 | A | G | 0.02639 | 0.52707 | 0.04172 | Fatty liver | rs4810227 | A | G | 0.05389 | 0.10535 | 0.03328 | Hepatic fibrosis | rs4810227 | A | G | 0.24741 | 0.05134 | 0.12697 | Cirrhosis | rs4810227 | A | G | 0.00696 | 0.77694 | 0.02456liver cancer rs4810227 A G -0.0226 0.69347 0.05736 Choleliths rs4810227 A G -0.0024 0.78032 0.00848 Acute pancreatitis rs4810227 A G 0.02014 0.30824 0.01977 Chronic pancreatitis rs4810227 A G -0.0139 0.60249 0.02665 Pancreatic cancer rs4810227 A G 0.06564 0.11224 0.04133 Ulcerative colitis rs4810227 A G 0.01317 0.54728 0.02188 Crohn's disease rs4810227 A G -0.0608 0.10731 0.03773 Irrtable bowel syndrome rs4810227 A G -0.0384 0.01906 0.01638 Acute appendicitis rs4810227 A G 0.00311 0.74139 0.00942 Colon cancer rs4810227 A G -0.02 0.43886 0.02577 Rectal cancer rs4810227 A G -0.0187 0.56681 0.03271 |
| Gastroesophageal reflux | rs4839155 | T | G | 0.00114 | 0.91713 | 0.01096 | Esophageal carcinoma | rs4839155 | T | G | -0.1116 | 0.1157 | 0.07095 | Acute gastritis | rs4839155 | T | G | 0.04663 | 0.17801 | 0.03462 | Chronic gastritis | rs4839155 | T | G | -0.014 | 0.42256 | 0.0175 | Gastroduodenal ulcer | rs4839155 | T | G | 0.01765 | 0.3243 | 0.0179 | Gastric cancer | rs4839155 | T | G | -0.0183 | 0.68692 | 0.0454 | Fatty liver | rs4839155 | T | G | 0.0056 | 0.87794 | 0.03644 | Hepatic fibrosis | rs4839155 | T | G | 0.04205 | 0.76284 | 0.13935 | Cirrhosis | rs4839155 | T | G | 0.00409 | 0.87867 | 0.02679liver cancer rs4839155 T G -0.0271 0.66423 0.06248 Choleliths rs4839155 T G 0.01019 0.27228 0.00928 Acute pancreatitis rs4839155 T G 0.03998 0.06438 0.02161 Chronic pancreatitis rs4839155 T G 0.07937 0.00687 0.02936 Pancreatic cancer rs4839155 T G -0.0075 0.86845 0.04518 Ulcerative colitis rs4839155 T G 0.03138 0.19129 0.02401 Crohn's disease rs4839155 T G 0.03377 0.41523 0.04145 Irrtable bowel syndrome rs4839155 T G 0.03893 0.03006 0.01794 Acute appendicitis rs4839155 T G 0.0103 0.31825 0.01032 Colon cancer rs4839155 T G 0.04056 0.14917 0.02812 Rectal cancer rs4839155 T G -0.0265 0.45711 0.03559 |
| Gastroesophageal reflux | rs4846724 | G | A | -0.0198 | 0.04586 | 0.00991 | Esophageal carcinoma | rs4846724 | G | A | 0.04984 | 0.43879 | 0.06438 | Acute gastritis | rs4846724 | G | A | 0.00939 | 0.76407 | 0.03127 | Chronic gastritis | rs4846724 | G | A | 0.00577 | 0.71532 | 0.01583 | Gastroduodenal ulcer | rs4846724 | G | A | -0.0037 | 0.81753 | 0.01621 | Gastric cancer | rs4846724 | G | A | 0.05466 | 0.18529 | 0.04127 | Fatty liver | rs4846724 | G | A | -0.0471 | 0.15249 | 0.03292 | Hepatic fibrosis | rs4846724 | G | A | -0.0702 | 0.57564 | 0.12549 | Cirrhosis | rs4846724 | G | A | -0.0245 | 0.31232 | 0.02426liver cancer rs4846724 G A -0.0825 0.14567 0.05668 Choleliths rs4846724 G A -0.0002 0.98239 0.00838 Acute pancreatitis rs4846724 G A 0.03461 0.07662 0.01955 Chronic pancreatitis rs4846724 G A -0.0021 0.93557 0.02636 Pancreatic cancer rs4846724 G A -0.0179 0.66145 0.04094 Ulcerative colitis rs4846724 G A 0.03466 0.10886 0.02162 Crohn's disease rs4846724 G A 0.0021 0.95516 0.03731 Irrtable bowel syndrome rs4846724 G A -0.0049 0.76313 0.0162 Acute appendicitis rs4846724 G A 0.0045 0.6286 0.00931 Colon cancer rs4846724 G A 0.00788 0.75695 0.02547 Rectal cancer rs4846724 G A -0.0746 0.02115 0.03237 |
| Gastroesophageal reflux | rs4870482 | G | C | 0.00936 | 0.39065 | 0.01091 | Esophageal carcinoma | rs4870482 | G | C | -0.0463 | 0.51086 | 0.07047 | Acute gastritis | rs4870482 | G | C | 0.0073 | 0.83209 | 0.03443 | Chronic gastritis | rs4870482 | G | C | 0.0034 | 0.84536 | 0.01743 | Gastroduodenal ulcer | rs4870482 | G | C | -0.0295 | 0.09807 | 0.01781 | Gastric cancer | rs4870482 | G | C | 0.0423 | 0.35084 | 0.04534 | Fatty liver | rs4870482 | G | C | -0.0399 | 0.27024 | 0.03621 | Hepatic fibrosis | rs4870482 | G | C | -0.0797 | 0.56281 | 0.1377 | Cirrhosis | rs4870482 | G | C | -0.0205 | 0.44054 | 0.02662liver cancer rs4870482 G C 0.06863 0.26769 0.06192 Choleliths rs4870482 G C 0.00087 0.92472 0.00922 Acute pancreatitis rs4870482 G C 0.00317 0.88288 0.02149 Chronic pancreatitis rs4870482 G C -0.0038 0.89445 0.02894 Pancreatic cancer rs4870482 G C 0.01791 0.68895 0.04473 Ulcerative colitis rs4870482 G C 0.00104 0.96525 0.02382 Crohn's disease rs4870482 G C -0.031 0.45086 0.04105 Irrtable bowel syndrome rs4870482 G C 0.00563 0.75197 0.01781 Acute appendicitis rs4870482 G C -0.0071 0.49058 0.01024 Colon cancer rs4870482 G C -0.0182 0.5169 0.02801 Rectal cancer rs4870482 G C 0.06923 0.05119 0.03551 |
| Gastroesophageal reflux | rs4888746 | G | A | -0.0161 | 0.10506 | 0.00994 | Esophageal carcinoma | rs4888746 | G | A | 0.00729 | 0.90958 | 0.06417 | Acute gastritis | rs4888746 | G | A | -0.0072 | 0.81819 | 0.03138 | Chronic gastritis | rs4888746 | G | A | 0.01265 | 0.42592 | 0.01589 | Gastroduodenal ulcer | rs4888746 | G | A | 0.01457 | 0.36999 | 0.01625 | Gastric cancer | rs4888746 | G | A | -0.0539 | 0.19227 | 0.04134 | Fatty liver | rs4888746 | G | A | 0.0197 | 0.55055 | 0.03299 | Hepatic fibrosis | rs4888746 | G | A | -0.036 | 0.77448 | 0.12551 | Cirrhosis | rs4888746 | G | A | -0.0432 | 0.07532 | 0.02429liver cancer rs4888746 G A 0.07845 0.165 0.0565 Choleliths rs4888746 G A -0.0056 0.5047 0.0084 Acute pancreatitis rs4888746 G A -0.015 0.44367 0.01958 Chronic pancreatitis rs4888746 G A 0.00589 0.82341 0.02638 Pancreatic cancer rs4888746 G A 0.01223 0.7647 0.04088 Ulcerative colitis rs4888746 G A 0.03354 0.12187 0.02168 Crohn's disease rs4888746 G A -0.0659 0.07841 0.03742 Irrtable bowel syndrome rs4888746 G A 0.0157 0.33356 0.01624 Acute appendicitis rs4888746 G A 0.01043 0.26341 0.00933 Colon cancer rs4888746 G A -0.0295 0.2473 0.02549 Rectal cancer rs4888746 G A -0.0728 0.02451 0.03237 |
| Gastroesophageal reflux | rs4895650 | T | C | -0.0063 | 0.52044 | 0.00984 | Esophageal carcinoma | rs4895650 | T | C | 0.06442 | 0.31316 | 0.06387 | Acute gastritis | rs4895650 | T | C | 0.02261 | 0.46675 | 0.03106 | Chronic gastritis | rs4895650 | T | C | 0.0195 | 0.21533 | 0.01574 | Gastroduodenal ulcer | rs4895650 | T | C | 0.01464 | 0.36302 | 0.01609 | Gastric cancer | rs4895650 | T | C | -0.0057 | 0.88843 | 0.04098 | Fatty liver | rs4895650 | T | C | -0.0357 | 0.27361 | 0.03265 | Hepatic fibrosis | rs4895650 | T | C | 0.09286 | 0.45618 | 0.12462 | Cirrhosis | rs4895650 | T | C | 0.03913 | 0.10408 | 0.02407liver cancer rs4895650 T C 0.0414 0.4619 0.05627 Choleliths rs4895650 T C -0.0054 0.51534 0.00832 Acute pancreatitis rs4895650 T C 0.00103 0.95769 0.01939 Chronic pancreatitis rs4895650 T C 0.00136 0.95863 0.02615 Pancreatic cancer rs4895650 T C -0.0335 0.40922 0.04063 Ulcerative colitis rs4895650 T C -0.0136 0.52659 0.02145 Crohn's disease rs4895650 T C -0.0255 0.49013 0.03699 Irrtable bowel syndrome rs4895650 T C -0.0045 0.77957 0.01608 Acute appendicitis rs4895650 T C -0.0115 0.21194 0.00924 Colon cancer rs4895650 T C 0.00017 0.99467 0.02528 Rectal cancer rs4895650 T C -0.0767 0.01711 0.03215 |
| Gastroesophageal reflux | rs4945424 | A | C | -0.0049 | 0.62055 | 0.00988 | Esophageal carcinoma | rs4945424 | A | C | -0.0353 | 0.58142 | 0.064 | Acute gastritis | rs4945424 | A | C | -0.0027 | 0.93051 | 0.03121 | Chronic gastritis | rs4945424 | A | C | 0.00744 | 0.63732 | 0.01579 | Gastroduodenal ulcer | rs4945424 | A | C | 0.02768 | 0.08658 | 0.01615 | Gastric cancer | rs4945424 | A | C | -0.0148 | 0.71967 | 0.04113 | Fatty liver | rs4945424 | A | C | -0.0017 | 0.95774 | 0.0328 | Hepatic fibrosis | rs4945424 | A | C | -0.0087 | 0.94465 | 0.12547 | Cirrhosis | rs4945424 | A | C | 0.01376 | 0.56853 | 0.02414liver cancer rs4945424 A C -0.0765 0.17477 0.05636 Choleliths rs4945424 A C -0.001 0.90766 0.00835 Acute pancreatitis rs4945424 A C -0.0031 0.87538 0.01945 Chronic pancreatitis rs4945424 A C 0.01815 0.48867 0.02621 Pancreatic cancer rs4945424 A C 0.0345 0.39623 0.04067 Ulcerative colitis rs4945424 A C 0.00686 0.75054 0.02157 Crohn's disease rs4945424 A C 0.01029 0.782 0.0372 Irrtable bowel syndrome rs4945424 A C 0.0142 0.37905 0.01614 Acute appendicitis rs4945424 A C 0.00327 0.72479 0.00928 Colon cancer rs4945424 A C 0.01507 0.55235 0.02536 Rectal cancer rs4945424 A C -0.0235 0.46506 0.03218 |
| Gastroesophageal reflux | rs4964046 | G | A | 0.01706 | 0.08662 | 0.00995 | Esophageal carcinoma | rs4964046 | G | A | -0.0355 | 0.58182 | 0.06443 | Acute gastritis | rs4964046 | G | A | 0.06647 | 0.03471 | 0.03148 | Chronic gastritis | rs4964046 | G | A | -0.0269 | 0.0906 | 0.01592 | Gastroduodenal ulcer | rs4964046 | G | A | 0.0119 | 0.46521 | 0.01629 | Gastric cancer | rs4964046 | G | A | 0.0104 | 0.80151 | 0.04136 | Fatty liver | rs4964046 | G | A | 0.00254 | 0.9388 | 0.03305 | Hepatic fibrosis | rs4964046 | G | A | 0.12441 | 0.32343 | 0.126 | Cirrhosis | rs4964046 | G | A | -0.0249 | 0.30636 | 0.02435liver cancer rs4964046 G A -0.0553 0.33116 0.05692 Choleliths rs4964046 G A 0.00615 0.4646 0.00842 Acute pancreatitis rs4964046 G A 0.03798 0.05294 0.01962 Chronic pancreatitis rs4964046 G A -0.0218 0.40889 0.02644 Pancreatic cancer rs4964046 G A 0.00413 0.9199 0.04105 Ulcerative colitis rs4964046 G A -0.0202 0.35284 0.02174 Crohn's disease rs4964046 G A -0.0049 0.89618 0.03752 Irrtable bowel syndrome rs4964046 G A -0.0164 0.31286 0.01628 Acute appendicitis rs4964046 G A 0.00261 0.7803 0.00936 Colon cancer rs4964046 G A 0.00708 0.78159 0.02555 Rectal cancer rs4964046 G A 0.03728 0.25016 0.03242 |
| Gastroesophageal reflux | rs4972400 | G | A | 0.00715 | 0.48508 | 0.01024 | Esophageal carcinoma | rs4972400 | G | A | 0.0531 | 0.42086 | 0.06596 | Acute gastritis | rs4972400 | G | A | -0.0049 | 0.87936 | 0.03233 | Chronic gastritis | rs4972400 | G | A | -0.0099 | 0.54484 | 0.01636 | Gastroduodenal ulcer | rs4972400 | G | A | -0.0082 | 0.62481 | 0.01673 | Gastric cancer | rs4972400 | G | A | 0.04349 | 0.30586 | 0.04247 | Fatty liver | rs4972400 | G | A | -0.0253 | 0.4564 | 0.034 | Hepatic fibrosis | rs4972400 | G | A | 0.01142 | 0.93002 | 0.13008 | Cirrhosis | rs4972400 | G | A | -0.0122 | 0.62557 | 0.02503liver cancer rs4972400 G A 0.00091 0.98754 0.05828 Choleliths rs4972400 G A -0.0166 0.05429 0.00865 Acute pancreatitis rs4972400 G A -0.0057 0.77621 0.02018 Chronic pancreatitis rs4972400 G A 0.00485 0.85863 0.02721 Pancreatic cancer rs4972400 G A -0.0298 0.47838 0.04206 Ulcerative colitis rs4972400 G A 0.04096 0.06702 0.02237 Crohn's disease rs4972400 G A 0.02216 0.56577 0.03859 Irrtable bowel syndrome rs4972400 G A 0.00177 0.91589 0.01675 Acute appendicitis rs4972400 G A -0.0122 0.20659 0.00963 Colon cancer rs4972400 G A 0.02784 0.28725 0.02616 Rectal cancer rs4972400 G A 0.02825 0.39419 0.03316 |
| Gastroesophageal reflux | rs4984541 | A | G | 0.01233 | 0.26499 | 0.01106 | Esophageal carcinoma | rs4984541 | A | G | 0.05524 | 0.43942 | 0.07145 | Acute gastritis | rs4984541 | A | G | -0.0263 | 0.45173 | 0.035 | Chronic gastritis | rs4984541 | A | G | 0.00785 | 0.6571 | 0.01768 | Gastroduodenal ulcer | rs4984541 | A | G | 0.02474 | 0.17186 | 0.01811 | Gastric cancer | rs4984541 | A | G | -0.0691 | 0.13402 | 0.04611 | Fatty liver | rs4984541 | A | G | -0.032 | 0.38285 | 0.03671 | Hepatic fibrosis | rs4984541 | A | G | 0.10253 | 0.46558 | 0.14052 | Cirrhosis | rs4984541 | A | G | 0.02626 | 0.33145 | 0.02704liver cancer rs4984541 A G -0.0515 0.41528 0.06319 Choleliths rs4984541 A G 0.00421 0.65324 0.00937 Acute pancreatitis rs4984541 A G 0.01909 0.38135 0.02181 Chronic pancreatitis rs4984541 A G 0.00845 0.77372 0.0294 Pancreatic cancer rs4984541 A G 0.00133 0.97674 0.04567 Ulcerative colitis rs4984541 A G -0.0045 0.8532 0.02412 Crohn's disease rs4984541 A G -0.0473 0.25615 0.04161 Irrtable bowel syndrome rs4984541 A G 0.03187 0.07818 0.01809 Acute appendicitis rs4984541 A G -0.0088 0.39561 0.01039 Colon cancer rs4984541 A G -0.0088 0.75745 0.02839 Rectal cancer rs4984541 A G 0.01152 0.7488 0.03599 |
| Gastroesophageal reflux | rs510706 | G | C | 0.02421 | 0.01712 | 0.01016 | Esophageal carcinoma | rs510706 | G | C | 0.01025 | 0.87647 | 0.06596 | Acute gastritis | rs510706 | G | C | 0.0056 | 0.86144 | 0.0321 | Chronic gastritis | rs510706 | G | C | -0.0228 | 0.16055 | 0.01623 | Gastroduodenal ulcer | rs510706 | G | C | 0.01272 | 0.44432 | 0.01663 | Gastric cancer | rs510706 | G | C | -0.0203 | 0.63152 | 0.04232 | Fatty liver | rs510706 | G | C | 0.0252 | 0.45558 | 0.03378 | Hepatic fibrosis | rs510706 | G | C | 0.04329 | 0.73731 | 0.12908 | Cirrhosis | rs510706 | G | C | 0.02603 | 0.29618 | 0.02492liver cancer rs510706 G C 0.00265 0.96353 0.05806 Choleliths rs510706 G C -0.0008 0.9234 0.00859 Acute pancreatitis rs510706 G C 0.01073 0.59259 0.02005 Chronic pancreatitis rs510706 G C 0.02474 0.36024 0.02705 Pancreatic cancer rs510706 G C 0.03308 0.43067 0.04197 Ulcerative colitis rs510706 G C -0.0131 0.55696 0.02223 Crohn's disease rs510706 G C 0.04128 0.28176 0.03835 Irrtable bowel syndrome rs510706 G C 0.00063 0.96977 0.01661 Acute appendicitis rs510706 G C 0.01612 0.09169 0.00956 Colon cancer rs510706 G C 0.0084 0.74758 0.0261 Rectal cancer rs510706 G C -0.0848 0.01055 0.03316 |
| Gastroesophageal reflux | rs532799 | G | A | ####### | 0.99879 | 0.01106 | Esophageal carcinoma | rs532799 | G | A | 0.10614 | 0.1388 | 0.0717 | Acute gastritis | rs532799 | G | A | -0.0313 | 0.36894 | 0.03485 | Chronic gastritis | rs532799 | G | A | 0.01452 | 0.41082 | 0.01765 | Gastroduodenal ulcer | rs532799 | G | A | -0.0005 | 0.97957 | 0.01807 | Gastric cancer | rs532799 | G | A | 0.1029 | 0.02422 | 0.04566 | Fatty liver | rs532799 | G | A | 0.0583 | 0.11253 | 0.03674 | Hepatic fibrosis | rs532799 | G | A | 0.04001 | 0.77586 | 0.14051 | Cirrhosis | rs532799 | G | A | 0.02249 | 0.4067 | 0.0271liver cancer rs532799 G A 0.05672 0.36768 0.06297 Choleliths rs532799 G A -0.0125 0.17932 0.00934 Acute pancreatitis rs532799 G A 0.01037 0.63455 0.02183 Chronic pancreatitis rs532799 G A -0.0207 0.4827 0.02949 Pancreatic cancer rs532799 G A -0.0564 0.21445 0.04547 Ulcerative colitis rs532799 G A -0.0197 0.41453 0.02417 Crohn's disease rs532799 G A 0.01295 0.75608 0.0417 Irrtable bowel syndrome rs532799 G A 0.00717 0.69145 0.01807 Acute appendicitis rs532799 G A 0.01883 0.07039 0.0104 Colon cancer rs532799 G A -0.0066 0.81532 0.02829 Rectal cancer rs532799 G A -0.0128 0.72184 0.03601 |
| Gastroesophageal reflux | rs535307 | A | G | -0.0003 | 0.97773 | 0.01166 | Esophageal carcinoma | rs535307 | A | G | -0.1028 | 0.16902 | 0.07471 | Acute gastritis | rs535307 | A | G | -0.0288 | 0.43235 | 0.03674 | Chronic gastritis | rs535307 | A | G | 0.00893 | 0.63138 | 0.01861 | Gastroduodenal ulcer | rs535307 | A | G | 0.00379 | 0.84238 | 0.01907 | Gastric cancer | rs535307 | A | G | -0.0092 | 0.84914 | 0.04817 | Fatty liver | rs535307 | A | G | -0.0419 | 0.27862 | 0.03869 | Hepatic fibrosis | rs535307 | A | G | 0.09881 | 0.5046 | 0.14808 | Cirrhosis | rs535307 | A | G | 0.02571 | 0.3679 | 0.02855liver cancer rs535307 A G -0.0223 0.73588 0.06625 Choleliths rs535307 A G -0.0096 0.33014 0.00983 Acute pancreatitis rs535307 A G 0.01024 0.65645 0.02302 Chronic pancreatitis rs535307 A G -0.0193 0.53525 0.03108 Pancreatic cancer rs535307 A G -0.0749 0.11663 0.04775 Ulcerative colitis rs535307 A G 0.03526 0.16597 0.02545 Crohn's disease rs535307 A G -0.0019 0.96628 0.04391 Irrtable bowel syndrome rs535307 A G -0.0053 0.78072 0.01908 Acute appendicitis rs535307 A G -0.0054 0.62378 0.01096 Colon cancer rs535307 A G -0.0195 0.51086 0.02967 Rectal cancer rs535307 A G 0.03149 0.40255 0.03762 |
| Gastroesophageal reflux | rs55736314 | C | G | -0.005 | 0.62728 | 0.0102 | Esophageal carcinoma | rs55736314 | C | G | -0.0134 | 0.83961 | 0.06603 | Acute gastritis | rs55736314 | C | G | 0.03139 | 0.32918 | 0.03217 | Chronic gastritis | rs55736314 | C | G | 0.01738 | 0.28672 | 0.01632 | Gastroduodenal ulcer | rs55736314 | C | G | 0.02078 | 0.21254 | 0.01667 | Gastric cancer | rs55736314 | C | G | 0.0039 | 0.92654 | 0.04232 | Fatty liver | rs55736314 | C | G | -0.0194 | 0.56698 | 0.03386 | Hepatic fibrosis | rs55736314 | C | G | 0.15241 | 0.23701 | 0.12889 | Cirrhosis | rs55736314 | C | G | -0.0419 | 0.09252 | 0.02492liver cancer rs55736314 C G -0.0477 0.41307 0.05829 Choleliths rs55736314 C G 0.02163 0.01218 0.00863 Acute pancreatitis rs55736314 C G 0.03098 0.12254 0.02006 Chronic pancreatitis rs55736314 C G 0.00743 0.78333 0.02702 Pancreatic cancer rs55736314 C G -0.0346 0.40963 0.04196 Ulcerative colitis rs55736314 C G -0.0129 0.56202 0.02222 Crohn's disease rs55736314 C G -0.0168 0.66242 0.03837 Irrtable bowel syndrome rs55736314 C G -0.0271 0.10366 0.01668 Acute appendicitis rs55736314 C G -0.0165 0.0851 0.00957 Colon cancer rs55736314 C G -0.0357 0.17198 0.02617 Rectal cancer rs55736314 C G -0.0774 0.0199 0.03324 |
| Gastroesophageal reflux | rs55771711 | C | G | -0.0299 | 0.00905 | 0.01145 | Esophageal carcinoma | rs55771711 | C | G | 0.20177 | 0.00399 | 0.07008 | Acute gastritis | rs55771711 | C | G | -0.0196 | 0.58546 | 0.03603 | Chronic gastritis | rs55771711 | C | G | -0.0251 | 0.16935 | 0.01824 | Gastroduodenal ulcer | rs55771711 | C | G | -0.0132 | 0.47824 | 0.01867 | Gastric cancer | rs55771711 | C | G | 0.06655 | 0.16121 | 0.04751 | Fatty liver | rs55771711 | C | G | -0.075 | 0.04788 | 0.03791 | Hepatic fibrosis | rs55771711 | C | G | 0.03737 | 0.79599 | 0.14456 | Cirrhosis | rs55771711 | C | G | -0.0065 | 0.81451 | 0.02791liver cancer rs55771711 C G 0.01249 0.84783 0.06511 Choleliths rs55771711 C G -0.0056 0.56225 0.00965 Acute pancreatitis rs55771711 C G -0.0504 0.02514 0.0225 Chronic pancreatitis rs55771711 C G -0.0494 0.10306 0.03032 Pancreatic cancer rs55771711 C G -0.0999 0.03431 0.0472 Ulcerative colitis rs55771711 C G -0.0082 0.74179 0.02493 Crohn's disease rs55771711 C G -0.0047 0.91354 0.04302 Irrtable bowel syndrome rs55771711 C G -0.0111 0.55109 0.01862 Acute appendicitis rs55771711 C G 0.02312 0.03117 0.01073 Colon cancer rs55771711 C G -0.0076 0.79405 0.02926 Rectal cancer rs55771711 C G -0.0188 0.61259 0.03711 |
| Gastroesophageal reflux | rs56391344 | A | G | -0.0042 | 0.72772 | 0.012 | Esophageal carcinoma | rs56391344 | A | G | 0.01643 | 0.83274 | 0.0778 | Acute gastritis | rs56391344 | A | G | -0.0179 | 0.63681 | 0.03799 | Chronic gastritis | rs56391344 | A | G | 0.00891 | 0.64234 | 0.01919 | Gastroduodenal ulcer | rs56391344 | A | G | -0.0373 | 0.05798 | 0.01966 | Gastric cancer | rs56391344 | A | G | -0.0474 | 0.34204 | 0.0499 | Fatty liver | rs56391344 | A | G | 0.04014 | 0.31346 | 0.03982 | Hepatic fibrosis | rs56391344 | A | G | -0.0001 | 0.99938 | 0.15249 | Cirrhosis | rs56391344 | A | G | 0.01443 | 0.62274 | 0.02933liver cancer rs56391344 A G 0.08222 0.2284 0.06826 Choleliths rs56391344 A G 0.00882 0.38561 0.01017 Acute pancreatitis rs56391344 A G 0.00509 0.82966 0.02367 Chronic pancreatitis rs56391344 A G 0.04628 0.14625 0.03185 Pancreatic cancer rs56391344 A G 0.03245 0.51168 0.04945 Ulcerative colitis rs56391344 A G 0.00295 0.91022 0.0262 Crohn's disease rs56391344 A G 0.04371 0.33397 0.04524 Irrtable bowel syndrome rs56391344 A G 0.01102 0.57484 0.01964 Acute appendicitis rs56391344 A G 0.00475 0.67401 0.01129 Colon cancer rs56391344 A G 0.02006 0.51435 0.03076 Rectal cancer rs56391344 A G 0.04757 0.22268 0.03901 |
| Gastroesophageal reflux | rs575113 | A | G | -0.0169 | 0.10904 | 0.01054 | Esophageal carcinoma | rs575113 | A | G | 0.03286 | 0.62998 | 0.06822 | Acute gastritis | rs575113 | A | G | -0.0153 | 0.64537 | 0.03325 | Chronic gastritis | rs575113 | A | G | -0.0226 | 0.17855 | 0.01683 | Gastroduodenal ulcer | rs575113 | A | G | -0.0265 | 0.12412 | 0.01722 | Gastric cancer | rs575113 | A | G | -0.0544 | 0.21475 | 0.04382 | Fatty liver | rs575113 | A | G | 0.04557 | 0.19207 | 0.03494 | Hepatic fibrosis | rs575113 | A | G | 0.10024 | 0.45233 | 0.13338 | Cirrhosis | rs575113 | A | G | 0.02026 | 0.43124 | 0.02574liver cancer rs575113 A G 0.01683 0.77961 0.06014 Choleliths rs575113 A G 0.01378 0.122 0.00891 Acute pancreatitis rs575113 A G 0.01209 0.56062 0.02078 Chronic pancreatitis rs575113 A G -0.0121 0.66548 0.02797 Pancreatic cancer rs575113 A G 0.00271 0.95017 0.04331 Ulcerative colitis rs575113 A G 0.00175 0.93935 0.023 Crohn's disease rs575113 A G -0.007 0.86039 0.03968 Irrtable bowel syndrome rs575113 A G -0.0066 0.70027 0.0172 Acute appendicitis rs575113 A G 0.00512 0.60506 0.00989 Colon cancer rs575113 A G 0.01208 0.65418 0.02696 Rectal cancer rs575113 A G -0.0313 0.36052 0.03426 |
| Gastroesophageal reflux | rs59123361 | A | G | 0.01386 | 0.50292 | 0.0207 | Esophageal carcinoma | rs59123361 | A | G | 0.11516 | 0.38596 | 0.13283 | Acute gastritis | rs59123361 | A | G | -0.061 | 0.35315 | 0.06565 | Chronic gastritis | rs59123361 | A | G | 0.02857 | 0.38706 | 0.03303 | Gastroduodenal ulcer | rs59123361 | A | G | 0.05472 | 0.10656 | 0.0339 | Gastric cancer | rs59123361 | A | G | 0.04093 | 0.62881 | 0.08468 | Fatty liver | rs59123361 | A | G | 0.01181 | 0.86413 | 0.06902 | Hepatic fibrosis | rs59123361 | A | G | -0.136 | 0.61709 | 0.27199 | Cirrhosis | rs59123361 | A | G | -0.089 | 0.08254 | 0.05124liver cancer rs59123361 A G 0.03515 0.76546 0.11783 Choleliths rs59123361 A G 0.00237 0.89209 0.01749 Acute pancreatitis rs59123361 A G 0.08997 0.02822 0.041 Chronic pancreatitis rs59123361 A G 0.0922 0.09812 0.05575 Pancreatic cancer rs59123361 A G -0.0476 0.57884 0.08579 Ulcerative colitis rs59123361 A G -0.0009 0.98437 0.04569 Crohn's disease rs59123361 A G -0.162 0.03901 0.07849 Irrtable bowel syndrome rs59123361 A G 0.05643 0.09648 0.03395 Acute appendicitis rs59123361 A G -0.0192 0.32725 0.0196 Colon cancer rs59123361 A G 0.02614 0.6182 0.05245 Rectal cancer rs59123361 A G -0.0185 0.78094 0.06664 |
| Gastroesophageal reflux | rs59480703 | G | C | 0.00239 | 0.8386 | 0.01174 | Esophageal carcinoma | rs59480703 | G | C | -0.0806 | 0.29105 | 0.07631 | Acute gastritis | rs59480703 | G | C | 0.01568 | 0.67219 | 0.03707 | Chronic gastritis | rs59480703 | G | C | -0.0004 | 0.98128 | 0.01877 | Gastroduodenal ulcer | rs59480703 | G | C | 0.00851 | 0.65776 | 0.0192 | Gastric cancer | rs59480703 | G | C | 0.09231 | 0.06002 | 0.04908 | Fatty liver | rs59480703 | G | C | 0.025 | 0.52075 | 0.03893 | Hepatic fibrosis | rs59480703 | G | C | -0.0701 | 0.63653 | 0.14824 | Cirrhosis | rs59480703 | G | C | -0.0724 | 0.01025 | 0.02822liver cancer rs59480703 G C -0.1149 0.08711 0.06713 Choleliths rs59480703 G C 0.02833 0.00446 0.00996 Acute pancreatitis rs59480703 G C 0.01049 0.64977 0.02309 Chronic pancreatitis rs59480703 G C 0.04845 0.11971 0.03114 Pancreatic cancer rs59480703 G C 0.08999 0.06383 0.04855 Ulcerative colitis rs59480703 G C -0.0045 0.8614 0.02556 Crohn's disease rs59480703 G C 0.03551 0.42108 0.04413 Irrtable bowel syndrome rs59480703 G C -0.0212 0.26895 0.01914 Acute appendicitis rs59480703 G C -0.0028 0.79674 0.01101 Colon cancer rs59480703 G C 0.01661 0.58212 0.03018 Rectal cancer rs59480703 G C -0.0188 0.62404 0.03844 |
| Gastroesophageal reflux | rs60483752 | G | C | -0.0049 | 0.61723 | 0.00985 | Esophageal carcinoma | rs60483752 | G | C | -0.0394 | 0.5378 | 0.06392 | Acute gastritis | rs60483752 | G | C | -0.0165 | 0.5955 | 0.0311 | Chronic gastritis | rs60483752 | G | C | -0.0096 | 0.54189 | 0.01575 | Gastroduodenal ulcer | rs60483752 | G | C | 0.02965 | 0.06601 | 0.01613 | Gastric cancer | rs60483752 | G | C | 0.00033 | 0.99357 | 0.04103 | Fatty liver | rs60483752 | G | C | 0.02686 | 0.41124 | 0.03269 | Hepatic fibrosis | rs60483752 | G | C | 0.05362 | 0.66703 | 0.12464 | Cirrhosis | rs60483752 | G | C | 0.00413 | 0.86403 | 0.0241liver cancer rs60483752 G C -0.051 0.3645 0.05628 Choleliths rs60483752 G C -0.0036 0.66231 0.00833 Acute pancreatitis rs60483752 G C 0.00088 0.96406 0.01943 Chronic pancreatitis rs60483752 G C 0.02502 0.3389 0.02616 Pancreatic cancer rs60483752 G C 0.01899 0.64025 0.04064 Ulcerative colitis rs60483752 G C 0.0413 0.05472 0.0215 Crohn's disease rs60483752 G C -0.023 0.53557 0.03711 Irrtable bowel syndrome rs60483752 G C -0.0125 0.43586 0.01608 Acute appendicitis rs60483752 G C -0.009 0.32808 0.00925 Colon cancer rs60483752 G C 0.00764 0.76287 0.02532 Rectal cancer rs60483752 G C -0.0036 0.91111 0.03211 |
| Gastroesophageal reflux | rs60904894 | A | C | 0.00844 | 0.39013 | 0.00982 | Esophageal carcinoma | rs60904894 | A | C | 0.14389 | 0.02349 | 0.06352 | Acute gastritis | rs60904894 | A | C | -0.0053 | 0.8647 | 0.03101 | Chronic gastritis | rs60904894 | A | C | -0.0043 | 0.78203 | 0.01572 | Gastroduodenal ulcer | rs60904894 | A | C | -0.0018 | 0.9092 | 0.01607 | Gastric cancer | rs60904894 | A | C | -0.01 | 0.80719 | 0.04077 | Fatty liver | rs60904894 | A | C | -0.0128 | 0.69419 | 0.03262 | Hepatic fibrosis | rs60904894 | A | C | -0.0342 | 0.78362 | 0.12467 | Cirrhosis | rs60904894 | A | C | 0.03728 | 0.12105 | 0.02405liver cancer rs60904894 A C -0.0393 0.48362 0.05604 Choleliths rs60904894 A C -0.0103 0.21704 0.00831 Acute pancreatitis rs60904894 A C 0.01642 0.39693 0.01938 Chronic pancreatitis rs60904894 A C -0.0558 0.03264 0.02613 Pancreatic cancer rs60904894 A C -0.0095 0.81396 0.04041 Ulcerative colitis rs60904894 A C -0.0198 0.35605 0.02146 Crohn's disease rs60904894 A C -0.0047 0.89981 0.03701 Irrtable bowel syndrome rs60904894 A C 0.00664 0.67934 0.01605 Acute appendicitis rs60904894 A C -0.0082 0.37147 0.00923 Colon cancer rs60904894 A C -0.0226 0.36995 0.0252 Rectal cancer rs60904894 A C 0.0579 0.0702 0.03198 |
| Gastroesophageal reflux | rs6123924 | A | G | -0.0074 | 0.62212 | 0.01498 | Esophageal carcinoma | rs6123924 | A | G | 0.0519 | 0.59413 | 0.0974 | Acute gastritis | rs6123924 | A | G | 0.02685 | 0.57121 | 0.04741 | Chronic gastritis | rs6123924 | A | G | -0.0687 | 0.00341 | 0.02347 | Gastroduodenal ulcer | rs6123924 | A | G | 0.04815 | 0.04979 | 0.02455 | Gastric cancer | rs6123924 | A | G | -0.0002 | 0.99787 | 0.06218 | Fatty liver | rs6123924 | A | G | -0.0836 | 0.09385 | 0.04993 | Hepatic fibrosis | rs6123924 | A | G | -0.3306 | 0.08473 | 0.19178 | Cirrhosis | rs6123924 | A | G | 0.02224 | 0.54682 | 0.03692liver cancer rs6123924 A G -0.1565 0.06835 0.08587 Choleliths rs6123924 A G 0.0065 0.6069 0.01264 Acute pancreatitis rs6123924 A G -0.0298 0.31487 0.02969 Chronic pancreatitis rs6123924 A G -0.0025 0.95109 0.04025 Pancreatic cancer rs6123924 A G 0.06874 0.2672 0.06195 Ulcerative colitis rs6123924 A G -0.0189 0.56636 0.03299 Crohn's disease rs6123924 A G -0.0153 0.78822 0.05678 Irrtable bowel syndrome rs6123924 A G 0.0047 0.84841 0.02457 Acute appendicitis rs6123924 A G 0.01283 0.36542 0.01417 Colon cancer rs6123924 A G -0.0495 0.19676 0.03838 Rectal cancer rs6123924 A G -0.0006 0.98941 0.04885 |
| Gastroesophageal reflux | rs613872 | G | T | -0.0287 | 0.0323 | 0.01341 | Esophageal carcinoma | rs613872 | G | T | -0.0313 | 0.71866 | 0.08698 | Acute gastritis | rs613872 | G | T | -0.0386 | 0.36099 | 0.04226 | Chronic gastritis | rs613872 | G | T | 0.01417 | 0.50761 | 0.02139 | Gastroduodenal ulcer | rs613872 | G | T | -0.001 | 0.96281 | 0.02191 | Gastric cancer | rs613872 | G | T | 0.00359 | 0.94883 | 0.05587 | Fatty liver | rs613872 | G | T | 0.10962 | 0.01382 | 0.04453 | Hepatic fibrosis | rs613872 | G | T | -0.0974 | 0.56699 | 0.17014 | Cirrhosis | rs613872 | G | T | 0.01409 | 0.66763 | 0.0328liver cancer rs613872 G T 0.05991 0.4333 0.07646 Choleliths rs613872 G T -0.0118 0.29807 0.01131 Acute pancreatitis rs613872 G T -0.0182 0.49121 0.02646 Chronic pancreatitis rs613872 G T 0.03404 0.33987 0.03566 Pancreatic cancer rs613872 G T -0.0445 0.41974 0.05516 Ulcerative colitis rs613872 G T 0.03496 0.23268 0.02929 Crohn's disease rs613872 G T -0.0123 0.80833 0.0505 Irrtable bowel syndrome rs613872 G T -0.0211 0.33538 0.02192 Acute appendicitis rs613872 G T 0.00981 0.43619 0.0126 Colon cancer rs613872 G T -0.0285 0.40664 0.03433 Rectal cancer rs613872 G T -0.0934 0.03277 0.04377 |
| Gastroesophageal reflux | rs62097985 | C | T | -0.0123 | 0.22153 | 0.01004 | Esophageal carcinoma | rs62097985 | C | T | 0.06153 | 0.34276 | 0.06486 | Acute gastritis | rs62097985 | C | T | 0.0217 | 0.49289 | 0.03164 | Chronic gastritis | rs62097985 | C | T | -0.0275 | 0.08612 | 0.01603 | Gastroduodenal ulcer | rs62097985 | C | T | -0.0012 | 0.94085 | 0.01642 | Gastric cancer | rs62097985 | C | T | -0.0775 | 0.06247 | 0.04162 | Fatty liver | rs62097985 | C | T | -0.0628 | 0.0596 | 0.03333 | Hepatic fibrosis | rs62097985 | C | T | 0.29694 | 0.01384 | 0.12064 | Cirrhosis | rs62097985 | C | T | 0.01327 | 0.58918 | 0.02457liver cancer rs62097985 C T 0.02122 0.71062 0.0572 Choleliths rs62097985 C T -0.03 0.00039 0.00846 Acute pancreatitis rs62097985 C T 0.01053 0.59503 0.01981 Chronic pancreatitis rs62097985 C T 0.00908 0.73393 0.02672 Pancreatic cancer rs62097985 C T -0.0158 0.70089 0.04122 Ulcerative colitis rs62097985 C T 0.02493 0.25554 0.02193 Crohn's disease rs62097985 C T -0.0017 0.96398 0.0378 Irrtable bowel syndrome rs62097985 C T -0.0234 0.15255 0.01639 Acute appendicitis rs62097985 C T -0.0048 0.6135 0.00943 Colon cancer rs62097985 C T 0.03167 0.2182 0.02572 Rectal cancer rs62097985 C T 0.01027 0.75245 0.03256 |
| Gastroesophageal reflux | rs62103084 | T | C | 0.00614 | 0.68933 | 0.01537 | Esophageal carcinoma | rs62103084 | T | C | 0.04721 | 0.63456 | 0.09932 | Acute gastritis | rs62103084 | T | C | 0.02882 | 0.55185 | 0.04843 | Chronic gastritis | rs62103084 | T | C | 0.02495 | 0.30916 | 0.02453 | Gastroduodenal ulcer | rs62103084 | T | C | 0.00404 | 0.87227 | 0.02513 | Gastric cancer | rs62103084 | T | C | 0.0335 | 0.6012 | 0.0641 | Fatty liver | rs62103084 | T | C | 0.01328 | 0.79442 | 0.05096 | Hepatic fibrosis | rs62103084 | T | C | 0.09881 | 0.61092 | 0.19422 | Cirrhosis | rs62103084 | T | C | -0.0124 | 0.74059 | 0.0376liver cancer rs62103084 T C -0.1222 0.16097 0.08719 Choleliths rs62103084 T C 0.01132 0.38287 0.01297 Acute pancreatitis rs62103084 T C -0.0246 0.41638 0.0303 Chronic pancreatitis rs62103084 T C 0.02227 0.58517 0.04081 Pancreatic cancer rs62103084 T C -0.0457 0.47086 0.06333 Ulcerative colitis rs62103084 T C 0.01653 0.62165 0.0335 Crohn's disease rs62103084 T C 0.01361 0.81374 0.05778 Irrtable bowel syndrome rs62103084 T C -0.0088 0.72532 0.02504 Acute appendicitis rs62103084 T C -0.0046 0.74774 0.01444 Colon cancer rs62103084 T C 0.05954 0.13086 0.03941 Rectal cancer rs62103084 T C -0.0076 0.87971 0.04998 |
| Gastroesophageal reflux | rs62157915 | T | C | 0.03477 | 0.14115 | 0.02363 | Esophageal carcinoma | rs62157915 | T | A | 1.7692 | 0.13553 | 1.18528 | Acute gastritis | rs62157915 | T | C | 0.08659 | 0.25167 | 0.07554 | Chronic gastritis | rs62157915 | T | A | 0.21554 | 0.4428 | 0.28085 | Gastroduodenal ulcer | rs62157915 | T | A | 0.07919 | 0.78004 | 0.28356 | Gastric cancer | rs62157915 | T | C | 0.04669 | 0.63438 | 0.09818 | Fatty liver | rs62157915 | T | C | 0.12581 | 0.11194 | 0.07915 | Hepatic fibrosis | rs62157915 | T | A | 0.37066 | 0.86588 | 2.19463 | Cirrhosis | rs62157915 | T | C | -0.0149 | 0.79685 | 0.05807liver cancer rs62157915 T A -0.074 0.93979 0.97949 Choleliths rs62157915 T C 0.03078 0.12396 0.02001 Acute pancreatitis rs62157915 T C 0.05103 0.27508 0.04676 Chronic pancreatitis rs62157915 T C 0.05398 0.39463 0.06341 Pancreatic cancer rs62157915 T A 0.03537 0.96161 0.73493 Ulcerative colitis rs62157915 T A -0.6896 0.06991 0.38045 Crohn's disease rs62157915 T A -0.0858 0.90046 0.68611 Irrtable bowel syndrome rs62157915 T C -0.0024 0.95061 0.03883 Acute appendicitis rs62157915 T A -0.2874 0.07759 0.16287 Colon cancer rs62157915 T C -0.027 0.65095 0.0597 Rectal cancer rs62157915 T C 0.01869 0.80547 0.07588 |
| Gastroesophageal reflux | rs62183776 | T | C | -0.0315 | 0.03173 | 0.01468 | Esophageal carcinoma | rs62183776 | T | C | -0.0589 | 0.5372 | 0.09545 | Acute gastritis | rs62183776 | T | C | -0.0117 | 0.80155 | 0.04657 | Chronic gastritis | rs62183776 | T | C | -0.0274 | 0.24189 | 0.02338 | Gastroduodenal ulcer | rs62183776 | T | C | -0.001 | 0.96752 | 0.02403 | Gastric cancer | rs62183776 | T | C | -0.0492 | 0.42097 | 0.06115 | Fatty liver | rs62183776 | T | C | 0.01792 | 0.71402 | 0.04889 | Hepatic fibrosis | rs62183776 | T | C | 0.07845 | 0.67569 | 0.18751 | Cirrhosis | rs62183776 | T | C | 0.06912 | 0.05563 | 0.03611liver cancer rs62183776 T C -0.0393 0.64147 0.08438 Choleliths rs62183776 T C 0.00673 0.58785 0.01241 Acute pancreatitis rs62183776 T C -0.0049 0.86597 0.02909 Chronic pancreatitis rs62183776 T C -0.0327 0.40466 0.03928 Pancreatic cancer rs62183776 T C -0.0462 0.44871 0.06096 Ulcerative colitis rs62183776 T C 0.02522 0.43438 0.03227 Crohn's disease rs62183776 T C 0.07591 0.17189 0.05556 Irrtable bowel syndrome rs62183776 T C 0.00296 0.90171 0.02401 Acute appendicitis rs62183776 T C -0.0129 0.35333 0.01387 Colon cancer rs62183776 T C -0.0308 0.41254 0.03764 Rectal cancer rs62183776 T C -0.0611 0.20126 0.04783 |
| Gastroesophageal reflux | rs62184480 | T | C | 0.02534 | 0.02254 | 0.01111 | Esophageal carcinoma | rs62184480 | T | C | 0.00277 | 0.96935 | 0.07196 | Acute gastritis | rs62184480 | T | C | 0.05867 | 0.09443 | 0.03508 | Chronic gastritis | rs62184480 | T | C | 0.02183 | 0.21897 | 0.01776 | Gastroduodenal ulcer | rs62184480 | T | C | 0.03431 | 0.05905 | 0.01817 | Gastric cancer | rs62184480 | T | C | -0.0019 | 0.96776 | 0.04618 | Fatty liver | rs62184480 | T | C | 0.07323 | 0.04664 | 0.03681 | Hepatic fibrosis | rs62184480 | T | C | 0.25098 | 0.0745 | 0.14072 | Cirrhosis | rs62184480 | T | C | 0.03232 | 0.23339 | 0.02712liver cancer rs62184480 T C -0.032 0.61404 0.0634 Choleliths rs62184480 T C 0.00984 0.29303 0.00936 Acute pancreatitis rs62184480 T C 0.0449 0.03995 0.02186 Chronic pancreatitis rs62184480 T C 0.0201 0.4949 0.02945 Pancreatic cancer rs62184480 T C 0.00407 0.9289 0.04564 Ulcerative colitis rs62184480 T C 0.00594 0.80619 0.02423 Crohn's disease rs62184480 T C -0.0241 0.56461 0.04186 Irrtable bowel syndrome rs62184480 T C 0.01827 0.31383 0.01814 Acute appendicitis rs62184480 T C 0.00035 0.97331 0.01041 Colon cancer rs62184480 T C -0.0449 0.1143 0.02846 Rectal cancer rs62184480 T C 0.01143 0.75177 0.03614 |
| Gastroesophageal reflux | rs622169 | C | T | -0.0008 | 0.93293 | 0.00999 | Esophageal carcinoma | rs622169 | C | T | -0.0968 | 0.13546 | 0.06485 | Acute gastritis | rs622169 | C | T | -0.01 | 0.75026 | 0.03154 | Chronic gastritis | rs622169 | C | T | -0.0144 | 0.36819 | 0.01599 | Gastroduodenal ulcer | rs622169 | C | T | -0.0238 | 0.14584 | 0.01636 | Gastric cancer | rs622169 | C | T | 0.00793 | 0.84922 | 0.04169 | Fatty liver | rs622169 | C | T | 0.04188 | 0.20616 | 0.03313 | Hepatic fibrosis | rs622169 | C | T | -0.1892 | 0.13394 | 0.12627 | Cirrhosis | rs622169 | C | T | 0.03275 | 0.17987 | 0.02442liver cancer rs622169 C T 0.02241 0.69575 0.05731 Choleliths rs622169 C T -0.0028 0.73838 0.00846 Acute pancreatitis rs622169 C T 0.02766 0.15987 0.01968 Chronic pancreatitis rs622169 C T -0.0348 0.18962 0.02651 Pancreatic cancer rs622169 C T -0.0278 0.50092 0.04125 Ulcerative colitis rs622169 C T -0.0069 0.75291 0.02179 Crohn's disease rs622169 C T -0.0514 0.17103 0.03752 Irrtable bowel syndrome rs622169 C T -0.0045 0.78412 0.01632 Acute appendicitis rs622169 C T 0.00993 0.28964 0.00938 Colon cancer rs622169 C T 0.04858 0.05878 0.02571 Rectal cancer rs622169 C T -0.0465 0.15382 0.03263 |
| Gastroesophageal reflux | rs62439690 | G | A | 0.00347 | 0.77553 | 0.01216 | Esophageal carcinoma | rs62439690 | G | A | -0.0453 | 0.56617 | 0.079 | Acute gastritis | rs62439690 | G | A | 0.01693 | 0.65882 | 0.03834 | Chronic gastritis | rs62439690 | G | A | -0.0072 | 0.71072 | 0.01941 | Gastroduodenal ulcer | rs62439690 | G | A | 0.0168 | 0.39843 | 0.01989 | Gastric cancer | rs62439690 | G | A | 0.01875 | 0.7116 | 0.05071 | Fatty liver | rs62439690 | G | A | -0.0264 | 0.51388 | 0.04037 | Hepatic fibrosis | rs62439690 | G | A | 0.31039 | 0.04426 | 0.1543 | Cirrhosis | rs62439690 | G | A | 0.03741 | 0.20872 | 0.02976liver cancer rs62439690 G A 0.16012 0.02102 0.06939 Choleliths rs62439690 G A -0.0137 0.1808 0.01026 Acute pancreatitis rs62439690 G A -0.0608 0.01118 0.02398 Chronic pancreatitis rs62439690 G A -0.0364 0.26097 0.03238 Pancreatic cancer rs62439690 G A -0.0182 0.71667 0.05012 Ulcerative colitis rs62439690 G A -0.0568 0.03228 0.02653 Crohn's disease rs62439690 G A 0.02884 0.52867 0.04578 Irrtable bowel syndrome rs62439690 G A 0.01082 0.58573 0.01986 Acute appendicitis rs62439690 G A 0.00251 0.8259 0.01142 Colon cancer rs62439690 G A -0.0546 0.07998 0.0312 Rectal cancer rs62439690 G A -0.0134 0.73613 0.03966 |
| Gastroesophageal reflux | rs62444881 | C | T | 0.03641 | 0.00089 | 0.01096 | Esophageal carcinoma | rs62444881 | C | T | 0.04011 | 0.57005 | 0.07062 | Acute gastritis | rs62444881 | C | T | 0.01858 | 0.58793 | 0.03429 | Chronic gastritis | rs62444881 | C | T | 0.00074 | 0.96635 | 0.01745 | Gastroduodenal ulcer | rs62444881 | C | T | 0.00857 | 0.63032 | 0.0178 | Gastric cancer | rs62444881 | C | T | 0.00607 | 0.89366 | 0.04539 | Fatty liver | rs62444881 | C | T | 0.02651 | 0.46389 | 0.03619 | Hepatic fibrosis | rs62444881 | C | T | -0.0009 | 0.99492 | 0.13759 | Cirrhosis | rs62444881 | C | T | -0.0501 | 0.0595 | 0.02661liver cancer rs62444881 C T 0.00647 0.91696 0.06209 Choleliths rs62444881 C T 0.01584 0.08546 0.00921 Acute pancreatitis rs62444881 C T 0.0434 0.04293 0.02144 Chronic pancreatitis rs62444881 C T 0.04853 0.0931 0.0289 Pancreatic cancer rs62444881 C T -0.0123 0.78285 0.04474 Ulcerative colitis rs62444881 C T 0.00462 0.84556 0.02373 Crohn's disease rs62444881 C T 0.11705 0.0047 0.0414 Irrtable bowel syndrome rs62444881 C T 0.01873 0.29255 0.0178 Acute appendicitis rs62444881 C T 0.00597 0.55877 0.01021 Colon cancer rs62444881 C T 0.0015 0.95721 0.02792 Rectal cancer rs62444881 C T -0.0205 0.56393 0.03547 |
| Gastroesophageal reflux | rs6493265 | C | T | 0.00216 | 0.83457 | 0.01036 | Esophageal carcinoma | rs6493265 | C | T | 0.02695 | 0.68836 | 0.06719 | Acute gastritis | rs6493265 | C | T | -0.0352 | 0.28048 | 0.03265 | Chronic gastritis | rs6493265 | C | T | -0.0033 | 0.841 | 0.01658 | Gastroduodenal ulcer | rs6493265 | C | T | -0.0212 | 0.21055 | 0.01693 | Gastric cancer | rs6493265 | C | T | -0.0463 | 0.28164 | 0.04304 | Fatty liver | rs6493265 | C | T | 0.07848 | 0.02248 | 0.03439 | Hepatic fibrosis | rs6493265 | C | T | -0.1423 | 0.27889 | 0.13141 | Cirrhosis | rs6493265 | C | T | 0.03606 | 0.15456 | 0.02533liver cancer rs6493265 C T 0.01608 0.78609 0.05924 Choleliths rs6493265 C T -0.0035 0.68796 0.00875 Acute pancreatitis rs6493265 C T 0.00715 0.72602 0.0204 Chronic pancreatitis rs6493265 C T 0.0199 0.46997 0.02755 Pancreatic cancer rs6493265 C T 0.00262 0.95108 0.04268 Ulcerative colitis rs6493265 C T -0.0102 0.65295 0.02262 Crohn's disease rs6493265 C T -0.0425 0.27567 0.03903 Irrtable bowel syndrome rs6493265 C T -0.0136 0.42176 0.01693 Acute appendicitis rs6493265 C T 0.00703 0.46973 0.00973 Colon cancer rs6493265 C T -0.0025 0.92531 0.02661 Rectal cancer rs6493265 C T -0.0213 0.52862 0.03381 |
| Gastroesophageal reflux | rs6513959 | A | G | 0.01166 | 0.24321 | 0.01 | Esophageal carcinoma | rs6513959 | A | G | 0.09637 | 0.13548 | 0.06455 | Acute gastritis | rs6513959 | A | G | 0.09363 | 0.00304 | 0.03159 | Chronic gastritis | rs6513959 | A | G | 0.01128 | 0.48026 | 0.01598 | Gastroduodenal ulcer | rs6513959 | A | G | 0.0098 | 0.54826 | 0.01633 | Gastric cancer | rs6513959 | A | G | -0.061 | 0.14202 | 0.04154 | Fatty liver | rs6513959 | A | G | 0.01323 | 0.69045 | 0.03322 | Hepatic fibrosis | rs6513959 | A | G | -0.0193 | 0.87951 | 0.12713 | Cirrhosis | rs6513959 | A | G | -0.0043 | 0.86193 | 0.02444liver cancer rs6513959 A G 0.02197 0.70011 0.05703 Choleliths rs6513959 A G 0.01437 0.0889 0.00845 Acute pancreatitis rs6513959 A G 0.00928 0.63767 0.01971 Chronic pancreatitis rs6513959 A G -0.0014 0.9585 0.02657 Pancreatic cancer rs6513959 A G -0.0129 0.75366 0.04112 Ulcerative colitis rs6513959 A G -0.0011 0.96148 0.02181 Crohn's disease rs6513959 A G -0.0678 0.0719 0.03769 Irrtable bowel syndrome rs6513959 A G 0.0256 0.11731 0.01634 Acute appendicitis rs6513959 A G 0.02386 0.01104 0.00939 Colon cancer rs6513959 A G 0.00957 0.7088 0.02564 Rectal cancer rs6513959 A G -0.0522 0.10815 0.03252 |
| Gastroesophageal reflux | rs6557171 | C | T | -0.0037 | 0.7196 | 0.01041 | Esophageal carcinoma | rs6557171 | C | T | -0.0394 | 0.56036 | 0.06773 | Acute gastritis | rs6557171 | C | T | 0.01683 | 0.60845 | 0.03286 | Chronic gastritis | rs6557171 | C | T | -0.0284 | 0.08817 | 0.01666 | Gastroduodenal ulcer | rs6557171 | C | T | -0.0394 | 0.02074 | 0.01703 | Gastric cancer | rs6557171 | C | T | 0.05591 | 0.19786 | 0.04342 | Fatty liver | rs6557171 | C | T | 0.02821 | 0.4139 | 0.03453 | Hepatic fibrosis | rs6557171 | C | T | -0.2964 | 0.02424 | 0.13154 | Cirrhosis | rs6557171 | C | T | -0.0332 | 0.19227 | 0.02547liver cancer rs6557171 C T -0.0645 0.27855 0.05956 Choleliths rs6557171 C T -0.0081 0.3569 0.00881 Acute pancreatitis rs6557171 C T -0.0073 0.72228 0.02052 Chronic pancreatitis rs6557171 C T -0.0445 0.10744 0.02766 Pancreatic cancer rs6557171 C T -0.0388 0.36627 0.04294 Ulcerative colitis rs6557171 C T -0.0185 0.41536 0.02269 Crohn's disease rs6557171 C T -0.0502 0.19917 0.03912 Irrtable bowel syndrome rs6557171 C T -0.0425 0.0124 0.017 Acute appendicitis rs6557171 C T -0.0073 0.45557 0.00976 Colon cancer rs6557171 C T -0.0129 0.63126 0.02678 Rectal cancer rs6557171 C T 0.02175 0.52324 0.03407 |
| Gastroesophageal reflux | rs663234 | G | C | 0.0179 | 0.07593 | 0.01009 | Esophageal carcinoma | rs663234 | G | C | -0.0927 | 0.15511 | 0.06518 | Acute gastritis | rs663234 | G | C | 0.06578 | 0.0386 | 0.0318 | Chronic gastritis | rs663234 | G | C | 0.01735 | 0.28185 | 0.01612 | Gastroduodenal ulcer | rs663234 | G | C | 0.03847 | 0.01956 | 0.01648 | Gastric cancer | rs663234 | G | C | -0.0633 | 0.13097 | 0.04194 | Fatty liver | rs663234 | G | C | 0.0078 | 0.81577 | 0.03346 | Hepatic fibrosis | rs663234 | G | C | 0.26753 | 0.03542 | 0.12718 | Cirrhosis | rs663234 | G | C | 0.01331 | 0.589 | 0.02463liver cancer rs663234 G C -0.0296 0.60669 0.05746 Choleliths rs663234 G C -0.0083 0.33011 0.00852 Acute pancreatitis rs663234 G C -0.0029 0.88567 0.01988 Chronic pancreatitis rs663234 G C 0.00785 0.76982 0.02681 Pancreatic cancer rs663234 G C -0.0166 0.6884 0.04143 Ulcerative colitis rs663234 G C -0.0035 0.87407 0.02197 Crohn's disease rs663234 G C 0.03025 0.42542 0.03795 Irrtable bowel syndrome rs663234 G C 0.00122 0.94114 0.01646 Acute appendicitis rs663234 G C -0.0095 0.31351 0.00946 Colon cancer rs663234 G C -0.0317 0.22169 0.02592 Rectal cancer rs663234 G C -0.0009 0.97779 0.03288 |
| Gastroesophageal reflux | rs6731373 | G | A | -0.014 | 0.23112 | 0.01172 | Esophageal carcinoma | rs6731373 | G | A | 0.11147 | 0.13954 | 0.07544 | Acute gastritis | rs6731373 | G | A | -0.0043 | 0.90786 | 0.03711 | Chronic gastritis | rs6731373 | G | A | 0.04259 | 0.02302 | 0.01874 | Gastroduodenal ulcer | rs6731373 | G | A | 0.02392 | 0.21308 | 0.01921 | Gastric cancer | rs6731373 | G | A | -0.0595 | 0.22068 | 0.04861 | Fatty liver | rs6731373 | G | A | 0.02374 | 0.54259 | 0.03898 | Hepatic fibrosis | rs6731373 | G | A | 0.03023 | 0.84017 | 0.14987 | Cirrhosis | rs6731373 | G | A | -0.0123 | 0.66944 | 0.02878liver cancer rs6731373 G A 0.07381 0.26848 0.0667 Choleliths rs6731373 G A -0.0143 0.14858 0.00991 Acute pancreatitis rs6731373 G A -0.0037 0.87498 0.0232 Chronic pancreatitis rs6731373 G A 0.0048 0.87823 0.03132 Pancreatic cancer rs6731373 G A 0.02291 0.63578 0.04837 Ulcerative colitis rs6731373 G A -0.0259 0.31242 0.02566 Crohn's disease rs6731373 G A 0.04444 0.31444 0.04418 Irrtable bowel syndrome rs6731373 G A -0.0288 0.1331 0.01917 Acute appendicitis rs6731373 G A -0.0066 0.55298 0.01105 Colon cancer rs6731373 G A -0.0318 0.28821 0.02996 Rectal cancer rs6731373 G A -0.044 0.24706 0.03799 |
| Gastroesophageal reflux | rs6731967 | C | G | -0.0013 | 0.90421 | 0.01096 | Esophageal carcinoma | rs6731967 | C | G | 0.12454 | 0.07935 | 0.07098 | Acute gastritis | rs6731967 | C | G | 0.00448 | 0.89717 | 0.03466 | Chronic gastritis | rs6731967 | C | G | 0.01901 | 0.27824 | 0.01753 | Gastroduodenal ulcer | rs6731967 | C | G | 0.00023 | 0.98969 | 0.01793 | Gastric cancer | rs6731967 | C | G | -0.0802 | 0.07814 | 0.04551 | Fatty liver | rs6731967 | C | G | -0.0014 | 0.96889 | 0.03636 | Hepatic fibrosis | rs6731967 | C | G | 0.17475 | 0.21001 | 0.13941 | Cirrhosis | rs6731967 | C | G | 0.0273 | 0.30853 | 0.02681liver cancer rs6731967 C G 0.05661 0.36512 0.06251 Choleliths rs6731967 C G 0.00262 0.77698 0.00925 Acute pancreatitis rs6731967 C G 7.3E-05 0.99729 0.0216 Chronic pancreatitis rs6731967 C G -0.0114 0.69432 0.02911 Pancreatic cancer rs6731967 C G 0.08808 0.05108 0.04515 Ulcerative colitis rs6731967 C G -0.0033 0.89046 0.02391 Crohn's disease rs6731967 C G 0.00699 0.86541 0.04126 Irrtable bowel syndrome rs6731967 C G -0.015 0.40199 0.0179 Acute appendicitis rs6731967 C G -0.0122 0.23721 0.01028 Colon cancer rs6731967 C G 0.03034 0.27989 0.02808 Rectal cancer rs6731967 C G 0.0362 0.30991 0.03565 |
| Gastroesophageal reflux | rs67885444 | T | C | -0.0071 | 0.5956 | 0.01331 | Esophageal carcinoma | rs67885444 | T | C | 0.14289 | 0.09642 | 0.08595 | Acute gastritis | rs67885444 | T | C | -0.01 | 0.81237 | 0.04196 | Chronic gastritis | rs67885444 | T | C | 0.03868 | 0.06881 | 0.02126 | Gastroduodenal ulcer | rs67885444 | T | C | -0.0031 | 0.8859 | 0.02171 | Gastric cancer | rs67885444 | T | C | -0.0348 | 0.53105 | 0.05555 | Fatty liver | rs67885444 | T | C | 0.0043 | 0.92242 | 0.0441 | Hepatic fibrosis | rs67885444 | T | C | 0.01722 | 0.91816 | 0.16761 | Cirrhosis | rs67885444 | T | C | -0.0063 | 0.84521 | 0.03244liver cancer rs67885444 T C 0.04933 0.5145 0.07567 Choleliths rs67885444 T C 0.00363 0.74693 0.01126 Acute pancreatitis rs67885444 T C 0.01606 0.5401 0.02621 Chronic pancreatitis rs67885444 T C 0.04796 0.17333 0.03523 Pancreatic cancer rs67885444 T C 0.01735 0.75145 0.05477 Ulcerative colitis rs67885444 T C -0.0411 0.15668 0.02901 Crohn's disease rs67885444 T C 0.00638 0.89864 0.0501 Irrtable bowel syndrome rs67885444 T C -0.04 0.0652 0.02171 Acute appendicitis rs67885444 T C 0.02981 0.01686 0.01247 Colon cancer rs67885444 T C 0.01785 0.60232 0.03426 Rectal cancer rs67885444 T C -0.0426 0.32577 0.04337 |
| Gastroesophageal reflux | rs67890737 | C | A | -0.0168 | 0.11181 | 0.01054 | Esophageal carcinoma | rs67890737 | C | A | -0.0349 | 0.60933 | 0.06821 | Acute gastritis | rs67890737 | C | A | -0.0591 | 0.07611 | 0.0333 | Chronic gastritis | rs67890737 | C | A | 0.02584 | 0.1247 | 0.01683 | Gastroduodenal ulcer | rs67890737 | C | A | -0.007 | 0.68356 | 0.01726 | Gastric cancer | rs67890737 | C | A | -0.0071 | 0.87037 | 0.04373 | Fatty liver | rs67890737 | C | A | 0.0375 | 0.28453 | 0.03504 | Hepatic fibrosis | rs67890737 | C | A | 0.15523 | 0.2462 | 0.13386 | Cirrhosis | rs67890737 | C | A | 0.01534 | 0.55262 | 0.02583liver cancer rs67890737 C A -0.0236 0.69602 0.06036 Choleliths rs67890737 C A -0.0114 0.19998 0.00891 Acute pancreatitis rs67890737 C A 0.01023 0.62272 0.0208 Chronic pancreatitis rs67890737 C A -0.0035 0.89943 0.02805 Pancreatic cancer rs67890737 C A 0.06734 0.12181 0.04352 Ulcerative colitis rs67890737 C A 0.00852 0.71109 0.02299 Crohn's disease rs67890737 C A 0.04088 0.30305 0.03969 Irrtable bowel syndrome rs67890737 C A -0.0107 0.53495 0.01723 Acute appendicitis rs67890737 C A -0.0086 0.38783 0.00991 Colon cancer rs67890737 C A 0.05753 0.03341 0.02704 Rectal cancer rs67890737 C A 0.04856 0.15777 0.03438 |
| Gastroesophageal reflux | rs6803651 | G | T | -0.0096 | 0.3392 | 0.01002 | Esophageal carcinoma | rs6803651 | G | T | -0.0517 | 0.42587 | 0.06496 | Acute gastritis | rs6803651 | G | T | 0.0118 | 0.70881 | 0.03159 | Chronic gastritis | rs6803651 | G | T | -0.0437 | 0.00607 | 0.01593 | Gastroduodenal ulcer | rs6803651 | G | T | -0.0104 | 0.52597 | 0.01638 | Gastric cancer | rs6803651 | G | T | -0.0693 | 0.09667 | 0.0417 | Fatty liver | rs6803651 | G | T | -0.0028 | 0.93207 | 0.03324 | Hepatic fibrosis | rs6803651 | G | T | -0.0147 | 0.90783 | 0.1273 | Cirrhosis | rs6803651 | G | T | 0.01676 | 0.49441 | 0.02453liver cancer rs6803651 G T 0.07388 0.19717 0.05729 Choleliths rs6803651 G T -0.0027 0.75162 0.00846 Acute pancreatitis rs6803651 G T 0.00306 0.87702 0.01976 Chronic pancreatitis rs6803651 G T -0.0067 0.80014 0.02665 Pancreatic cancer rs6803651 G T 0.01196 0.77214 0.04129 Ulcerative colitis rs6803651 G T -0.0306 0.16247 0.0219 Crohn's disease rs6803651 G T -0.0073 0.84776 0.03781 Irrtable bowel syndrome rs6803651 G T 0.00359 0.82636 0.01638 Acute appendicitis rs6803651 G T 0.00657 0.48538 0.00942 Colon cancer rs6803651 G T -0.0265 0.30185 0.02569 Rectal cancer rs6803651 G T -0.0096 0.76812 0.03262 |
| Gastroesophageal reflux | rs6805241 | C | T | 0.00381 | 0.73894 | 0.01145 | Esophageal carcinoma | rs6805241 | C | T | -0.0636 | 0.39155 | 0.07424 | Acute gastritis | rs6805241 | C | T | -0.0117 | 0.74675 | 0.03626 | Chronic gastritis | rs6805241 | C | T | 0.04354 | 0.01736 | 0.0183 | Gastroduodenal ulcer | rs6805241 | C | T | 0.01869 | 0.31939 | 0.01877 | Gastric cancer | rs6805241 | C | T | 0.05106 | 0.2841 | 0.04767 | Fatty liver | rs6805241 | C | T | -0.0589 | 0.12155 | 0.03802 | Hepatic fibrosis | rs6805241 | C | T | 0.10163 | 0.4853 | 0.14565 | Cirrhosis | rs6805241 | C | T | 0.03023 | 0.28152 | 0.02807liver cancer rs6805241 C T 0.01234 0.85048 0.06546 Choleliths rs6805241 C T 0.0111 0.25166 0.00968 Acute pancreatitis rs6805241 C T 0.00567 0.80225 0.02264 Chronic pancreatitis rs6805241 C T -0.0314 0.30249 0.0305 Pancreatic cancer rs6805241 C T -0.0538 0.25481 0.04727 Ulcerative colitis rs6805241 C T -0.0042 0.86527 0.02502 Crohn's disease rs6805241 C T 0.03298 0.44474 0.04316 Irrtable bowel syndrome rs6805241 C T -0.0049 0.79439 0.0187 Acute appendicitis rs6805241 C T 0.01625 0.13115 0.01076 Colon cancer rs6805241 C T 0.05384 0.06712 0.02941 Rectal cancer rs6805241 C T -0.0172 0.64435 0.03733 |
| Gastroesophageal reflux | rs6867851 | C | G | -0.0089 | 0.39258 | 0.01037 | Esophageal carcinoma | rs6867851 | C | G | -0.049 | 0.46376 | 0.06683 | Acute gastritis | rs6867851 | C | G | -0.0085 | 0.7947 | 0.03276 | Chronic gastritis | rs6867851 | C | G | -0.0082 | 0.62204 | 0.01656 | Gastroduodenal ulcer | rs6867851 | C | G | -0.0192 | 0.25652 | 0.01696 | Gastric cancer | rs6867851 | C | G | 0.02422 | 0.57188 | 0.04285 | Fatty liver | rs6867851 | C | G | -0.0372 | 0.27995 | 0.03447 | Hepatic fibrosis | rs6867851 | C | G | 0.00977 | 0.94099 | 0.13197 | Cirrhosis | rs6867851 | C | G | -0.0376 | 0.13867 | 0.02539liver cancer rs6867851 C G -0.1232 0.03718 0.05911 Choleliths rs6867851 C G -0.0006 0.94989 0.00876 Acute pancreatitis rs6867851 C G 0.04797 0.0191 0.02047 Chronic pancreatitis rs6867851 C G 0.02877 0.29784 0.02764 Pancreatic cancer rs6867851 C G -0.0157 0.71358 0.04265 Ulcerative colitis rs6867851 C G -0.0018 0.93728 0.02268 Crohn's disease rs6867851 C G 0.01311 0.73757 0.03912 Irrtable bowel syndrome rs6867851 C G -0.0424 0.01238 0.01694 Acute appendicitis rs6867851 C G -0.0097 0.3212 0.00975 Colon cancer rs6867851 C G 0.00147 0.95565 0.02651 Rectal cancer rs6867851 C G -0.0109 0.74545 0.03362 |
| Gastroesophageal reflux | rs6938002 | G | A | 0.02089 | 0.03361 | 0.00983 | Esophageal carcinoma | rs6938002 | G | A | 0.02331 | 0.71484 | 0.06379 | Acute gastritis | rs6938002 | G | A | -0.0475 | 0.12632 | 0.03106 | Chronic gastritis | rs6938002 | G | A | 0.00756 | 0.63069 | 0.01572 | Gastroduodenal ulcer | rs6938002 | G | A | -0.0118 | 0.46296 | 0.01607 | Gastric cancer | rs6938002 | G | A | 0.06461 | 0.11422 | 0.04091 | Fatty liver | rs6938002 | G | A | 0.00966 | 0.76731 | 0.03263 | Hepatic fibrosis | rs6938002 | G | A | 0.09057 | 0.46829 | 0.12487 | Cirrhosis | rs6938002 | G | A | 0.01485 | 0.53644 | 0.02403liver cancer rs6938002 G A 0.06877 0.22205 0.05632 Choleliths rs6938002 G A -0.0113 0.17324 0.00831 Acute pancreatitis rs6938002 G A 0.0103 0.59512 0.01939 Chronic pancreatitis rs6938002 G A 0.0193 0.45991 0.02612 Pancreatic cancer rs6938002 G A 0.04518 0.26433 0.04047 Ulcerative colitis rs6938002 G A 0.00216 0.91969 0.02142 Crohn's disease rs6938002 G A -0.0646 0.0803 0.03696 Irrtable bowel syndrome rs6938002 G A -0.0092 0.5674 0.01606 Acute appendicitis rs6938002 G A -0.0023 0.80077 0.00923 Colon cancer rs6938002 G A -0.0363 0.1507 0.02525 Rectal cancer rs6938002 G A 0.01526 0.63406 0.03205 |
| Gastroesophageal reflux | rs6959891 | G | A | -0.0229 | 0.04519 | 0.01141 | Esophageal carcinoma | rs6959891 | G | A | 0.01314 | 0.85863 | 0.07379 | Acute gastritis | rs6959891 | G | A | 0.03569 | 0.32111 | 0.03597 | Chronic gastritis | rs6959891 | G | A | 0.01863 | 0.30606 | 0.01821 | Gastroduodenal ulcer | rs6959891 | G | A | -0.0332 | 0.07519 | 0.01864 | Gastric cancer | rs6959891 | G | A | -0.0024 | 0.96028 | 0.04743 | Fatty liver | rs6959891 | G | A | -0.0052 | 0.89075 | 0.03789 | Hepatic fibrosis | rs6959891 | G | A | -0.0145 | 0.91995 | 0.1442 | Cirrhosis | rs6959891 | G | A | -0.012 | 0.66695 | 0.02792liver cancer rs6959891 G A -0.055 0.39855 0.06512 Choleliths rs6959891 G A -0.0065 0.50169 0.00965 Acute pancreatitis rs6959891 G A -0.0116 0.60725 0.02251 Chronic pancreatitis rs6959891 G A -0.0102 0.73789 0.03036 Pancreatic cancer rs6959891 G A 0.02993 0.52266 0.04682 Ulcerative colitis rs6959891 G A 0.0159 0.52396 0.02495 Crohn's disease rs6959891 G A -0.0789 0.06679 0.04302 Irrtable bowel syndrome rs6959891 G A 0.03587 0.05469 0.01867 Acute appendicitis rs6959891 G A -0.0019 0.85847 0.01073 Colon cancer rs6959891 G A 0.00232 0.93665 0.0292 Rectal cancer rs6959891 G A -0.052 0.16011 0.03702 |
| Gastroesophageal reflux | rs7012546 | C | T | 0.0111 | 0.2634 | 0.00992 | Esophageal carcinoma | rs7012546 | C | T | -0.0448 | 0.48472 | 0.06414 | Acute gastritis | rs7012546 | C | T | 0.03452 | 0.26976 | 0.03128 | Chronic gastritis | rs7012546 | C | T | 0.00186 | 0.90661 | 0.01585 | Gastroduodenal ulcer | rs7012546 | C | T | 0.03516 | 0.0303 | 0.01623 | Gastric cancer | rs7012546 | C | T | -0.0476 | 0.24829 | 0.04126 | Fatty liver | rs7012546 | C | T | 0.04977 | 0.13092 | 0.03295 | Hepatic fibrosis | rs7012546 | C | T | -0.2183 | 0.08317 | 0.12601 | Cirrhosis | rs7012546 | C | T | -0.0178 | 0.46466 | 0.02429liver cancer rs7012546 C T -0.0148 0.79369 0.05672 Choleliths rs7012546 C T 0.00182 0.82827 0.00838 Acute pancreatitis rs7012546 C T 0.008 0.68265 0.01956 Chronic pancreatitis rs7012546 C T -0.018 0.49525 0.02639 Pancreatic cancer rs7012546 C T 0.04452 0.27599 0.04087 Ulcerative colitis rs7012546 C T 0.04169 0.05458 0.02169 Crohn's disease rs7012546 C T -0.0684 0.06741 0.03741 Irrtable bowel syndrome rs7012546 C T 0.02992 0.06491 0.01621 Acute appendicitis rs7012546 C T 0.00814 0.38271 0.00933 Colon cancer rs7012546 C T -0.0374 0.1415 0.02543 Rectal cancer rs7012546 C T -0.0273 0.39788 0.0323 |
| Gastroesophageal reflux | rs7016302 | G | C | 0.01143 | 0.37286 | 0.01283 | Esophageal carcinoma | rs7016302 | G | C | -0.0964 | 0.24723 | 0.0833 | Acute gastritis | rs7016302 | G | C | -0.0687 | 0.09065 | 0.04059 | Chronic gastritis | rs7016302 | G | C | 0.00108 | 0.95809 | 0.02052 | Gastroduodenal ulcer | rs7016302 | G | C | 0.02088 | 0.31961 | 0.02098 | Gastric cancer | rs7016302 | G | C | 0.04062 | 0.44718 | 0.05344 | Fatty liver | rs7016302 | G | C | 0.03239 | 0.44664 | 0.04256 | Hepatic fibrosis | rs7016302 | G | C | -0.2224 | 0.16956 | 0.16189 | Cirrhosis | rs7016302 | G | C | -0.0076 | 0.80855 | 0.03132liver cancer rs7016302 G C 0.09312 0.2046 0.0734 Choleliths rs7016302 G C 0.01819 0.09303 0.01083 Acute pancreatitis rs7016302 G C -0.0295 0.24332 0.02525 Chronic pancreatitis rs7016302 G C -0.037 0.27732 0.03406 Pancreatic cancer rs7016302 G C -0.0105 0.84225 0.05286 Ulcerative colitis rs7016302 G C 0.00672 0.81019 0.02796 Crohn's disease rs7016302 G C -0.0526 0.27463 0.04816 Irrtable bowel syndrome rs7016302 G C -0.0078 0.70898 0.02097 Acute appendicitis rs7016302 G C -0.0025 0.8382 0.01202 Colon cancer rs7016302 G C -0.0559 0.09009 0.03301 Rectal cancer rs7016302 G C 0.00904 0.82906 0.04189 |
| Gastroesophageal reflux | rs702606 | T | C | 0.002 | 0.88582 | 0.01391 | Esophageal carcinoma | rs702606 | T | C | -0.0396 | 0.66196 | 0.09057 | Acute gastritis | rs702606 | T | C | 0.02573 | 0.55752 | 0.04387 | Chronic gastritis | rs702606 | T | C | -0.0052 | 0.81441 | 0.02227 | Gastroduodenal ulcer | rs702606 | T | C | -0.0143 | 0.52949 | 0.02276 | Gastric cancer | rs702606 | T | C | -0.0071 | 0.90297 | 0.05818 | Fatty liver | rs702606 | T | C | 0.12321 | 0.00904 | 0.0472 | Hepatic fibrosis | rs702606 | T | C | -0.1017 | 0.55995 | 0.17445 | Cirrhosis | rs702606 | T | C | -0.0063 | 0.85179 | 0.03393liver cancer rs702606 T C 0.10488 0.18889 0.07983 Choleliths rs702606 T C 0.0063 0.59273 0.01177 Acute pancreatitis rs702606 T C -0.0656 0.01653 0.02735 Chronic pancreatitis rs702606 T C -0.0355 0.33524 0.03683 Pancreatic cancer rs702606 T C -0.0061 0.91519 0.05747 Ulcerative colitis rs702606 T C -0.0591 0.05062 0.03026 Crohn's disease rs702606 T C 0.0261 0.61682 0.05216 Irrtable bowel syndrome rs702606 T C -0.0172 0.44702 0.02268 Acute appendicitis rs702606 T C -0.0151 0.24675 0.01302 Colon cancer rs702606 T C -0.0445 0.21616 0.03597 Rectal cancer rs702606 T C 0.00138 0.97594 0.04574 |
| Gastroesophageal reflux | rs7029718 | G | A | 0.00775 | 0.43442 | 0.00991 | Esophageal carcinoma | rs7029718 | G | A | -0.02 | 0.75619 | 0.06432 | Acute gastritis | rs7029718 | G | A | 0.01749 | 0.5762 | 0.03129 | Chronic gastritis | rs7029718 | G | A | 0.00402 | 0.79964 | 0.01586 | Gastroduodenal ulcer | rs7029718 | G | A | 0.03314 | 0.04098 | 0.01622 | Gastric cancer | rs7029718 | G | A | 0.00631 | 0.8785 | 0.04126 | Fatty liver | rs7029718 | G | A | -0.02 | 0.54331 | 0.03291 | Hepatic fibrosis | rs7029718 | G | A | -0.0611 | 0.62691 | 0.12568 | Cirrhosis | rs7029718 | G | A | 0.01963 | 0.41832 | 0.02425liver cancer rs7029718 G A 0.04104 0.4696 0.05676 Choleliths rs7029718 G A -0.0041 0.62628 0.00838 Acute pancreatitis rs7029718 G A 0.02237 0.25199 0.01953 Chronic pancreatitis rs7029718 G A 0.07533 0.0042 0.02632 Pancreatic cancer rs7029718 G A 0.04804 0.24018 0.0409 Ulcerative colitis rs7029718 G A -0.0165 0.4466 0.02162 Crohn's disease rs7029718 G A 0.05344 0.15108 0.03722 Irrtable bowel syndrome rs7029718 G A 0.0154 0.34141 0.01619 Acute appendicitis rs7029718 G A 0.01087 0.24241 0.0093 Colon cancer rs7029718 G A 0.00838 0.74228 0.02547 Rectal cancer rs7029718 G A 0.03665 0.25649 0.0323 |
| Gastroesophageal reflux | rs7031698 | C | T | 0.00945 | 0.40693 | 0.0114 | Esophageal carcinoma | rs7031698 | C | T | 0.02354 | 0.74875 | 0.07348 | Acute gastritis | rs7031698 | C | T | -0.0416 | 0.24664 | 0.0359 | Chronic gastritis | rs7031698 | C | T | -0.0115 | 0.52645 | 0.01822 | Gastroduodenal ulcer | rs7031698 | C | T | 0.01031 | 0.57917 | 0.01859 | Gastric cancer | rs7031698 | C | T | 0.03261 | 0.48914 | 0.04714 | Fatty liver | rs7031698 | C | T | 0.04339 | 0.2512 | 0.03782 | Hepatic fibrosis | rs7031698 | C | T | 0.04789 | 0.74106 | 0.14492 | Cirrhosis | rs7031698 | C | T | 0.01493 | 0.59185 | 0.02785liver cancer rs7031698 C T 0.03264 0.61412 0.06473 Choleliths rs7031698 C T -0.0193 0.04501 0.00961 Acute pancreatitis rs7031698 C T 0.00817 0.71589 0.02246 Chronic pancreatitis rs7031698 C T 0.03043 0.31497 0.03029 Pancreatic cancer rs7031698 C T -0.0039 0.93305 0.04681 Ulcerative colitis rs7031698 C T -0.0227 0.36078 0.02488 Crohn's disease rs7031698 C T -0.0262 0.54211 0.04299 Irrtable bowel syndrome rs7031698 C T -0.0148 0.42578 0.01862 Acute appendicitis rs7031698 C T 0.00256 0.8106 0.01069 Colon cancer rs7031698 C T -0.0103 0.72427 0.02917 Rectal cancer rs7031698 C T 0.02533 0.4942 0.03705 |
| Gastroesophageal reflux | rs710629 | A | G | -0.0209 | 0.03584 | 0.00997 | Esophageal carcinoma | rs710629 | A | G | -0.0571 | 0.37708 | 0.0646 | Acute gastritis | rs710629 | A | G | 0.01089 | 0.72941 | 0.0315 | Chronic gastritis | rs710629 | A | G | -0.0138 | 0.38543 | 0.01594 | Gastroduodenal ulcer | rs710629 | A | G | -0.0007 | 0.96613 | 0.01629 | Gastric cancer | rs710629 | A | G | 0.01133 | 0.78467 | 0.04147 | Fatty liver | rs710629 | A | G | -0.0412 | 0.21367 | 0.03315 | Hepatic fibrosis | rs710629 | A | G | -0.0249 | 0.84375 | 0.12627 | Cirrhosis | rs710629 | A | G | 0.00137 | 0.95528 | 0.02437liver cancer rs710629 A G 0.0663 0.24386 0.05689 Choleliths rs710629 A G -0.0219 0.00919 0.00842 Acute pancreatitis rs710629 A G -0.0269 0.17088 0.01966 Chronic pancreatitis rs710629 A G -0.0311 0.24026 0.02647 Pancreatic cancer rs710629 A G -0.0243 0.55479 0.04106 Ulcerative colitis rs710629 A G 0.0152 0.48493 0.02177 Crohn's disease rs710629 A G 0.04248 0.25842 0.03759 Irrtable bowel syndrome rs710629 A G -0.0139 0.39409 0.0163 Acute appendicitis rs710629 A G -0.0003 0.97834 0.00937 Colon cancer rs710629 A G -0.0001 0.99638 0.0256 Rectal cancer rs710629 A G -0.0027 0.93282 0.03251 |
| Gastroesophageal reflux | rs71646142 | C | T | -0.0136 | 0.31849 | 0.0136 | Esophageal carcinoma | rs71646142 | C | T | -0.0528 | 0.55033 | 0.0884 | Acute gastritis | rs71646142 | C | T | -0.0335 | 0.43563 | 0.04292 | Chronic gastritis | rs71646142 | C | T | -0.0378 | 0.08258 | 0.02177 | Gastroduodenal ulcer | rs71646142 | C | T | -0.0274 | 0.21971 | 0.02231 | Gastric cancer | rs71646142 | C | T | -0.0016 | 0.9771 | 0.05713 | Fatty liver | rs71646142 | C | T | -0.0479 | 0.28868 | 0.04511 | Hepatic fibrosis | rs71646142 | C | T | -0.0007 | 0.99666 | 0.17219 | Cirrhosis | rs71646142 | C | T | -0.0238 | 0.47497 | 0.03333liver cancer rs71646142 C T 0.00663 0.93232 0.07803 Choleliths rs71646142 C T -0.0036 0.75349 0.0115 Acute pancreatitis rs71646142 C T 0.02023 0.45184 0.02688 Chronic pancreatitis rs71646142 C T -0.035 0.33357 0.0362 Pancreatic cancer rs71646142 C T -0.1197 0.03376 0.05637 Ulcerative colitis rs71646142 C T -0.0095 0.7497 0.02964 Crohn's disease rs71646142 C T 0.00683 0.8937 0.05114 Irrtable bowel syndrome rs71646142 C T 0.00208 0.9253 0.02217 Acute appendicitis rs71646142 C T 0.00442 0.72903 0.01277 Colon cancer rs71646142 C T -0.0158 0.6532 0.0352 Rectal cancer rs71646142 C T 0.05556 0.21418 0.04473 |
| Gastroesophageal reflux | rs7233920 | A | G | 0.01362 | 0.21684 | 0.01103 | Esophageal carcinoma | rs7233920 | A | G | -0.0243 | 0.73196 | 0.07096 | Acute gastritis | rs7233920 | A | G | -0.0001 | 0.99662 | 0.03471 | Chronic gastritis | rs7233920 | A | G | 0.00794 | 0.65277 | 0.01765 | Gastroduodenal ulcer | rs7233920 | A | G | 0.01172 | 0.51468 | 0.01799 | Gastric cancer | rs7233920 | A | G | 0.01277 | 0.78019 | 0.04577 | Fatty liver | rs7233920 | A | G | 0.01125 | 0.75873 | 0.03664 | Hepatic fibrosis | rs7233920 | A | G | -0.2459 | 0.07911 | 0.14003 | Cirrhosis | rs7233920 | A | G | -0.0284 | 0.29185 | 0.02691liver cancer rs7233920 A G 0.01289 0.83722 0.06272 Choleliths rs7233920 A G 0.00183 0.84438 0.0093 Acute pancreatitis rs7233920 A G 0.03437 0.11289 0.02168 Chronic pancreatitis rs7233920 A G 0.02708 0.35419 0.02923 Pancreatic cancer rs7233920 A G 0.02798 0.53476 0.04508 Ulcerative colitis rs7233920 A G 0.0157 0.51435 0.02407 Crohn's disease rs7233920 A G 0.01993 0.63124 0.04152 Irrtable bowel syndrome rs7233920 A G 0.01328 0.46111 0.01802 Acute appendicitis rs7233920 A G 0.02758 0.00734 0.01029 Colon cancer rs7233920 A G 0.04058 0.15053 0.02823 Rectal cancer rs7233920 A G 0.03287 0.35841 0.03579 |
| Gastroesophageal reflux | rs7257460 | T | C | -0.0075 | 0.51469 | 0.01154 | Esophageal carcinoma | rs7257460 | T | C | 0.00488 | 0.94777 | 0.07444 | Acute gastritis | rs7257460 | T | C | -0.0884 | 0.01559 | 0.03656 | Chronic gastritis | rs7257460 | T | C | -0.0182 | 0.32443 | 0.01845 | Gastroduodenal ulcer | rs7257460 | T | C | -0.0214 | 0.25858 | 0.0189 | Gastric cancer | rs7257460 | T | C | -0.0223 | 0.64148 | 0.04788 | Fatty liver | rs7257460 | T | C | -0.0306 | 0.42454 | 0.03836 | Hepatic fibrosis | rs7257460 | T | C | -0.1509 | 0.30614 | 0.14743 | Cirrhosis | rs7257460 | T | C | -0.0269 | 0.34165 | 0.02831liver cancer rs7257460 T C 0.00916 0.88933 0.06584 Choleliths rs7257460 T C -0.0097 0.32189 0.00976 Acute pancreatitis rs7257460 T C -0.0251 0.27027 0.02279 Chronic pancreatitis rs7257460 T C -0.0627 0.04177 0.03079 Pancreatic cancer rs7257460 T C 0.02707 0.56938 0.04758 Ulcerative colitis rs7257460 T C -0.0397 0.11561 0.0252 Crohn's disease rs7257460 T C 0.03798 0.38194 0.04344 Irrtable bowel syndrome rs7257460 T C 0.01146 0.54317 0.01885 Acute appendicitis rs7257460 T C 0.00974 0.36942 0.01086 Colon cancer rs7257460 T C 0.02541 0.38883 0.02949 Rectal cancer rs7257460 T C -0.0052 0.88845 0.03743 |
| Gastroesophageal reflux | rs7278859 | A | T | 0.00476 | 0.67322 | 0.01129 | Esophageal carcinoma | rs7278859 | A | T | 0.07789 | 0.28259 | 0.07249 | Acute gastritis | rs7278859 | A | T | -0.0156 | 0.66116 | 0.03563 | Chronic gastritis | rs7278859 | A | T | -0.0285 | 0.11352 | 0.01803 | Gastroduodenal ulcer | rs7278859 | A | T | -0.0382 | 0.03843 | 0.01845 | Gastric cancer | rs7278859 | A | T | 0.00292 | 0.95046 | 0.04696 | Fatty liver | rs7278859 | A | T | -0.0547 | 0.14517 | 0.03752 | Hepatic fibrosis | rs7278859 | A | T | 0.03874 | 0.78735 | 0.14362 | Cirrhosis | rs7278859 | A | T | -0.0279 | 0.31192 | 0.02759liver cancer rs7278859 A T -0.1319 0.03935 0.064 Choleliths rs7278859 A T 0.01264 0.18529 0.00955 Acute pancreatitis rs7278859 A T 0.03305 0.13716 0.02223 Chronic pancreatitis rs7278859 A T 0.008 0.79001 0.03003 Pancreatic cancer rs7278859 A T 0.0421 0.36301 0.04628 Ulcerative colitis rs7278859 A T 0.00518 0.83357 0.02467 Crohn's disease rs7278859 A T -0.0233 0.58433 0.04262 Irrtable bowel syndrome rs7278859 A T -0.0054 0.7708 0.01847 Acute appendicitis rs7278859 A T 0.00029 0.97818 0.01061 Colon cancer rs7278859 A T 0.02887 0.31658 0.02883 Rectal cancer rs7278859 A T 0.01824 0.61848 0.03664 |
| Gastroesophageal reflux | rs72807818 | G | A | -0.007 | 0.67675 | 0.01684 | Esophageal carcinoma | rs72807818 | G | A | -0.0523 | 0.63268 | 0.10941 | Acute gastritis | rs72807818 | G | A | -0.0435 | 0.41229 | 0.05304 | Chronic gastritis | rs72807818 | G | A | -0.0079 | 0.76946 | 0.0268 | Gastroduodenal ulcer | rs72807818 | G | A | -0.0167 | 0.54541 | 0.02757 | Gastric cancer | rs72807818 | G | A | 0.02537 | 0.71617 | 0.06977 | Fatty liver | rs72807818 | G | A | -0.0015 | 0.97866 | 0.05602 | Hepatic fibrosis | rs72807818 | G | A | -0.0941 | 0.66249 | 0.21555 | Cirrhosis | rs72807818 | G | A | -0.0472 | 0.25547 | 0.04148liver cancer rs72807818 G A -0.0118 0.90203 0.09604 Choleliths rs72807818 G A -0.0205 0.14807 0.0142 Acute pancreatitis rs72807818 G A -0.0015 0.96433 0.03341 Chronic pancreatitis rs72807818 G A 0.01697 0.70773 0.04526 Pancreatic cancer rs72807818 G A 0.16927 0.01497 0.06957 Ulcerative colitis rs72807818 G A 0.01695 0.64651 0.03697 Crohn's disease rs72807818 G A -0.0343 0.58914 0.0636 Irrtable bowel syndrome rs72807818 G A -0.0385 0.1614 0.02746 Acute appendicitis rs72807818 G A 0.01342 0.39796 0.01588 Colon cancer rs72807818 G A 0.05242 0.22665 0.04335 Rectal cancer rs72807818 G A -0.0837 0.1269 0.05485 |
| Gastroesophageal reflux | rs72828517 | C | T | -0.0258 | 0.06101 | 0.0138 | Esophageal carcinoma | rs72828517 | C | T | 0.04983 | 0.57774 | 0.08951 | Acute gastritis | rs72828517 | C | T | -0.0481 | 0.26937 | 0.04353 | Chronic gastritis | rs72828517 | C | T | -0.011 | 0.61816 | 0.02205 | Gastroduodenal ulcer | rs72828517 | C | T | -0.015 | 0.5067 | 0.02258 | Gastric cancer | rs72828517 | C | T | 0.054 | 0.34686 | 0.0574 | Fatty liver | rs72828517 | C | T | -0.0254 | 0.57919 | 0.04582 | Hepatic fibrosis | rs72828517 | C | T | -0.1902 | 0.27969 | 0.17598 | Cirrhosis | rs72828517 | C | T | -0.0823 | 0.0152 | 0.03391liver cancer rs72828517 C T -0.1583 0.04583 0.07929 Choleliths rs72828517 C T -0.0326 0.00547 0.01172 Acute pancreatitis rs72828517 C T -0.0679 0.01274 0.02728 Chronic pancreatitis rs72828517 C T -0.0569 0.12191 0.03679 Pancreatic cancer rs72828517 C T -0.0219 0.7006 0.05688 Ulcerative colitis rs72828517 C T -0.0226 0.45457 0.03016 Crohn's disease rs72828517 C T 0.06196 0.2326 0.05191 Irrtable bowel syndrome rs72828517 C T -0.0212 0.3452 0.02251 Acute appendicitis rs72828517 C T 0.02147 0.0978 0.01297 Colon cancer rs72828517 C T 0.02496 0.48166 0.03548 Rectal cancer rs72828517 C T -0.0647 0.15041 0.04498 |
| Gastroesophageal reflux | rs72840994 | G | T | 0.00212 | 0.86511 | 0.01251 | Esophageal carcinoma | rs72840994 | G | T | 0.02068 | 0.79779 | 0.08072 | Acute gastritis | rs72840994 | G | T | 0.007 | 0.85839 | 0.03924 | Chronic gastritis | rs72840994 | G | T | -0.004 | 0.84158 | 0.01999 | Gastroduodenal ulcer | rs72840994 | G | T | -0.0402 | 0.04855 | 0.02037 | Gastric cancer | rs72840994 | G | T | -0.0954 | 0.06468 | 0.05162 | Fatty liver | rs72840994 | G | T | 0.06252 | 0.13229 | 0.04154 | Hepatic fibrosis | rs72840994 | G | T | 0.12719 | 0.42044 | 0.15787 | Cirrhosis | rs72840994 | G | T | 0.0059 | 0.84667 | 0.03049liver cancer rs72840994 G T -0.0985 0.16544 0.07103 Choleliths rs72840994 G T -0.0154 0.14364 0.01056 Acute pancreatitis rs72840994 G T -0.0324 0.1866 0.02457 Chronic pancreatitis rs72840994 G T -0.0009 0.9783 0.03313 Pancreatic cancer rs72840994 G T -0.0008 0.98815 0.05119 Ulcerative colitis rs72840994 G T -0.0182 0.50445 0.02726 Crohn's disease rs72840994 G T -0.0397 0.39937 0.04715 Irrtable bowel syndrome rs72840994 G T 0.00363 0.85908 0.02042 Acute appendicitis rs72840994 G T -0.0115 0.32625 0.0117 Colon cancer rs72840994 G T 0.02607 0.4161 0.03206 Rectal cancer rs72840994 G T 0.05196 0.2017 0.0407 |
| Gastroesophageal reflux | rs730384 | G | A | 0.00274 | 0.78314 | 0.00997 | Esophageal carcinoma | rs730384 | G | A | -0.0133 | 0.83694 | 0.06451 | Acute gastritis | rs730384 | G | A | 0.03562 | 0.25794 | 0.03149 | Chronic gastritis | rs730384 | G | A | 0.01253 | 0.43163 | 0.01593 | Gastroduodenal ulcer | rs730384 | G | A | 0.01701 | 0.29687 | 0.0163 | Gastric cancer | rs730384 | G | A | -0.0252 | 0.54372 | 0.04143 | Fatty liver | rs730384 | G | A | 0.03514 | 0.28829 | 0.0331 | Hepatic fibrosis | rs730384 | G | A | -0.0773 | 0.54059 | 0.12625 | Cirrhosis | rs730384 | G | A | 0.01619 | 0.50655 | 0.02437liver cancer rs730384 G A 0.00717 0.89943 0.05677 Choleliths rs730384 G A -0.002 0.81313 0.00843 Acute pancreatitis rs730384 G A -0.0016 0.93575 0.01966 Chronic pancreatitis rs730384 G A -0.0361 0.17341 0.02651 Pancreatic cancer rs730384 G A -0.0482 0.24024 0.04106 Ulcerative colitis rs730384 G A 0.042 0.05377 0.02178 Crohn's disease rs730384 G A -0.0174 0.64285 0.03753 Irrtable bowel syndrome rs730384 G A 0.01067 0.51253 0.01629 Acute appendicitis rs730384 G A 0.0099 0.29035 0.00937 Colon cancer rs730384 G A -0.0157 0.54038 0.02557 Rectal cancer rs730384 G A -0.0398 0.22024 0.03245 |
| Gastroesophageal reflux | rs7315713 | A | T | -0.0351 | 0.00089 | 0.01055 | Esophageal carcinoma | rs7315713 | A | T | -0.157 | 0.02139 | 0.06823 | Acute gastritis | rs7315713 | A | T | -0.0324 | 0.32968 | 0.0332 | Chronic gastritis | rs7315713 | A | T | -0.0211 | 0.2085 | 0.01681 | Gastroduodenal ulcer | rs7315713 | A | T | -0.0243 | 0.15712 | 0.0172 | Gastric cancer | rs7315713 | A | T | -0.061 | 0.16378 | 0.04382 | Fatty liver | rs7315713 | A | T | -0.0248 | 0.47783 | 0.03494 | Hepatic fibrosis | rs7315713 | A | T | 0.01172 | 0.93 | 0.13345 | Cirrhosis | rs7315713 | A | T | -0.0284 | 0.2706 | 0.02575liver cancer rs7315713 A T 0.0372 0.53668 0.06022 Choleliths rs7315713 A T -0.0042 0.63434 0.00889 Acute pancreatitis rs7315713 A T -0.0202 0.33113 0.02074 Chronic pancreatitis rs7315713 A T 0.02566 0.35884 0.02797 Pancreatic cancer rs7315713 A T 0.04325 0.31994 0.04349 Ulcerative colitis rs7315713 A T 0.00442 0.84751 0.02299 Crohn's disease rs7315713 A T -0.0336 0.39767 0.03969 Irrtable bowel syndrome rs7315713 A T -0.0287 0.09463 0.01719 Acute appendicitis rs7315713 A T 0.00066 0.94666 0.00989 Colon cancer rs7315713 A T 0.04355 0.10644 0.02698 Rectal cancer rs7315713 A T -0.0319 0.35307 0.03436 |
| Gastroesophageal reflux | rs73301698 | A | G | 0.01379 | 0.26307 | 0.01232 | Esophageal carcinoma | rs73301698 | A | G | -0.0752 | 0.34399 | 0.07952 | Acute gastritis | rs73301698 | A | G | 0.03227 | 0.40674 | 0.0389 | Chronic gastritis | rs73301698 | A | G | 0.02735 | 0.16485 | 0.01969 | Gastroduodenal ulcer | rs73301698 | A | G | 0.03941 | 0.05025 | 0.02013 | Gastric cancer | rs73301698 | A | G | -0.0093 | 0.85521 | 0.05107 | Fatty liver | rs73301698 | A | G | -0.0167 | 0.68341 | 0.04094 | Hepatic fibrosis | rs73301698 | A | G | 0.05758 | 0.71425 | 0.15726 | Cirrhosis | rs73301698 | A | G | 0.03163 | 0.29495 | 0.0302liver cancer rs73301698 A G 0.05128 0.46526 0.07023 Choleliths rs73301698 A G 0.01874 0.07169 0.01041 Acute pancreatitis rs73301698 A G 0.02501 0.304 0.02433 Chronic pancreatitis rs73301698 A G 0.02818 0.39106 0.03285 Pancreatic cancer rs73301698 A G -0.0287 0.56991 0.05059 Ulcerative colitis rs73301698 A G -0.018 0.5032 0.02694 Crohn's disease rs73301698 A G 0.01694 0.71541 0.04646 Irrtable bowel syndrome rs73301698 A G 0.00676 0.7367 0.02012 Acute appendicitis rs73301698 A G 0.00856 0.45991 0.01158 Colon cancer rs73301698 A G -0.0134 0.67179 0.03157 Rectal cancer rs73301698 A G -0.0675 0.09205 0.04006 |
| Gastroesophageal reflux | rs7332724 | T | C | 0.03119 | 0.00307 | 0.01054 | Esophageal carcinoma | rs7332724 | T | C | -0.0014 | 0.98357 | 0.06852 | Acute gastritis | rs7332724 | T | C | 0.05214 | 0.11802 | 0.03335 | Chronic gastritis | rs7332724 | T | C | 0.01055 | 0.53278 | 0.01692 | Gastroduodenal ulcer | rs7332724 | T | C | 0.01466 | 0.39691 | 0.0173 | Gastric cancer | rs7332724 | T | C | 0.01351 | 0.75887 | 0.04401 | Fatty liver | rs7332724 | T | C | 0.04819 | 0.17016 | 0.03513 | Hepatic fibrosis | rs7332724 | T | C | -0.1256 | 0.3486 | 0.13399 | Cirrhosis | rs7332724 | T | C | 0.01481 | 0.5664 | 0.02584liver cancer rs7332724 T C -0.0267 0.65926 0.06053 Choleliths rs7332724 T C 0.0025 0.77979 0.00894 Acute pancreatitis rs7332724 T C 0.03332 0.10985 0.02084 Chronic pancreatitis rs7332724 T C -0.0133 0.63481 0.02809 Pancreatic cancer rs7332724 T C 0.00866 0.84245 0.04356 Ulcerative colitis rs7332724 T C -0.0006 0.97803 0.02306 Crohn's disease rs7332724 T C 0.02862 0.47154 0.03975 Irrtable bowel syndrome rs7332724 T C -0.016 0.35459 0.01729 Acute appendicitis rs7332724 T C -0.0011 0.9129 0.00992 Colon cancer rs7332724 T C 0.00988 0.71575 0.02715 Rectal cancer rs7332724 T C -0.0413 0.23061 0.03449 |
| Gastroesophageal reflux | rs73344830 | G | A | 0.00975 | 0.3237 | 0.00988 | Esophageal carcinoma | rs73344830 | G | A | -0.0016 | 0.98063 | 0.06399 | Acute gastritis | rs73344830 | G | A | 0.0618 | 0.04774 | 0.03122 | Chronic gastritis | rs73344830 | G | A | 0.00637 | 0.68671 | 0.01579 | Gastroduodenal ulcer | rs73344830 | G | A | 0.03447 | 0.03288 | 0.01615 | Gastric cancer | rs73344830 | G | A | 0.02393 | 0.5596 | 0.04102 | Fatty liver | rs73344830 | G | A | 0.08439 | 0.01009 | 0.0328 | Hepatic fibrosis | rs73344830 | G | A | -0.0224 | 0.85789 | 0.12524 | Cirrhosis | rs73344830 | G | A | 0.01582 | 0.51263 | 0.02417liver cancer rs73344830 G A 0.08371 0.1365 0.05622 Choleliths rs73344830 G A 0.01355 0.10488 0.00836 Acute pancreatitis rs73344830 G A -0.0041 0.8344 0.01947 Chronic pancreatitis rs73344830 G A 0.03184 0.22556 0.02627 Pancreatic cancer rs73344830 G A -0.0148 0.71495 0.04062 Ulcerative colitis rs73344830 G A 0.00568 0.79207 0.02156 Crohn's disease rs73344830 G A -3E-05 0.99945 0.03717 Irrtable bowel syndrome rs73344830 G A -0.0216 0.18057 0.01615 Acute appendicitis rs73344830 G A -0.0059 0.52446 0.00928 Colon cancer rs73344830 G A -0.0308 0.22395 0.02532 Rectal cancer rs73344830 G A -0.0501 0.11898 0.03214 |
| Gastroesophageal reflux | rs736282 | C | T | -0.0071 | 0.47695 | 0.00997 | Esophageal carcinoma | rs736282 | C | T | 0.02446 | 0.70464 | 0.06453 | Acute gastritis | rs736282 | C | T | 0.02322 | 0.4608 | 0.03149 | Chronic gastritis | rs736282 | C | T | 0.02642 | 0.09753 | 0.01594 | Gastroduodenal ulcer | rs736282 | C | T | 0.00811 | 0.61888 | 0.01631 | Gastric cancer | rs736282 | C | T | -0.0002 | 0.99626 | 0.04149 | Fatty liver | rs736282 | C | T | 0.05669 | 0.08688 | 0.03311 | Hepatic fibrosis | rs736282 | C | T | 0.17824 | 0.15832 | 0.12634 | Cirrhosis | rs736282 | C | T | -0.0015 | 0.94966 | 0.0244liver cancer rs736282 C T -0.0585 0.30481 0.05699 Choleliths rs736282 C T 0.01645 0.05116 0.00844 Acute pancreatitis rs736282 C T -0.0063 0.75014 0.01968 Chronic pancreatitis rs736282 C T -0.0452 0.08847 0.02651 Pancreatic cancer rs736282 C T 0.00645 0.87544 0.04113 Ulcerative colitis rs736282 C T -0.0243 0.26408 0.02177 Crohn's disease rs736282 C T 0.04902 0.19179 0.03755 Irrtable bowel syndrome rs736282 C T 0.00769 0.63659 0.01629 Acute appendicitis rs736282 C T 0.01365 0.14498 0.00937 Colon cancer rs736282 C T 0.01198 0.63958 0.02558 Rectal cancer rs736282 C T 0.02972 0.36072 0.03251 |
| Gastroesophageal reflux | rs73874335 | C | T | -0.0232 | 0.37797 | 0.0263 | Esophageal carcinoma | rs73874335 | C | T | -0.3229 | 0.05521 | 0.1684 | Acute gastritis | rs73874335 | C | T | -0.0378 | 0.65127 | 0.08352 | Chronic gastritis | rs73874335 | C | T | 0.02014 | 0.63131 | 0.04197 | Gastroduodenal ulcer | rs73874335 | C | T | 0.01515 | 0.72457 | 0.043 | Gastric cancer | rs73874335 | C | T | -0.0417 | 0.70293 | 0.10946 | Fatty liver | rs73874335 | C | T | 0.00165 | 0.98484 | 0.087 | Hepatic fibrosis | rs73874335 | C | T | 0.48408 | 0.15173 | 0.3377 | Cirrhosis | rs73874335 | C | T | 0.03839 | 0.54993 | 0.06422liver cancer rs73874335 C T 0.06777 0.64965 0.1492 Choleliths rs73874335 C T -0.0025 0.9103 0.02212 Acute pancreatitis rs73874335 C T -0.0597 0.25095 0.05197 Chronic pancreatitis rs73874335 C T -0.0871 0.21448 0.07013 Pancreatic cancer rs73874335 C T 0.00523 0.96131 0.10772 Ulcerative colitis rs73874335 C T -0.0011 0.98496 0.05774 Crohn's disease rs73874335 C T -0.0252 0.7991 0.09912 Irrtable bowel syndrome rs73874335 C T -0.0191 0.65608 0.04292 Acute appendicitis rs73874335 C T -9E-05 0.99714 0.02481 Colon cancer rs73874335 C T -0.0516 0.43898 0.06669 Rectal cancer rs73874335 C T -0.0965 0.25491 0.0848 |
| Gastroesophageal reflux | rs743316 | T | C | 0.0003 | 0.97854 | 0.01104 | Esophageal carcinoma | rs743316 | T | C | -0.0097 | 0.89185 | 0.07145 | Acute gastritis | rs743316 | T | C | 0.01709 | 0.62411 | 0.03488 | Chronic gastritis | rs743316 | T | C | -0.0069 | 0.69708 | 0.01766 | Gastroduodenal ulcer | rs743316 | T | C | -0.0081 | 0.65236 | 0.01803 | Gastric cancer | rs743316 | T | C | 0.00858 | 0.85109 | 0.04572 | Fatty liver | rs743316 | T | C | 0.00831 | 0.82063 | 0.03665 | Hepatic fibrosis | rs743316 | T | C | 0.26697 | 0.05573 | 0.13955 | Cirrhosis | rs743316 | T | C | -0.0329 | 0.22325 | 0.027liver cancer rs743316 T C -0.1221 0.05261 0.06299 Choleliths rs743316 T C 0.00028 0.97581 0.00933 Acute pancreatitis rs743316 T C -0.0303 0.16368 0.02175 Chronic pancreatitis rs743316 T C -0.0486 0.09711 0.02931 Pancreatic cancer rs743316 T C 0.00864 0.84909 0.04541 Ulcerative colitis rs743316 T C -0.0162 0.50111 0.02406 Crohn's disease rs743316 T C -0.0124 0.76473 0.04152 Irrtable bowel syndrome rs743316 T C 0.01034 0.56647 0.01804 Acute appendicitis rs743316 T C 0.00222 0.83039 0.01035 Colon cancer rs743316 T C -0.0388 0.1701 0.02828 Rectal cancer rs743316 T C -0.0015 0.96702 0.03604 |
| Gastroesophageal reflux | rs74643044 | T | C | 0.0014 | 0.94689 | 0.02098 | Esophageal carcinoma | rs74643044 | T | C | 0.03837 | 0.77761 | 0.13586 | Acute gastritis | rs74643044 | T | C | -0.0492 | 0.46143 | 0.06687 | Chronic gastritis | rs74643044 | T | C | -0.0291 | 0.38629 | 0.0336 | Gastroduodenal ulcer | rs74643044 | T | C | 0.03785 | 0.27117 | 0.0344 | Gastric cancer | rs74643044 | T | C | 0.02076 | 0.81025 | 0.08646 | Fatty liver | rs74643044 | T | C | 0.12053 | 0.08342 | 0.06962 | Hepatic fibrosis | rs74643044 | T | C | 0.20237 | 0.45017 | 0.26799 | Cirrhosis | rs74643044 | T | C | 0.07753 | 0.13295 | 0.0516liver cancer rs74643044 T C 0.04856 0.68505 0.11972 Choleliths rs74643044 T C -0.013 0.46507 0.01775 Acute pancreatitis rs74643044 T C 0.0616 0.13554 0.04127 Chronic pancreatitis rs74643044 T C 0.06326 0.25517 0.05559 Pancreatic cancer rs74643044 T C -0.0357 0.67911 0.08641 Ulcerative colitis rs74643044 T C 0.00371 0.93543 0.04585 Crohn's disease rs74643044 T C -0.0377 0.63296 0.07903 Irrtable bowel syndrome rs74643044 T C -0.0501 0.14339 0.03423 Acute appendicitis rs74643044 T C 0.02227 0.25921 0.01974 Colon cancer rs74643044 T C -0.0271 0.61434 0.0537 Rectal cancer rs74643044 T C 0.05332 0.4334 0.06807 |
| Gastroesophageal reflux | rs74701752 | G | T | -0.0145 | 0.38005 | 0.01654 | Esophageal carcinoma | rs74701752 | G | T | -0.1812 | 0.09197 | 0.10754 | Acute gastritis | rs74701752 | G | T | 0.03171 | 0.54296 | 0.05213 | Chronic gastritis | rs74701752 | G | T | -0.0385 | 0.14493 | 0.02643 | Gastroduodenal ulcer | rs74701752 | G | T | -0.0571 | 0.03408 | 0.02696 | Gastric cancer | rs74701752 | G | T | -0.1004 | 0.14281 | 0.06848 | Fatty liver | rs74701752 | G | T | -0.0813 | 0.13814 | 0.05486 | Hepatic fibrosis | rs74701752 | G | T | -0.0886 | 0.67402 | 0.21055 | Cirrhosis | rs74701752 | G | T | -0.0305 | 0.45035 | 0.04047liver cancer rs74701752 G T -0.0759 0.421 0.0943 Choleliths rs74701752 G T -0.0058 0.67462 0.01392 Acute pancreatitis rs74701752 G T -0.0159 0.62377 0.03247 Chronic pancreatitis rs74701752 G T 0.07359 0.0944 0.04399 Pancreatic cancer rs74701752 G T 0.0746 0.27299 0.06805 Ulcerative colitis rs74701752 G T 0.03257 0.36585 0.03602 Crohn's disease rs74701752 G T 0.03657 0.55614 0.06213 Irrtable bowel syndrome rs74701752 G T -0.013 0.62974 0.02691 Acute appendicitis rs74701752 G T -0.0243 0.11625 0.01548 Colon cancer rs74701752 G T 0.04061 0.33857 0.04243 Rectal cancer rs74701752 G T 0.0243 0.65063 0.05367 |
| Gastroesophageal reflux | rs74998289 | T | G | 0.03821 | 0.03173 | 0.01779 | Esophageal carcinoma | rs74998289 | T | G | -0.0196 | 0.86486 | 0.11515 | Acute gastritis | rs74998289 | T | G | -0.0558 | 0.32043 | 0.05616 | Chronic gastritis | rs74998289 | T | G | 0.01998 | 0.48103 | 0.02835 | Gastroduodenal ulcer | rs74998289 | T | G | -0.0348 | 0.23252 | 0.02915 | Gastric cancer | rs74998289 | T | G | 0.11378 | 0.1197 | 0.07312 | Fatty liver | rs74998289 | T | G | -0.0591 | 0.3187 | 0.05925 | Hepatic fibrosis | rs74998289 | T | G | -0.2772 | 0.23633 | 0.23412 | Cirrhosis | rs74998289 | T | G | 0.07026 | 0.11283 | 0.04431liver cancer rs74998289 T G 0.18384 0.07307 0.10257 Choleliths rs74998289 T G 0.01533 0.30615 0.01498 Acute pancreatitis rs74998289 T G -0.0197 0.57758 0.03539 Chronic pancreatitis rs74998289 T G -0.0301 0.53341 0.04826 Pancreatic cancer rs74998289 T G -0.0311 0.67415 0.07386 Ulcerative colitis rs74998289 T G 0.03875 0.32243 0.03916 Crohn's disease rs74998289 T G 0.09814 0.14556 0.06743 Irrtable bowel syndrome rs74998289 T G 0.00234 0.93582 0.02904 Acute appendicitis rs74998289 T G 0.0153 0.36441 0.01687 Colon cancer rs74998289 T G 0.05597 0.2141 0.04505 Rectal cancer rs74998289 T G 0.03429 0.54837 0.05712 |
| Gastroesophageal reflux | rs7543462 | A | G | 0.01896 | 0.05326 | 0.00981 | Esophageal carcinoma | rs7543462 | A | G | 0.04699 | 0.45941 | 0.06352 | Acute gastritis | rs7543462 | A | G | 0.03865 | 0.21183 | 0.03096 | Chronic gastritis | rs7543462 | A | G | 0.02797 | 0.07454 | 0.01568 | Gastroduodenal ulcer | rs7543462 | A | G | 0.02748 | 0.08665 | 0.01604 | Gastric cancer | rs7543462 | A | G | 0.06513 | 0.11153 | 0.04093 | Fatty liver | rs7543462 | A | G | -0.0454 | 0.16374 | 0.03259 | Hepatic fibrosis | rs7543462 | A | G | 0.03711 | 0.76567 | 0.1245 | Cirrhosis | rs7543462 | A | G | 0.00731 | 0.76036 | 0.02398liver cancer rs7543462 A G -0.0789 0.15925 0.05606 Choleliths rs7543462 A G 0.00279 0.73655 0.0083 Acute pancreatitis rs7543462 A G 0.00197 0.91873 0.01934 Chronic pancreatitis rs7543462 A G 0.05147 0.04831 0.02606 Pancreatic cancer rs7543462 A G 0.00836 0.83639 0.04049 Ulcerative colitis rs7543462 A G 0.0243 0.25595 0.02139 Crohn's disease rs7543462 A G 0.01004 0.78562 0.03691 Irrtable bowel syndrome rs7543462 A G -0.0027 0.86724 0.01604 Acute appendicitis rs7543462 A G 0.00561 0.54247 0.00921 Colon cancer rs7543462 A G -0.0104 0.6814 0.02523 Rectal cancer rs7543462 A G -0.0072 0.82296 0.03202 |
| Gastroesophageal reflux | rs7594904 | C | T | -0.0081 | 0.41302 | 0.00984 | Esophageal carcinoma | rs7594904 | C | T | 0.05897 | 0.35427 | 0.06366 | Acute gastritis | rs7594904 | C | T | -0.0488 | 0.11644 | 0.03107 | Chronic gastritis | rs7594904 | C | T | -0.002 | 0.90007 | 0.01574 | Gastroduodenal ulcer | rs7594904 | C | T | -0.0259 | 0.10757 | 0.01609 | Gastric cancer | rs7594904 | C | T | -0.013 | 0.75048 | 0.041 | Fatty liver | rs7594904 | C | T | -0.0207 | 0.52581 | 0.03264 | Hepatic fibrosis | rs7594904 | C | T | -0.0683 | 0.58333 | 0.12454 | Cirrhosis | rs7594904 | C | T | -0.0059 | 0.8067 | 0.02403liver cancer rs7594904 C T -0.0574 0.30707 0.05622 Choleliths rs7594904 C T -0.0039 0.63643 0.00831 Acute pancreatitis rs7594904 C T -0.0101 0.60404 0.01938 Chronic pancreatitis rs7594904 C T 0.02971 0.25518 0.02611 Pancreatic cancer rs7594904 C T -0.0029 0.94276 0.04057 Ulcerative colitis rs7594904 C T 0.02564 0.23122 0.02142 Crohn's disease rs7594904 C T 0.06305 0.08815 0.03697 Irrtable bowel syndrome rs7594904 C T -0.0025 0.87596 0.01607 Acute appendicitis rs7594904 C T -0.0015 0.86676 0.00922 Colon cancer rs7594904 C T 0.03059 0.22677 0.0253 Rectal cancer rs7594904 C T -0.0182 0.57131 0.03214 |
| Gastroesophageal reflux | rs7603132 | G | A | 0.01215 | 0.30342 | 0.0118 | Esophageal carcinoma | rs7603132 | G | A | 0.09893 | 0.1973 | 0.07673 | Acute gastritis | rs7603132 | G | A | -0.0232 | 0.5335 | 0.03719 | Chronic gastritis | rs7603132 | G | A | 0.00273 | 0.88505 | 0.01891 | Gastroduodenal ulcer | rs7603132 | G | A | 0.01231 | 0.52307 | 0.01928 | Gastric cancer | rs7603132 | G | A | -0.0214 | 0.66351 | 0.04928 | Fatty liver | rs7603132 | G | A | -0.0466 | 0.23449 | 0.03916 | Hepatic fibrosis | rs7603132 | G | A | 0.08026 | 0.58863 | 0.14841 | Cirrhosis | rs7603132 | G | A | 0.00167 | 0.95379 | 0.02877liver cancer rs7603132 G A 0.02616 0.69771 0.06735 Choleliths rs7603132 G A 0.01875 0.05978 0.00996 Acute pancreatitis rs7603132 G A 0.02221 0.33867 0.02321 Chronic pancreatitis rs7603132 G A 0.0432 0.16646 0.03122 Pancreatic cancer rs7603132 G A 0.04689 0.33456 0.04859 Ulcerative colitis rs7603132 G A -0.046 0.07314 0.02566 Crohn's disease rs7603132 G A 0.09406 0.03365 0.04428 Irrtable bowel syndrome rs7603132 G A -0.0094 0.62333 0.01924 Acute appendicitis rs7603132 G A -0.0111 0.31323 0.01104 Colon cancer rs7603132 G A -0.001 0.97376 0.03044 Rectal cancer rs7603132 G A 0.07157 0.06386 0.03862 |
| Gastroesophageal reflux | rs76076331 | C | T | 0.02965 | 0.01708 | 0.01243 | Esophageal carcinoma | rs76076331 | C | T | -0.0858 | 0.2848 | 0.08024 | Acute gastritis | rs76076331 | C | T | 0.05003 | 0.20136 | 0.03916 | Chronic gastritis | rs76076331 | C | T | 0.02519 | 0.20527 | 0.01989 | Gastroduodenal ulcer | rs76076331 | C | T | 0.01347 | 0.5069 | 0.0203 | Gastric cancer | rs76076331 | C | T | 0.03947 | 0.44771 | 0.05198 | Fatty liver | rs76076331 | C | T | -0.0115 | 0.78056 | 0.04118 | Hepatic fibrosis | rs76076331 | C | T | -0.1709 | 0.27434 | 0.15635 | Cirrhosis | rs76076331 | C | T | 0.00106 | 0.97206 | 0.03029liver cancer rs76076331 C T -0.0667 0.34497 0.07062 Choleliths rs76076331 C T 0.01101 0.29378 0.01049 Acute pancreatitis rs76076331 C T 0.01148 0.63846 0.02443 Chronic pancreatitis rs76076331 C T 0.00196 0.95256 0.03287 Pancreatic cancer rs76076331 C T 0.0307 0.54832 0.05114 Ulcerative colitis rs76076331 C T 0.00245 0.92789 0.02706 Crohn's disease rs76076331 C T 0.05997 0.19822 0.04661 Irrtable bowel syndrome rs76076331 C T 0.00476 0.81455 0.02028 Acute appendicitis rs76076331 C T -0.0295 0.01126 0.01164 Colon cancer rs76076331 C T -0.0269 0.40074 0.03198 Rectal cancer rs76076331 C T -0.0284 0.4841 0.04064 |
| Gastroesophageal reflux | rs7650602 | T | C | -0.0023 | 0.81699 | 0.00985 | Esophageal carcinoma | rs7650602 | T | C | 0.05375 | 0.401 | 0.064 | Acute gastritis | rs7650602 | T | C | -0.0262 | 0.40045 | 0.03112 | Chronic gastritis | rs7650602 | T | C | -0.01 | 0.52347 | 0.01575 | Gastroduodenal ulcer | rs7650602 | T | C | -0.0059 | 0.71326 | 0.0161 | Gastric cancer | rs7650602 | T | C | -0.0146 | 0.72238 | 0.04104 | Fatty liver | rs7650602 | T | C | 0.00397 | 0.90339 | 0.0327 | Hepatic fibrosis | rs7650602 | T | C | 0.18692 | 0.13508 | 0.12509 | Cirrhosis | rs7650602 | T | C | 0.08348 | 0.00052 | 0.02405liver cancer rs7650602 T C -0.0315 0.57661 0.05647 Choleliths rs7650602 T C -0.0121 0.14589 0.00833 Acute pancreatitis rs7650602 T C 0.00819 0.67296 0.0194 Chronic pancreatitis rs7650602 T C 0.02538 0.33217 0.02617 Pancreatic cancer rs7650602 T C 0.00213 0.95821 0.04068 Ulcerative colitis rs7650602 T C 0.00862 0.68855 0.0215 Crohn's disease rs7650602 T C -0.0999 0.0065 0.03672 Irrtable bowel syndrome rs7650602 T C 0.01802 0.26265 0.01609 Acute appendicitis rs7650602 T C -0.0054 0.55997 0.00925 Colon cancer rs7650602 T C -0.0387 0.12715 0.02534 Rectal cancer rs7650602 T C -0.0465 0.14798 0.03215 |
| Gastroesophageal reflux | rs76608582 | C | A | -0.0135 | 0.52604 | 0.02122 | Esophageal carcinoma | rs76608582 | C | A | 0.22166 | 0.10571 | 0.13702 | Acute gastritis | rs76608582 | C | A | 0.09506 | 0.15413 | 0.0667 | Chronic gastritis | rs76608582 | C | A | 0.01204 | 0.72255 | 0.03392 | Gastroduodenal ulcer | rs76608582 | C | A | -0.0134 | 0.69897 | 0.03474 | Gastric cancer | rs76608582 | C | A | 0.09647 | 0.27794 | 0.08891 | Fatty liver | rs76608582 | C | A | -0.027 | 0.70138 | 0.07036 | Hepatic fibrosis | rs76608582 | C | A | -0.6509 | 0.01497 | 0.26751 | Cirrhosis | rs76608582 | C | A | 0.05523 | 0.28435 | 0.05159liver cancer rs76608582 C A 0.14583 0.22693 0.12069 Choleliths rs76608582 C A 0.02288 0.20324 0.01798 Acute pancreatitis rs76608582 C A 0.04187 0.31581 0.04174 Chronic pancreatitis rs76608582 C A -0.0274 0.62509 0.05604 Pancreatic cancer rs76608582 C A 0.06454 0.46325 0.08799 Ulcerative colitis rs76608582 C A 0.01513 0.74307 0.04617 Crohn's disease rs76608582 C A -0.1051 0.18573 0.07941 Irrtable bowel syndrome rs76608582 C A -0.0333 0.33459 0.03456 Acute appendicitis rs76608582 C A -0.0217 0.27478 0.01985 Colon cancer rs76608582 C A -0.0112 0.83802 0.05468 Rectal cancer rs76608582 C A 0.13661 0.04885 0.06935 |
| Gastroesophageal reflux | rs76878669 | C | G | -0.0007 | 0.95061 | 0.01139 | Esophageal carcinoma | rs76878669 | C | G | 0.01291 | 0.86113 | 0.07379 | Acute gastritis | rs76878669 | C | G | -0.0143 | 0.69131 | 0.03607 | Chronic gastritis | rs76878669 | C | G | -0.0238 | 0.19138 | 0.01822 | Gastroduodenal ulcer | rs76878669 | C | G | 0.00806 | 0.66567 | 0.01866 | Gastric cancer | rs76878669 | C | G | -0.0468 | 0.3249 | 0.04755 | Fatty liver | rs76878669 | C | G | 0.02507 | 0.5074 | 0.03783 | Hepatic fibrosis | rs76878669 | C | G | 0.0988 | 0.49224 | 0.14386 | Cirrhosis | rs76878669 | C | G | 0.02161 | 0.43743 | 0.02783liver cancer rs76878669 C G -0.0035 0.95664 0.06515 Choleliths rs76878669 C G -0.0101 0.29307 0.00965 Acute pancreatitis rs76878669 C G -0.0197 0.3804 0.02245 Chronic pancreatitis rs76878669 C G -0.0095 0.75435 0.03028 Pancreatic cancer rs76878669 C G 0.05977 0.204 0.04706 Ulcerative colitis rs76878669 C G -0.0769 0.00167 0.02448 Crohn's disease rs76878669 C G -0.1055 0.0137 0.0428 Irrtable bowel syndrome rs76878669 C G -0.0245 0.18792 0.01862 Acute appendicitis rs76878669 C G 0.00982 0.35892 0.01071 Colon cancer rs76878669 C G -0.0658 0.02429 0.02919 Rectal cancer rs76878669 C G -0.049 0.18692 0.03714 |
| Gastroesophageal reflux | rs77025239 | A | G | 0.00958 | 0.46117 | 0.01299 | Esophageal carcinoma | rs77025239 | A | G | -0.1268 | 0.13019 | 0.08379 | Acute gastritis | rs77025239 | A | G | 0.07662 | 0.06237 | 0.04111 | Chronic gastritis | rs77025239 | A | G | 0.03417 | 0.09987 | 0.02077 | Gastroduodenal ulcer | rs77025239 | A | G | 0.0185 | 0.38454 | 0.02127 | Gastric cancer | rs77025239 | A | G | 0.00295 | 0.95655 | 0.05413 | Fatty liver | rs77025239 | A | G | -0.0357 | 0.40712 | 0.04304 | Hepatic fibrosis | rs77025239 | A | G | -0.0652 | 0.69028 | 0.1636 | Cirrhosis | rs77025239 | A | G | 0.01473 | 0.64208 | 0.03169liver cancer rs77025239 A G 0.0008 0.99136 0.07431 Choleliths rs77025239 A G -0.0053 0.63221 0.01099 Acute pancreatitis rs77025239 A G -0.0175 0.4932 0.02555 Chronic pancreatitis rs77025239 A G -0.0499 0.1462 0.03437 Pancreatic cancer rs77025239 A G -0.0151 0.77703 0.05334 Ulcerative colitis rs77025239 A G -0.0134 0.63583 0.02829 Crohn's disease rs77025239 A G 0.01723 0.72354 0.04872 Irrtable bowel syndrome rs77025239 A G -0.0053 0.80363 0.02124 Acute appendicitis rs77025239 A G 0.00835 0.49357 0.01219 Colon cancer rs77025239 A G 0.00949 0.7758 0.03331 Rectal cancer rs77025239 A G -0.0962 0.0226 0.04219 |
| Gastroesophageal reflux | rs77128898 | T | C | -0.0107 | 0.6283 | 0.02215 | Esophageal carcinoma | rs77128898 | T | C | -0.2018 | 0.1616 | 0.14414 | Acute gastritis | rs77128898 | T | C | 0.03597 | 0.60722 | 0.06997 | Chronic gastritis | rs77128898 | T | C | 0.02909 | 0.41173 | 0.03544 | Gastroduodenal ulcer | rs77128898 | T | C | 0.03364 | 0.35395 | 0.03629 | Gastric cancer | rs77128898 | T | C | 0.05244 | 0.57521 | 0.09357 | Fatty liver | rs77128898 | T | C | 0.17037 | 0.02027 | 0.0734 | Hepatic fibrosis | rs77128898 | T | C | 0.08248 | 0.76592 | 0.27704 | Cirrhosis | rs77128898 | T | C | -0.0405 | 0.4513 | 0.0538liver cancer rs77128898 T C -0.1089 0.38781 0.12606 Choleliths rs77128898 T C -0.0081 0.66676 0.01876 Acute pancreatitis rs77128898 T C 0.03143 0.47132 0.04364 Chronic pancreatitis rs77128898 T C 0.00077 0.9895 0.05857 Pancreatic cancer rs77128898 T C -0.0727 0.42617 0.09141 Ulcerative colitis rs77128898 T C -0.0263 0.58374 0.04806 Crohn's disease rs77128898 T C -0.0677 0.41321 0.08279 Irrtable bowel syndrome rs77128898 T C 0.04672 0.1983 0.03632 Acute appendicitis rs77128898 T C 0.02752 0.18435 0.02073 Colon cancer rs77128898 T C -0.0138 0.80822 0.05701 Rectal cancer rs77128898 T C 0.09507 0.19134 0.07276 |
| Gastroesophageal reflux | rs77702622 | A | G | 0.02861 | 0.27088 | 0.02598 | Esophageal carcinoma | rs77702622 | A | G | -0.1078 | 0.51918 | 0.16718 | Acute gastritis | rs77702622 | A | G | -0.1397 | 0.09237 | 0.08303 | Chronic gastritis | rs77702622 | A | G | 0.02389 | 0.5643 | 0.04144 | Gastroduodenal ulcer | rs77702622 | A | G | 0.04268 | 0.317 | 0.04265 | Gastric cancer | rs77702622 | A | G | -0.1075 | 0.32387 | 0.10901 | Fatty liver | rs77702622 | A | G | -0.018 | 0.83521 | 0.08647 | Hepatic fibrosis | rs77702622 | A | G | -0.497 | 0.13461 | 0.33216 | Cirrhosis | rs77702622 | A | G | 0.00021 | 0.99736 | 0.0639liver cancer rs77702622 A G -0.2377 0.10963 0.14861 Choleliths rs77702622 A G -0.02 0.36641 0.02216 Acute pancreatitis rs77702622 A G -0.0332 0.52114 0.05168 Chronic pancreatitis rs77702622 A G 0.06031 0.38629 0.06961 Pancreatic cancer rs77702622 A G 0.05981 0.58216 0.1087 Ulcerative colitis rs77702622 A G -0.0997 0.08191 0.05732 Crohn's disease rs77702622 A G -0.0101 0.91823 0.09845 Irrtable bowel syndrome rs77702622 A G 0.0401 0.34567 0.04252 Acute appendicitis rs77702622 A G -0.0002 0.99498 0.0246 Colon cancer rs77702622 A G -0.0054 0.93548 0.06727 Rectal cancer rs77702622 A G -0.1474 0.08425 0.08539 |
| Gastroesophageal reflux | rs77719387 | A | T | 0.03782 | 0.40031 | 0.04496 | Esophageal carcinoma | rs77719387 | A | T | 0.39774 | 0.18113 | 0.29742 | Acute gastritis | rs77719387 | A | T | -0.0901 | 0.5327 | 0.14442 | Chronic gastritis | rs77719387 | A | T | 0.07655 | 0.28647 | 0.07182 | Gastroduodenal ulcer | rs77719387 | A | T | 0.14892 | 0.04293 | 0.07356 | Gastric cancer | rs77719387 | A | T | 0.03096 | 0.86998 | 0.18914 | Fatty liver | rs77719387 | A | T | -0.1026 | 0.49037 | 0.14879 | Hepatic fibrosis | rs77719387 | A | T | 0.10092 | 0.86192 | 0.58021 | Cirrhosis | rs77719387 | A | T | 0.0995 | 0.3644 | 0.1097liver cancer rs77719387 A T 0.20699 0.42264 0.25814 Choleliths rs77719387 A T 0.03268 0.3889 0.03792 Acute pancreatitis rs77719387 A T 0.01442 0.8704 0.08837 Chronic pancreatitis rs77719387 A T -0.0066 0.95601 0.11934 Pancreatic cancer rs77719387 A T 0.24076 0.20507 0.18999 Ulcerative colitis rs77719387 A T -0.0084 0.93195 0.09829 Crohn's disease rs77719387 A T 0.11514 0.4965 0.16932 Irrtable bowel syndrome rs77719387 A T 0.11117 0.12972 0.07337 Acute appendicitis rs77719387 A T 0.09524 0.02463 0.04238 Colon cancer rs77719387 A T -0.1533 0.18359 0.11528 Rectal cancer rs77719387 A T 0.36256 0.01254 0.14522 |
| Gastroesophageal reflux | rs7773815 | A | C | 0.00403 | 0.6913 | 0.01015 | Esophageal carcinoma | rs7773815 | A | C | -0.049 | 0.45745 | 0.06593 | Acute gastritis | rs7773815 | A | C | -0.0168 | 0.60011 | 0.03207 | Chronic gastritis | rs7773815 | A | C | -0.028 | 0.08433 | 0.01622 | Gastroduodenal ulcer | rs7773815 | A | C | -0.0252 | 0.12932 | 0.01663 | Gastric cancer | rs7773815 | A | C | 0.0236 | 0.57634 | 0.04224 | Fatty liver | rs7773815 | A | C | -0.02 | 0.55316 | 0.03372 | Hepatic fibrosis | rs7773815 | A | C | 0.11851 | 0.35815 | 0.12897 | Cirrhosis | rs7773815 | A | C | 0.03289 | 0.1869 | 0.02492liver cancer rs7773815 A C -0.0359 0.53673 0.0581 Choleliths rs7773815 A C -0.0053 0.53674 0.00858 Acute pancreatitis rs7773815 A C -0.0044 0.82481 0.02005 Chronic pancreatitis rs7773815 A C -0.0169 0.53199 0.02707 Pancreatic cancer rs7773815 A C 0.02339 0.57686 0.04193 Ulcerative colitis rs7773815 A C 0.00689 0.75624 0.02219 Crohn's disease rs7773815 A C -0.0075 0.84511 0.03823 Irrtable bowel syndrome rs7773815 A C -0.0115 0.48859 0.01656 Acute appendicitis rs7773815 A C 0.01851 0.05256 0.00955 Colon cancer rs7773815 A C -0.0003 0.98963 0.02608 Rectal cancer rs7773815 A C -0.043 0.1934 0.03308 |
| Gastroesophageal reflux | rs77835879 | A | G | 0.00233 | 0.9005 | 0.01866 | Esophageal carcinoma | rs77835879 | A | G | 0.21621 | 0.07495 | 0.12141 | Acute gastritis | rs77835879 | A | G | 0.02673 | 0.65115 | 0.05912 | Chronic gastritis | rs77835879 | A | G | -0.0167 | 0.57443 | 0.02982 | Gastroduodenal ulcer | rs77835879 | A | G | 0.02061 | 0.50143 | 0.03066 | Gastric cancer | rs77835879 | A | G | -0.0131 | 0.86624 | 0.07769 | Fatty liver | rs77835879 | A | G | 0.01451 | 0.81461 | 0.06188 | Hepatic fibrosis | rs77835879 | A | G | -0.0049 | 0.98375 | 0.23886 | Cirrhosis | rs77835879 | A | G | 0.01911 | 0.67697 | 0.04587liver cancer rs77835879 A G 0.1444 0.17334 0.10606 Choleliths rs77835879 A G 0.01846 0.24324 0.01582 Acute pancreatitis rs77835879 A G -0.0258 0.48451 0.03694 Chronic pancreatitis rs77835879 A G 0.00431 0.93113 0.04987 Pancreatic cancer rs77835879 A G 0.06739 0.38312 0.07727 Ulcerative colitis rs77835879 A G -0.0066 0.87147 0.0409 Crohn's disease rs77835879 A G -0.0451 0.52044 0.07025 Irrtable bowel syndrome rs77835879 A G -0.0529 0.0822 0.03042 Acute appendicitis rs77835879 A G 0.03662 0.03744 0.0176 Colon cancer rs77835879 A G 0.0334 0.48674 0.04802 Rectal cancer rs77835879 A G 0.07819 0.19715 0.06063 |
| Gastroesophageal reflux | rs7796203 | G | A | 0.014 | 0.16764 | 0.01015 | Esophageal carcinoma | rs7796203 | G | A | 0.05511 | 0.402 | 0.06575 | Acute gastritis | rs7796203 | G | A | -0.0118 | 0.71253 | 0.03209 | Chronic gastritis | rs7796203 | G | A | -0.0179 | 0.26862 | 0.01622 | Gastroduodenal ulcer | rs7796203 | G | A | -0.0323 | 0.05163 | 0.0166 | Gastric cancer | rs7796203 | G | A | 0.00449 | 0.91515 | 0.04212 | Fatty liver | rs7796203 | G | A | 0.00484 | 0.88602 | 0.03374 | Hepatic fibrosis | rs7796203 | G | A | 0.23603 | 0.06712 | 0.12892 | Cirrhosis | rs7796203 | G | A | 0.00145 | 0.95363 | 0.02489liver cancer rs7796203 G A 0.06136 0.28969 0.05796 Choleliths rs7796203 G A 0.01136 0.18568 0.00858 Acute pancreatitis rs7796203 G A -0.0342 0.08761 0.02004 Chronic pancreatitis rs7796203 G A -0.0265 0.32681 0.02704 Pancreatic cancer rs7796203 G A -0.0504 0.22859 0.04184 Ulcerative colitis rs7796203 G A 0.02816 0.20438 0.02219 Crohn's disease rs7796203 G A -0.003 0.93823 0.03825 Irrtable bowel syndrome rs7796203 G A 0.0351 0.03439 0.01659 Acute appendicitis rs7796203 G A 0.01169 0.22086 0.00955 Colon cancer rs7796203 G A -0.0061 0.8133 0.02602 Rectal cancer rs7796203 G A 0.01966 0.55109 0.03298 |
| Gastroesophageal reflux | rs7803932 | A | G | -0.0104 | 0.44911 | 0.01376 | Esophageal carcinoma | rs7803932 | A | G | 0.02354 | 0.7913 | 0.08894 | Acute gastritis | rs7803932 | A | G | -0.0202 | 0.6416 | 0.04344 | Chronic gastritis | rs7803932 | A | G | -0.0365 | 0.09587 | 0.02193 | Gastroduodenal ulcer | rs7803932 | A | G | -0.0135 | 0.54755 | 0.02243 | Gastric cancer | rs7803932 | A | G | -0.0693 | 0.22414 | 0.05698 | Fatty liver | rs7803932 | A | G | 0.01133 | 0.80466 | 0.0458 | Hepatic fibrosis | rs7803932 | A | G | 0.2885 | 0.10078 | 0.1758 | Cirrhosis | rs7803932 | A | G | 0.03538 | 0.29479 | 0.03377liver cancer rs7803932 A G -0.0178 0.82121 0.07871 Choleliths rs7803932 A G 0.01662 0.15285 0.01162 Acute pancreatitis rs7803932 A G -0.0467 0.08571 0.02718 Chronic pancreatitis rs7803932 A G -0.044 0.23155 0.03677 Pancreatic cancer rs7803932 A G -0.013 0.81924 0.05676 Ulcerative colitis rs7803932 A G -0.0438 0.14696 0.03017 Crohn's disease rs7803932 A G -0.0544 0.29505 0.05199 Irrtable bowel syndrome rs7803932 A G 0.0161 0.4749 0.02253 Acute appendicitis rs7803932 A G -0.0201 0.12187 0.01296 Colon cancer rs7803932 A G -0.0042 0.90482 0.03515 Rectal cancer rs7803932 A G -0.0013 0.97605 0.04472 |
| Gastroesophageal reflux | rs7808399 | A | G | 0.03097 | 0.00157 | 0.0098 | Esophageal carcinoma | rs7808399 | A | G | -0.0047 | 0.94121 | 0.06326 | Acute gastritis | rs7808399 | A | G | 0.01612 | 0.60219 | 0.03093 | Chronic gastritis | rs7808399 | A | G | 0.02266 | 0.14786 | 0.01566 | Gastroduodenal ulcer | rs7808399 | A | G | 0.00129 | 0.93564 | 0.01603 | Gastric cancer | rs7808399 | A | G | -0.0202 | 0.61987 | 0.04074 | Fatty liver | rs7808399 | A | G | -0.024 | 0.46075 | 0.03254 | Hepatic fibrosis | rs7808399 | A | G | 0.01612 | 0.89667 | 0.12413 | Cirrhosis | rs7808399 | A | G | 0.04947 | 0.03921 | 0.02399liver cancer rs7808399 A G -0.0365 0.51291 0.05585 Choleliths rs7808399 A G 0.00999 0.22781 0.00829 Acute pancreatitis rs7808399 A G 0.0477 0.01351 0.01931 Chronic pancreatitis rs7808399 A G -0.0129 0.62143 0.02604 Pancreatic cancer rs7808399 A G -0.0264 0.51334 0.04034 Ulcerative colitis rs7808399 A G 0.00569 0.79005 0.02137 Crohn's disease rs7808399 A G -0.0159 0.66673 0.03685 Irrtable bowel syndrome rs7808399 A G -0.0003 0.98349 0.01602 Acute appendicitis rs7808399 A G 0.00403 0.66169 0.0092 Colon cancer rs7808399 A G 0.03552 0.15727 0.02511 Rectal cancer rs7808399 A G -0.0218 0.49515 0.03191 |
| Gastroesophageal reflux | rs7833201 | C | G | 0.00203 | 0.90332 | 0.01673 | Esophageal carcinoma | rs7833201 | C | G | -0.0576 | 0.5911 | 0.10719 | Acute gastritis | rs7833201 | C | G | 0.05245 | 0.32075 | 0.05283 | Chronic gastritis | rs7833201 | C | G | 0.00563 | 0.83266 | 0.02664 | Gastroduodenal ulcer | rs7833201 | C | G | -0.0031 | 0.90861 | 0.02735 | Gastric cancer | rs7833201 | C | G | -0.0842 | 0.22705 | 0.0697 | Fatty liver | rs7833201 | C | G | -0.005 | 0.92786 | 0.05554 | Hepatic fibrosis | rs7833201 | C | G | 0.07831 | 0.71269 | 0.21267 | Cirrhosis | rs7833201 | C | G | 0.02312 | 0.57126 | 0.04084liver cancer rs7833201 C G 0.14801 0.11871 0.09487 Choleliths rs7833201 C G 0.01708 0.22708 0.01414 Acute pancreatitis rs7833201 C G -0.0043 0.89636 0.03305 Chronic pancreatitis rs7833201 C G -0.0049 0.91322 0.04462 Pancreatic cancer rs7833201 C G -0.0792 0.24996 0.06883 Ulcerative colitis rs7833201 C G 0.00096 0.97909 0.03672 Crohn's disease rs7833201 C G -0.0012 0.9852 0.06305 Irrtable bowel syndrome rs7833201 C G -0.0161 0.55791 0.02739 Acute appendicitis rs7833201 C G 0.01678 0.28669 0.01575 Colon cancer rs7833201 C G -0.0231 0.58812 0.0427 Rectal cancer rs7833201 C G -0.0105 0.84546 0.05409 |
| Gastroesophageal reflux | rs7863447 | G | A | -0.0083 | 0.50157 | 0.01229 | Esophageal carcinoma | rs7863447 | G | A | 0.08726 | 0.27578 | 0.08007 | Acute gastritis | rs7863447 | G | A | -0.028 | 0.46982 | 0.03881 | Chronic gastritis | rs7863447 | G | A | 0.00748 | 0.70374 | 0.01967 | Gastroduodenal ulcer | rs7863447 | G | A | 0.02266 | 0.25942 | 0.0201 | Gastric cancer | rs7863447 | G | A | -0.0015 | 0.97591 | 0.05113 | Fatty liver | rs7863447 | G | A | 0.06487 | 0.11097 | 0.0407 | Hepatic fibrosis | rs7863447 | G | A | -0.1211 | 0.43569 | 0.15538 | Cirrhosis | rs7863447 | G | A | 0.05059 | 0.09243 | 0.03006liver cancer rs7863447 G A 0.09255 0.18867 0.0704 Choleliths rs7863447 G A 0.01293 0.21293 0.01038 Acute pancreatitis rs7863447 G A 0.06244 0.00885 0.02385 Chronic pancreatitis rs7863447 G A -0.0191 0.55707 0.03259 Pancreatic cancer rs7863447 G A -0.0111 0.82731 0.05069 Ulcerative colitis rs7863447 G A -0.0301 0.2611 0.02675 Crohn's disease rs7863447 G A -0.0353 0.44457 0.04618 Irrtable bowel syndrome rs7863447 G A -0.0086 0.6688 0.02003 Acute appendicitis rs7863447 G A 0.00358 0.75578 0.01151 Colon cancer rs7863447 G A 0.0279 0.37891 0.0317 Rectal cancer rs7863447 G A 0.00667 0.86826 0.04021 |
| Gastroesophageal reflux | rs7864396 | C | A | -0.0014 | 0.89135 | 0.01003 | Esophageal carcinoma | rs7864396 | C | A | 0.06203 | 0.33981 | 0.06498 | Acute gastritis | rs7864396 | C | A | 0.03646 | 0.24949 | 0.03166 | Chronic gastritis | rs7864396 | C | A | 0.01587 | 0.32195 | 0.01602 | Gastroduodenal ulcer | rs7864396 | C | A | -0.0206 | 0.20964 | 0.01639 | Gastric cancer | rs7864396 | C | A | 0.03408 | 0.41346 | 0.04167 | Fatty liver | rs7864396 | C | A | 0.02957 | 0.37446 | 0.03329 | Hepatic fibrosis | rs7864396 | C | A | -0.0174 | 0.89097 | 0.12694 | Cirrhosis | rs7864396 | C | A | -0.0348 | 0.15521 | 0.02451liver cancer rs7864396 C A -0.0435 0.44572 0.0571 Choleliths rs7864396 C A 0.01046 0.21779 0.00849 Acute pancreatitis rs7864396 C A -0.0446 0.02398 0.01977 Chronic pancreatitis rs7864396 C A -0.0814 0.00228 0.02667 Pancreatic cancer rs7864396 C A -0.0719 0.08205 0.04133 Ulcerative colitis rs7864396 C A 0.01616 0.45993 0.02187 Crohn's disease rs7864396 C A -0.0331 0.37934 0.03768 Irrtable bowel syndrome rs7864396 C A -0.0049 0.76474 0.01639 Acute appendicitis rs7864396 C A -0.0016 0.86128 0.00942 Colon cancer rs7864396 C A 0.01927 0.4535 0.02571 Rectal cancer rs7864396 C A -0.0251 0.44249 0.03267 |
| Gastroesophageal reflux | rs78721320 | G | A | 0.00704 | 0.58014 | 0.01273 | Esophageal carcinoma | rs78721320 | G | A | 0.00709 | 0.93105 | 0.08194 | Acute gastritis | rs78721320 | G | A | 0.02016 | 0.61536 | 0.04013 | Chronic gastritis | rs78721320 | G | A | 0.00174 | 0.9318 | 0.02028 | Gastroduodenal ulcer | rs78721320 | G | A | 0.01716 | 0.40865 | 0.02077 | Gastric cancer | rs78721320 | G | A | 0.10051 | 0.05688 | 0.05278 | Fatty liver | rs78721320 | G | A | -0.027 | 0.5227 | 0.0422 | Hepatic fibrosis | rs78721320 | G | A | -0.2649 | 0.09865 | 0.16038 | Cirrhosis | rs78721320 | G | A | 0.04422 | 0.15509 | 0.0311liver cancer rs78721320 G A 0.03791 0.59979 0.07226 Choleliths rs78721320 G A 0.01533 0.15422 0.01076 Acute pancreatitis rs78721320 G A 0.01303 0.60222 0.02499 Chronic pancreatitis rs78721320 G A -0.0185 0.58299 0.0337 Pancreatic cancer rs78721320 G A 0.00016 0.99757 0.05225 Ulcerative colitis rs78721320 G A -0.0064 0.81843 0.02769 Crohn's disease rs78721320 G A 0.0187 0.69563 0.04779 Irrtable bowel syndrome rs78721320 G A -0.0306 0.14053 0.02078 Acute appendicitis rs78721320 G A 0.00359 0.76335 0.01193 Colon cancer rs78721320 G A -0.007 0.83091 0.03271 Rectal cancer rs78721320 G A 0.0455 0.27151 0.04138 |
| Gastroesophageal reflux | rs790647 | C | A | 0.00091 | 0.94038 | 0.01212 | Esophageal carcinoma | rs790647 | C | A | 0.01953 | 0.80328 | 0.0784 | Acute gastritis | rs790647 | C | A | -0.0425 | 0.26629 | 0.03827 | Chronic gastritis | rs790647 | C | A | -0.0093 | 0.63221 | 0.01938 | Gastroduodenal ulcer | rs790647 | C | A | -0.0047 | 0.81156 | 0.01982 | Gastric cancer | rs790647 | C | A | 0.05844 | 0.24561 | 0.05033 | Fatty liver | rs790647 | C | A | -0.0229 | 0.56971 | 0.04023 | Hepatic fibrosis | rs790647 | C | A | -0.1362 | 0.37539 | 0.15363 | Cirrhosis | rs790647 | C | A | 0.0071 | 0.81087 | 0.02966liver cancer rs790647 C A -0.0111 0.87179 0.06905 Choleliths rs790647 C A 0.00198 0.84676 0.01024 Acute pancreatitis rs790647 C A 0.01553 0.51541 0.02387 Chronic pancreatitis rs790647 C A 0.00051 0.98737 0.03217 Pancreatic cancer rs790647 C A 0.01635 0.74283 0.04982 Ulcerative colitis rs790647 C A -0.0164 0.53619 0.02646 Crohn's disease rs790647 C A 0.04793 0.29431 0.0457 Irrtable bowel syndrome rs790647 C A -0.0166 0.40054 0.0198 Acute appendicitis rs790647 C A 0.00872 0.44344 0.01138 Colon cancer rs790647 C A 0.00462 0.88151 0.03099 Rectal cancer rs790647 C A -0.0201 0.60983 0.03942 |
| Gastroesophageal reflux | rs7920624 | T | A | 0.00695 | 0.47998 | 0.00984 | Esophageal carcinoma | rs7920624 | T | A | 0.03733 | 0.55753 | 0.06364 | Acute gastritis | rs7920624 | T | A | -0.0294 | 0.34307 | 0.03103 | Chronic gastritis | rs7920624 | T | A | 0.00235 | 0.881 | 0.01572 | Gastroduodenal ulcer | rs7920624 | T | A | 0.00214 | 0.89415 | 0.01606 | Gastric cancer | rs7920624 | T | A | 0.07618 | 0.06258 | 0.04091 | Fatty liver | rs7920624 | T | A | -0.0026 | 0.93586 | 0.03268 | Hepatic fibrosis | rs7920624 | T | A | -0.0682 | 0.58551 | 0.12498 | Cirrhosis | rs7920624 | T | A | 0.04447 | 0.06456 | 0.02406liver cancer rs7920624 T A 0.12565 0.02542 0.05622 Choleliths rs7920624 T A 0.00167 0.84112 0.00832 Acute pancreatitis rs7920624 T A 0.00313 0.87169 0.01939 Chronic pancreatitis rs7920624 T A -0.0512 0.05015 0.02616 Pancreatic cancer rs7920624 T A -0.0453 0.26474 0.04057 Ulcerative colitis rs7920624 T A -0.0455 0.03427 0.02148 Crohn's disease rs7920624 T A -0.0033 0.92885 0.03711 Irrtable bowel syndrome rs7920624 T A -0.0093 0.56509 0.01611 Acute appendicitis rs7920624 T A -0.0134 0.1475 0.00925 Colon cancer rs7920624 T A -0.0259 0.30587 0.02525 Rectal cancer rs7920624 T A 0.01046 0.74435 0.03209 |
| Gastroesophageal reflux | rs7924036 | T | G | 0.00137 | 0.88886 | 0.0098 | Esophageal carcinoma | rs7924036 | T | G | 0.0368 | 0.56239 | 0.06352 | Acute gastritis | rs7924036 | T | G | -0.0077 | 0.80237 | 0.03092 | Chronic gastritis | rs7924036 | T | G | 0.01528 | 0.32942 | 0.01566 | Gastroduodenal ulcer | rs7924036 | T | G | -0.0213 | 0.18401 | 0.01601 | Gastric cancer | rs7924036 | T | G | -0.0233 | 0.56819 | 0.04082 | Fatty liver | rs7924036 | T | G | 0.08396 | 0.00951 | 0.03238 | Hepatic fibrosis | rs7924036 | T | G | 0.18759 | 0.1303 | 0.12399 | Cirrhosis | rs7924036 | T | G | 0.03024 | 0.20645 | 0.02394liver cancer rs7924036 T G 0.00891 0.87349 0.05597 Choleliths rs7924036 T G 0.04618 2.4E-08 0.00828 Acute pancreatitis rs7924036 T G -0.001 0.95965 0.0193 Chronic pancreatitis rs7924036 T G -0.0017 0.94717 0.02605 Pancreatic cancer rs7924036 T G -0.0609 0.13055 0.0403 Ulcerative colitis rs7924036 T G 0.05031 0.01856 0.02137 Crohn's disease rs7924036 T G 0.02865 0.43738 0.03689 Irrtable bowel syndrome rs7924036 T G -0.0087 0.58612 0.01601 Acute appendicitis rs7924036 T G -0.0077 0.40058 0.0092 Colon cancer rs7924036 T G -0.0119 0.63664 0.02517 Rectal cancer rs7924036 T G -0.0061 0.84802 0.03194 |
| Gastroesophageal reflux | rs79265434 | A | G | 0.01066 | 0.50124 | 0.01585 | Esophageal carcinoma | rs79265434 | A | G | -0.1284 | 0.21184 | 0.10282 | Acute gastritis | rs79265434 | A | G | 0.00394 | 0.93712 | 0.04993 | Chronic gastritis | rs79265434 | A | G | -0.0037 | 0.88533 | 0.02539 | Gastroduodenal ulcer | rs79265434 | A | G | 0.00276 | 0.91548 | 0.02599 | Gastric cancer | rs79265434 | A | G | -0.0043 | 0.94725 | 0.06564 | Fatty liver | rs79265434 | A | G | -0.014 | 0.7899 | 0.05268 | Hepatic fibrosis | rs79265434 | A | G | -0.1126 | 0.57685 | 0.20181 | Cirrhosis | rs79265434 | A | G | -0.0056 | 0.88525 | 0.03897liver cancer rs79265434 A G -0.2018 0.02629 0.09081 Choleliths rs79265434 A G 0.00248 0.85343 0.01341 Acute pancreatitis rs79265434 A G 0.01584 0.61369 0.03138 Chronic pancreatitis rs79265434 A G 0.05654 0.18253 0.04241 Pancreatic cancer rs79265434 A G -0.055 0.4005 0.06537 Ulcerative colitis rs79265434 A G 0.01384 0.68933 0.03462 Crohn's disease rs79265434 A G 0.04154 0.48755 0.05984 Irrtable bowel syndrome rs79265434 A G -0.0272 0.29324 0.02592 Acute appendicitis rs79265434 A G 0.02274 0.12742 0.01492 Colon cancer rs79265434 A G 0.05791 0.15467 0.04069 Rectal cancer rs79265434 A G -0.0153 0.76576 0.05148 |
| Gastroesophageal reflux | rs79269403 | G | A | -0.0152 | 0.18501 | 0.01149 | Esophageal carcinoma | rs79269403 | G | A | 0.12247 | 0.10168 | 0.07482 | Acute gastritis | rs79269403 | G | A | 0.04689 | 0.19613 | 0.03627 | Chronic gastritis | rs79269403 | G | A | -0.0029 | 0.87683 | 0.01841 | Gastroduodenal ulcer | rs79269403 | G | A | 0.01146 | 0.54226 | 0.0188 | Gastric cancer | rs79269403 | G | A | -0.0405 | 0.39835 | 0.04792 | Fatty liver | rs79269403 | G | A | -0.0127 | 0.73887 | 0.0382 | Hepatic fibrosis | rs79269403 | G | A | -0.1856 | 0.20246 | 0.14564 | Cirrhosis | rs79269403 | G | A | -0.0251 | 0.37038 | 0.02808liver cancer rs79269403 G A -0.0757 0.24948 0.06575 Choleliths rs79269403 G A 0.00385 0.69176 0.00971 Acute pancreatitis rs79269403 G A 0.02234 0.32331 0.02262 Chronic pancreatitis rs79269403 G A -0.0095 0.75542 0.0305 Pancreatic cancer rs79269403 G A -0.0398 0.40213 0.04754 Ulcerative colitis rs79269403 G A 0.01684 0.50099 0.02502 Crohn's disease rs79269403 G A 0.06017 0.1641 0.04324 Irrtable bowel syndrome rs79269403 G A -0.0055 0.76992 0.01878 Acute appendicitis rs79269403 G A 0.00137 0.899 0.01077 Colon cancer rs79269403 G A -0.0382 0.19737 0.02964 Rectal cancer rs79269403 G A -0.0744 0.04765 0.03757 |
| Gastroesophageal reflux | rs7928622 | T | A | -0.0019 | 0.85754 | 0.01051 | Esophageal carcinoma | rs7928622 | T | A | 0.09342 | 0.17026 | 0.06812 | Acute gastritis | rs7928622 | T | A | -0.0137 | 0.68059 | 0.03321 | Chronic gastritis | rs7928622 | T | A | -0.0255 | 0.1285 | 0.01678 | Gastroduodenal ulcer | rs7928622 | T | A | -0.0185 | 0.28226 | 0.0172 | Gastric cancer | rs7928622 | T | A | -0.0467 | 0.28529 | 0.04375 | Fatty liver | rs7928622 | T | A | 0.01439 | 0.68055 | 0.03495 | Hepatic fibrosis | rs7928622 | T | A | -0.1796 | 0.17909 | 0.13364 | Cirrhosis | rs7928622 | T | A | 0.02778 | 0.28155 | 0.02579liver cancer rs7928622 T A 0.03701 0.53792 0.06009 Choleliths rs7928622 T A -0.0215 0.01575 0.00891 Acute pancreatitis rs7928622 T A 0.01716 0.40907 0.02078 Chronic pancreatitis rs7928622 T A -0.0182 0.51586 0.02801 Pancreatic cancer rs7928622 T A -0.0545 0.20898 0.04337 Ulcerative colitis rs7928622 T A -0.0431 0.06092 0.023 Crohn's disease rs7928622 T A -0.0287 0.46895 0.0397 Irrtable bowel syndrome rs7928622 T A 0.00881 0.60808 0.01719 Acute appendicitis rs7928622 T A -0.0036 0.7188 0.00991 Colon cancer rs7928622 T A -0.0226 0.40157 0.02696 Rectal cancer rs7928622 T A -0.0236 0.49041 0.0342 |
| Gastroesophageal reflux | rs795230 | T | C | -0.0302 | 0.00307 | 0.01019 | Esophageal carcinoma | rs795230 | T | C | 0.00906 | 0.89035 | 0.06572 | Acute gastritis | rs795230 | T | C | -0.0023 | 0.94396 | 0.03207 | Chronic gastritis | rs795230 | T | C | 0.01207 | 0.45775 | 0.01625 | Gastroduodenal ulcer | rs795230 | T | C | -0.0076 | 0.64559 | 0.01663 | Gastric cancer | rs795230 | T | C | -0.075 | 0.07571 | 0.04224 | Fatty liver | rs795230 | T | C | 0.01891 | 0.57628 | 0.03384 | Hepatic fibrosis | rs795230 | T | C | -0.0084 | 0.94807 | 0.12875 | Cirrhosis | rs795230 | T | C | 0.01892 | 0.44674 | 0.02487liver cancer rs795230 T C 0.05352 0.35428 0.05777 Choleliths rs795230 T C 0.00335 0.69671 0.0086 Acute pancreatitis rs795230 T C 0.02526 0.20774 0.02005 Chronic pancreatitis rs795230 T C -0.0054 0.84077 0.02706 Pancreatic cancer rs795230 T C -0.0406 0.33047 0.04171 Ulcerative colitis rs795230 T C -0.0126 0.56984 0.02224 Crohn's disease rs795230 T C 0.02107 0.58316 0.0384 Irrtable bowel syndrome rs795230 T C -0.0315 0.05831 0.01664 Acute appendicitis rs795230 T C 0.00056 0.95368 0.00956 Colon cancer rs795230 T C 0.03555 0.17241 0.02605 Rectal cancer rs795230 T C -0.0113 0.73269 0.03314 |
| Gastroesophageal reflux | rs79523955 | A | G | -0.0031 | 0.83607 | 0.01482 | Esophageal carcinoma | rs79523955 | A | G | -0.032 | 0.73882 | 0.09603 | Acute gastritis | rs79523955 | A | G | -0.0538 | 0.24825 | 0.04659 | Chronic gastritis | rs79523955 | A | G | -0.011 | 0.64259 | 0.02366 | Gastroduodenal ulcer | rs79523955 | A | G | 0.00889 | 0.71314 | 0.02418 | Gastric cancer | rs79523955 | A | G | 0.14876 | 0.01573 | 0.0616 | Fatty liver | rs79523955 | A | G | -0.014 | 0.77687 | 0.04923 | Hepatic fibrosis | rs79523955 | A | G | 0.25247 | 0.17677 | 0.18691 | Cirrhosis | rs79523955 | A | G | 0.03031 | 0.40211 | 0.03617liver cancer rs79523955 A G 0.03069 0.71783 0.08492 Choleliths rs79523955 A G -0.0166 0.18529 0.01253 Acute pancreatitis rs79523955 A G -0.0364 0.21221 0.02914 Chronic pancreatitis rs79523955 A G 5.4E-05 0.99891 0.03934 Pancreatic cancer rs79523955 A G -9E-05 0.99877 0.06092 Ulcerative colitis rs79523955 A G -0.002 0.9508 0.03227 Crohn's disease rs79523955 A G -0.0595 0.2844 0.0556 Irrtable bowel syndrome rs79523955 A G -0.0092 0.70466 0.02418 Acute appendicitis rs79523955 A G -0.0047 0.73471 0.01388 Colon cancer rs79523955 A G 0.03005 0.43072 0.03813 Rectal cancer rs79523955 A G -0.0325 0.50215 0.04846 |
| Gastroesophageal reflux | rs7977614 | G | A | -0.0109 | 0.32013 | 0.01098 | Esophageal carcinoma | rs7977614 | G | A | -0.0426 | 0.55016 | 0.0713 | Acute gastritis | rs7977614 | G | A | -0.0068 | 0.84486 | 0.03467 | Chronic gastritis | rs7977614 | G | A | -0.0183 | 0.29657 | 0.01753 | Gastroduodenal ulcer | rs7977614 | G | A | 0.00881 | 0.62314 | 0.01794 | Gastric cancer | rs7977614 | G | A | 0.06568 | 0.15153 | 0.0458 | Fatty liver | rs7977614 | G | A | -0.0472 | 0.19527 | 0.03643 | Hepatic fibrosis | rs7977614 | G | A | 0.02345 | 0.86572 | 0.13866 | Cirrhosis | rs7977614 | G | A | -0.0038 | 0.88746 | 0.02681liver cancer rs7977614 G A 0.02849 0.65051 0.06289 Choleliths rs7977614 G A -0.0047 0.61093 0.00928 Acute pancreatitis rs7977614 G A 0.00579 0.78898 0.02162 Chronic pancreatitis rs7977614 G A -0.0002 0.99403 0.02913 Pancreatic cancer rs7977614 G A -0.0788 0.08231 0.04537 Ulcerative colitis rs7977614 G A -0.0022 0.92792 0.02393 Crohn's disease rs7977614 G A -0.0308 0.45566 0.04126 Irrtable bowel syndrome rs7977614 G A 0.0127 0.4787 0.01793 Acute appendicitis rs7977614 G A 0.00978 0.34281 0.01031 Colon cancer rs7977614 G A -0.0237 0.4016 0.0283 Rectal cancer rs7977614 G A 0.00337 0.92514 0.03582 |
| Gastroesophageal reflux | rs7993663 | T | C | 0.00455 | 0.65891 | 0.0103 | Esophageal carcinoma | rs7993663 | T | C | 0.00317 | 0.96226 | 0.06699 | Acute gastritis | rs7993663 | T | C | -0.0362 | 0.2651 | 0.03247 | Chronic gastritis | rs7993663 | T | C | 0.01788 | 0.27716 | 0.01645 | Gastroduodenal ulcer | rs7993663 | T | C | 0.00571 | 0.73426 | 0.01683 | Gastric cancer | rs7993663 | T | C | 0.04578 | 0.286 | 0.04291 | Fatty liver | rs7993663 | T | C | -0.0533 | 0.11922 | 0.03423 | Hepatic fibrosis | rs7993663 | T | C | -0.2183 | 0.09462 | 0.1306 | Cirrhosis | rs7993663 | T | C | -0.0136 | 0.58995 | 0.02518liver cancer rs7993663 T C 0.01396 0.81223 0.05875 Choleliths rs7993663 T C 0.00157 0.85673 0.00871 Acute pancreatitis rs7993663 T C -0.0053 0.79291 0.02031 Chronic pancreatitis rs7993663 T C -0.0099 0.71678 0.02736 Pancreatic cancer rs7993663 T C -0.0171 0.68608 0.04242 Ulcerative colitis rs7993663 T C -0.0064 0.77469 0.02251 Crohn's disease rs7993663 T C -0.0413 0.28756 0.0388 Irrtable bowel syndrome rs7993663 T C 0.02042 0.2252 0.01684 Acute appendicitis rs7993663 T C 0.00595 0.53851 0.00968 Colon cancer rs7993663 T C 0.00437 0.86902 0.02648 Rectal cancer rs7993663 T C 0.03172 0.34614 0.03366 |
| Gastroesophageal reflux | rs80171383 | A | G | 0.00271 | 0.84667 | 0.01399 | Esophageal carcinoma | rs80171383 | A | G | -0.0053 | 0.95305 | 0.0895 | Acute gastritis | rs80171383 | A | G | -0.0695 | 0.11553 | 0.04414 | Chronic gastritis | rs80171383 | A | G | -0.0101 | 0.65074 | 0.02241 | Gastroduodenal ulcer | rs80171383 | A | G | -0.0063 | 0.78361 | 0.02283 | Gastric cancer | rs80171383 | A | G | -0.072 | 0.21235 | 0.05771 | Fatty liver | rs80171383 | A | G | 0.01257 | 0.78643 | 0.04639 | Hepatic fibrosis | rs80171383 | A | G | -0.1856 | 0.29344 | 0.17667 | Cirrhosis | rs80171383 | A | G | -0.0605 | 0.07581 | 0.03405liver cancer rs80171383 A G -0.0585 0.45802 0.07883 Choleliths rs80171383 A G -0.0033 0.77962 0.0118 Acute pancreatitis rs80171383 A G -0.0737 0.00813 0.02785 Chronic pancreatitis rs80171383 A G -0.0365 0.32403 0.03705 Pancreatic cancer rs80171383 A G -0.1004 0.07782 0.05693 Ulcerative colitis rs80171383 A G 0.01504 0.62164 0.03047 Crohn's disease rs80171383 A G -0.0278 0.59698 0.0526 Irrtable bowel syndrome rs80171383 A G -0.0728 0.00173 0.02324 Acute appendicitis rs80171383 A G 0.01543 0.2381 0.01308 Colon cancer rs80171383 A G -0.0164 0.64555 0.03565 Rectal cancer rs80171383 A G -0.0471 0.29818 0.04523 |
| Gastroesophageal reflux | rs8020034 | G | A | 0.04343 | 0.00821 | 0.01643 | Esophageal carcinoma | rs8020034 | G | A | 0.09583 | 0.36855 | 0.10658 | Acute gastritis | rs8020034 | G | A | 0.15375 | 0.00376 | 0.05307 | Chronic gastritis | rs8020034 | G | A | 0.01299 | 0.61704 | 0.02597 | Gastroduodenal ulcer | rs8020034 | G | A | 0.02355 | 0.37766 | 0.02669 | Gastric cancer | rs8020034 | G | A | 0.0859 | 0.20263 | 0.06742 | Fatty liver | rs8020034 | G | A | 0.04726 | 0.38321 | 0.05419 | Hepatic fibrosis | rs8020034 | G | A | -0.3296 | 0.11554 | 0.20942 | Cirrhosis | rs8020034 | G | A | -0.0251 | 0.53161 | 0.04015liver cancer rs8020034 G A 0.06817 0.46671 0.09366 Choleliths rs8020034 G A -0.0008 0.95098 0.01377 Acute pancreatitis rs8020034 G A 0.02541 0.43216 0.03235 Chronic pancreatitis rs8020034 G A 0.12205 0.00666 0.04498 Pancreatic cancer rs8020034 G A -0.0162 0.81102 0.0678 Ulcerative colitis rs8020034 G A -0.0014 0.9688 0.0357 Crohn's disease rs8020034 G A -0.0145 0.81262 0.06131 Irrtable bowel syndrome rs8020034 G A -0.0125 0.63908 0.02663 Acute appendicitis rs8020034 G A 0.00041 0.97858 0.01536 Colon cancer rs8020034 G A 0.05961 0.15437 0.04185 Rectal cancer rs8020034 G A 0.01507 0.77585 0.05294 |
| Gastroesophageal reflux | rs818415 | T | G | -0.0028 | 0.83464 | 0.01343 | Esophageal carcinoma | rs818415 | T | G | 0.13493 | 0.12046 | 0.08689 | Acute gastritis | rs818415 | T | G | -0.0764 | 0.07252 | 0.04257 | Chronic gastritis | rs818415 | T | G | -0.0134 | 0.53283 | 0.02147 | Gastroduodenal ulcer | rs818415 | T | G | 0.02101 | 0.34087 | 0.02205 | Gastric cancer | rs818415 | T | G | 0.01496 | 0.78872 | 0.05581 | Fatty liver | rs818415 | T | G | -0.0527 | 0.23769 | 0.04465 | Hepatic fibrosis | rs818415 | T | G | 0.08786 | 0.60962 | 0.17207 | Cirrhosis | rs818415 | T | G | 0.0561 | 0.08944 | 0.03303liver cancer rs818415 T G 0.10933 0.15482 0.07684 Choleliths rs818415 T G 0.00322 0.77667 0.01136 Acute pancreatitis rs818415 T G 0.09677 0.00035 0.02707 Chronic pancreatitis rs818415 T G 0.05537 0.12288 0.03589 Pancreatic cancer rs818415 T G -0.0512 0.35665 0.05549 Ulcerative colitis rs818415 T G 0.04841 0.1002 0.02945 Crohn's disease rs818415 T G -0.0811 0.1107 0.05083 Irrtable bowel syndrome rs818415 T G -0.0038 0.8625 0.02194 Acute appendicitis rs818415 T G -0.0127 0.31738 0.01267 Colon cancer rs818415 T G -0.0054 0.87433 0.03432 Rectal cancer rs818415 T G 0.02502 0.56567 0.04356 |
| Gastroesophageal reflux | rs837080 | C | T | -0.0025 | 0.79999 | 0.00987 | Esophageal carcinoma | rs837080 | C | T | 0.05717 | 0.37112 | 0.06392 | Acute gastritis | rs837080 | C | T | 0.00778 | 0.80312 | 0.0312 | Chronic gastritis | rs837080 | C | T | -0.0036 | 0.8189 | 0.0158 | Gastroduodenal ulcer | rs837080 | C | T | 0.01299 | 0.42123 | 0.01616 | Gastric cancer | rs837080 | C | T | -0.0256 | 0.53256 | 0.04106 | Fatty liver | rs837080 | C | T | -0.0361 | 0.27053 | 0.03278 | Hepatic fibrosis | rs837080 | C | T | 0.05758 | 0.64543 | 0.12514 | Cirrhosis | rs837080 | C | T | 0.02428 | 0.31492 | 0.02416liver cancer rs837080 C T -0.0324 0.56613 0.05644 Choleliths rs837080 C T 0.00617 0.45985 0.00835 Acute pancreatitis rs837080 C T -0.0011 0.95393 0.01947 Chronic pancreatitis rs837080 C T -0.0012 0.9628 0.02626 Pancreatic cancer rs837080 C T 0.02186 0.59113 0.0407 Ulcerative colitis rs837080 C T -0.0107 0.62013 0.02155 Crohn's disease rs837080 C T -0.0006 0.98716 0.03716 Irrtable bowel syndrome rs837080 C T -0.0321 0.04649 0.01613 Acute appendicitis rs837080 C T 0.01928 0.03759 0.00927 Colon cancer rs837080 C T 0.01101 0.66415 0.02536 Rectal cancer rs837080 C T 0.01521 0.63618 0.03214 |
| Gastroesophageal reflux | rs892612 | C | A | -0.0044 | 0.74288 | 0.01347 | Esophageal carcinoma | rs892612 | C | A | 0.00514 | 0.95298 | 0.08711 | Acute gastritis | rs892612 | C | A | -0.0257 | 0.54428 | 0.04238 | Chronic gastritis | rs892612 | C | A | -0.0643 | 0.00243 | 0.0212 | Gastroduodenal ulcer | rs892612 | C | A | 0.04508 | 0.03998 | 0.02195 | Gastric cancer | rs892612 | C | A | -0.0191 | 0.73187 | 0.05572 | Fatty liver | rs892612 | C | A | 0.02825 | 0.52804 | 0.04477 | Hepatic fibrosis | rs892612 | C | A | -0.123 | 0.46999 | 0.17026 | Cirrhosis | rs892612 | C | A | -0.0188 | 0.56775 | 0.0329liver cancer rs892612 C A 0.09659 0.20947 0.07696 Choleliths rs892612 C A -0.0175 0.12356 0.01135 Acute pancreatitis rs892612 C A -0.0026 0.92169 0.02653 Chronic pancreatitis rs892612 C A 0.0075 0.83403 0.03579 Pancreatic cancer rs892612 C A 0.01166 0.83262 0.05519 Ulcerative colitis rs892612 C A -0.0744 0.00995 0.02887 Crohn's disease rs892612 C A 0.01088 0.83064 0.05085 Irrtable bowel syndrome rs892612 C A -0.0224 0.30958 0.022 Acute appendicitis rs892612 C A 0.02149 0.08929 0.01265 Colon cancer rs892612 C A -0.0191 0.58017 0.03448 Rectal cancer rs892612 C A -0.0459 0.29537 0.04382 |
[truncated: 238,299 more chars]
